# Ligand Influence on the Performance of Cesium Lead Bromide Perovskite Quantum Dots in Photocatalytic C(sp^3^)–H Bromination Reactions

**DOI:** 10.1021/jacs.4c17013

**Published:** 2025-02-28

**Authors:** Willi M. Amberg, Henry Lindner, Yesim Sahin, Erich Staudinger, Viktoriia Morad, Sebastian Sabisch, Leon G. Feld, Yuxuan Li, Dmitry N. Dirin, Maksym V. Kovalenko, Erick M. Carreira

**Affiliations:** † Department of Chemistry and Applied Biosciences, ETH Zürich, 8093 Zurich, Switzerland; ⊥ NCCR Catalysis, ETH Zürich, 8093 Zurich, Switzerland; ‡ Laboratory for Thin Films and Photovoltaics, Empa—Swiss Federal Laboratories for Materials Science and Technology, CH-8600 Dübendorf, Switzerland

## Abstract

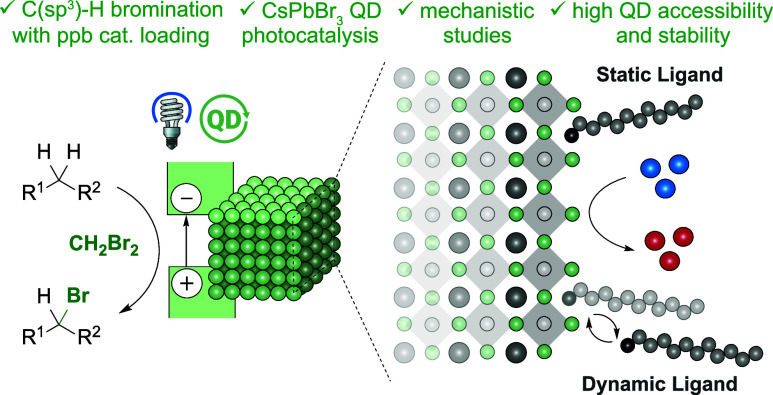

Lead halide perovskite quantum dots (LHP QDs) CsPbX_3_ generate immense interest as narrow-band emitters for displays, lasers, and quantum light sources. All QD applications rely on suited engineering of surface capping ligands. The first generation of LHP QDs employed oleic acid/oleyl amine capping and have found only a limited use in photoredox catalysis. These catalysts have been reported to be unstable and decompose over the course of the reaction, thus reducing turnover numbers (TONs) and limiting their synthetic ability. Herein, the impact of eight distinct surface ligands on monodisperse CsPbBr_3_ QDs is reported, affording a thorough comprehension of their performance in photocatalytic C–H brominations. These efforts yielded QDs operating at extremely low catalyst loadings (<100 ppb) with TONs over 9,000,000 per LHP QD. We emphasize that the optimal catalytic performance requires increased QD surface accessibility without compromising the QD structural and colloidal integrity. Control experiments indicated that well-known photoredox catalysts such as Ir(ppy)_3_, Ru(bpy)_3_Cl_2_, or 4CzlPN are ineffective in the same reaction. Mechanistic studies reveal that the C–Br bond reduction in CH_2_Br_2_ is the rate-limiting step and is likely facilitated through interaction with the CsPbBr_3_ QD surface. This work outlines a holistic approach toward the design of practically useful photocatalysts out of QDs comprising structurally soft QD cores and dynamically bound capping ligands.

## Introduction

**Figure 1:**
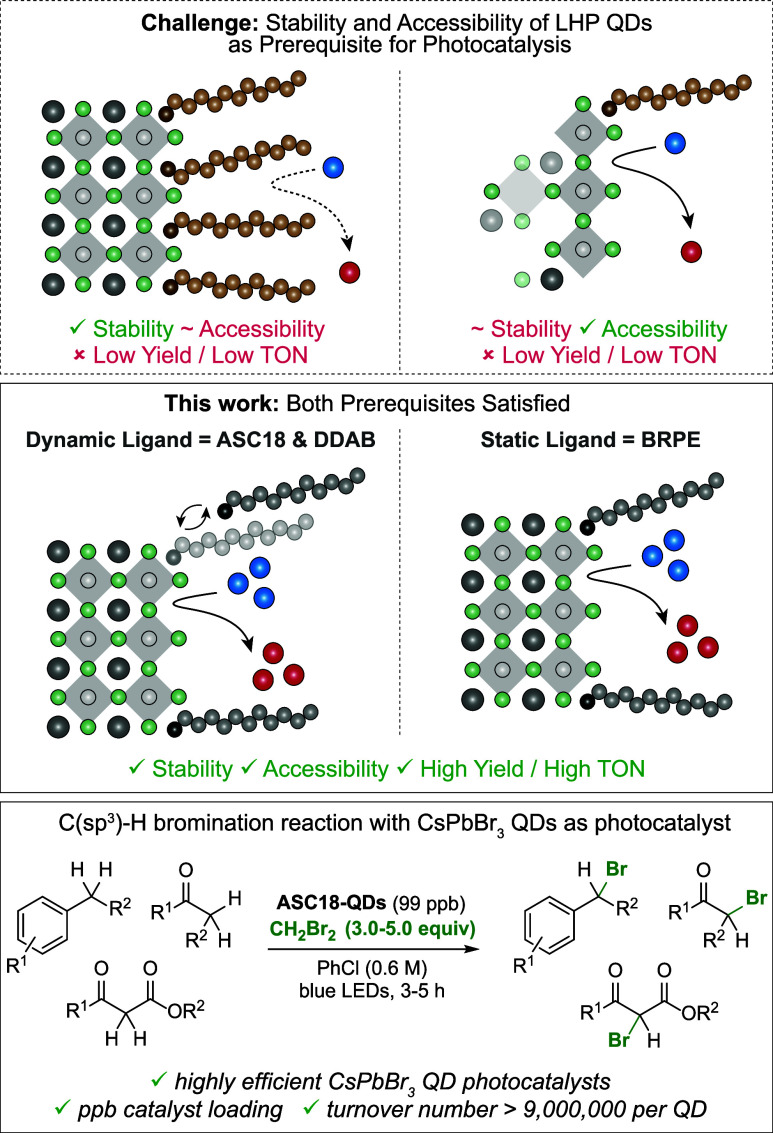
Investigation of LHP QDs in organic synthesis. 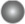 = Cs, 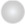 = Pb, 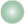 = Br, 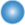 = starting material, and 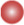 = product. For structures of **BRPE**, **DDAB**, and **ASC18**, see Figure 2.

Colloidal cesium lead halide perovskite quantum dots (LHP QDs) CsPbX_3_ (X = Cl, Br, I), first introduced in 2015, are of immense interest for applications in displays, photodetectors, solar cells, lasers, and quantum light sources.^1^ The physical properties of LHP QDs, in particular large extinction coefficients, near unity photoluminescence quantum yields, small exciton binding energies, and shallow trap states, make them compelling as photocatalysts in the service of organic synthesis.^2^ Furthermore, the LHP QD band gap can be tuned by varying the QD size and composition.^1a^ Photoredox catalysis in organic synthetic methods has been dominated by Ir-, Ru-, and organo-photocatalysts, which typically perform with turnover numbers (TONs) of up to 10,000.^3^ Yet, for the generation of sustainable processes, even higher TONs while maintaining high turnover frequencies are desirable, resulting in lower catalyst loadings. Metals displaying low carbon footprints are particularly preferable (Fe < Pb < Cu < Ni < 20 kg CO_2_/kg metal vs Pt group metals ≈3000 kg CO_2_/kg metal).^4^ Herein, we report detailed studies of LHP QDs with eight distinct surface capping ligands to elucidate their photochemical performance as well as moisture and solvent stability and provide a blueprint for developing photoredox reactions with LHP QDs. The C–H brominations of alkylarenes, ketones, and β-ketoesters were chosen as model reactions. A salient feature of LHP QDs studied herein is their exceptionally high efficiency with TONs of >9,000,000 per QD at ppb catalyst loading, whereas traditional photocatalysts did not yield any products.^5^ We attribute this difference to the accessibility of the LHP QD surface, to allow for a photochemical reaction step to proceed efficiently (Figure 1).

Well-performing LHP QD photocatalysts were observed to provide surface accessibility by either of two scenarios: the use of dynamically binding ligands or static ligands, which cover the LHP QD partially. Taken together, this study highlights the unique features and advantages of LHP QDs, which are poised to be harnessed in organic synthesis.

Oleic acid/oleyl amine has been used as a surface ligand system for LHP QDs applied in photoredox catalysis. The reported transformations include the homocoupling of benzyl bromides, alkylation of aldehydes, annellation of benzylidene malononitriles, bromination of electron rich arenes and activated olefins, as well as the bromination of three benzylic substrates in low yield, among others.^6^ In their application as photocatalysts, these QDs undergo irreversible structural degradation both during storage and over the course of the reaction.^2b,7^ Furthermore, these LHP QDs have usually been prepared with large size distributions, resulting in a range of different redox potentials and poorly defined catalytic systems.^6c,6d,6i^ We envision that future applications in organic synthesis would significantly benefit from the availability of LHP QDs with narrow size distributions that are stable over an extended time.

Since the discovery of LHP QDs, the prevailing notion has been to seek effective stabilization by high coverage of tightly binding ligands.^7,8^ Over the course of this study, we have noted that for catalysis to occur more efficiently, ligand-capped LHP QDs need to exhibit a proper balance between stability and surface accessibility.^9^ This necessary condition can be met in two different ways: LHP QDs with reversibly dissociating surface ligands or, alternatively, nondissociating ligands at a persistent surface coverage.

## Results and Discussion

Selective bromination of activated C–H bonds has been a reoccurring challenge in industry and academia.^10^ We envisioned the use of LHP QDs as photoredox catalysts to provide new and efficient routes for the monobromination of organic substrates. We decided to investigate ethylbenzene (**1a**) as a model substrate. As a bromine source, CH_2_Br_2_ was chosen because it is an inexpensive and abundant commodity chemical produced in 500–9000 tons per year in the United States alone.^11^ It is less mass intensive than NBS, generally considered inert, and has found industrial applications, e.g., as solvent and water treatment products.^12^ As a Br transfer reagent, CH_2_Br_2_ remains underappreciated.^13^

**Figure 2:**
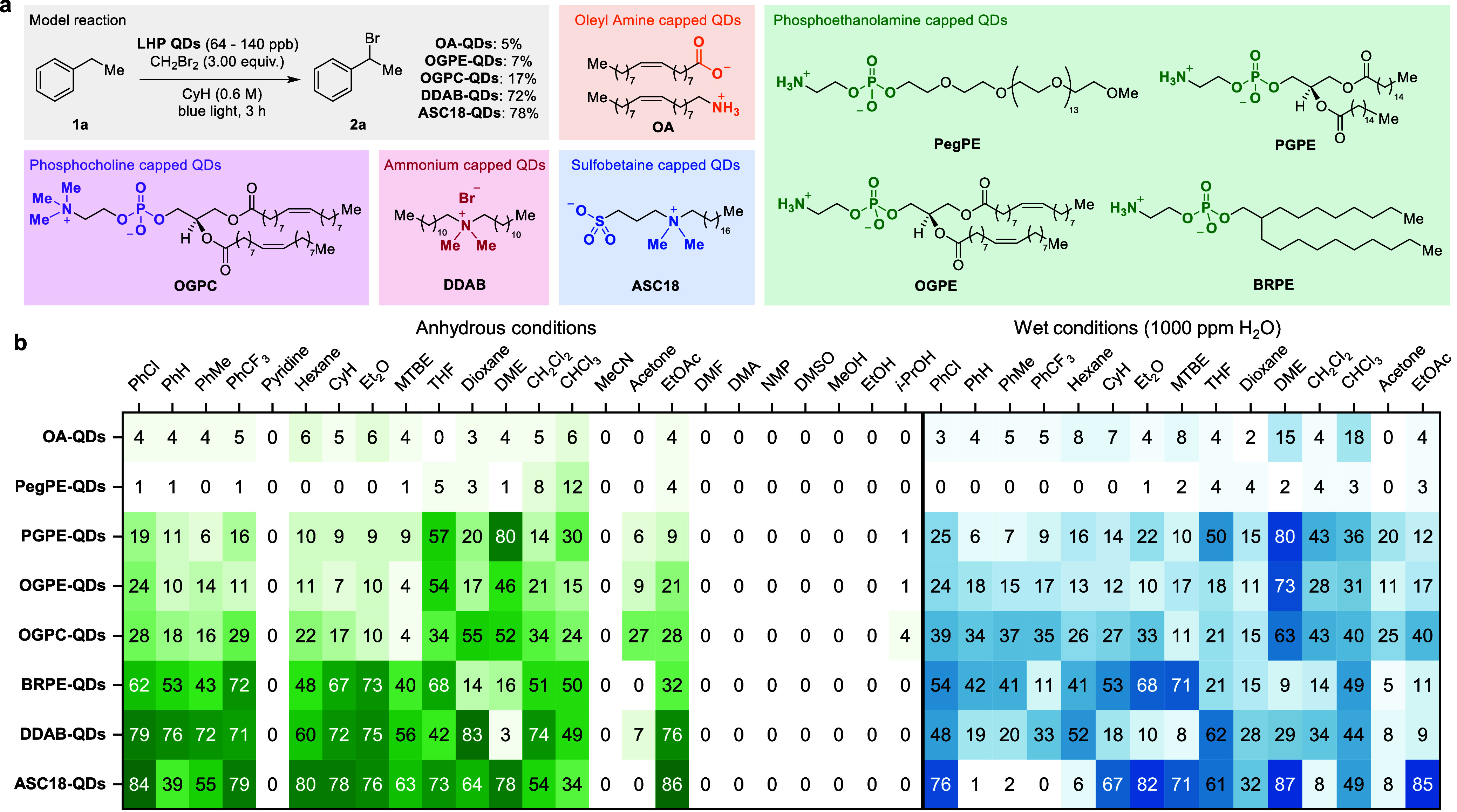
Photoredox performance of LHP QDs. (a) Benzylic bromination of ethylbenzene and structure of the five classes of ligands investigated in this study. (b) The green heat map displays ^1^H NMR yields with mesitylene as an internal standard for the benzylic bromination of PhEt (**1a**) under anhydrous conditions in 24 different solvents. The blue heat map displays ^1^H NMR yields with mesitylene as an internal standard for the benzylic bromination of PhEt (**1a**) in the presence of 1000 ppm of H_2_O in 15 different solvents as part of a moisture study.

Some of us have recently reported sulfobetaine-, phosphocholine-, and phosphoethanolamine-based zwitterionic ligands as well as quaternary ammonium ligands for improved LHP QD stability compared to oleic acid/oleyl amine-capped ones.^7,8,14^ However, no studies are available that examine the influence of these surface ligands on the photoredox performance of LHP QDs. At the onset of the study, we decided to probe the utility of commercially available ligands with the objective of covering a broad spectrum of different head groups. In five separate experiments, ethylbenzene (**1a**, 0.60 mmol) and CH_2_Br_2_ (1.8 mmol) were dissolved in 1.0 mL anhydrous cyclohexane and **OA-QDs** (oleic acid/oleyl amine capped), **OGPE-QDs** (phosphoethanolamine capped), **ASC18-QDs** (sulfobetaine capped), **DDAB-QDs** (ammonium capped), or **OGPC-QDs** (phosphocholine capped) were added as photocatalysts, respectively (Figure 2a). The use of LHP QDs as catalysts at very low loadings would constitute an important contribution to the synthetic methodology. Accordingly, we examined catalyst loadings of 6.4 to 14 × 10^–6^ mol % (64–140 ppb; for details, see the Supporting Information). The reactions were irradiated with blue light (λ_max_ = 446 nm, 350 W blue-LED photoreactor; for technical details, see the Supporting Information) for 3 h.

Intriguingly, α-bromo ethylbenzene (**2a**) was afforded in a wide range of yields 5% (**OA-QDs**), 7% (**OGPE-QDs**), 17% (**OGPC-QDs**), 72% (**DDAB-QDs**), and 78% (**ASC18-QDs**). The fact that the reaction mixtures containing **OA-QDs**, **OGPE-QDs**, and **OGPC-QDs** were not photoluminescent after the reaction can be attributed to irreversible catalyst degradation. Only the reactions containing **ASC18-QDs** and **DDAB-QDs** remained photoluminescent. In the absence of either an LHP QD or light, no reaction was observed. Similarly, no conversion of PhEt was detected when only **ASC18** or **DDAB** was employed instead of the corresponding QDs. To benchmark LHP QD performance against photocatalysts which cover a wide range of excited-state reduction potentials, bromination of **1a** was attempted with 1.00 mol % of *N*,*N*,*N*,*N*-tetramethylbenzene-1,4-diamine, Ir(ppy)_3_, 12-phenyl-12*H*-benzo[5,6][1,4]thiazino [2,3-*b*]quinoxaline, Ru(bpy)_3_Cl_2_, 4CzlPN, and Eosin Y. None promoted the transformation as only traces (<1%) of **2a** were observed in the ^1^H NMR spectra of the unpurified reaction mixtures with the mass balance being remaining starting material. We were intrigued by the fact that LHP QDs have unique properties that are unmatched by those of established catalysts. Furthermore, the stark differences observed in the initial experiments led us to investigate the effect of ligands on catalyst performance and stability of CsPbBr_3_ QDs.

Adding to the five different head groups examined in the preliminary experiment, we investigated 2-ammonioethyl (poly(ethylene)glycol) phosphate (**PegPE**), 2-ammonioethyl (dipalmitoyl-*sn*-glycero) phosphate (**PGPE**), and 2-ammonioethyl (2-octyldodecyl) phosphate (**BRPE**) to identify the influence of different tail groups (saturated, unsaturated, and heteroatom rich). With this selection, we aimed to gain insights into the effect of ligand structure on the integrity of the QD over the course of the reaction, along with its catalyst performance.

LHP QD structural integrity is susceptible to solvent effects.^15^ Consequently, we studied the influence of 24 organic solvents commonly employed in synthesis. In these experiments, ethylbenzene (0.60 mmol), 64–177 ppb LHP QD (see the Supporting Information for details about QD colloids), and 3.0 equiv of CH_2_Br_2_ were added to 1.0 mL of the solvent. After 3 h of blue-light irradiation, the NMR yields of **2a** were determined (Figure 2b). The study revealed that sulfobetaine-capped **ASC18-QDs** performed best in the widest range of solvents (>50% yield in 12 solvents), followed by ammonium-capped **DDAB-QDs** (>50% yield in 11 solvents) and phosphoethanolamine-capped **BRPE-QDs** (>50% yield in 7 solvents). For **PGPE-QDs** (>50% yield in 2 solvents), **OGPE-QDs** (>50% yield in 1 solvent), **OA-QDs** (<10% yield in every solvent), and **PegPE-QDs** (<5% yield in every solvent), poor performance was observed. Very polar solvents like DMF can dissolve LHP QDs which was also observed in our investigations with the notable exceptions of EtOAc, THF, and DME.^16^

Moisture has generally been considered detrimental for LHP QD stability as water can dissolve CsPbBr_3_; however, studies investigating the influence of water in ligand-capped LHP QD photocatalysis remain elusive.^17^ Consequently, we set out to investigate the benzylic bromination of ethylbenzene in the presence of 1000 ppm of H_2_O (1 μL/1 mL solvent) (Figure 2b). In PhCl and ethers, the added water had minimal influence on the product formation catalyzed by **ASC18-QDs** and **DDAB-QDs** (for more details, see the Supporting Information). These examples highlight that water is not necessarily detrimental to QD photoredox catalysis, allowing their use under ambient conditions (vide infra). When considering new avenues for future photocatalyst design for organic synthesis, we conclude that the QD performance can be tuned with surface ligands. Especially, hydrocarbon-based side chains, as seen in **BRPE**, **DDAB**, and **ASC18**, appear to be advantageous.

The observation that **PegPE-QDs** performed worst among all investigated QDs came as a particular surprise, considering that **PegPE-QDs** are colloidally stable in the widest range of solvents (polar and apolar).^8^ Evidently, broad colloidal stability of QDs does not necessarily translate into well-performing photocatalysts. Colloidal instability may be caused by at least two pathways, namely, LHP QD degradation by solvents or antisolvent-induced precipitation of LHP QDs via interactions between surface ligands without altering their core integrity.^16^ For example, **OA-QDs** aggregated to bulk material in Et_2_O and MTBE, which shut down catalytic activity of the QD. By contrast, the use of antisolvents as reaction media for **PGPE-QDs**, **OGPE-QDs**, **OGPC-QDs**, **BRPE-QDs**, **DDAB-QDs**, and **ASC18-QDs** led to turbidity, but the product still formed in up to 86% yield (for details, see the Supporting Information). Surprisingly, this indicates that surface-ligand aggregates of LHP QDs retain catalytic activity as long as their core structure remains intact.

**Figure 3:**
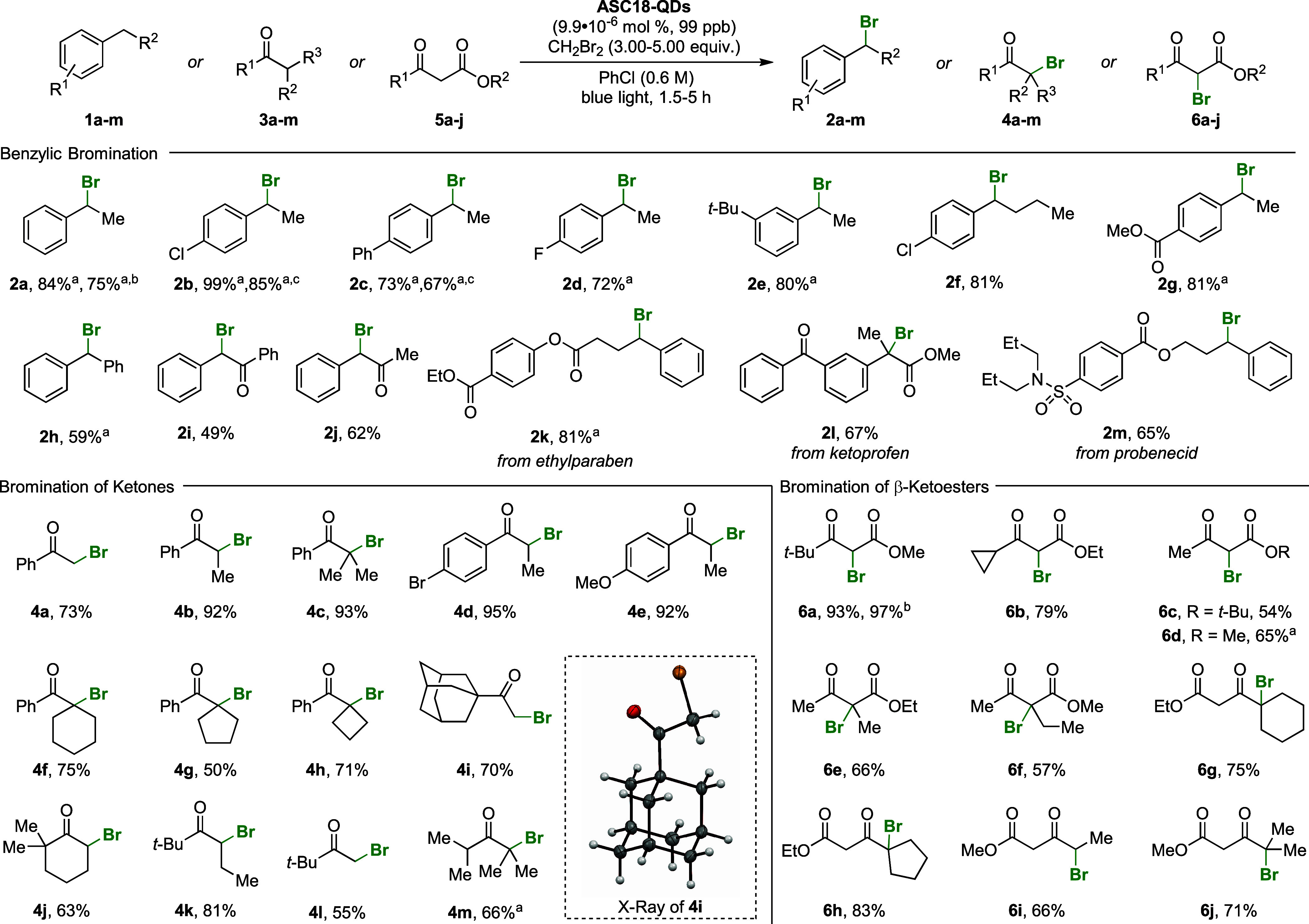
Substrate scope. Reaction conditions: substrate (0.60 mmol), CH_2_Br_2_ (1.8 mmol), **ASC18-QDs** (9.9 × 10^–6^ mol %, 99 ppb) in PhCl (1.0 mL), irradiation in a 350 W photoreactor for 1.5–5 h. ^a^Yield obtained by ^1^H NMR with mesitylene as an internal standard. ^b^Conducted on a 10 mmol scale. ^c^Conducted without exclusion of air and moisture. The CCDC deposition number for **4i** is 2404075.

We subsequently focused our study on **ASC18-QDs** for the photochemical bromination, as they provided the best compromise between stability and activity. We initially investigated the scope of the alkylarene substrates (Figure 3). Guided by the data in Figure 2, and a preliminary screening, we chose PhCl as the solvent for the substrate scope. When PhEt was subjected to the optimized conditions (3.0 equiv of CH_2_Br_2_ and 99 ppb **ASC18-QDs** in 0.6 M PhCl) and irradiated for 3 h, bromide **2a** was observed in 84% yield. To highlight the synthetic utility of our transformation, bromination of **1a** was conducted on a 10 mmol scale (6.0 M). Gratifyingly, **2a** was formed in a 75% yield. The reaction conditions tolerated differently substituted arenes, affording **2b**–**2j** in 49–99% yield.

An ethylparaben-derived substrate yielded **2k** in 81%, and bromides **2l** and **2m**, derived from the active pharmaceutical ingredients ketoprofen and probenecid, were isolated in 67 and 65% yield, respectively. The consistently high performance of 99 ppb **ASC18-QDs** throughout the reaction scope is remarkable, with TONs reaching as high as 9,650,000 per LHP QD. Additionally, we were able to effect the benzylic bromination reaction without the exclusion of air and moisture with a minimal decrease in yield for **2b** and **2c** (for details, see the Supporting Information).

The performance of **ASC18-QDs** prompted us to investigate the α-bromination of ketones under the same conditions next. Acetophenone was successfully converted to α-bromo acetophenone **4a** in 73% yield (Figure 3). Other aryl alkyl ketones were also successfully brominated, giving rise to secondary or tertiary bromides **4b–4h** in 50–95% yield. Dialkyl ketones were amenable to the reaction conditions, affording **4i–4m** in 66 to 81% yield.

From our previous work on photocatalytic cyclopropanation of unactivated olefins with α-bromo-β-ketoesters, we were aware that monobromination of β-ketoesters often suffers from dibrominated side products.^10b,18^ Consequently, β-ketoesters were also subjected to the reaction conditions (Figure 3). Methyl 2-bromo-4,4-dimethyl-3-oxopentanoate (**6a**) was isolated in 93% yield. On a larger scale (10 mmol), **5a** was brominated in 97% yield without formation of the dibrominated product. The reaction tolerated different ester groups, giving rise to **6b** and **6c** in 79% and 54% yield, respectively. Methyl 3-oxobutanoate (**5d**) was brominated in 65% yield. α-Alkyl β-ketoesters were also amenable to the reaction conditions, affording **6e** and **6f** in 66% and 57% yield, respectively.

When ethyl 3-cyclohexyl-3-oxopropanoate (**5g**) was subjected to 99 ppb **ASC18-QDs** and CH_2_Br_2_ (3.0 equiv) in PhCl and irradiated for 5 h with blue light, no α-bromo-β-ketoester was obtained. Instead, selective bromination at the γ-position was observed, and **6g** was isolated in 75% yield. Bozzelli and co-workers have shown that α-alkyl substitution of a ketone diminishes the α-C–H bond dissociation energy by up to 8 kcal/mol.^19^ Since γ-brominated products are of interest as synthetic intermediates in drug and agrochemical research, further substrates were investigated.^20^ γ-Alkyl-β-ketoesters were subjected to the reaction conditions and only γ-bromo-β-ketoesters **6g–6j** were isolated in 66–83% yield.

**Figure 4:**
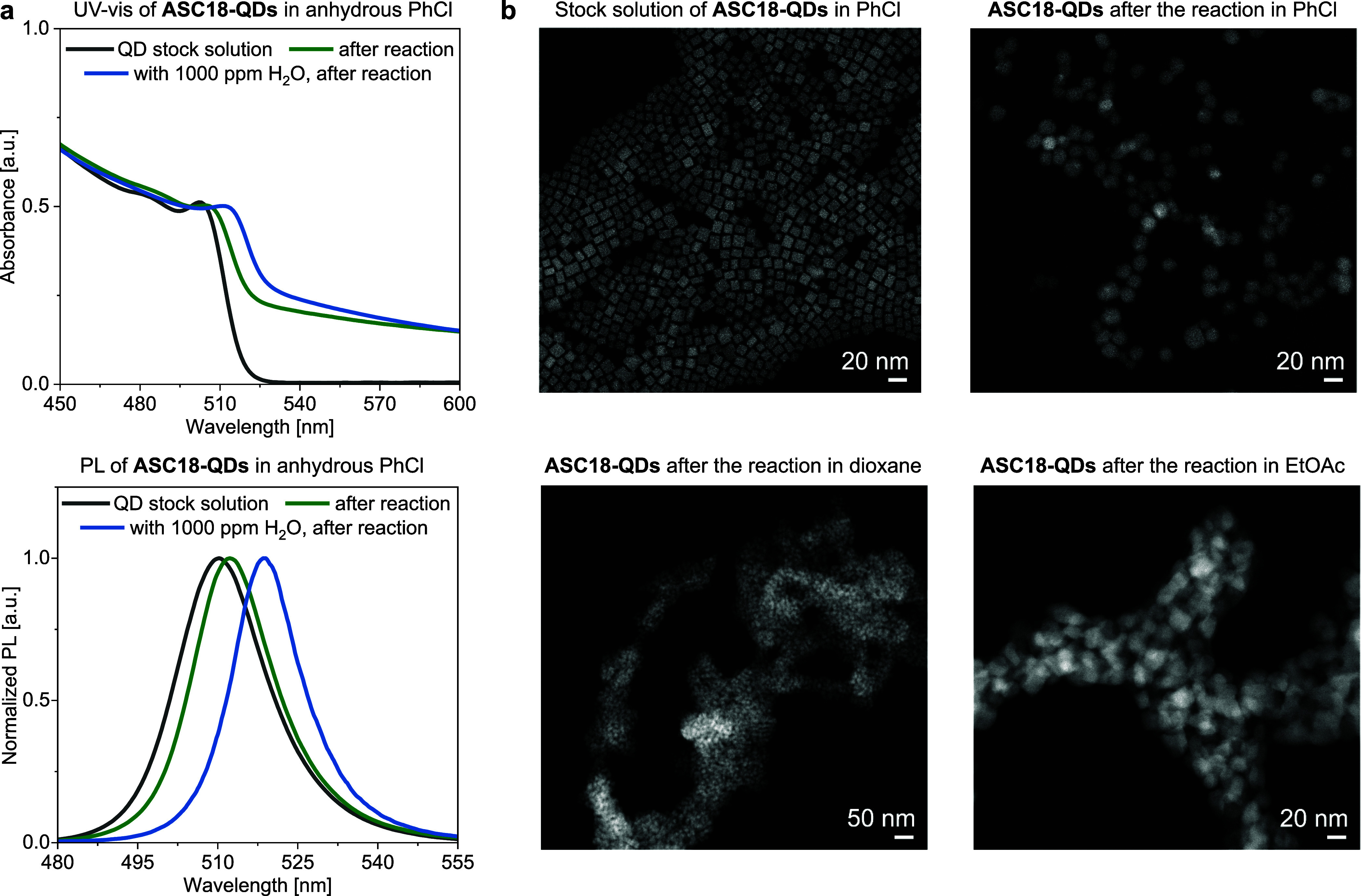
Characterization of **ASC18-QDs** post reaction. (a) UV–vis and photoluminescence (PL) spectra of **ASC18-QDs** before and after the reaction in PhCl. (b) Scanning transmission electron microscopy (STEM) images of **ASC18-QDs** in PhCl, dioxane, and EtOAc after the bromination reaction. The unpurified reaction mixture was used to prepare the TEM grids.

With the substrate scope in hand, we proceeded to investigate whether the LHP QDs change in size and shape over the course of the reaction. Because **ASC18-QDs** were the most general catalyst in our solvent study, we studied the QDs post-reaction with UV–vis and photoluminescence spectroscopy (Figure 4a). The spectroscopic analysis revealed only minute shifts in the absorbance and photoluminescence maxima (compared to native **ASC18-QDs**; for a full comparison, see the Supporting Information). This indicates that the average particle size does not significantly change during the reaction.^7^ When the benzylic bromination was conducted in the presence of 1000 ppm of water in PhCl, a significant red shift was observed in UV–vis (+9 nm) and PL (+8 nm), indicating particle growth.

To further assess the quality of the LHP QD catalyst at the end of the reaction, we recorded transmission electron microscopy (TEM) images of the residuals from all anhydrous reactions which gave >10% yield catalyzed by **ASC18-QDs** (for full comparisons including **DDAB-QDs** and **BRPE-QDs**, see the Supporting Information). Interestingly, there was no direct correlation between the product yields and QD morphology after the reaction. As an example, **ASC18-QDs** catalyze the formation of **2a** in PhCl, dioxane, and EtOAc in comparable yield (84%, 64%, and 86%, respectively). The STEM images after the reaction showed QDs of varying quality (Figure 4b). While in anhydrous PhCl the size of **ASC18-QDs** were retained, etching to smaller or sintering to larger particles was observed in dioxane and EtOAc. We conclude that a change in shape during the reaction is not necessarily detrimental to the catalyst’s performance.

**Figure 5:**
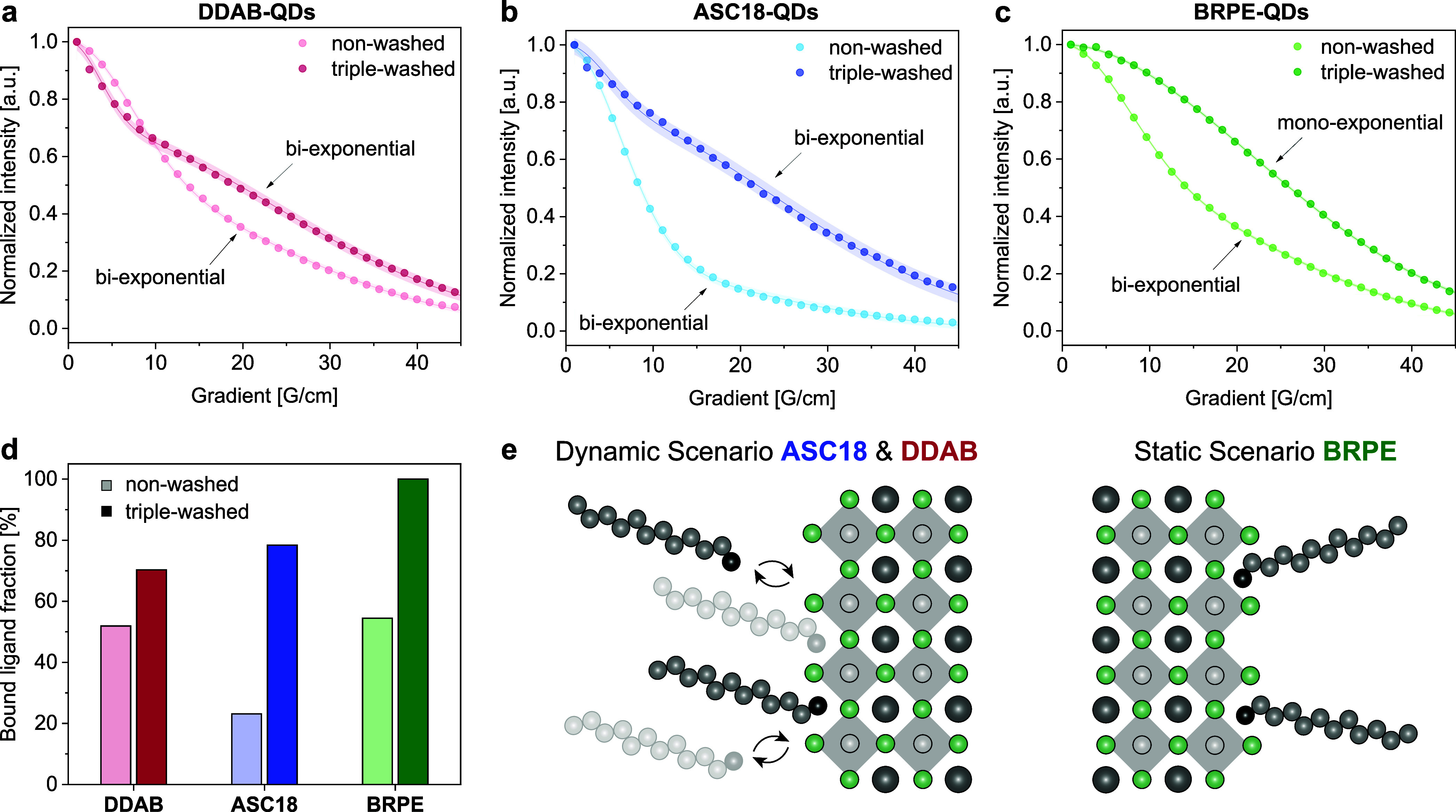
Investigation of ligand dynamics. (a–c) DOSY traces for **DDAB-**, **ASC18**-, and **BRPE**-capped QDs before and after three washing cycles. The experiments were carried out using a double-stimulated echo sequence with a diffusion time (Δ) of 200 ms and a gradient time (δ) of 4 ms.^24^ (d) Ratio between the fast and slow components of the biexponential fit outlining the fraction of the bound ligand on the QD. (e) Graphical representation of the dynamic and static scenario.

A prerequisite for catalysis is the proximity between reactants and catalysts. However, the presence of surface-bound ligands on LHP QDs required for stabilization can have the undesired consequence of hampered accessibility to the surface by the reactants.^9c^ These two opposing conditions (surface coverage for stability vs surface accessibility for reactivity) can be nonetheless satisfied in two scenarios: (1) QDs with nondissociating ligands at some optimal coverage to ensure stability and persistent open sites or (2) QDs with ligands that reversibly dissociate in a process that enables surface access. It was unclear which of these scenarios was operative for the various QDs in the solvent study (Figure 2). We envisioned that both scenarios could be distinguished by diffusion-ordered NMR spectroscopy (DOSY, Figure 5a–c).^21^

DOSY allows for the separation of ^1^H NMR signals of different species based on their diffusion coefficients. The measurement consists of a series of spin echo experiments paired with pulsed field gradients of increasing strength. The application of the gradient pulses results in diffusion-dependent ^1^H NMR signal decay which can be analyzed.^22^ For ligand-capped QDs, two distinct species could be differentiated: free ligands in solution displaying fast diffusion (10^–9^ m^2^/s) and ligands bound to the LHP QD surface showing slow diffusion (10^–11^ m^2^/s).^7^

DOSY experiments were carried out for the three well-performing ligands with characteristically different headgroups (**BRPE**, **DDAB**, and **ASC18**) to elucidate whether the ligands are dissociating (dynamic vs static). In the experiment, the LHP QDs were freshly prepared with excess ligand, precipitated, centrifuged, and redispersed in deuterated cyclohexane. Acquisition of the first set of NMR data (labeled non-washed) was followed by QD precipitation, centrifugation, and washing (3 times; for details, see the Supporting Information). A second set of NMR data was acquired for comparison (labeled as triple-washed). The acquired data of the non-washed LHP QDs was fitted first by using a two-component version of the Stejskal–Tanner equation (eq 1). All three samples displayed a two-component decay (biexponential fit) of the ^1^H NMR signal corresponding to the terminal methyl groups of the ligand tails (Figure 5a–c).^23^
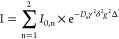
1

The fast component, which dominates the signal decay up until a gradient of 20 G/cm, has a diffusion coefficient on the order of 10^–9^ m^2^/s, consistent with the free ligand in solution below its critical micelle concentration. The slow component fits a diffusion coefficient of 5 × 10^–11^ m^2^/s consistent with the solvo-dynamic radius of an LHP QD. We define the ratio between the fast and slow components as the ratio of the free to bound ligand (bound ligand fraction, Figure 5d). The bound ligand fraction of the non-washed **DDAB**, **ASC18**, and **BRPE** samples was determined to be 52% (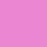), 23% (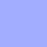), and 54% (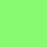), respectively. We concluded that for the nonwashed case, not all ligands are bound to the QD surface, which can be attributed to excess ligands used in the LHP QD synthesis.

After three washing cycles, the DOSY data of **DDAB-QDs** and **ASC18-QDs** still displayed biexponential behavior with fast and slow diffusing components (triple-washed, Figure 5a,b). As shown in Figure 5d, this corresponds to bound ligand fractions of 70% for **DDAB** (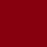) and 78% for **ASC18** (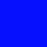). As the washing steps removed excess **DDAB** and **ASC18** from the LHP QD solution, the presence of the free ligand (29% and 22%, respectively) is the result of the re-establishment of equilibria between bound and free ligands. This unveils the dynamic nature of **DDAB** and **ASC18** which can generate catalytically active surface sites (Figure 5e). Triple-washed LHP QDs capped with **BRPE** show slow monoexponential signal decay in the DOSY experiment (Figure 5c). With a bound ligand fraction of 100% (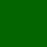), no ligands are dissociating from the surface (Figure 5d,e), which corresponds to scenario one.

With these observations, we conclude that high-yielding QDs fall into one of two categories: Triple-washed **ASC18-QDs** (78% yield) and **DDAB-QDs** (72% yield) are covered with ligands that are able to generate open surface sites via reversible, dynamic dissociation, which allows for substrates to approach the QD surface. **BRPE** (67% yield) has persistent ligand coverage that retains active surface sites. In all three cases, the ligand sufficiently stabilizes the LHP QD for catalysis to occur. In contrast, the low-yielding QDs (from Figure 2), **OA-QDs**, **OGPE-QDs**, and **OGPC-QDs**, do not fulfill the stability criteria under the reaction conditions (for details, see the Supporting Information). For **PegPE-QDs**, we hypothesize that the surface is inaccessible because the oxygen-rich ligand tail interacts with the QD surface, further covering it in the process.

**Figure 6:**
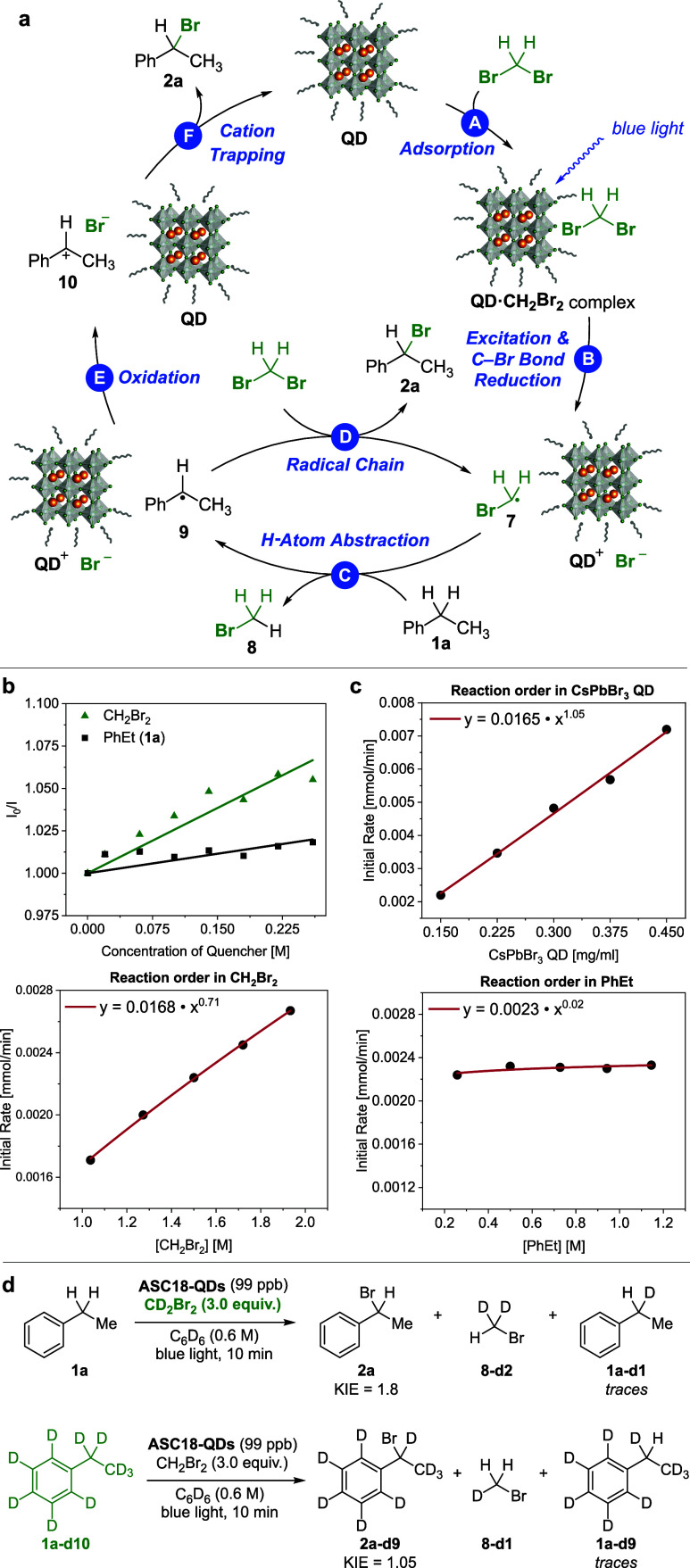
Mechanistic studies. (a) Proposed catalytic cycle. (b) Stern–Volmer quenching studies. (c) Reaction order for each reactant. (d) Kinetic isotope effect (KIE) for CD_2_Br_2_ vs CH_2_Br_2_ and **1a** vs **1a–d10**.

At the onset of our mechanistic investigation, we were intrigued that a range of photoredox catalysts (vide supra) did not promote C–H bromination in our hands. While for the related oleic acid/oleyl amine-capped quantum dots an excited-state reduction potential in the range of −1.15 V vs SHE has been measured (Pt electrode, 50 mM TPAB in 4:1 MeCN/PhMe), numerous reduction potentials for CH_2_Br_2_ can be found in the literature.^25^ Fedurco and co-workers attributed the differences in the reduction potential to solvent effects, which influence the overpotential during the electron transfer from the electrode to CH_2_Br_2_. For instance, the authors report a reduction potential of −2.60 V in DMF/0.1 M TBAPF_6_ vs Fc/Fc^+^ and −1.05 V in H_2_O/1 M NaClO_4_ vs NHE.^26^ The reduction potential of oleic acid/oleyl amine-capped QDs would lie within the range of investigated photocatalysts. We concluded that LHP QDs may have a unique property for the reaction to proceed. Given the heterogeneous nature of LHP QDs, we hypothesize that CH_2_Br_2_ may adsorb onto the CsPbBr_3_ QD surface to facilitate subsequent C–Br bond cleavage (Figure 6a, Step A and B).^6k,27^ Conventional homogeneous photocatalysts, however, lack an analogous surface, hampering reductive C–Br bond cleavage.^25,26^ When Stern–Volmer quenching studies were conducted with **ASC18-QDs**, the observed quenching of the excited-state QD photocatalyst, albeit weak in intensity, suggests energy or charge transfer from **ASC18-QDs** to CH_2_Br_2_ (Figure 6b).

We determined the reaction order of **ASC18-QDs**, CH_2_Br_2_, and PhEt by measuring the initial rate at varying reactant concentrations (Figure 6c). We found that the reaction is first order in **ASC18-QDs**, while CH_2_Br_2_ has a calculated order of 0.71. To explain the subfirst-order value of the latter, a number of effects can be hypothesized: Surface saturation of **ASC18-QDs** by CH_2_Br_2_ and thermodynamic equilibria in the adsorption of CH_2_Br_2_ could alter the reaction rate at varying CH_2_Br_2_ concentrations. The rate could further be limited by the diffusion rate of CH_2_Br_2_ to the surface of **ASC18-QDs**.^28^ Alternatively, with increasing CH_2_Br_2_ concentration, the physicochemical properties of the reaction media change, which could expedite partial catalyst decomposition. The reaction order in ethylbenzene is zero.

KIE experiments (Figure 6d) from parallel reactions (initial rate; for details, see the Supporting Information) furthermore revealed a secondary KIE for CD_2_Br_2_ (KIE = 1.8), while **1a–d10** displayed a negligible KIE of only 1.05. From these experiments, we concluded that the C–Br bond reduction of CH_2_Br_2_ is the rate-determining step.^29^

Notably, in the course of the KIE study, we also observed the formation of CD_2_HBr and **1a–d9**. This is consistent with a bromomethyl radical abstracting a benzylic H atom in Step C to generate benzyl radical **9**. The latter can subsequently proceed through two productive pathways: Br-atom abstraction from CH_2_Br_2_ (Step D), which leads to product formation consistent with a radical chain mechanism, or oxidation to a benzyl cation (Step E), which is subsequently trapped by bromide (Step F) and corresponds to a closed catalytic cycle.

Radical quenching experiments were conducted next. TsCN (1.00 equiv) was employed as a radical scavenger, as TEMPO (1.00 equiv) was not tolerated by **ASC18-QDs**.^30^ In the experiment, α-cyanoethylbenzene and bromoacetonitrile were observed in the ^1^H NMR spectrum of the unpurified reaction mixture (for details, see the Supporting Information). These products substantiate the formation of intermediates **7** and **9** in the catalytic cycle. Lastly, we determined the reaction quantum yield for the benzylic bromination of **1a** as Φ = 0.011 (for details, see the Supporting Information). This value is consistent with a closed catalytic cycle but does not rule out a radical chain mechanism.

## Conclusions

We have developed a novel photocatalytic bromination reaction that allowed us to study the photocatalytic performance of CsPbBr_3_ QDs. To demonstrate the synthetic utility, a wide variety of functionalized substrates (alkyl benzenes, ketones, and β-ketoesters) were brominated. Interestingly, for certain β-ketoesters, high selectivity for γ-bromination over α is observed. The salient features of the transformation are its air and moisture tolerance and functional group compatibility. Additionally, ligand-capped CsPbBr_3_ QDs exhibit high activity as photocatalysts with loadings in the <100 ppb range, exceeding the activity of conventional photocatalysts substantively. We hypothesize that CsPbBr_3_ QDs exhibit surface effects that separate them from established small-molecule photocatalysts. Our comparative study featuring eight different ligand-capped LHP QDs revealed that colloidal stability and quality of the QDs after the reaction are only limited indicators for QD photocatalyst performance. DOSY experiments revealed two possible scenarios for generating catalytically active LHP QDs while maintaining stability: dynamic ligand coverage or use of static ligands at an optimal persistent surface coverage. Within these scenarios, ligand and LHP QD design offers opportunities for the next generation of photocatalysts in synthesis. We expect that the insights we provided in this work will lead to new applications with lead halide perovskite QDs in photoredox catalysis.

## Supporting Information Available

Experimental procedures, quantum dot preparation and characterization, TEM images, UV–vis and photoluminescence spectroscopy, ^1^H NMR spectra, ^13^C NMR spectra, IR, and HRMS (PDF)
