# Supplementary material for: Ligand Influence on the Performance of Cesium Lead Bromide Perovskite Quantum Dots in Photocatalytic C(sp3)–H Bromination Reactions
Source: J Am Chem Soc. 2025 Feb 28;147(10):8548–58. doi: 10.1021/jacs.4c17013 (PMC11912481; doi:10.1021/jacs.4c17013)
Supplement: Supplementary file 1 — ja4c17013_si_001.pdf [file ja4c17013_si_001.pdf]

Supporting Information

Ligand Influence on the Performance of Cesium Lead Bromide Perovskite Quantum Dots in Photocatalytic $C(sp^3)$-H Bromination Reactions

Willi M. Amberg,‡ Henry Lindner,‡ Yesim Sahin,‡ Erich Staudinger, Viktoriia Morad, Sebastian Sabisch, Leon G. Feld, Yuxuan Li, Dmitry N. Dirin, Maksym V. Kovalenko,* and Erick M. Carreira*

Table of Contents

1. General remarks 3
2. Instruments for $CsPbBr_3$ quantum dots characterization 6
3. Photoreactor set-up 8
4. Quantum dots investigated in this study 9
5. Synthesis of ligand capped $CsPbBr_3$ quantum dots 10
6. Calculation of catalyst loading 17
7. Performance of different ligand capped $CsPbBr_3$ QDs as photocatalysts 19
8. Full comparison of TEM images 23
9. Additional STEM images 39
10. UV-Vis and PL spectra of ASC18-QDs after the reaction 41
11. Diffusion ordered spectroscopy (DOSY) experiments 63
12. Stern-Volmer quenching studies 66
13. Further mechanistic studies 68
14. General procedures for C–H bromination 78
15. Substrate scope 80
16. Synthesis of starting materials 116

17. ^{1}H NMR and ^{13}C NMR spectra 118

18. X-Ray crystallographic data 149

19. References 151

1. General remarks

Procedure

Unless otherwise stated, all reactions were carried out in a nitrogen filled glove box. Reactions in the 350 W photoreactor were performed in 13x40 mm screw-thread vials (ROFRA GmbH, Mat. Nr. 14.020.92) that were charged with a magnetic stirrer bar (PTFE, 3x8 mm, Semadeni Plastics Group, Art. 244) and sealed with a screwcap. Reactions at 10.0 mmol scale were conducted in 38.2x22 mm crimp-neck vials (Labsolute, Art. Nr. 7615908) that were charged with a magnetic stirrer bar (PTFE, 6x15 mm, Semadeni Plastics Group, Art. 249) and sealed with a crimp cap (infochroma ag, 8087-Bu).

Chemicals

Unless otherwise stated, reagents and solvents were purchased from commercial suppliers (ABCR, Acros, Sigma Aldrich, Fluka, TCI, Strem, Alfa, Combi-Blocks or Fluorochem) and used as received. For flash column chromatography Sigma-Aldrich silica gel sorbent (high purity grade (9385), 230-400 mesh particle size, pore size 60) was used as a stationary phase.

Thin-Layer Chromatography

Analytical thin layer chromatography (TLC) was performed on glass plates from Supelco® (TLC silica gel 60 F_{254}: 25 glass plates, 20 x 20 cm) and visualized via exposure to ultraviolet light (254 nm or 365 nm) or TLC stain (aqueous potassium permanganate solution followed by heating or aqueous ceric ammonium molybdate solution followed by heating).

Nuclear Magnetic Resonance Spectroscopy

All NMR spectra were measured in deuterated solvents at room temperature with a Bruker Avance 400 (400 MHz, equipped with 9.4 T magnet, BBFO probe and avance neo console), Bruker Ascend 400 (400 MHz, equipped with 9.4 T magnet, BBFO probe, and avance III console), Bruker Ultrashield 400 (400 MHz, equipped with 9.4 T

magnet, BBFO probe, and avance III console), Oxford 400 (400 MHz, equipped with 9.4 T magnet, BBFO probe, and avance III HD console) or Bruker Avance 500 (500 MHz, equipped with 11.7 T magnet, BBFO probe, and avance neo console). Chemical shifts are referenced to the solvent residual signal ($CDCl_3$, ^{1}H: δ = 7.26 ppm, ^{13}C: δ = 77.16 ppm) and reported in parts per million (ppm). The following abbreviations are used in reporting NMR data: s = singlet, d = doublet, t = triplet, q = quartet, b = broad, dd = doublet of doublets, m = multiplet, etc.

High-Resolution Mass Spectrometry

High resolution mass spectrometric data were obtained by the mass spectrometry service of the Laboratory of Organic Chemistry at ETH Zurich on a Bruker Daltonics maXis ESI-QTOF or a Bruker Daltonics maXis II ESI-QTOF or a Thermo Q-Exactive GC Orbitrap instrument and are reported as (*m/z*).

IR Spectroscopy

Infrared spectra were recorded on a Perkin Elmer Two FT-IR spectrometer as thin films. Absorptions are reported as absorption maxima in wavenumbers (cm^{-1}).

X-Ray Crystallographic Analysis

The X-Ray diffraction was measured on a Rigaku Oxford Diffraction XtaLAB Synergy-S Dualflex kappa diffractometer equipped with a Dectris Pilatus 300 HPAD detector and using microfocus sealed tube Cu-Kα radiation with mirror optics (λ = 1.54178 Å). All measurements were carried out at 100K using an Oxford Cryosystems Cryostream 800 sample cryostat. Data collected on the Rigaku instrument were integrated using CrysAlisPro and corrected for absorption effects using a combination of empirical (ABSPACK) and numerical corrections. The structures were solved using SHELXT and refined by full-matrix least-squares analysis (SHELXL) using the program package OLEX2. All non-hydrogen atoms were refined anisotropically and hydrogen atoms were constrained to ideal geometries and refined with fixed isotropic displacement parameters (in terms of a riding model). The data

was measured and analyzed by Dr. Michael Wörle, Dr. Nils Trapp, and Michael Solar (all Small Molecule Crystallography Center, ETH Zurich).

2. Instruments for $CsPbBr_3$ quantum dots characterization

Diffusion-Ordered-Spectroscopy Nuclear Magnetic Resonance (DOSY-NMR)

DOSY spectra were recorded using a double stimulated echo pulse sequence using a 5 mm PABBO probe with a 50 G/cm gradient at a field of 11.7 T (γ_H = 500 MHz) with an avance III HD console.[1] Convection compensation is employed for the DOSY pulse sequence.

Absorption spectra (UV-Vis)

Optical characterizations were performed at ambient conditions. UV-Vis absorption spectra of colloidal QDs were collected using a Jasco V670 spectrometer in transmission mode.

Photoluminescence (PL)

A Fluorolog iHR 320 Horiba Jobin Yvon spectrofluorometer equipped with a PMT detector was used to acquire steady-state PL spectra. QD solutions were measured in the same dilutions and solvents as the absorption measurements.

Scanning transmission electron microscopy (STEM)

The STEM investigations were performed on an aberration-corrected, dedicated STEM microscope, a HD-2700CS (Hitachi). The microscope was operated at an acceleration potential of 200 kV (cold field emitter). A probe corrector (CEOS) that is incorporated in the microscope column between the condenser lens and the probe forming objective lens provides a resolution below 0.1 nm. By high-angle annular dark field (HAADF) STEM, the image is generated with incoherently scattered electrons resulting in an intensity strongly increasing with the atomic number (Z-contrast). Furthermore, a secondary electron detector is installed inside the column of the HD-2700CS microscope allowing one to study the sample morphology as well. The images (1024 × 1024 pixels) were recorded with frame times between 10 and 20 s. The data was measured by Dr. F. Krumeich (Scientific Center for Optical and Electron Microscopy (ScopeM) at ETH Zurich).

Transmission electron microscopy (TEM)

Transmission electron microscopy images were collected using a Hitachi HT7700 microscope operated at 100 kV. Images were processed using ImageJ.

3. Photoreactor set-up

All photoreactions were carried out in a custom-designed photoreactor.[2] It features 10 circularly arranged blue LEDs, mounted on copper heat sinks, which surround the central reaction vessel holder (see left picture). The reactor is air cooled with four fans (see picture right) and water cooled. Fresh cold water is continuously provided to the reactor. The blue LEDs (manufacturing number: SBR-70-B-R75-KG300; manufacturer: Luminus) were bought from Mouser Electronics.

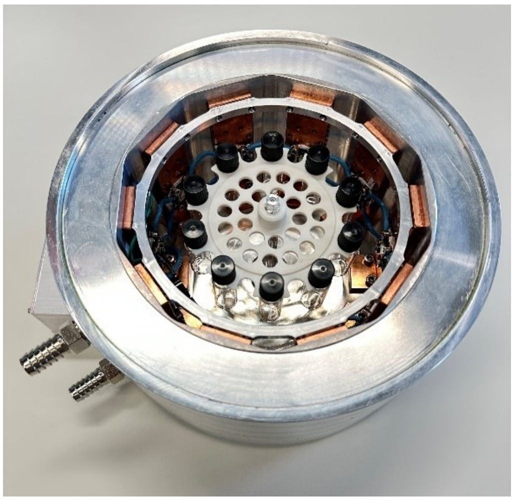

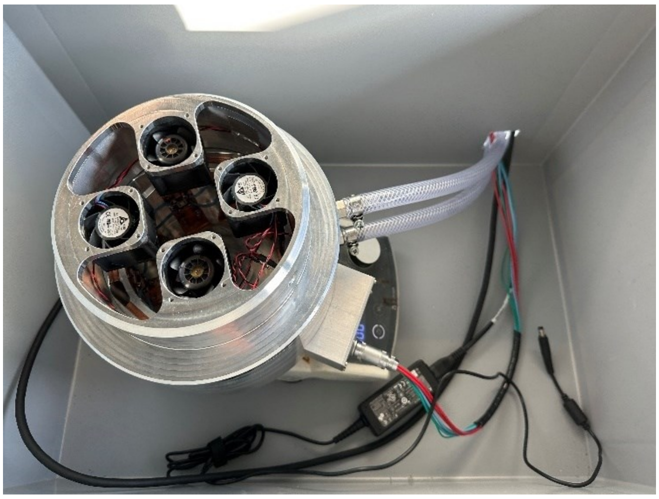

The emission spectrum (see below) of the blue LED reactor shows a maximum intensity at a wavelength of $\lambda_{max,\ emission}$ = 446 nm.[3]

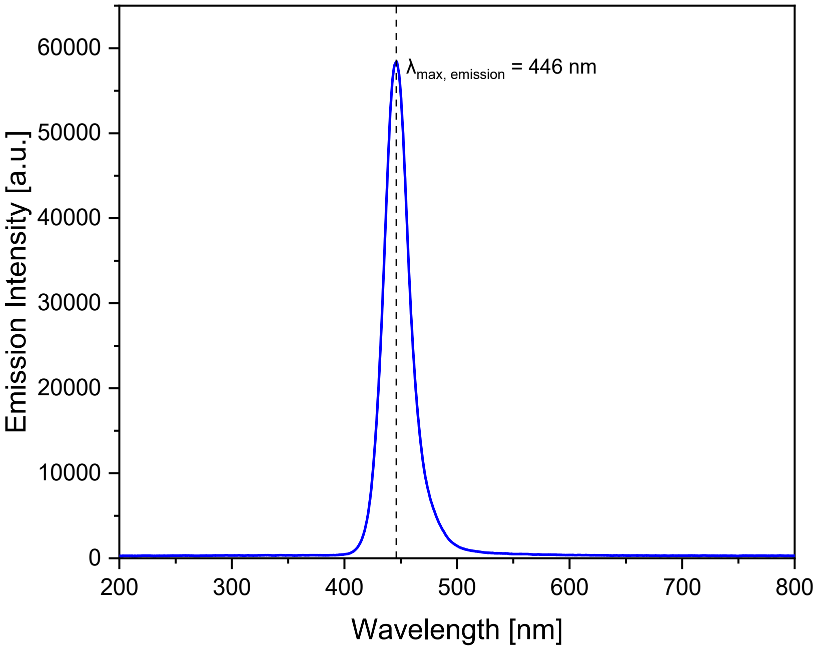

4. Quantum dots investigated in this study

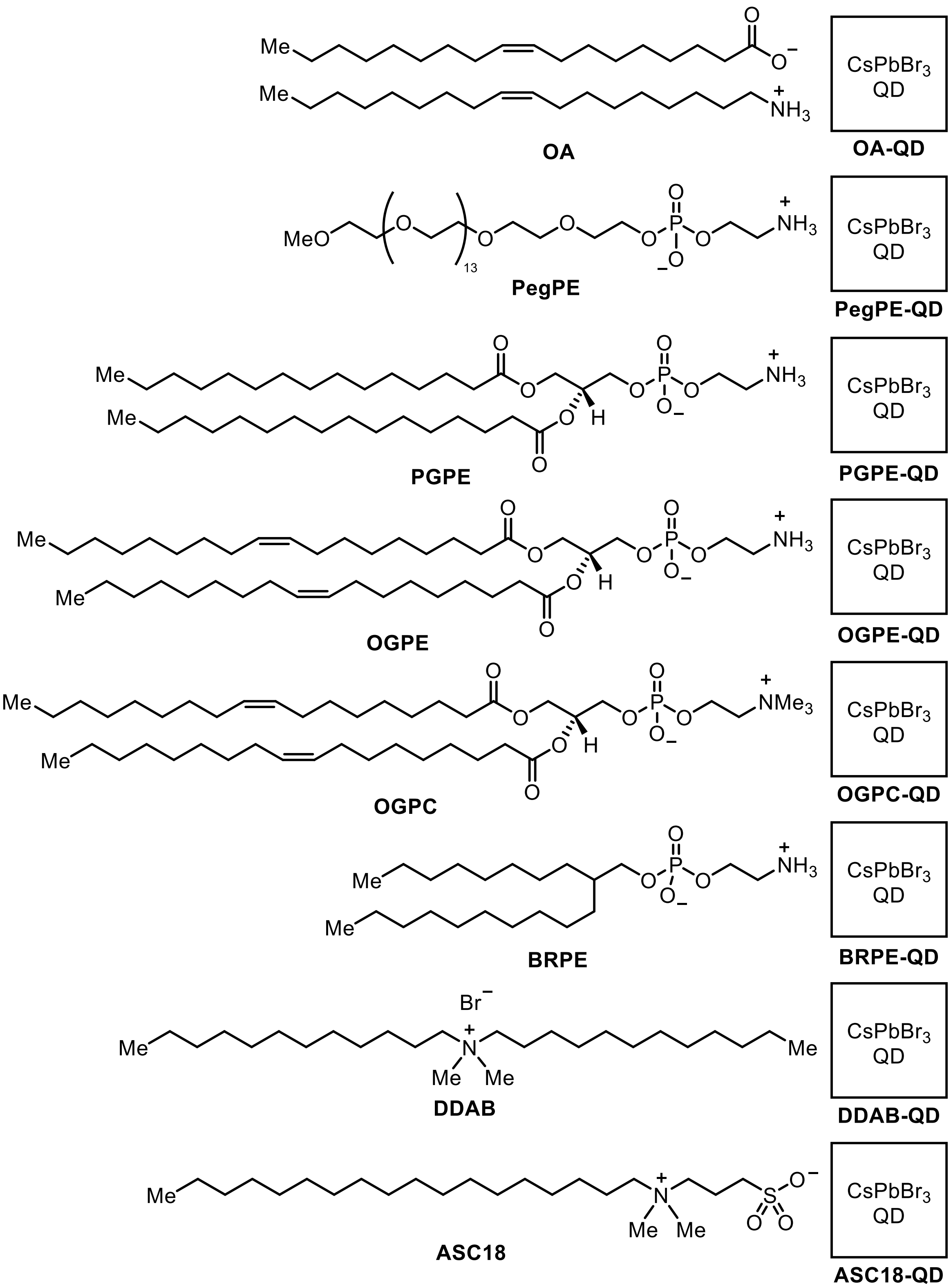

5. Synthesis of ligand capped $CsPbBr_3$ quantum dots

Chemicals: Cesium carbonate (Cs_2CO_3, 99.9%), 1-octadecene (ODE, 90%) and 3-(*N*,*N*-dimethyloctadecylammonio) propanesulfonate (ASC18, $\geq$99.0%) were purchased from Sigma-Aldrich; lead(II) acetate trihydrate (>99%, for analysis) and bromine (Br_2, >99%) from Acros Organics; trioctylphosphine (TOP), trioctylphosphine oxide (TOPO, 90%), and oleylamine (95%) from Strem; toluene (99.85%, extra dry over molecular sieve, AcroSeal®) and oleic acid (90%) from Thermo Scientific; bis(2,4,4-trimethylpentyl)phosphinic acid (DOPA) from Fluorochem; and ethyl acetate (EtOAc, > 99.7%, HPLC grade) from Fisher Scientific.

If the quantum dot synthesis required heating, internal temperature control was generally achieved with a Teflon coated thermocouple

Precursor syntheses

Cesium oleate: A three-necked flask equipped with a stir bar was charged with Cs_2CO_3 (1.63 g, 5.00 mmol, 1.00 equiv), oleic acid (5.05 mL, 4.52 g, 16.0 mmol, 3.30 equiv), and ODE (20 mL). The reaction flask was kept at room temperature and set under vacuum. The reaction mixture was stirred until the gas evolution stopped. The flask was subsequently heated to 120 °C and stirred for an additional 1 h under vacuum. This yields a 0.4 M solution of Cs-oleate in ODE. Once the reaction cooled to room temperature, the solution becomes a solid which was stored under nitrogen. Prior to the quantum dot synthesis, the solid was melted by gently heating it with a heat gun.

Lead-oleate: A three-necked flask equipped with a stir bar was charged with Lead (II) acetate trihydrate (4.55 g, 12.0 mmol, 1.00 equiv), oleic acid (7.57 mL, 6.78 g, 24.0 mmol, 2.00 equiv), and ODE (16.4 mL). The reaction flask was kept at room temperature and set under vacuum. The reaction mixture was stirred until the gas evolution stopped. The flask was subsequently heated to 120 °C and stirred for an additional 1 h under vacuum. This yields a 0.5 M solution of Pb-oleate in ODE. Once the reaction was cooled to room temperature, the solution becomes a solid which was

stored under nitrogen. Prior to the quantum dot synthesis, the solid was melted by gently heating it with a heat gun.

TOP-Br_2: A Schlenk flask equipped with a stir bar was charged with TOP (6.00 mL, 4.99 g, 13.5 mmol, 1.00 equiv.) and 18.7 mL anhydrous toluene. The solution was stirred vigorously and Br_2 (0.600 mL, 1.87 g, 11.7 mmol, 0.87 equiv.) was added dropwise over two minutes. After completed addition, the resulting solution was stirred for 1 h under a nitrogen atmosphere. This led to the formation of a pale-yellow viscous solution (app. 0.46 M) which was stored under nitrogen. Prior to the quantum dot synthesis, the viscous solution was liquified by gently heating it with a heat gun.

$CsPbBr_3$ quantum dots synthesis (hot injection method):

ASC18-QDs: 3-(*N*,*N*-dimethyloctadecylammonio) propanesulfonate (ASC18) (63.0 mg, 0.150 mmol, 0.313 equiv.), 1.20 mL Cs-oleate (0.4 M solution, 0.480 mmol, 1.00 equiv), 1.50 mL Pb-oleate (0.5 M solution, 0.750 mmol, 1.56 equiv.), and 5.00 mL ODE were added to a 100 mL three-necked round-bottom flask equipped with a stir bar. After evacuating and backfilling the reaction flask with nitrogen three times, the reaction mixture was heated to 130°C under a nitrogen atmosphere. Under vigorous stirring, 1.50 mL TOP-Br_2 (0.46 M solution, 0.690 mmol, 1.44 equiv.) were added at the same temperature. The resulting solution was rapidly cooled to room temperature with an ice-water bath and subsequently centrifuged at 12.1 krpm for 10 minutes. The supernatant was transferred into a fresh centrifuge tube and 20 mL EtOAc were added, followed by centrifugation at 12.1 krpm for 10 minutes. The precipitate was redispersed in 3 mL PhMe followed by 6 mL EtOAc and centrifugation at 12.1 krpm for one minute. The latter procedure (redispersion in toluene, precipitation with EtOAc and centrifugation) was repeated two more times. After the third centrifugation cycle, the precipitate was redispersed in 3 mL toluene. The resulting solution was centrifuged at 12.1 krpm for 3 minutes one last time to remove any larger aggregates. The supernatant was stored and used as catalyst stock solution in the reactions.

DDAB-QDs: A 100 mL flask equipped with a stir bar was charged with Cs_2CO_3 (814 mg, 2.50 mmol, 1.00 equiv), oleic acid (2.50 mL, 2.24 g, 7.92 mmol, 3.17 equiv), and distilled ODE (40 mL). The resulting reaction mixture was degassed under vacuum at room temperature. Once the gas evolution ceased, the reaction mixture was heated to 100°C and stirred for 40 min while maintaining the vacuum. After this time, the reaction was allowed to cool to room temperature. The resulting solution (0.118M) was stored in a nitrogen filled glove box.

A 500 mL three-necked round-bottom flask equipped with a stir bar, was charged with $PbBr_2$ (1.10 g, 3.00 mmol, 1.00 equiv) and distilled ODE (100 mL). The flask was placed into a heating mantle and the resulting mixture was stirred for 30 min at 100°C under vacuum and subsequently backfilled with nitrogen. The solution was then heated rapidly to 180 °C. The rapid heating was achieved by increasing the temperature of the heating mantel to the maximum which allowed for reaching 180 °C after approx. 30 minutes. When the temperature reached 120 °C, dried oleic acid (10.0 mL, 8.95 g, 31.7 mmol, 10.6 equiv) and distilled oleyl amine (10 mL, 8.13 g, 30.4 mmol, 10.1 equiv) were injected. At 180°C, the flask is set under vacuum and 16 mL Cs-oleate (0.118 M stock solution, 1.89 mmol, 0.630 equiv) are injected from a 50-mL dropping funnel. After 15 seconds, the resulting solution was rapidly cooled to room temperature using an ice-water bath. The unpurified solution was spread over three centrifuge tubes and subjected to centrifugation at 12.1 krpm for five minutes. The resulting supernatant was discarded, and the precipitate was redispersed in anhydrous hexane (2 mL for each tube, total volume: 6 mL,). The solutions were centrifuged a second time for 2min at 10 krpm and the precipitate was discarded. The supernatants were combined and diluted with additional anhydrous 6 mL hexane (total volume now: 12 mL).

Note: The oleic acid was dried by heating it to 100 °C under vacuum for 2 hours. After this time, the dried oleic acid was transferred into a nitrogen filled glove box.

<u>For the treatment with DDAB/$PbBr_2$:</u> A 10 mL flask equipped with a stir bar was charged with, DDAB (92.5 mg, 0.200 mmol), $PbBr_2$ (36.7 mg, 0.100 mmol), and 3 mL toluene. The reaction mixture was heated to 100°C and stirred until it became clear. After this time, the solution was allowed to cool to room temperature and used immediately.

The previously prepared QDs in 12 mL hexane were transferred into a flask equipped with a stir bar. 12 mL anhydrous toluene, and 3 mL of the DDAB/$PbBr_2$/toluene solution were subsequently added. The resulting reaction mixture was stirred for 1 h at room temperature. After this time, the mixture was washed by adding 36 mL EtOAc, followed by centrifugation at 12.1 krpm for 5 min. The supernatant was discarded, and the precipitate was redispersed in 20 mL anhydrous toluene. The resulting stock solution contained the DDAB treated QDs which were employed in our reaction. The solution was stored in a nitrogen filled glovebox.

OA-QDs: A 100 mL three-necked round-bottom flask equipped with a stir bar and a Teflon coated thermocouple to monitor the internal temperature of the flask, was charged with 138 mg $PbBr_2$ (0.376 mmol, 1.00 equiv) and 10 mL ODE. The flask was put under vacuum and heated to 80°C. Once the gas evolution ceased, the temperature was increased to 120°C and stirred for 10 minutes. After this time, the flask was backfilled with nitrogen and heated to 180 °C. Once the temperature was reached, 1.00 mL oleic acid (895 mg, 3.17 mmol, 8.43 equiv) and 1.00 mL oleylamine (813 mg, 3.04 mmol, 8.08 equiv) were injected followed by fast injection of 0.800 mL Cs-oleate (0.118M solution in ODE, 0.0944 mmol, 0.250 equiv). Immediately upon addition, the flask was rapidly cooled to room temperature using an ice-water bath. The reaction mixture was subsequently centrifuged at 12.1 krpm for 10 minutes. The resulting precipitate was redispersed in 1.00 mL toluene and centrifuged again at 12.1 krpm for 3 minutes to remove larger aggregates. The supernatant was the stock solution containing OAQD, employed in our reaction. The solution was stored in a nitrogen filled glove box.

$CsPbBr_3$ Quantum Dots Synthesis (TOPO-DOPA Method):

Precursor Syntheses

$PbBr_2$-TOPO (0.04 M): The stock solution was prepared without the exclusion of air. A 40 mL glass vial equipped with a stir bar was charged with $PbBr_2$ (367 mg, 1.00 mmol, 1.00 equiv), TOPO (1.93 g, 5.00 mmol, 5.00 equiv) and 5.00 mL *n*-octane. The reaction mixture was heated to 120 °C and stirred at the same temperature until the $PbBr_2$ was dissolved (approx. 45 min). After this time, the vial was allowed to cool

to room temperature and 20 mL of anhydrous hexane were added. The solution was filtered with a 0.45 µm PTFE syringe filter and stored under ambient conditions.

Cs-DOPA (0.02 M): The stock solution was prepared without the exclusion of air. A 40 mL glass vial equipped with a stir bar was charged with Cs_2CO_3, (100 mg, 0.307 mmol, 1.00 equiv), DOPA (1.00 mL, 916 mg, 3.15 mmol, 10.3 equiv), and 2.00 mL *n*-octane. The reaction mixture was heated to 120 °C and stirred at the same temperature until the Cs_2CO_3 was dissolved (approx. 30 min). After this time, the vial was allowed to cool to room temperature and 27 mL of anhydrous hexane were added. The solution was filtered with a 0.45 µm PTFE syringe filter and stored under ambient conditions.

Ligand Solutions: Each ligand was dissolved in a specific solvent prior to the addition in the TOPO-DOPA synthesis. Concentrations were determined to add the same amount of PEA/PC head group to the reaction mixture.

Table 1: Ligand solutions

Ligand	Solvent	Concentration [mg/µL]
**BRPE**	mesitylene	0.100
**PegPE**	$CHCl_3$	0.100
**PGPE**	9:1 $CHCl_3$:MeOH	0.160
**OGPE**	PhMe	0.176
**OGPC**	PhMe	0.186

$CsPbBr_3$ quantum dots synthesis

The procedure was adopted with slight modifications from the literature.[4] A 20 mL glass vial equipped with a stir bar was charged with 4.00 mL hexane (except when **PegPE** was used as ligand, where no hexane was added), 4.00 mL $PbBr_2$-TOPO (0.04 M stock solution, 0.160 mmol) and 2.00 mL Cs-DOPA (0.02 M stock solution, 0.040 mmol) while stirring vigorously at room temperature. The particles were grown for 10 minutes, after which the ligand solution (0.200 mL) was injected. The resulting mixture was stirred for an additional minute at room temperature to ensure surface passivation. After this time, the QDs were precipitated by addition of the corresponding

anti-solvent (see Table 2) and the mixture was centrifuged for one minute at 12.1 krpm. The precipitate was redispersed in the corresponding washing solvent (see Table 2) before adding the anti-solvent again and centrifuging the mixture at 12.1 krpm for 1 minute. This procedure was repeated two more times. The precipitate was redispersed in the final solvent for dispersion (see Table 2) and centrifuged at 12.1 krpm for 3 minutes to remove larger aggregates. The supernatant containing the QDs was stored in a nitrogen filled glovebox as catalyst stock solution for our reaction.

Table 2: Treatment of LHP QDs after synthesis

Ligand	Washing Solvent	Washing Anti-Solvent	Final Solvent for Dispersion
**BRPE**	Hexane (1.00 mL)	Acetone (1.00 mL)	Hexane
**PegPE**	$CHCl_3$ (1.00 mL)	Hexane (3.00 mL)	PhCl
**PGPE**	CyH (1.00 mL)	2:1 EtOAc:MeCN (2.00 mL)	CyH
**OGPE**	PhMe (1.00 mL)	Acetone (1.00 mL)	Hexane
**OGPC**	PhMe (1.00 mL)	Acetone (1.00 mL)	Hexane

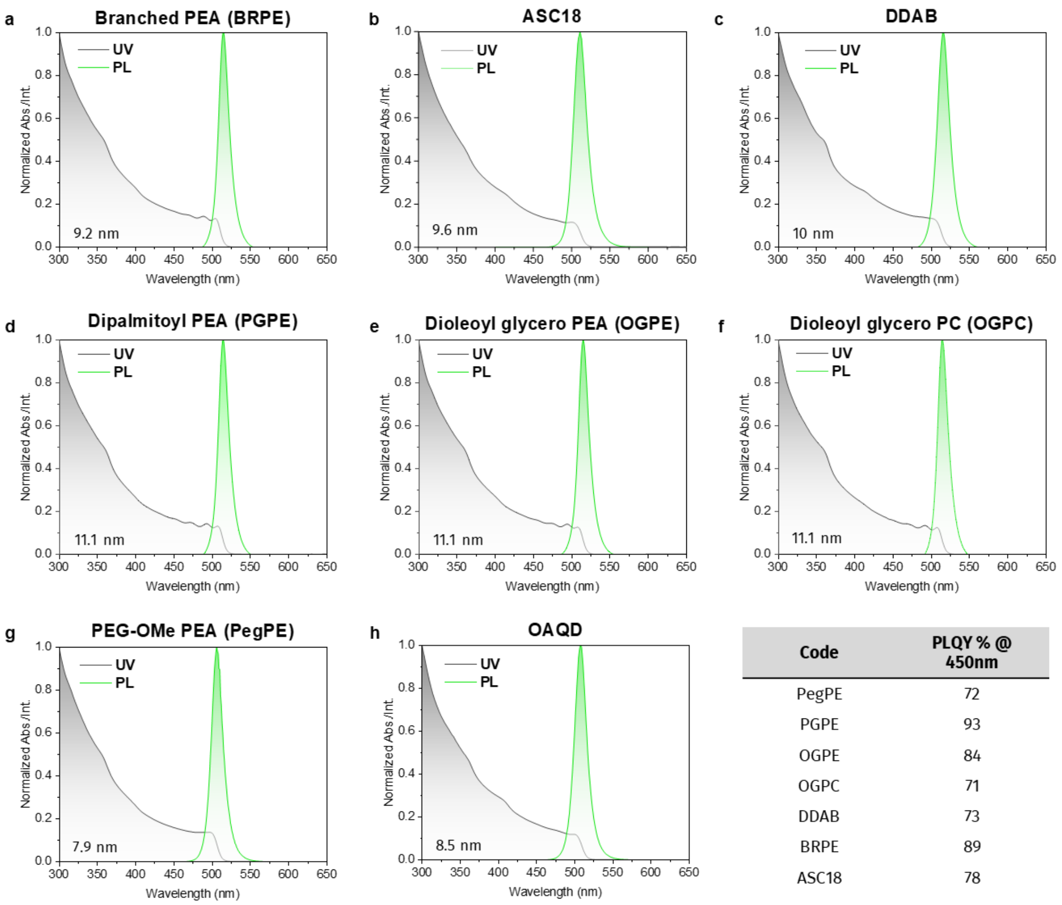

Code	PLQY % @ 450nm
PegPE	72
PGPE	93
OGPE	84
OGPC	71
DDAB	73
BRPE	89
ASC18	78

Figure 1: Characterization data for synthesized LHP QDs.

Table 3: Size, Concentration, and Catalyst Loading of Synthesized LHP QDs

Entry	QDs	QDs size	QD stock solution in	Conc. of QD stock solution	Catalyst loading [mol %]
1	**OA-QDs**	8.5 nm	PhMe	21.60 mg/mL	$14.2 \cdot 10^{-6}$
2	**PegPE-QDs**	7.9 nm	PhCl	11.04 mg/mL	$17.7 \cdot 10^{-6}$
3	**PGPE-QDs**	11.1 nm	CyH	11.41 mg/mL	$6.39 \cdot 10^{-6}$
4	**OGPE-QDs**	11.1 nm	Hexane	22.63 mg/mL	$6.39 \cdot 10^{-6}$
5	**OGPC-QDs**	11.1 nm	Hexane	19.46 mg/mL	$6.39 \cdot 10^{-6}$
6	**BRPE-QDs**	9.2 nm	Hexane	23.69 mg/mL	$11.2 \cdot 10^{-6}$
7	**DDAB-QDs**	10.0 nm	PhMe	10.73 mg/mL	$8.74 \cdot 10^{-6}$
8	**ASC18-QDs**	9.6 nm	PhMe	44.77 mg/mL	$9.88 \cdot 10^{-6}$

6. Calculation of catalyst loading

The following calculations allow us to determine the catalyst loading of the employed LHP QDs. This is subsequently shown with **PegPE-QDs** as example. It is known from transmission electron spectroscopy that $CsPbBr_3$ QDs are cuboidal.[5] Consequently, as the edge length of **PegPE-QD** is d = 7.9 nm (Figure 1 and Table 3), the volume V_{QD} of **PegPE-QD** is calculated according to equation **E1**:

$$V_{QD}(\mathbf{PegPEQD}) = d^3 \tag{E1}$$

with d = edge length of **PegPE-QD** in nanometer (nm). Next, we had to determine the number of $CsPbBr_3$ unit cells N_{UC} which fit into one **PegPE-QD**. According to Møller's work and later reports, the dimension a of one cubic $CsPbBr_3$ unit cell amounts to a = 0.5874 nm.[6] Consequently, the volume of the $CsPbBr_3$ unit cell V_{UC} equals:

$$V_{UC}(CsPbBr_3) = a^3 = 0.2027\, nm^3 \tag{E2}$$

The number N_{UC} of $CsPbBr_3$ unit cells fitting into one **PegPE-QD** is calculated according to equation **E3:**

$$N_{UC} = \frac{V_{QD}(\mathbf{PegPEQD})}{V_{UC}(CsPbBr_3)} = 2459 \tag{E3}$$

We decided to add 0.150 mg = 0.259 µmol $CsPbBr_3$ (calculated based on the molecular weight of $CsPbBr_3$: M = 579.82 g/mol) to 1.00 mL solvent for our reaction. Thus, the concentration c of $CsPbBr_3$ in our reaction is:

$$c(CsPbBr_3) = \frac{n(CsPbBr_3)}{V_{Reaction}} = \frac{0.000259\, mmol}{1.00\, ml} = 2.59 \cdot 10^{-4} M \tag{E4}$$

The concentration of quantum dots in solution is obtained by dividing c($CsPbBr_3$) by the number N_{UC} of $CsPbBr_3$ unit cells in one **PegPE-QD** which we already determined in equation **E3** to be N_{UC} = 2459.

$$\mathrm{c}(\mathbf{PegPEQD}) = \frac{\mathrm{c}(CsPbBr_3)}{N_{UC}} = 1.05 \cdot 10^{-7} M \tag{E5}$$

Lastly, the amount n(**PegPE-QD**) in our reaction is determined by multiplying c(**PegPE-QD**) with the reaction volume $V_{Reaction}$ = 0.001 L:

$$\mathrm{n}(\mathbf{PegPEQD}) = \mathrm{c}(\mathbf{PegPEQD}) \cdot \mathrm{V}_{Reaction} = 1.05 \cdot 10^{-10} mol \qquad \textbf{(E6)}$$

We decided to run the bromination reaction on $n_{Reaction}$ = 0.6 mmol scale. With n(**PegPE-QD**) = 1.05•10^{-10} mol, we calculated the catalyst loading of **PegPE-QD** according to equation **E7**:

$$mol\%(\mathbf{PegPEQD}) = \frac{\mathrm{n}(\mathbf{PegPEQD})}{\mathrm{n}_{Reaction}} \cdot 100 = 1.77 \cdot 10^{-5} mol\ \% \qquad \textbf{(E7)}$$

7. Performance of different ligand capped $CsPbBr_3$ QDs as photocatalysts

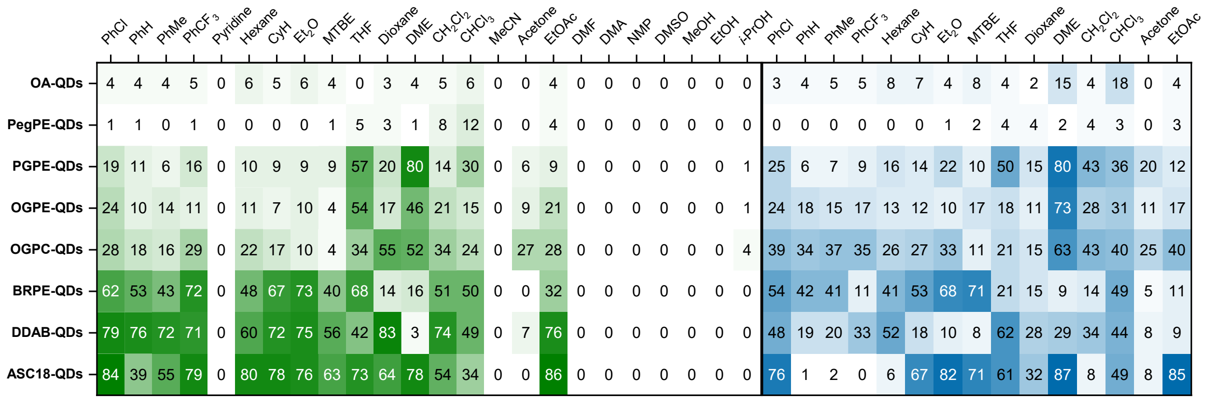

Figure 2: The green heat map displays ^1H NMR yields with mesitylene as an internal standard for the benzylic bromination of PhEt (**1a**). The blue heat map displays ^1H NMR yields with mesitylene as an internal standard for the benzylic bromination of PhEt (**1a**) in the presence of 1000 ppm H_2O in 15 different solvents as part of a moisture study.

The data presented in Figure 2 (Figure 2b in the main manuscript) was obtained as follows:

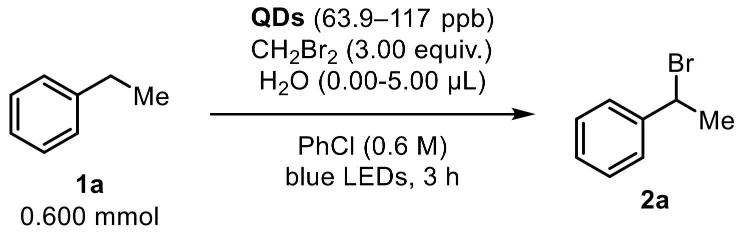

Anhydrous reaction conditions – Procedure P1 (green heat map)

All reactions were conducted in a nitrogen filled glove box. The 24 anhydrous solvents were purchased from Acros or Sigma Aldrich and used as received. A glass vial was sequentially charged with solvent (1.00 mL), CH_2Br_2 (125.4 µL, 1.800 mmol, 3.00 equiv), PhEt (73.5 µL, 63.7 mg, 0.600 mmol, 1.00 equiv), and **QDs** (0.15 mg, see Table 3 for concentrations of the stock solutions and resulting catalyst loading). The vial was equipped with a magnetic stirrer bar and capped with a screwcap before irradiating the reaction in a 350 W photoreactor (*vide supra*) for 3 h. After this time, 1,3,5-mesitylene (28.0 µL, 24.4 mg, 0.200 mmol) was added to the reaction mixture. 50 µL of the resulting solution were transferred into an NMR tube, followed by 600 µL $CDCl_3$. The NMR yield was determined by ^1H NMR spectroscopy.

Product quantification via ^1H NMR spectroscopy

To quantify the products via ^1H NMR spectroscopy, a t_1 measurement of the reaction mixture was conducted. For this purpose, a reaction was set up according to procedure P1 with **ASC18-QDs** in anhydrous C_6D_6 as solvent. 0.800 mL of the reaction mixture were subsequently transferred into an oven dried NMR tube. The latter was placed into the blue LED photoreactor and irradiated for 20 minutes. After this time, a t_1 measurement of the reaction was conducted. We determined the following relaxation times for the characteristic proton signals in **2a**:

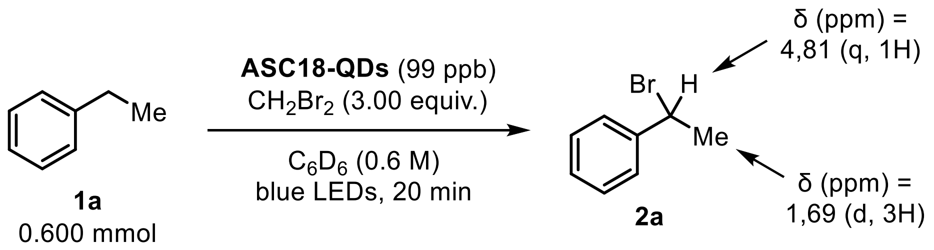

Entry	Compound	Signal in $CDCl_3$ (400 MHz)	Relaxation Time t_1
1	**2a**	δ (ppm) = 1.69 (d, 3H)	4.69 s
2	**2a**	δ (ppm) = 4.81 (q, 1H)	2.16 s

For quantitative NMR analysis, an interscan delay (d1) of five times the longest relaxation time is typically chosen.[7] In our case this would amount to a minimum of d1 = 23.5 seconds. To ensure high precision in integration throughout different solvents, an interscan delay of 30.0 seconds was chosen. Whenever possible, the ^1H NMR signal at 1.69 ppm and the signal at 4.81 ppm were both integrated. No discrepancies were found in the calculation of the ^1H NMR yield from both signals.

Wet reaction conditions – Procedure P2 (1.00 µL; 1000 ppm H_2O, blue heat map)

Our investigation of the $CsPbBr_3$ QDs catalyzed benzylic bromination under anhydrous conditions has shown that no reaction occurs in pyridine, MeCN, DMF, DMA, NMP, DMSO, MeOH, EtOH and *i*-PrOH. This can be attributed to decomposition of the LHP QDs in the respective solvents which were consequently not investigated

under wet conditions. The remaining solvents investigated in the presence of 1.00 µL H_2O were: PhCl, PhH, PhMe, $PhCF_3$, hexane, CyH, Et_2O, MTBE, THF, dioxane, DME, CH_2Cl_2, $CHCl_3$, acetone, and EtOAc.

All reactions were conducted in a nitrogen filled glove box. The 15 anhydrous solvents were purchased from Acros and used as received. A glass vial was sequentially charged with solvent (1.00 mL), CH_2Br_2 (125.4 µL, 1.800 mmol, 3.00 equiv), PhEt (73.5 µL, 63.7 mg, 0.600 mmol, 1.00 equiv) and 1.00 µL H_2O. The vial was capped and shaken vigorously for 30 seconds to ensure even distribution of the water. Then, the **QD** catalyst (0.15 mg, see Table 3 for concentrations of the stock solutions and resulting catalyst loading) was added, followed by a magnetic stirrer bar. The vial was capped with a screwcap before irradiating the reaction in a 350 W photoreactor (*vide supra*) for 3 h. After this time, 1,3,5-mesitylene (28.0 µL, 24.4 mg, 0.200 mmol) was added to the reaction mixture. 50 µL of the resulting solution were transferred into an NMR tube, followed by 600 µL $CDCl_3$. The NMR yield was determined by ^1H NMR spectroscopy.

Wet reaction conditions (2.00 – 5.00 µL; 2000 – 5000 ppm H_2O)

Amongst the eight investigated LHP QDs, **ASC18-QDs** performed best in the presence of 1.00 µL H_2O. We were subsequently interested how **ASC18-QDs** would perform with a further increasing level of moisture (2.00 – 5.00 µL). The reactions were set up according to procedure P2 (*vide supra*).

2.00 µL (2000 ppm) H_2O

Every solvent containing 1.00 µL H_2O in which **2a** was produced in >50% yield was investigated with 2.00 µL H_2O as well. These were: PhCl, CyH, Et_2O, MTBE, THF, DME, and EtOAc. The yields of **2a** are summarized in Table 4.

3.00 – 5.00 µL (3000 – 5000 ppm) H_2O

In the presence of 2.00 µL H_2O, **2a** was produced in <10% yield in PhCl and EtOAc. The remaining solvents (CyH, Et2O, MTBE, THF, DME) were investigated in the presence of 3.00, 4.00, and 5.00 µL H_2O. The yields of **2a** are summarized in Table 4.

Table 4: Yields of **2a** in the presence of 2.00-5.00 µL H_2O.

Solvent	Yield with 2.00 µL H_2O	Yield with 3.00 µL H_2O	Yield with 4.00 µL H_2O	Yield with 5.00 µL H_2O
PhCl	2%	–	–	–
CyH	59%	48%	26%	10%
Et_2O	58%	33%	9%	2%
MTBE	66%	8%	8%	9%
THF	62%	64%	10%	3%
DME	77%	71%	29%	7%
EtOAc	8%	–	–	–

8. Full comparison of TEM images

Our study on the performance of $CsPbBr_3$ QDs under anhydrous conditions and in the presence of 1.00 µL water showed that **ASC18-QDs**, **DDAB-QDs**, and **BrPE-QDs** performed best. Ideally, a photocatalyst is unchanged after the reaction. Consequently, we proceeded to investigate whether **ASC18-QDs**, **DDAB-QDs**, and **BrPE-QDs** change in size and shape over the course of the reaction. As analytical method, transmission electron microscopy (TEM) was chosen, which allowed us to evaluate shape and size of the particles.

To obtain a transmission electron microscopy image of the LHP QDs after the reaction, a fresh reaction mixture was prepared following the same procedure as described in chapter 7. However, after 3 h of blue light irradiation, no 1,3,5-mesitylene was added. Instead, the reaction vial remained closed and was transferred into a nitrogen filled glove-box to ensure that the QDs remain unaltered by air or moisture. The vial was subsequently opened and one drop of the unpurified reaction mixture was deposited onto a perforated carbon foil supported on a copper grid. After evaporation of the solvent, the grid was mounted on the single tilt holder of the microscope.

For **ASC18-QDs**, all reactions containing >50% yield were investigated. As the TEM images were studied, we noticed that depending on the solvent, the shape of **ASC18-QDs** was either cuboidal or spherical. The latter shape is usually attributed to surface etching. While we could not identify a reason for the observed surface etching, we have no evidence suggesting its impact on the photocatalytic properties of **ASC18-QDs**. Importantly, the quantum dots largely retained their size in most solvents compared to the reference (reference = **ASC18-QDs** in 1.00 mL PhCl without irradiation or reagents). Subsequently, two TEM images for each reaction mixture containing **ASC18-QDs** will briefly be discussed in the figure caption (Figure 3-Figure 15).

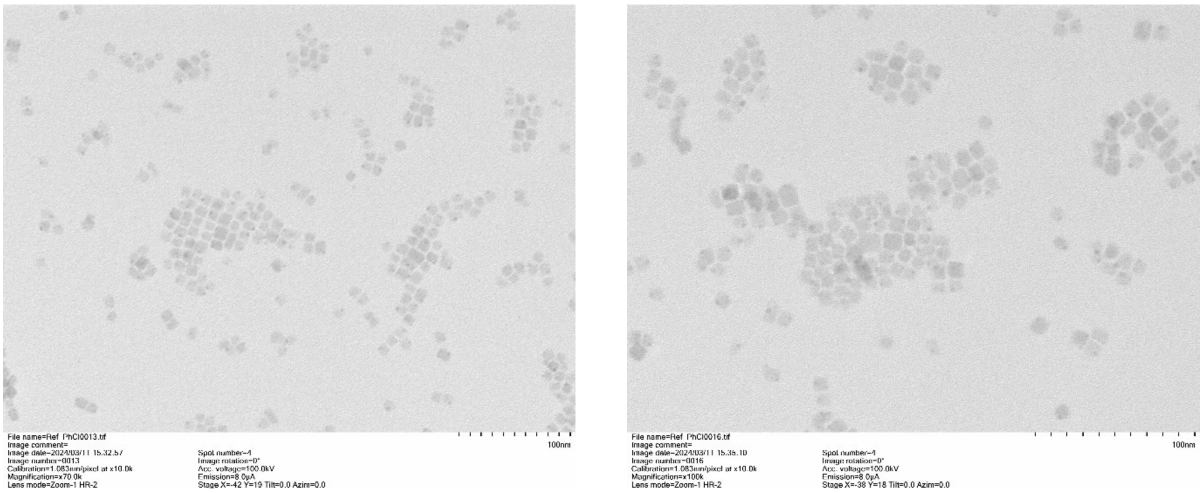

Figure 3: Sample: Reference with **ASC18-QDs** in 1.00 mL PhCl. Black spots on the quantum dots indicate the formation of elemental lead which can be attributed to the reduction of Pb^{2+} to Pb^{0} caused by the electron beam.

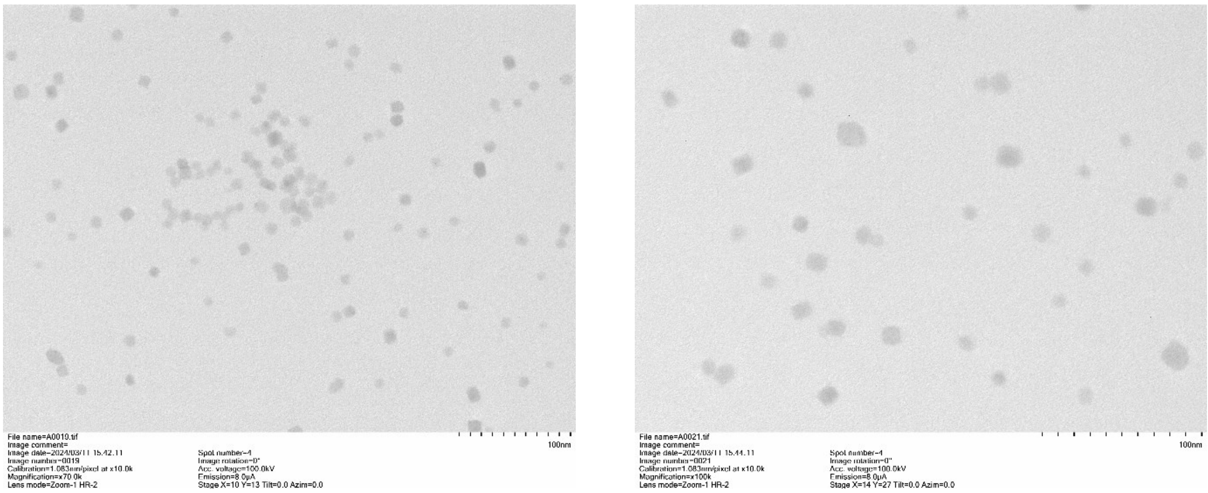

Figure 4: Sample: Reaction in PhCl. Yield: 84%. **ASC18-QDs** are more rounded compared to the reference. Most particles retained their original size. Particles with larger diameter (+ 5 nm) are encountered sporadically (e.g. 2nd TEM image right bottom corner)

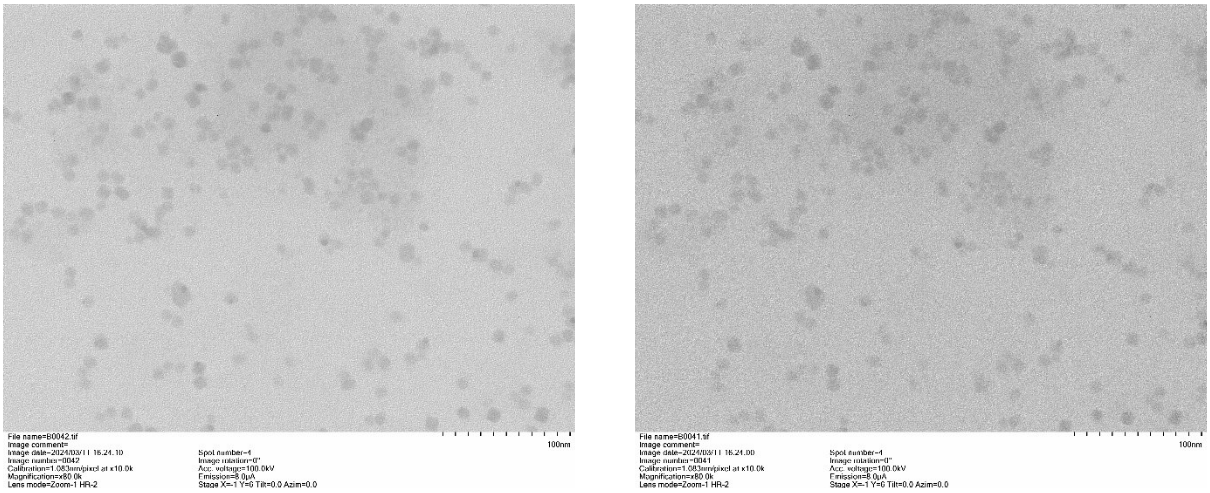

Figure 5: Sample: Reaction in PhMe. Yield: 55%. **ASC18-QDs** are etched. Most QDs retained their original size. Particles with larger diameter (+ 5 nm) are encountered sporadically.

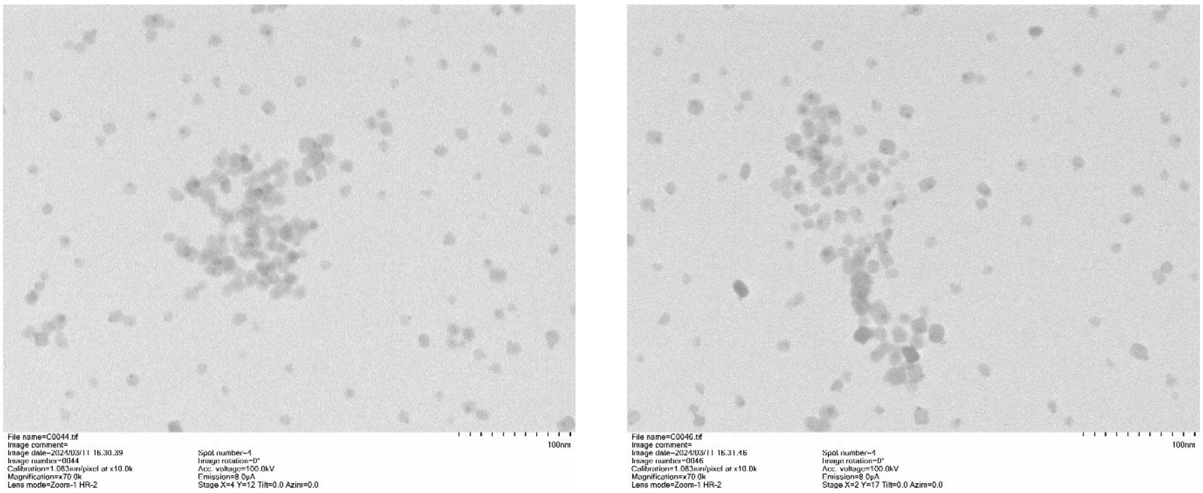

Figure 6: Sample: Reaction in $PhCF_3$. Yield: 79%. **ASC18-QDs** are etched. In a few cases, etching resulted in smaller particles compared to their original size. Some particles are spherical.

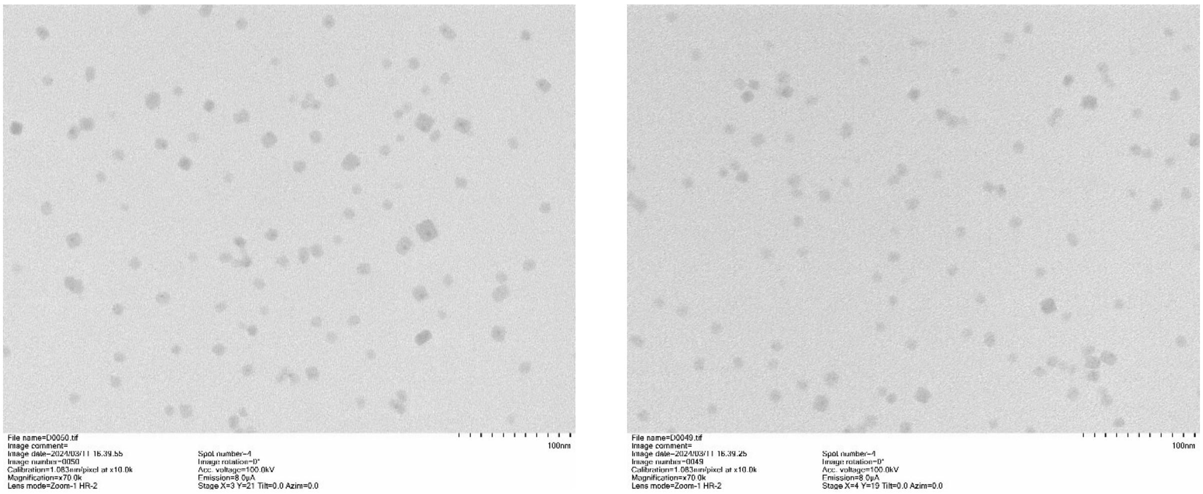

Figure 7: Sample: Reaction in hexane. Yield: 80%. **ASC18-QDs** largely maintain its original size. Etching is observed.

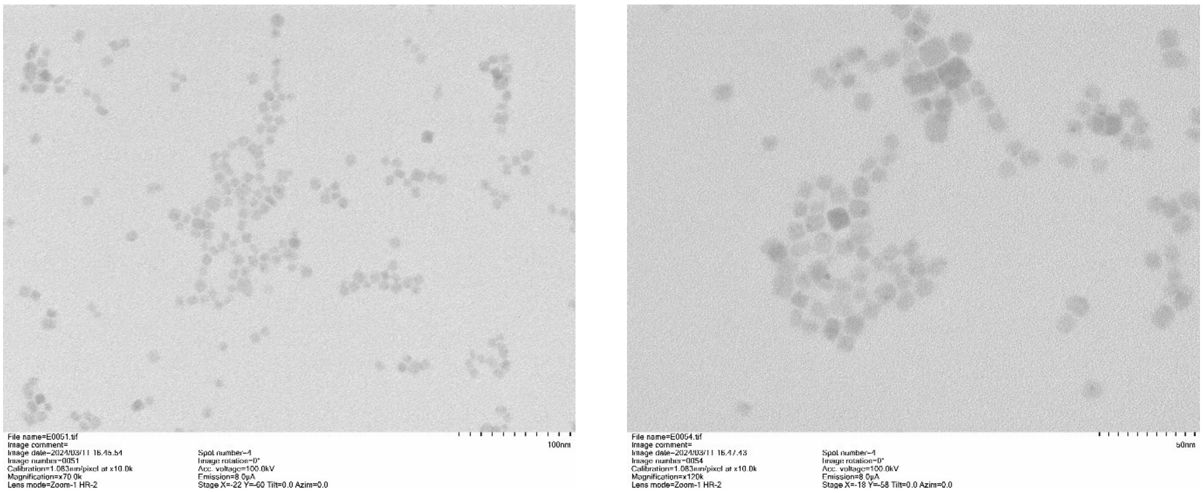

Figure 8: Sample: Reaction in CyH. Yield: 78%. **ASC18-QDs** largely maintain cubic shape and original size. Sporadically, selective etching for one lateral side is observed, resulting in a rectangular appearance of the QDs.

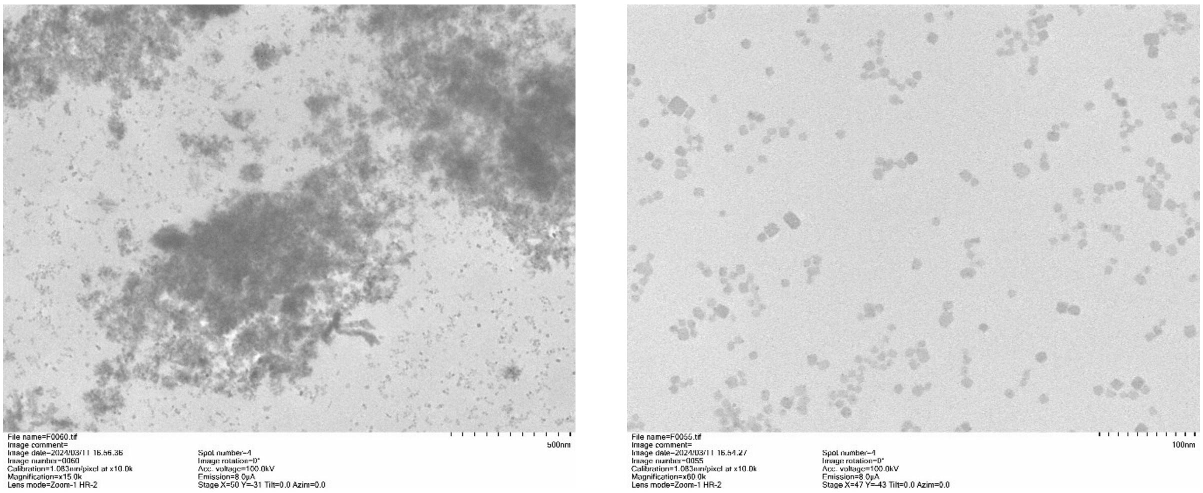

Figure 9: Sample: Reaction in Et_2O. Yield: 76%. Due to the hydrophobic chain in ligand **ASC18**, QDs can form large aggregates in polar solvents (left). Generally, etching is observed. Many particles retained the cubic shape and original size.

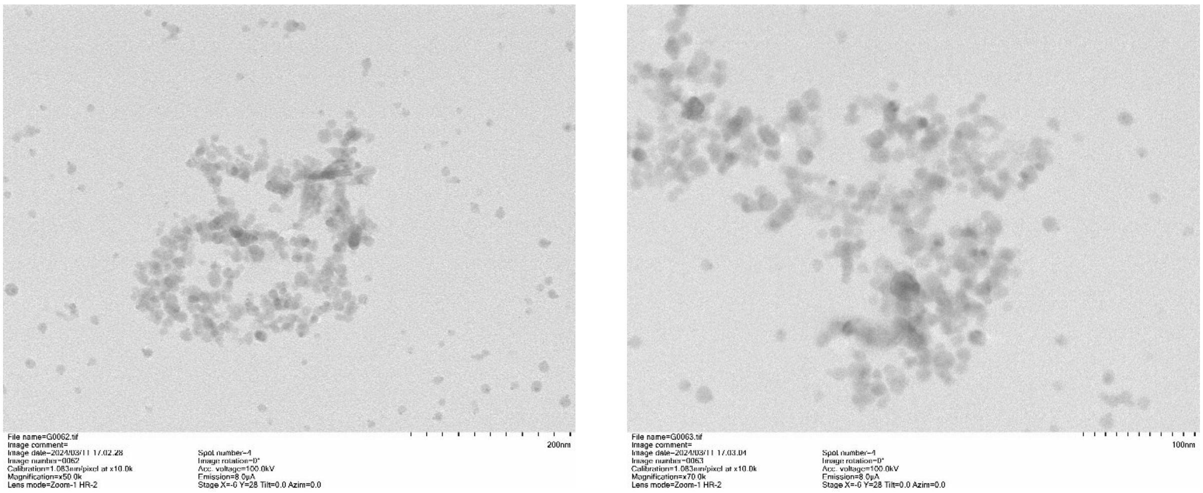

Figure 10: Sample: Reaction in MTBE. Yield: 63%. **ASC18-QDs** are etched or spherical. Strong etching results in some particles being smaller than their original size.

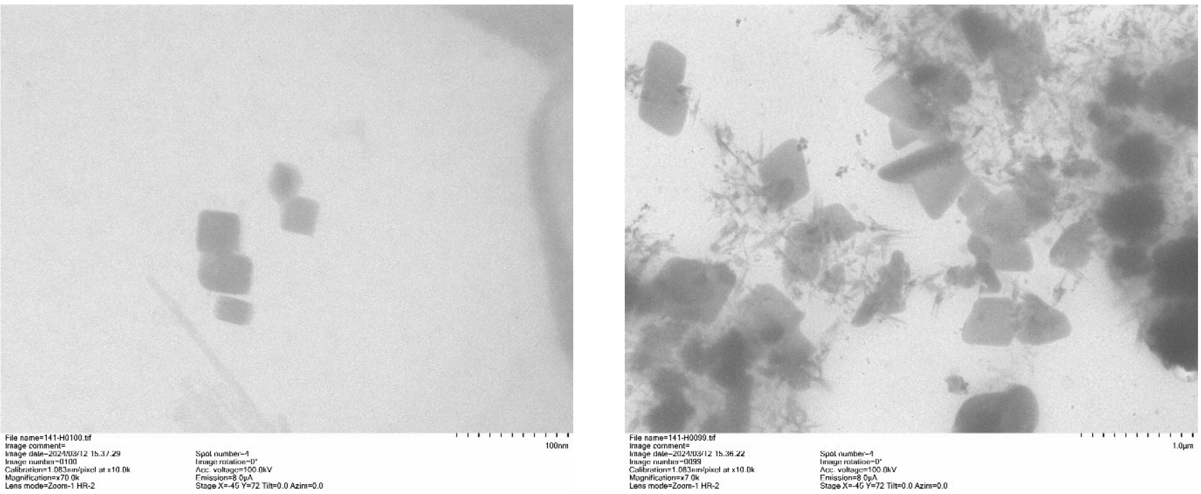

Figure 11: Sample: Reaction in THF. Yield: 73%. Some intact **ASC18-QDs** were found which retained their shape and size (left). However, higher structures were also observed (right). The latter could be organic residues or large $CsPbBr_3$ structures exceeding 100 nm in size.

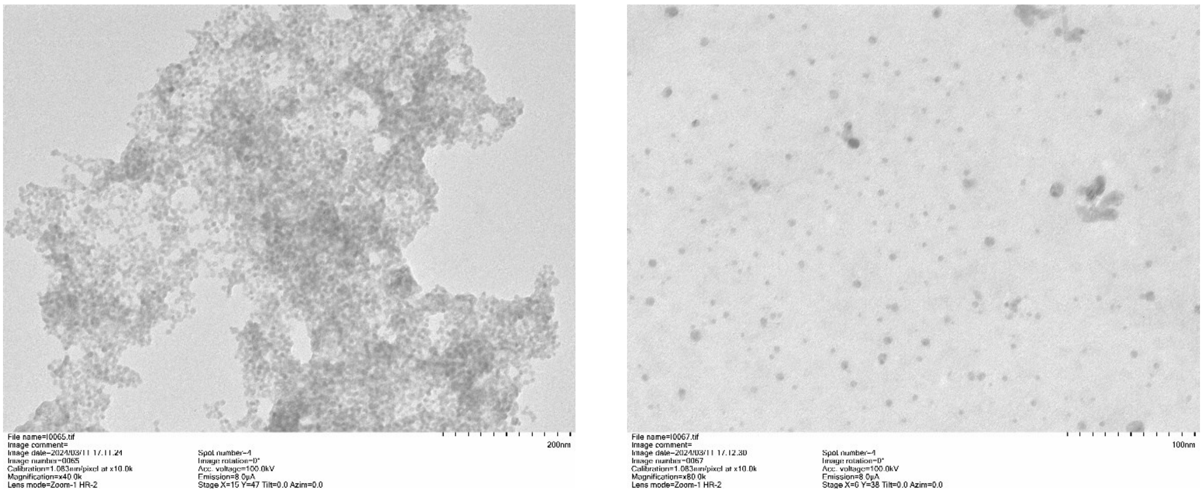

Figure 12: Sample: Reaction in dioxane. Yield: 64%. The hydrophobic chain in ligand **ASC18** results in aggregation of **ASC18-QDs**. QDs in aggregate are etched but largely retained original size (left). Particles outside of the aggregate are strongly etched, and particle size did clearly diminish (right).

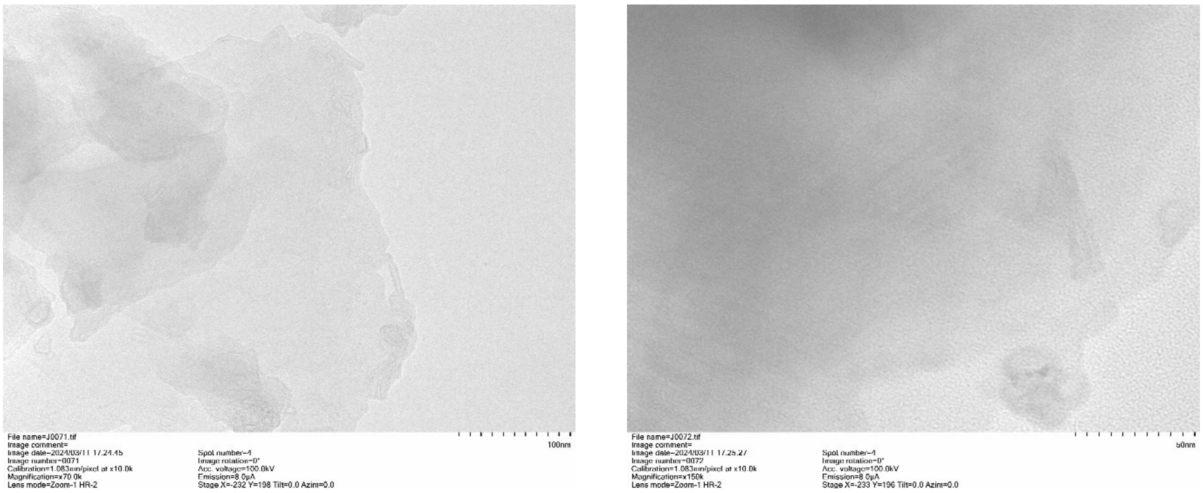

Figure 13: Sample: Reaction in DME. Yield: 78%. No **ASC18-QDs** were observed. Likely scenario: One large aggregate contains most QDs and was not found. Instead, we observed what appeared to be organic residues of some kind which could cover QDs.

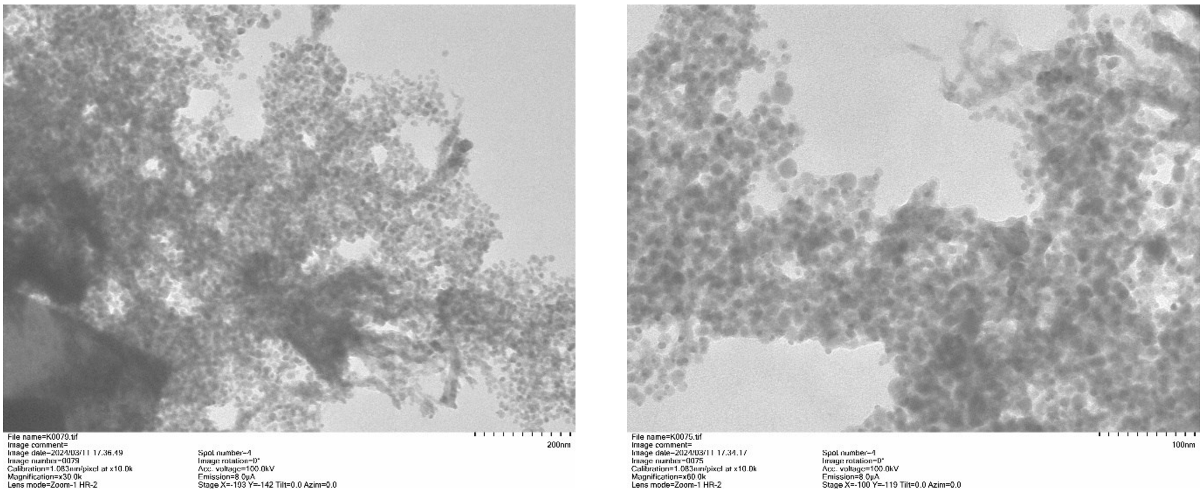

Figure 14: Sample: Reaction in CH_2Cl_2. Yield: 54%. **ASC18-QDs** form multilayered aggregates. QDs are strongly etched and spherical. Smaller and larger size QDs are observed.

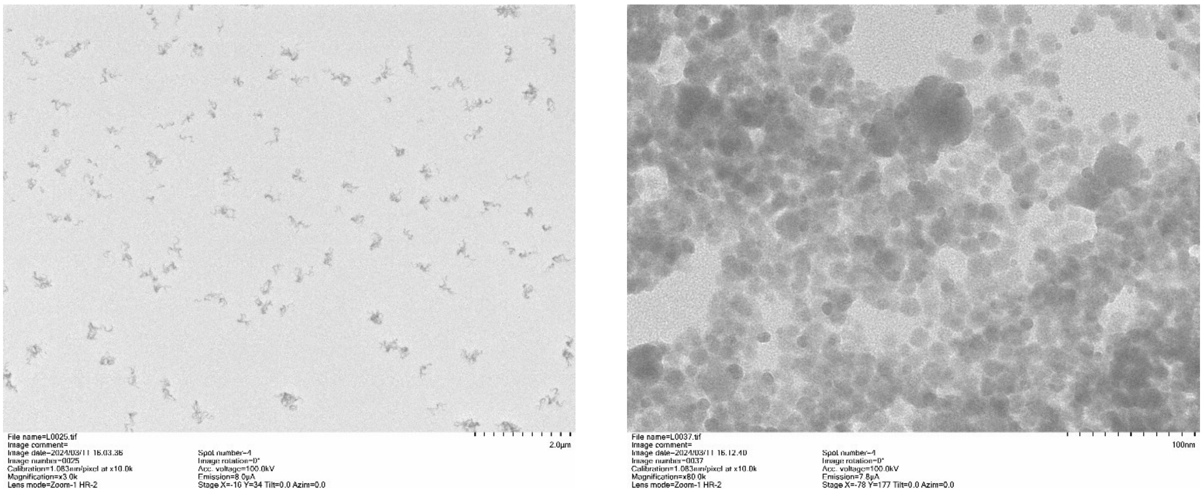

Figure 15: Sample: reaction in EtOAc. Yield: 86%. Organic residues have been observed (left). **ASC18-QDs** form aggregates in EtOAc. Particles are etched and spherical. Some QDs are larger in size compared to reference.

Next, TEM images for the reaction mixtures containing **DDAB-QDs** were measured, as it was the second best performing LHP QD. All reactions containing >40% yield were investigated. Subsequently, two TEM images for each reaction mixture will be briefly discussed in the figure caption (Figure 16-Figure 27).

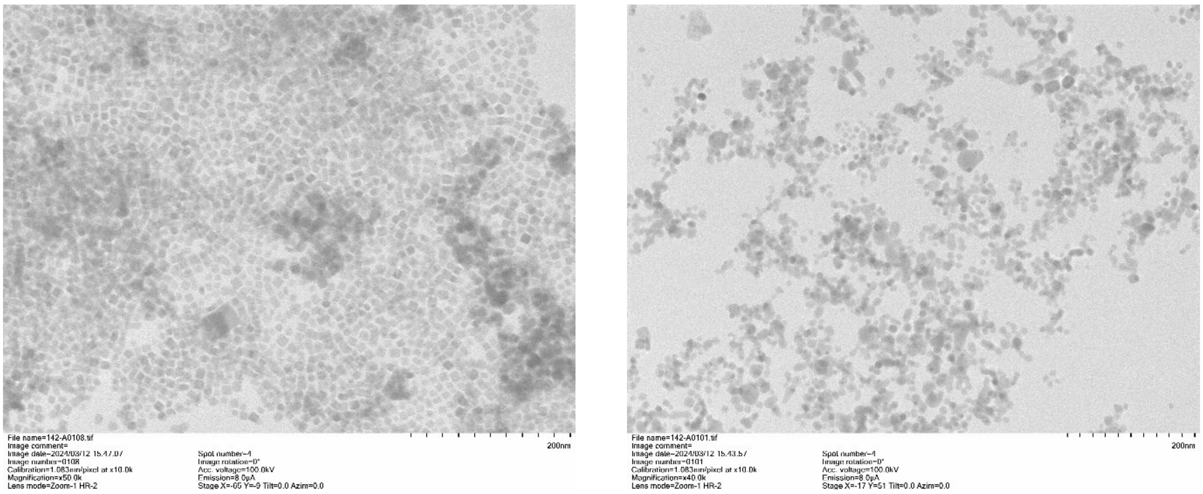

Figure 16: Sample: Reaction with **DDAB-QDs** in PhCl. Yield: 79%. Most particles retained the original size. QDs in aggregates (left) retained their cubic shape. Particles outside of aggregates are etched and have greater variation in size.

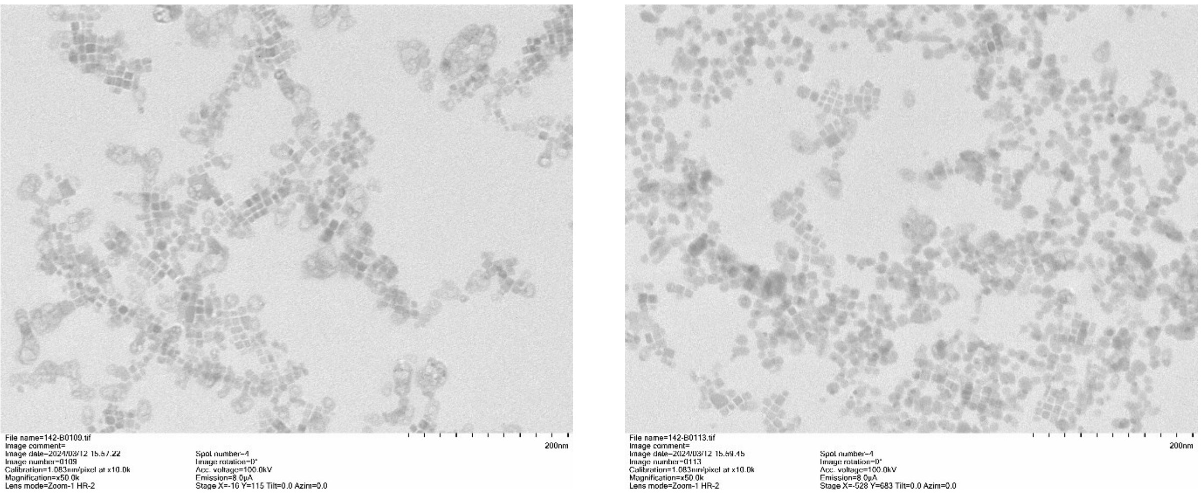

Figure 17: Sample: Reaction with **DDAB-QDs** in PhH. Yield: 76%. Most particles retained their original size, but some sintering is observed. Rounded and cubic QDs were observed.

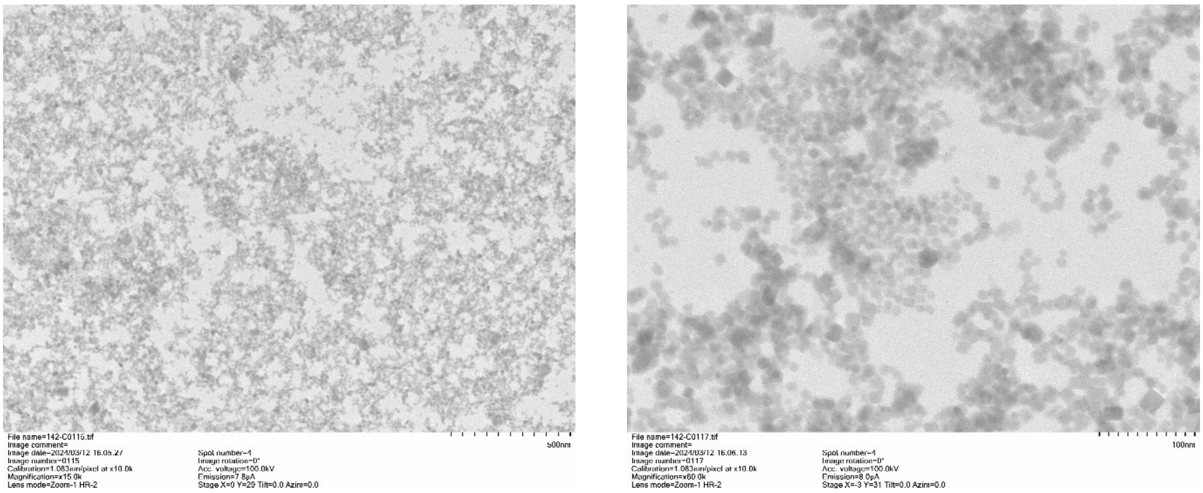

Figure 18: Sample: Reaction with **DDAB-QDs** in PhMe. Yield: 72%. Most QDs retained their original size and their cubic shape. Occasional etching and rounded QDs are observed.

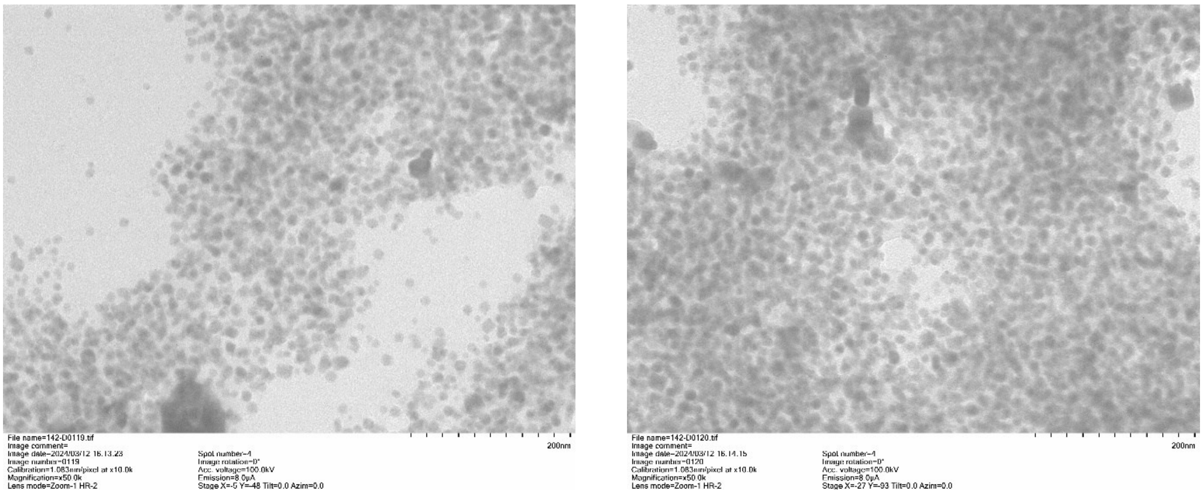

Figure 19: Sample: Reaction with **DDAB-QDs** in $PhCF_3$. Yield: 71%. Most particles are etched and appear rounded. Some QDs shrank below their original size. Average size distribution after the reaction is still in line with QDs before reaction.

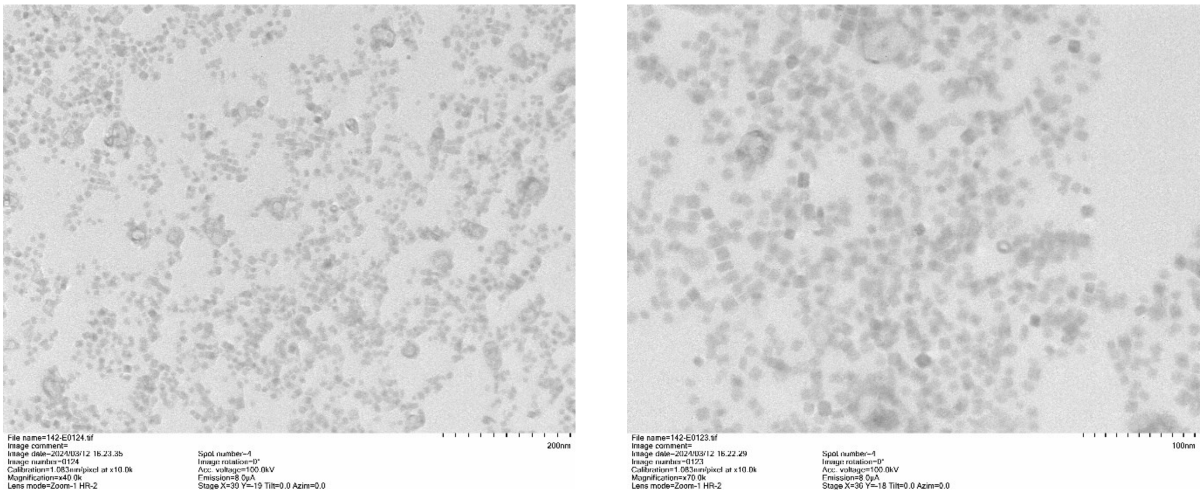

Figure 20: Sample: Reaction with **DDAB-QDs** in hexane. Yield: 60%. Most particles retained their original size and their cubic shape. In a few cases, QDs underwent slight etching.

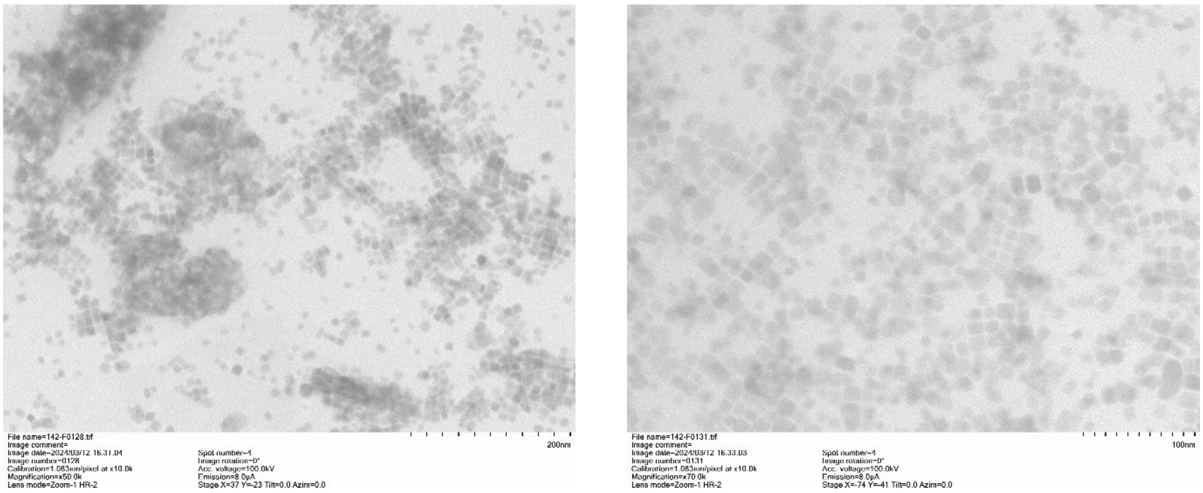

Figure 21: Sample: Reaction with **DDAB-QDs** in CyH. Yield: 72%. The state of the QDs is comparable with TEM image in hexane. Most particles retained their original size and shape.

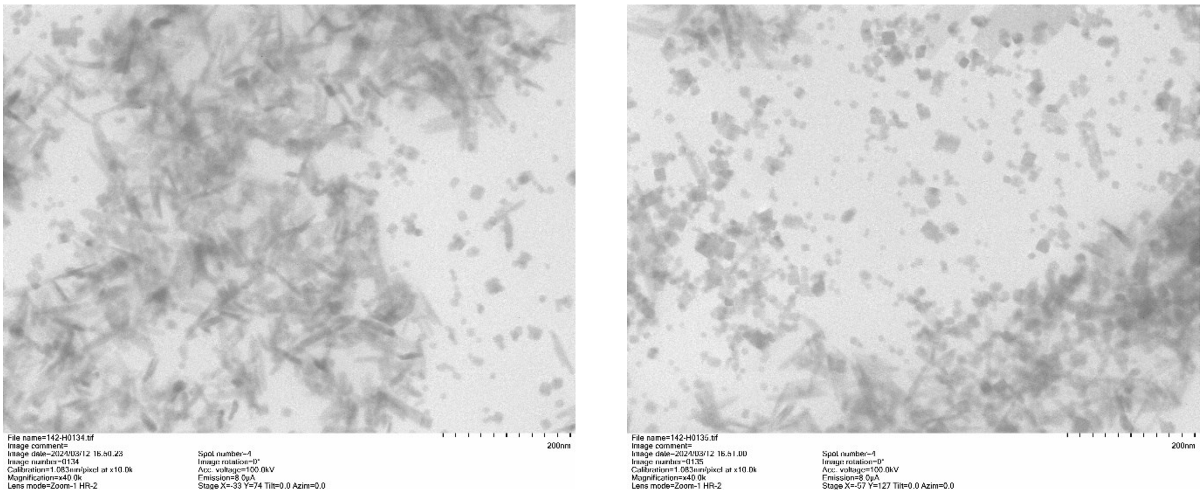

Figure 22: Sample: Reaction with **DDAB-QDs** in MTBE. Yield: 56%. QDs differ significantly from their original size and shape. Sintering along one dimension led to the formation of nanorods (left). Their photocatalytic properties are unknown. Many particles are spherical, and smaller in size than original QDs.

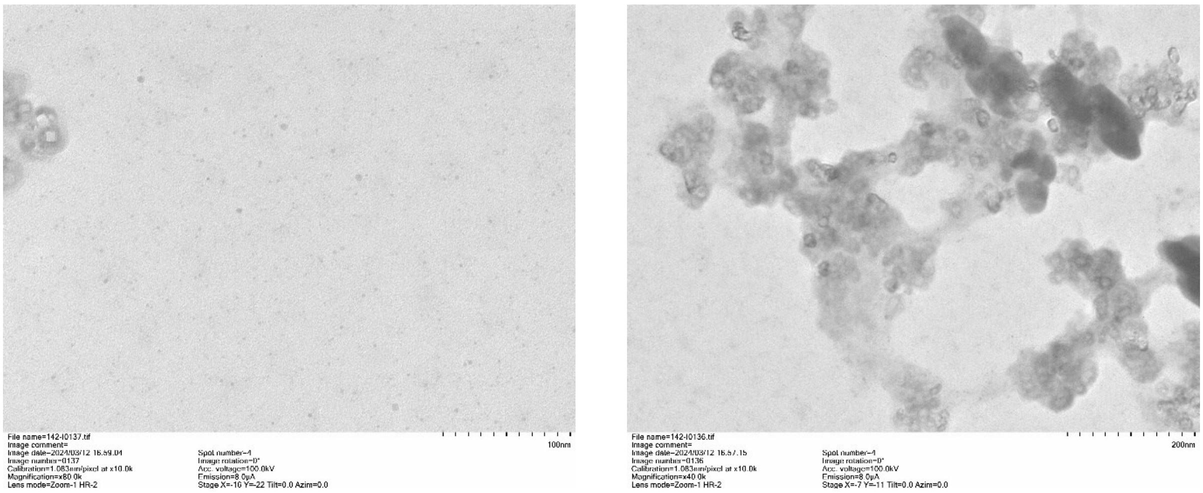

Figure 23: Sample: Reaction with **DDAB-QDs** in THF. Yield: 42%. No QDs with cubic shape or original size could be found. Instead, strongly etched, spherical QDs with a significantly smaller size than original QDs were observed (left). Higher structures (right) consisting of organic residues or sintered QDs were also found.

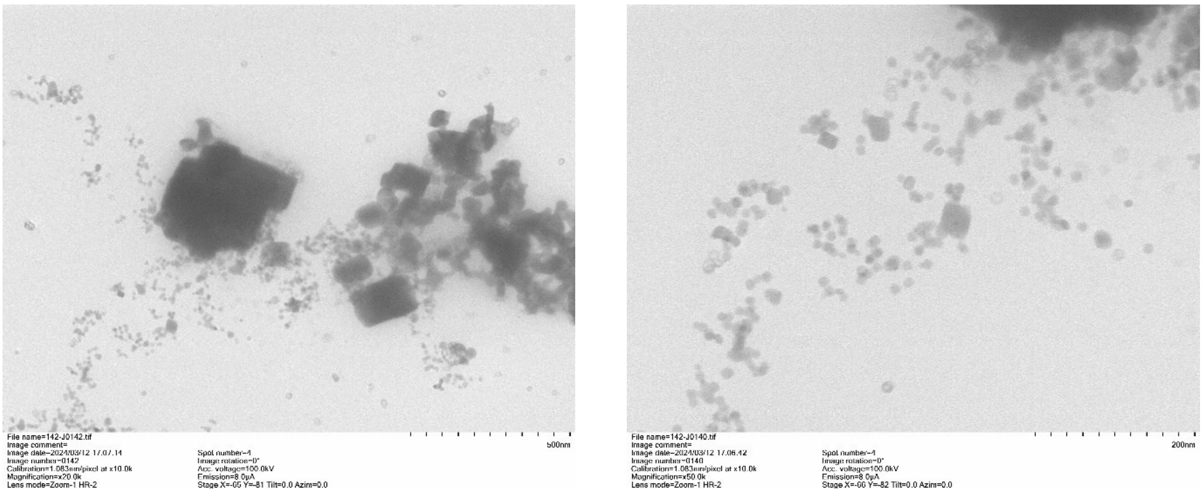

Figure 24: Sample: Reaction with **DDAB-QDs** in 1,4-dioxane. Yield: 83%. QDs are etched and smaller in size. Larger structures were also found.

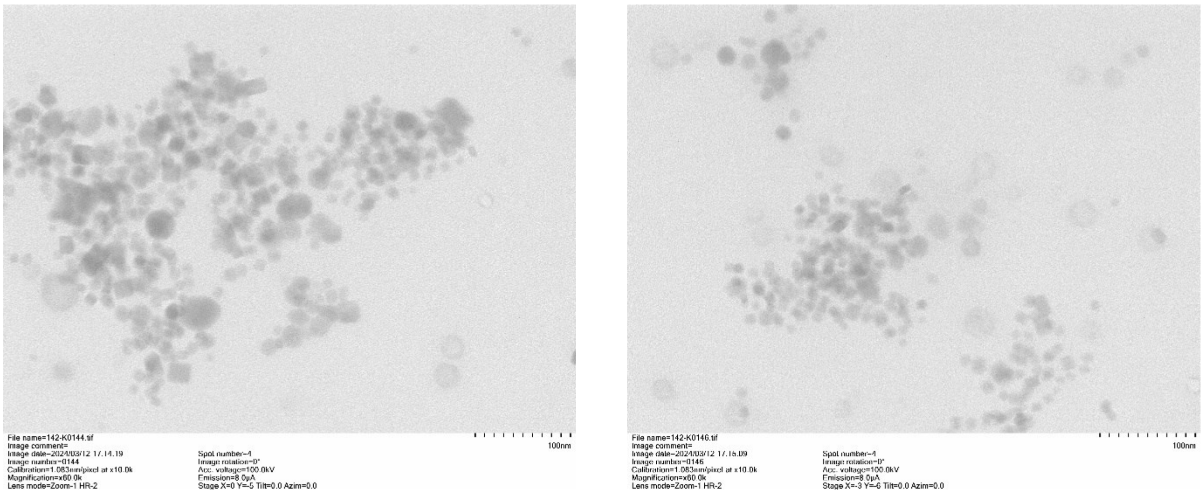

Figure 25: Sample: Reaction with **DDAB-QDs** in CH_2Cl_2. Yield: 74%. QDs are etched with varying shapes and sizes.

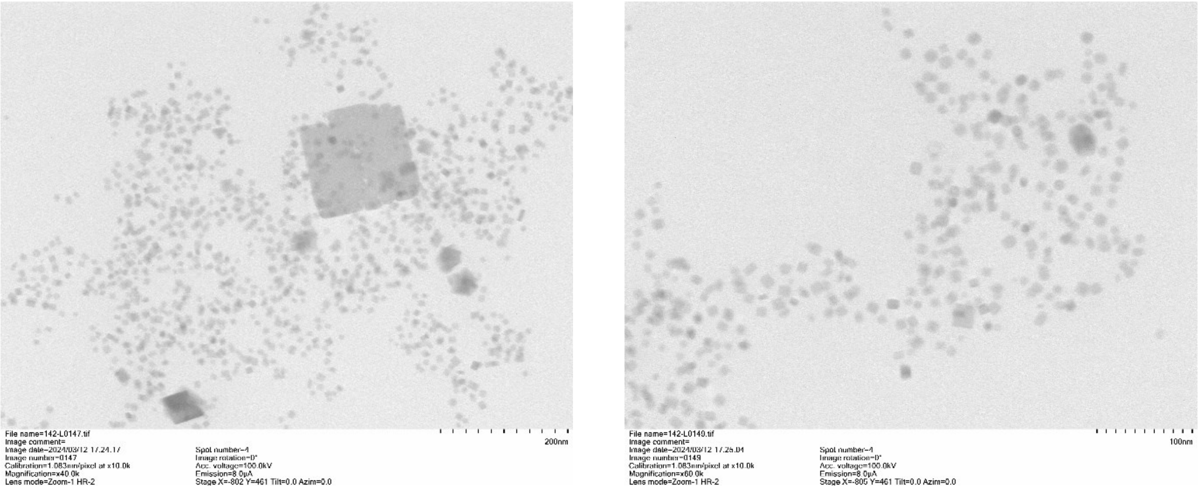

Figure 26: Sample: Reaction with **DDAB-QDs** in $CHCl_3$. Yield: 49%. A distribution between etched QDs, and particles with retained shape and size was observed.

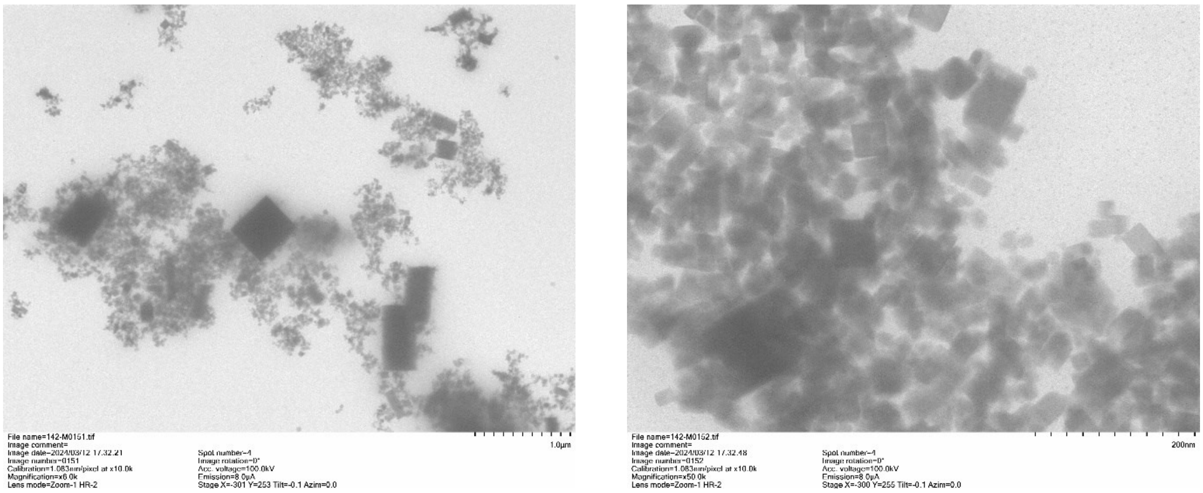

Figure 27: Sample: Reaction with **DDAB-QDs** in EtOAc. Yield: 76%. The polarity of the solvent and apolar alkyl chain of DDAB leads to the formation of QD aggregates. Particles grew in diameter but mostly retained their shape.

BrPEQD is the third best performing catalyst in the solvent and ligand study. TEM images for the reaction mixtures containing **BrPEQD** were measured, for reactions containing >10% yield of product. Subsequently, two TEM images for each reaction mixture will be briefly discussed in the figure caption (Figure 28-Figure 41).

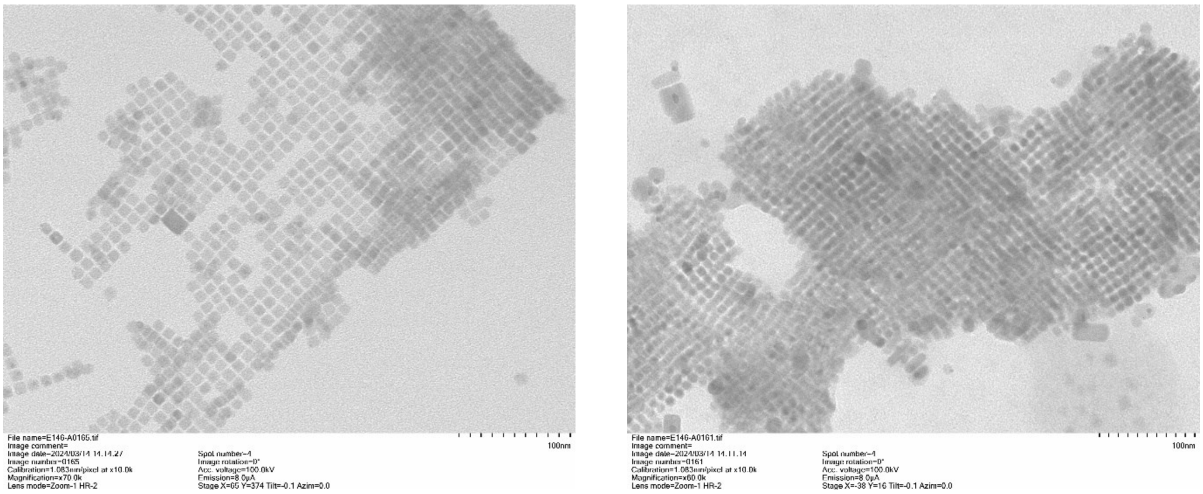

Figure 28: Sample: Reaction with **BRPE-QDs** in PhCl. Yield: 62%. QDs are in excellent condition. Particles retained shape and size.

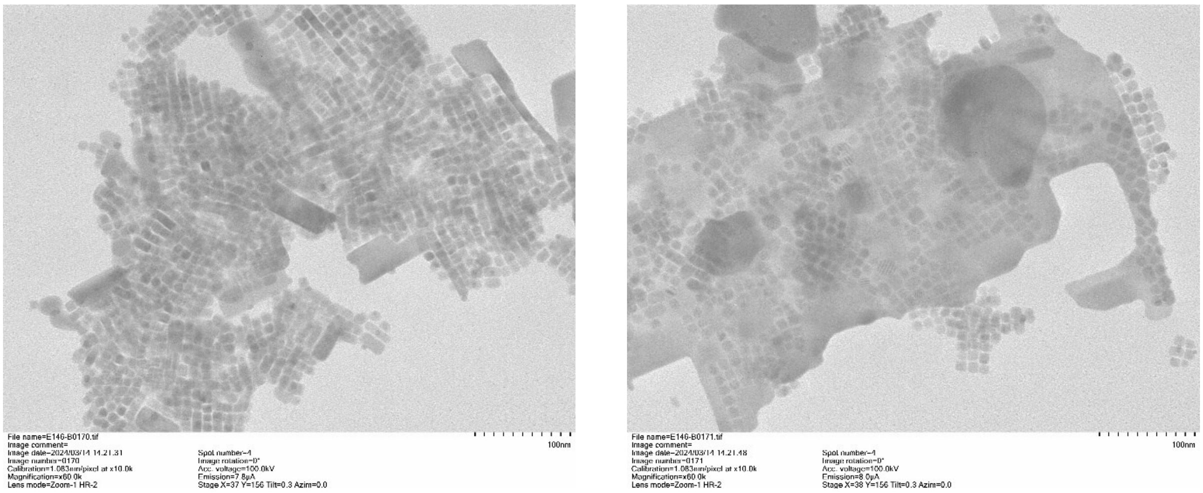

Figure 29: Sample: Reaction with **BRPE-QDs** in PhH. Yield. 53%. QDs mostly retained shape and size. Occasional sintering to larger particles observed. The image on the right likely shows organic residue obstructing the few on the remaining QDs.

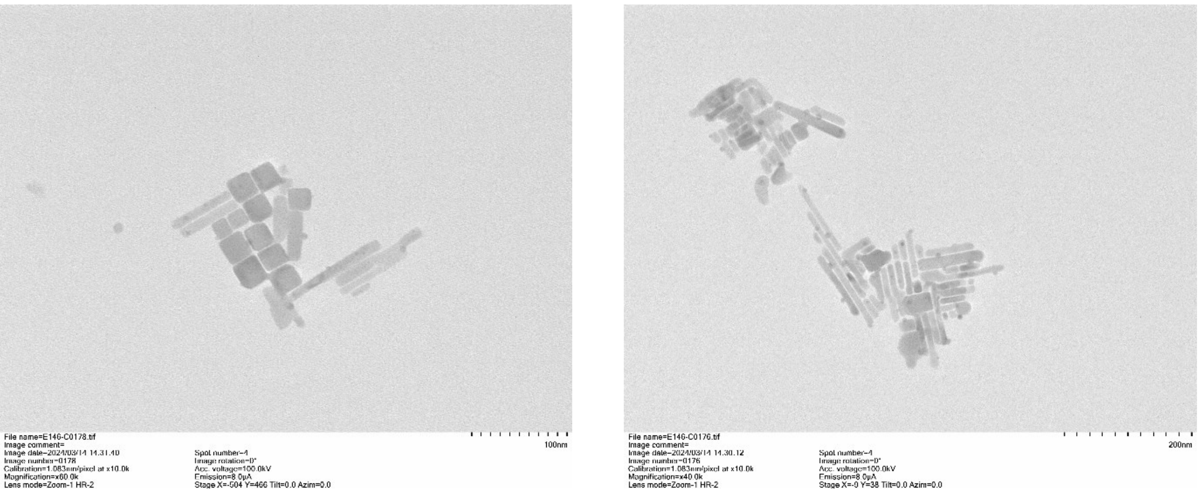

Figure 30: Sample: Reaction with **BRPE-QDs** in PhMe. Yield: 43%. Particles which retained shape but grew in size were found (left). Furthermore, sintering along one dimension was observed obstructing the integrity of the QD and creating nanorods (right).

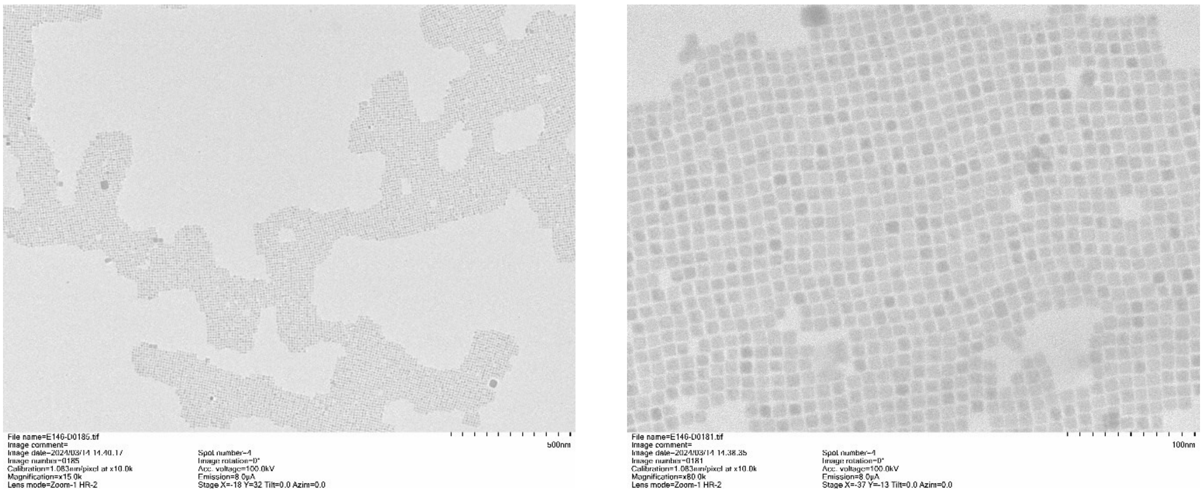

Figure 31: Sample: Reaction with **BRPE-QDs** in $PhCF_3$. Yield: 72%. QDs are in excellent condition. Particles retained shape and size.

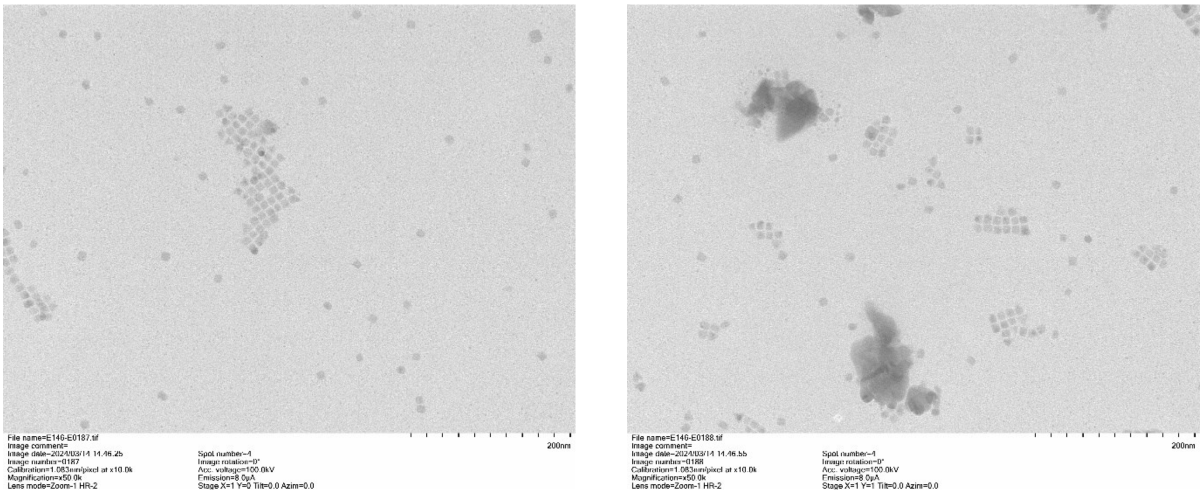

Figure 32: Sample: Reaction with **BRPE-QDs** in hexane. Yield: 48%. Particles with intact shape and size were observed (left). Weak etching for some QDs but also strong etching observed (right TEM image, top left quadrant). Furthermore, sintering to larger QDs was observed.

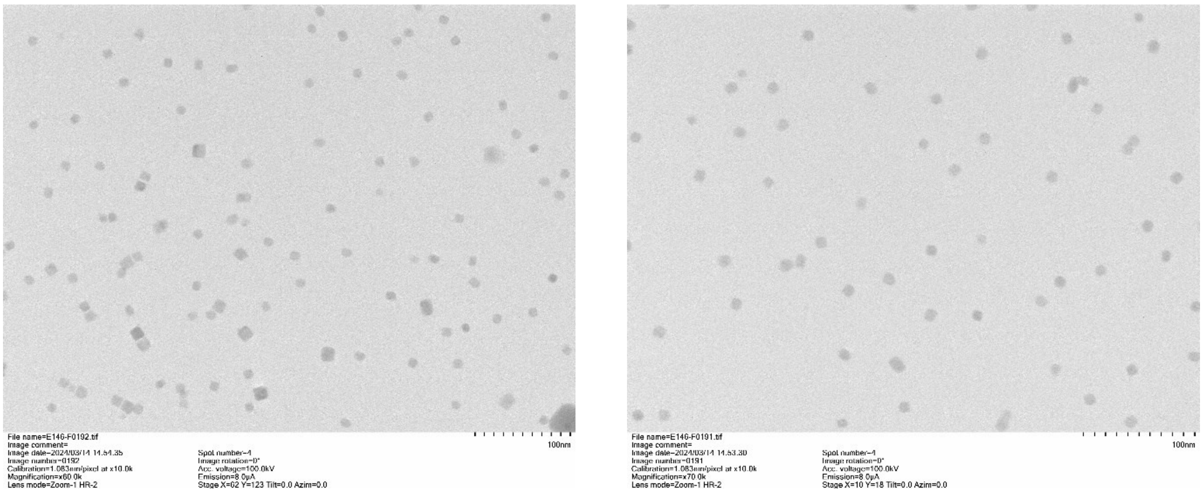

Figure 33: Sample: Reaction with **BRPE-QDs** in CyH. Yield: 67%. Rounded corners of QDs indicate etching. Particles largely maintained their original size.

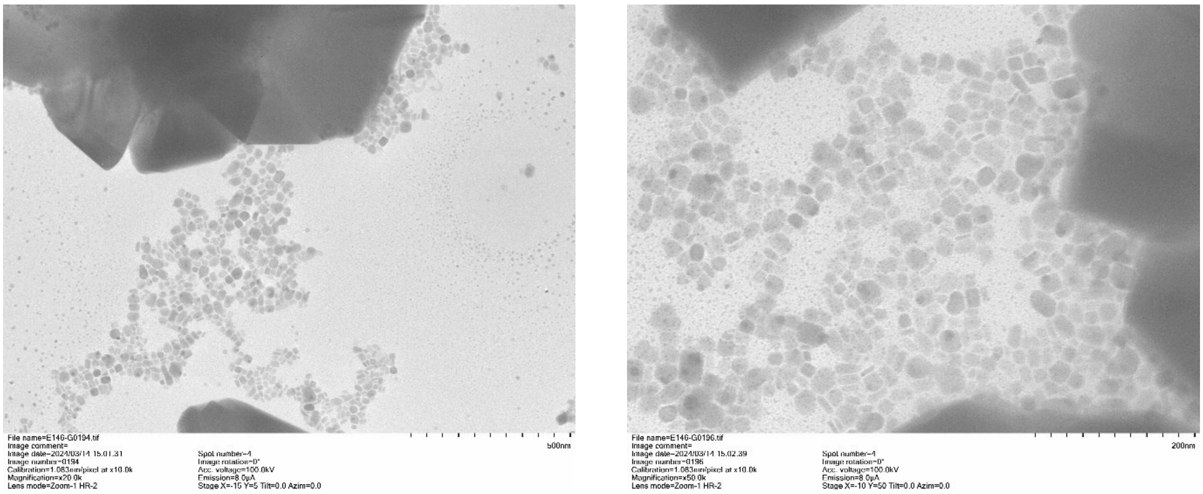

Figure 34: Sample: Reaction with **BRPE-QDs** in Et_2O. Yield: 73%. Organic or inorganic residues obstruct view on QDs. Some particles are partially etched, others strongly decreased in size resulting in particles with <5 nm in size.

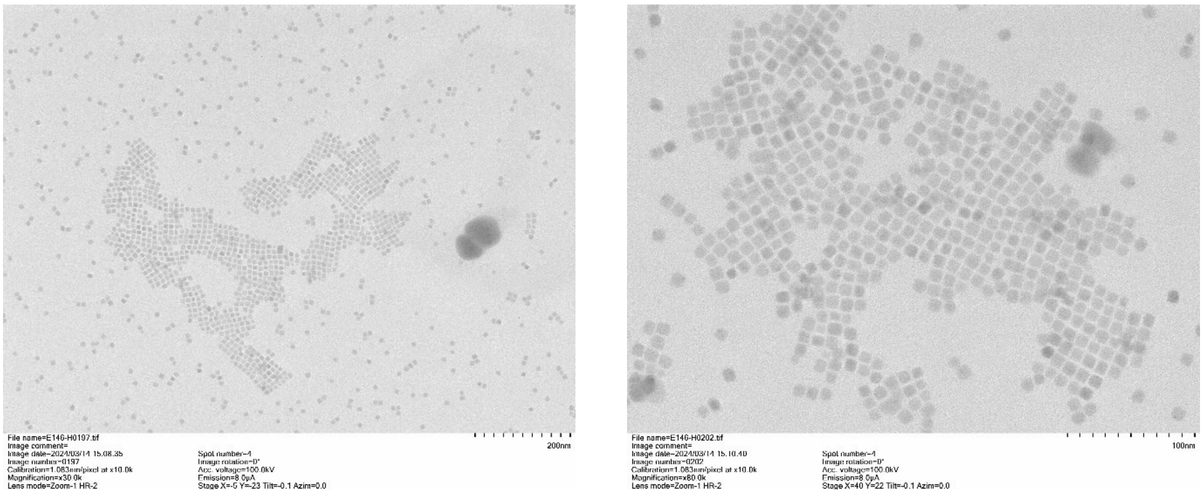

Figure 35: Sample: Reaction with **BRPE-QDs** in MTBE. Yield: 40%. QDs are in excellent condition. Particles retained shape and size.

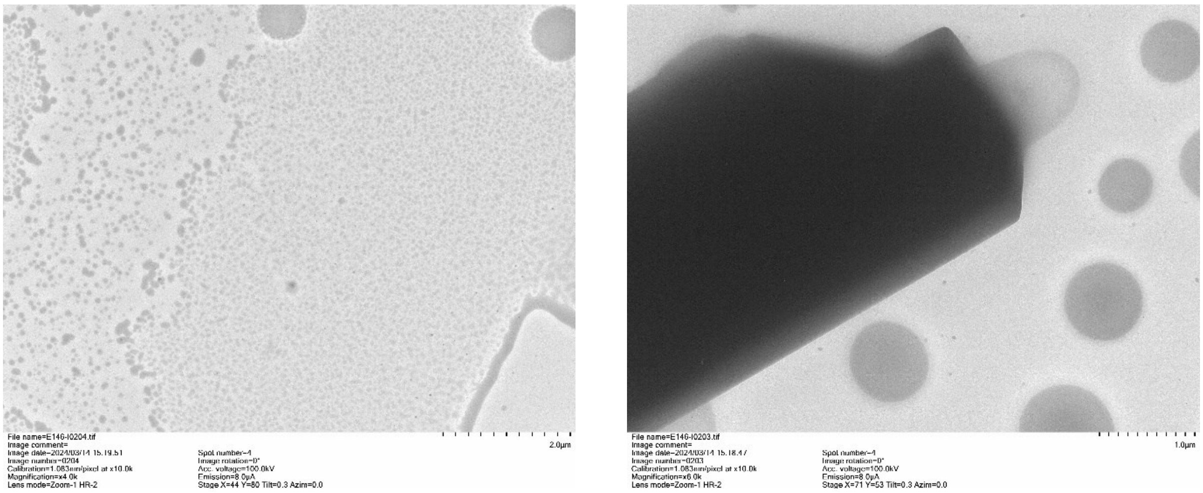

Figure 36: Sample: Reaction with **BRPE-QDs** in THF. Yield: 68%. All observed QDs are spherical and strongly decreased in size. We know that THF can dissolve ions from the surface of the QD which would translate to smaller QDs.

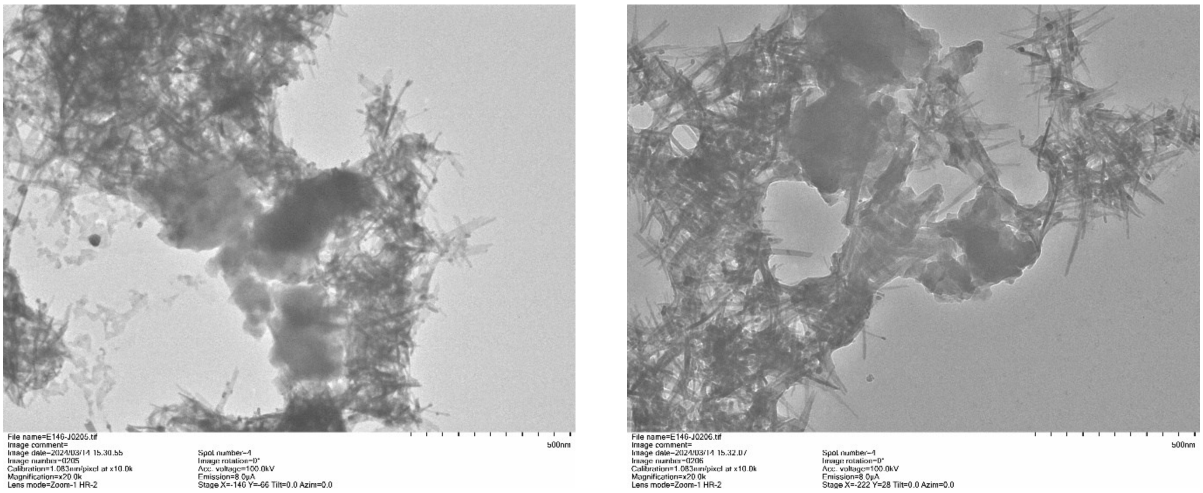

Figure 37: Sample: Reaction with **BRPE-QDs** in 1,4-dioxane. Yield: 14%. No intact QDs were found. Higher structures could indicate organic or inorganic residues. Rod-like structures could indicate nanorod formation.

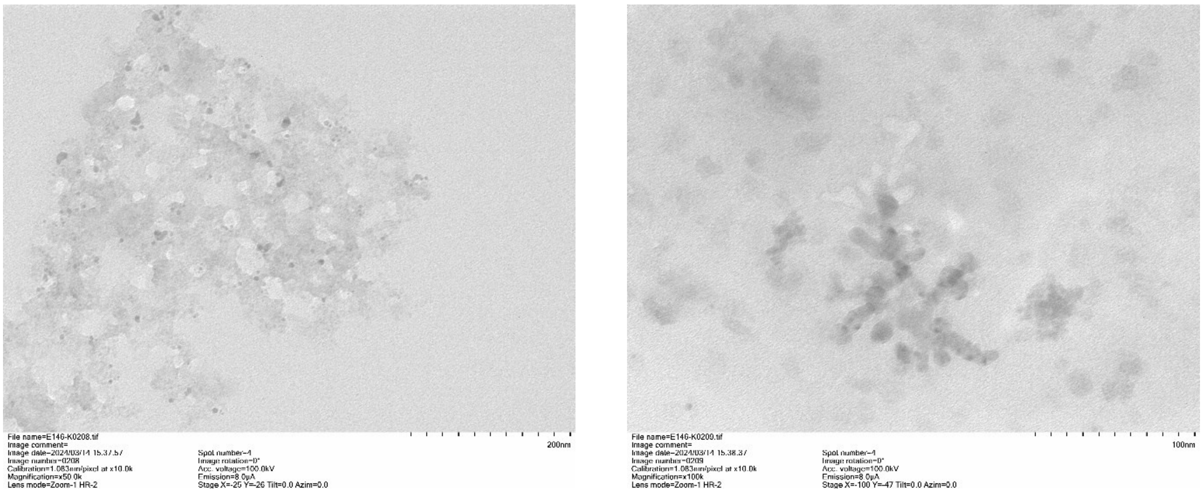

Figure 38: Sample: Reaction with **BRPE-QDs** in DME. Yield: 16%. No intact QDs were found.

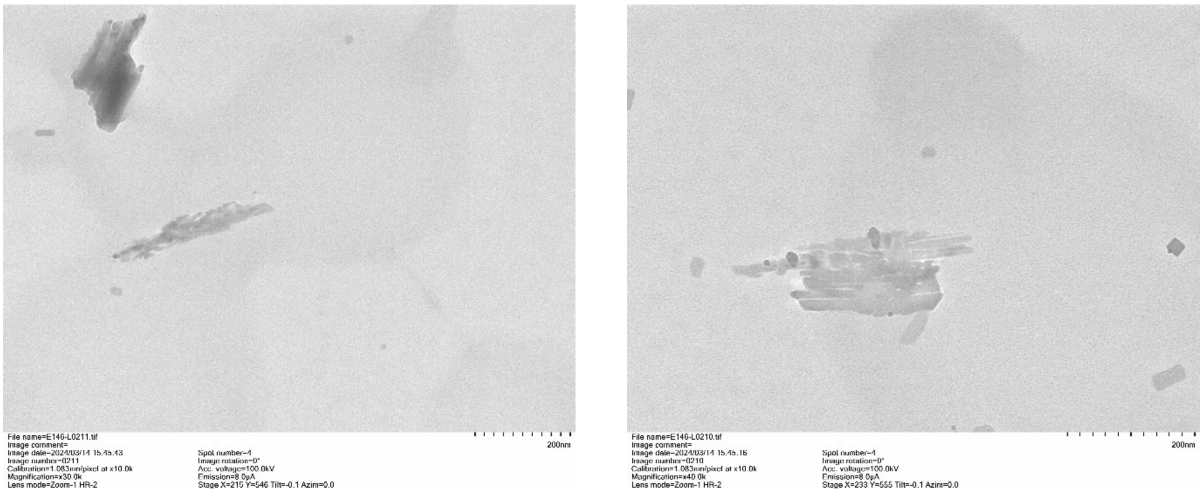

Figure 39: Sample: Reaction with **BRPE-QDs** in CH_2Cl_2. Yield: 51%. Quantum dots are sintered in one dimension and formed nanorods.

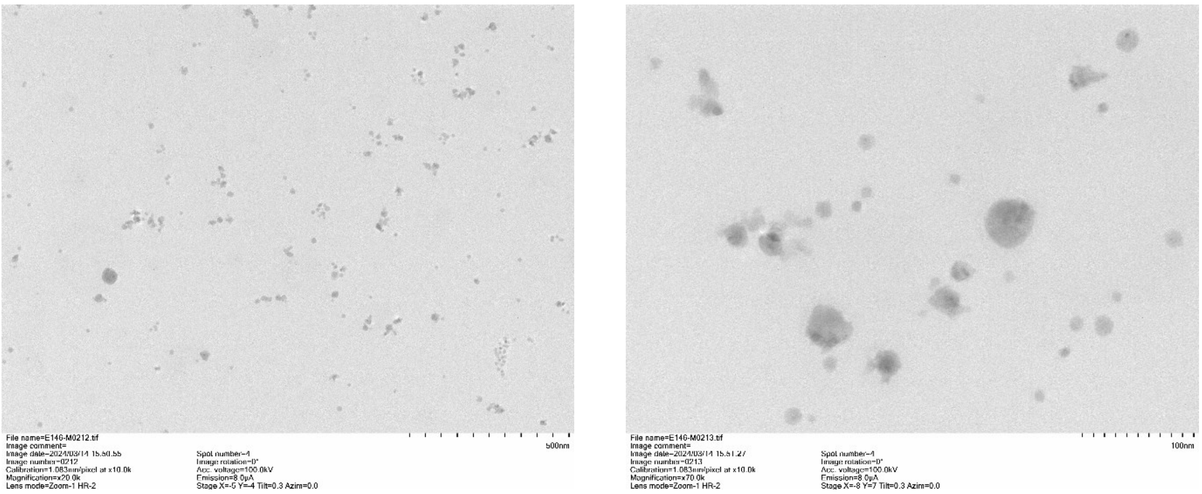

Figure 40: Sample: Reaction with **BRPE-QDs** in $CHCl_3$. Yield: 50%. QDs differ significantly from their original shape and size and have a spherical appearance and a large size distribution (right).

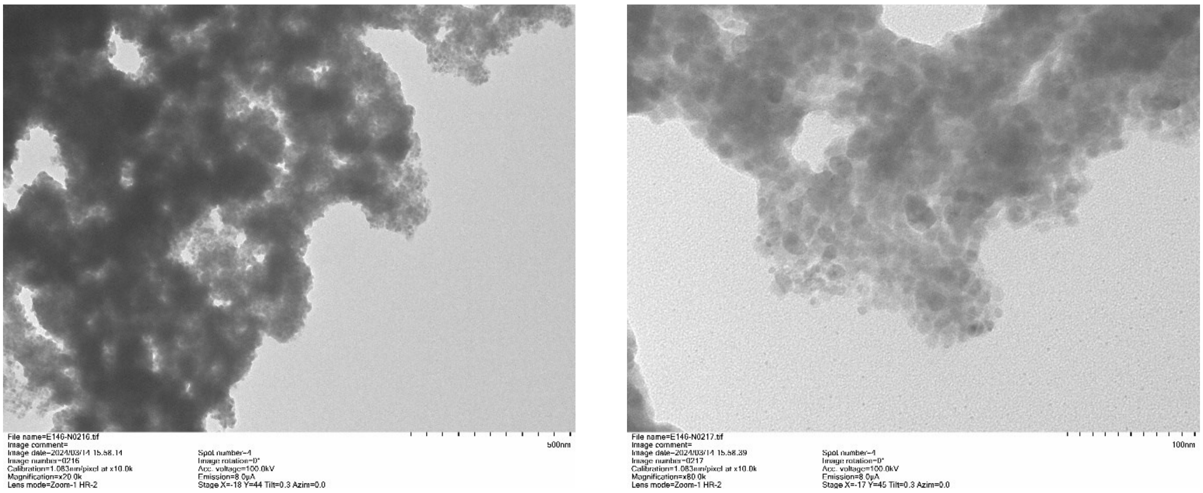

Figure 41: Sample: Reaction with **BRPE-QDs** in EtOAc. Yield: 32%. The high solvent polarity and apolar tail of **BRPE** leads to the formation of multilayer aggregates. QDs are etched but mostly maintained their size.

9. Additional STEM images

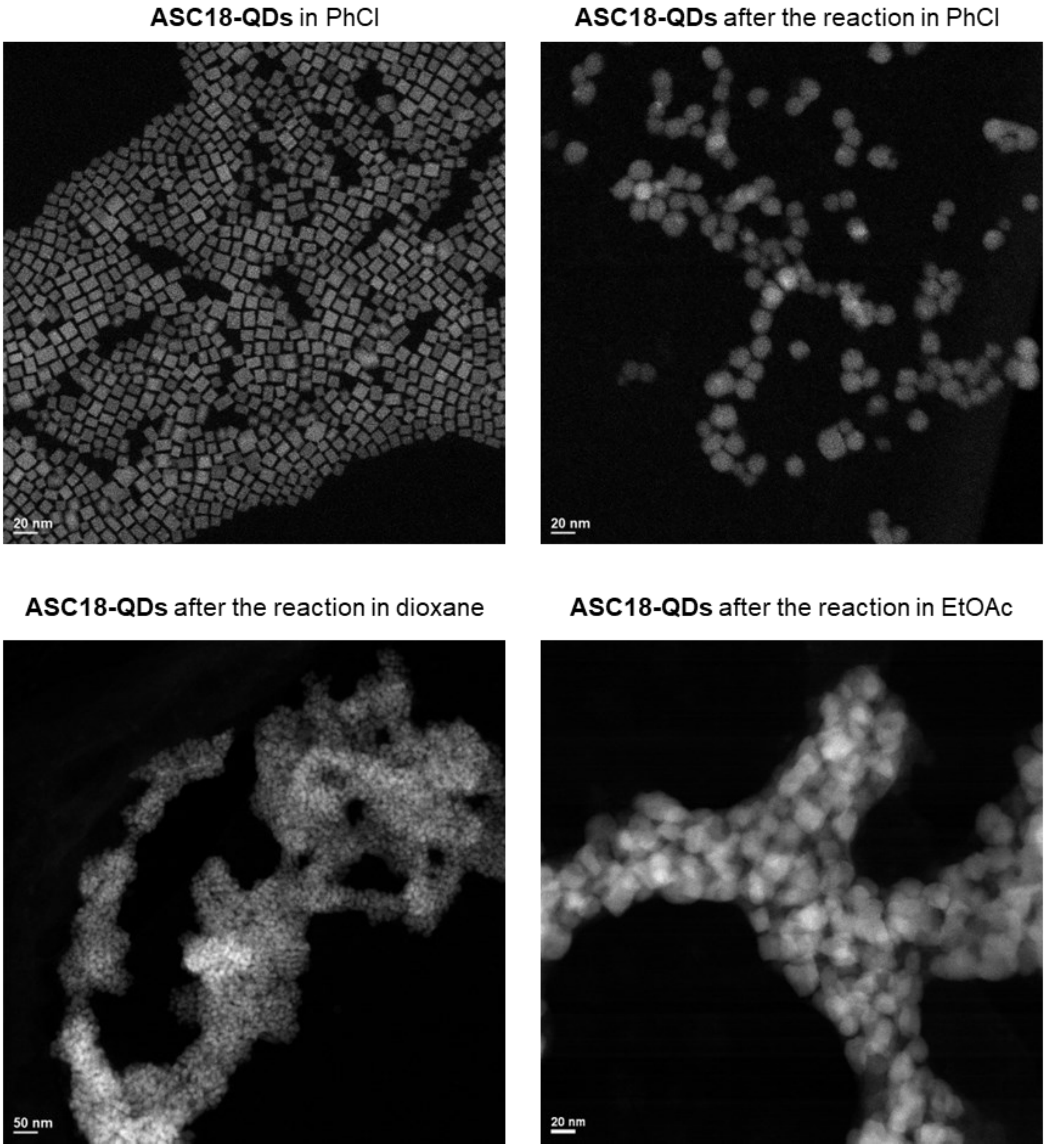

Figure 42: Full size STEM images shown in figure 4b of the main manuscript.

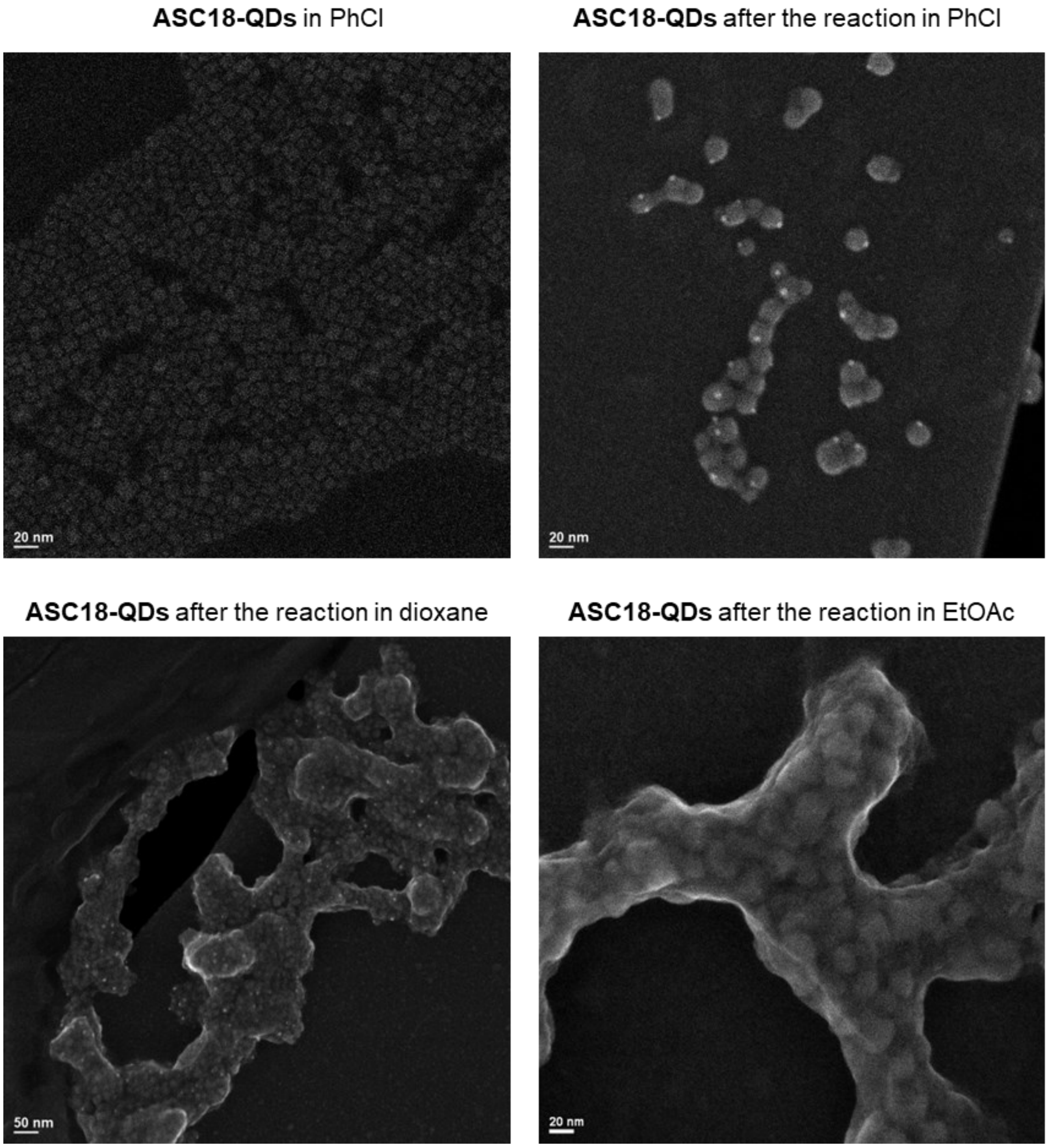

Figure 43: Secondary electron images recorded in addition to the images shown in figure 4b of the main manuscript.

10. UV-Vis and PL spectra of ASC18-QDs after the reaction

Our study on the performance of $CsPbBr_3$ QDs under anhydrous conditions and in the presence of 1.00 µL water (Chapter 7) revealed that **ASC18-QDs** perform best (>50% yield in the most amounts of solvent). After investigating the shape and size of **ASC18-QDs** under anhydrous conditions via TEM, we measured the UV-Vis and photoluminescence spectra of the reaction mixtures. The sample preparation (transfer of reaction mixture into a cuvette) as well as the UV-Vis and PL measurement were conducted under ambient conditions.

Note: The freshly prepared reaction vials were only opened immediately before UV-Vis and PL measurement. This prevents further alteration of the LHP QD through air or moisture.

Reaction Conditions: Anhydrous

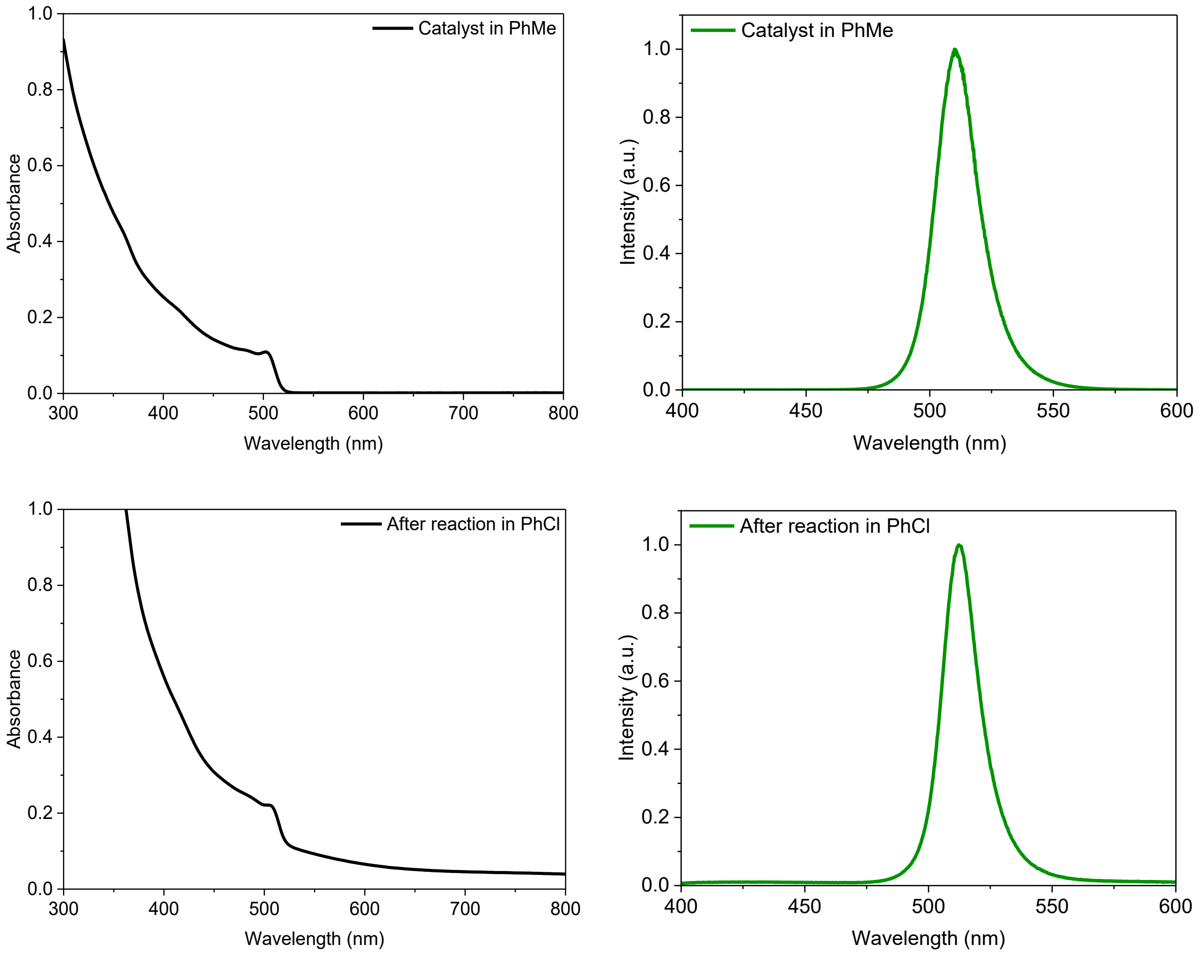

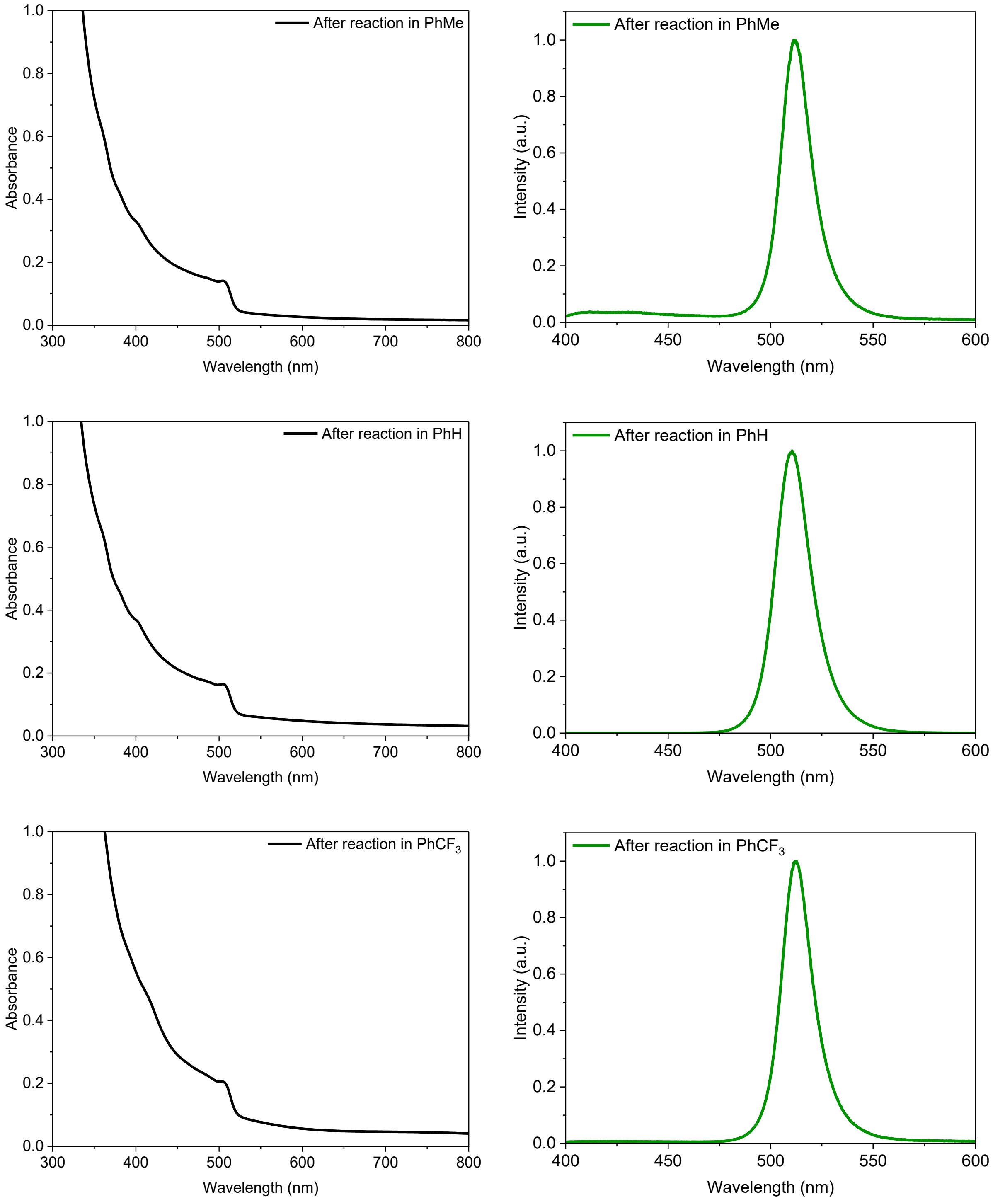
After reaction in PhMe
Absorbance
Wavelength (nm)
After reaction in PhMe
Intensity (a.u.)
Wavelength (nm)
After reaction in PhH
Absorbance
Wavelength (nm)
After reaction in PhH
Intensity (a.u.)
Wavelength (nm)
After reaction in $PhCF_3$
Absorbance
Wavelength (nm)
After reaction in $PhCF_3$
Intensity (a.u.)
Wavelength (nm)

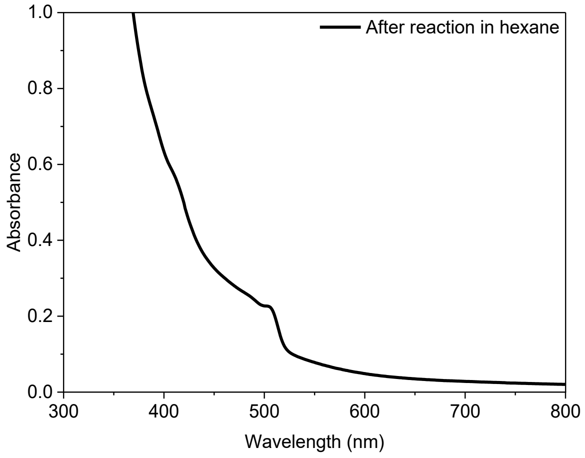
After reaction in hexane
Absorbance
Wavelength (nm)
1.0
0.8
0.6
0.4
0.2
0.0
300
400
500
600
700
800

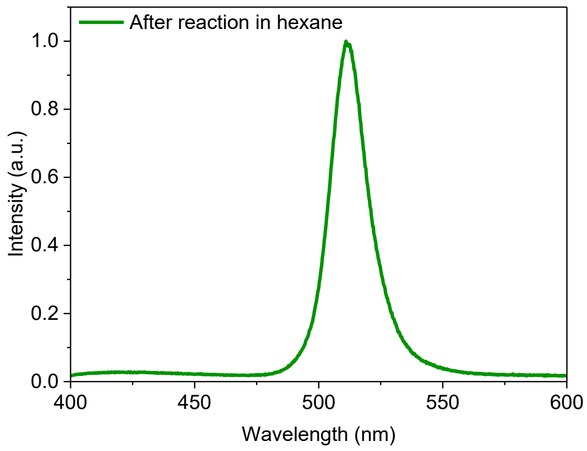
After reaction in hexane
Intensity (a.u.)
Wavelength (nm)
1.0
0.8
0.6
0.4
0.2
0.0
400
450
500
550
600

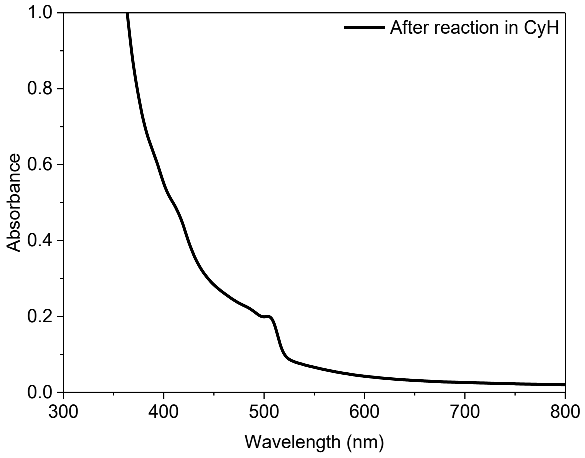
After reaction in CyH
Absorbance
Wavelength (nm)
1.0
0.8
0.6
0.4
0.2
0.0
300
400
500
600
700
800

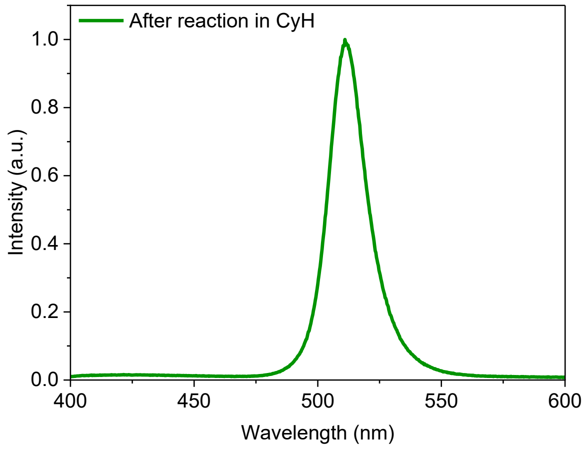
After reaction in CyH
Intensity (a.u.)
Wavelength (nm)
1.0
0.8
0.6
0.4
0.2
0.0
400
450
500
550
600

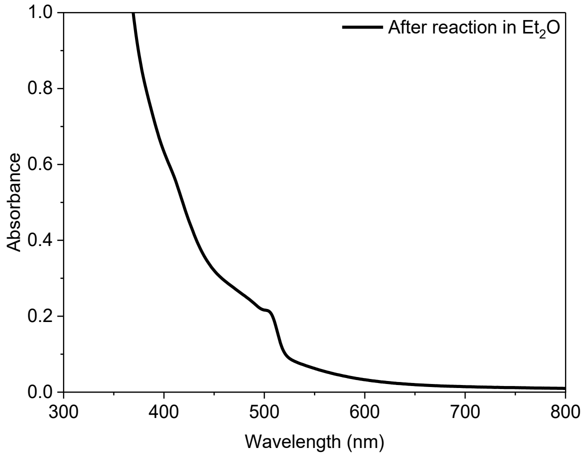
After reaction in Et_2O
Absorbance
Wavelength (nm)
1.0
0.8
0.6
0.4
0.2
0.0
300
400
500
600
700
800

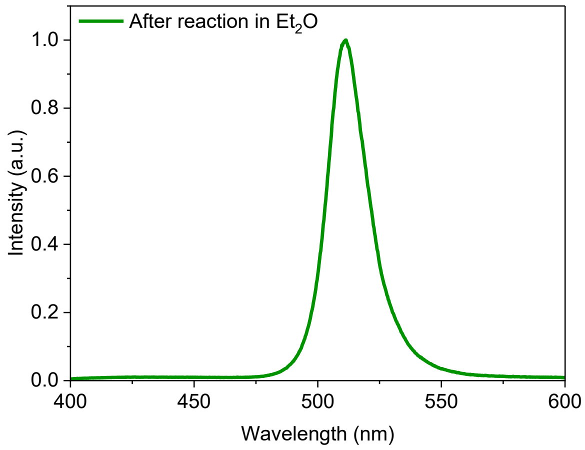
After reaction in Et_2O
Intensity (a.u.)
Wavelength (nm)
1.0
0.8
0.6
0.4
0.2
0.0
400
450
500
550
600

UV-Vis and PL spectra of **ASC18-QDs** after the reaction

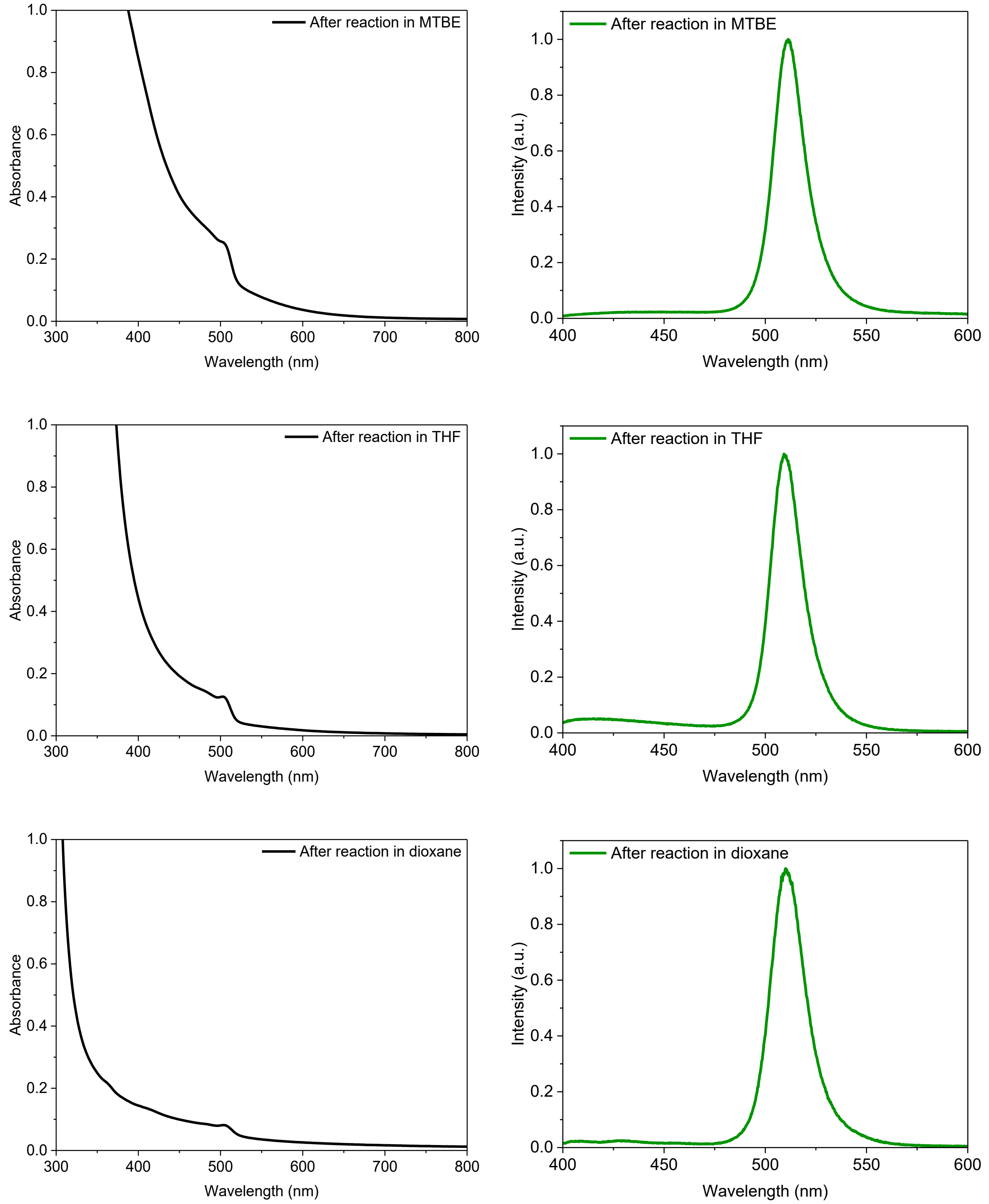

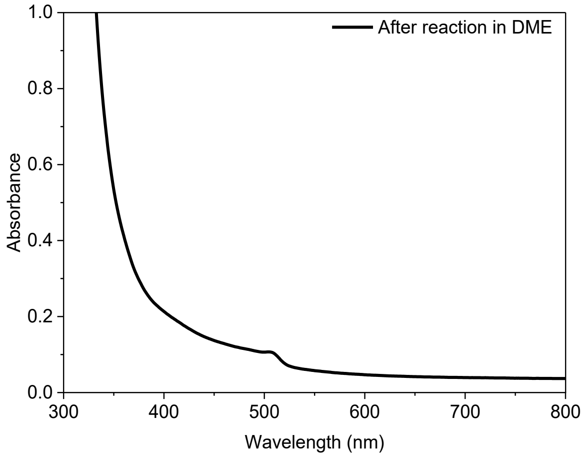
After reaction in DME
Absorbance
Wavelength (nm)

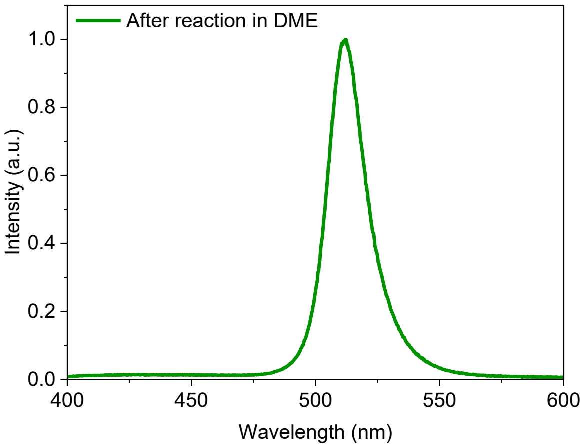
After reaction in DME
Intensity (a.u.)
Wavelength (nm)

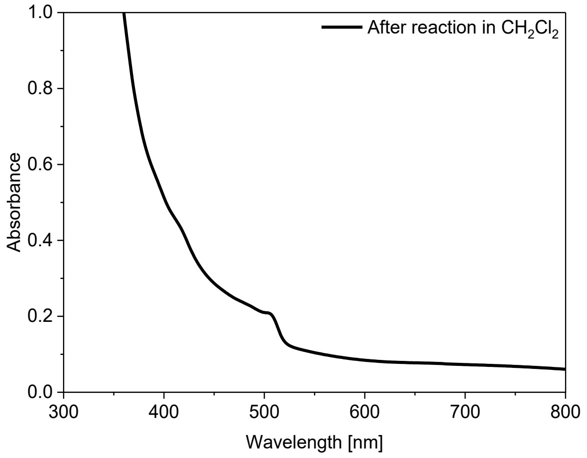
After reaction in CH_2Cl_2
Absorbance
Wavelength [nm]

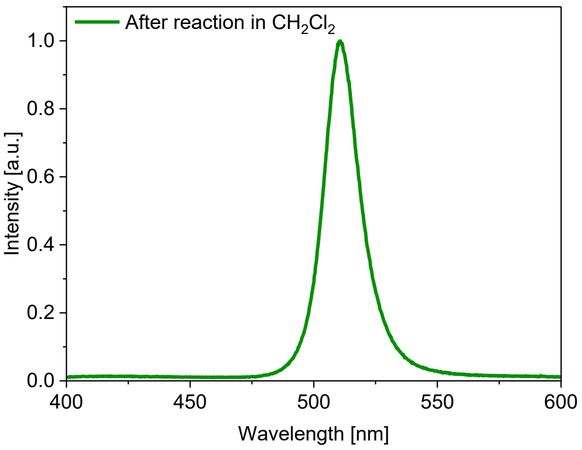
After reaction in CH_2Cl_2
Intensity [a.u.]
Wavelength [nm]

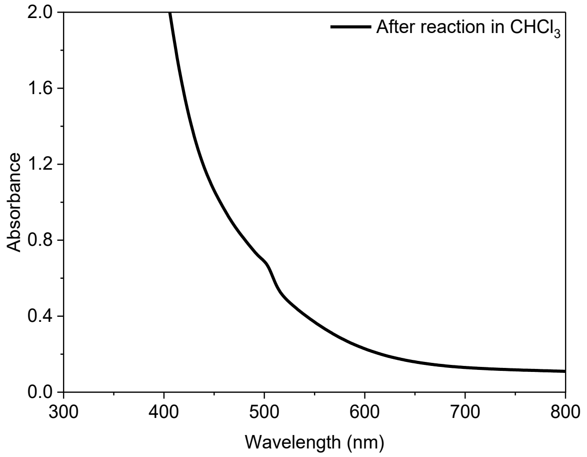
After reaction in $CHCl_3$
Absorbance
Wavelength (nm)

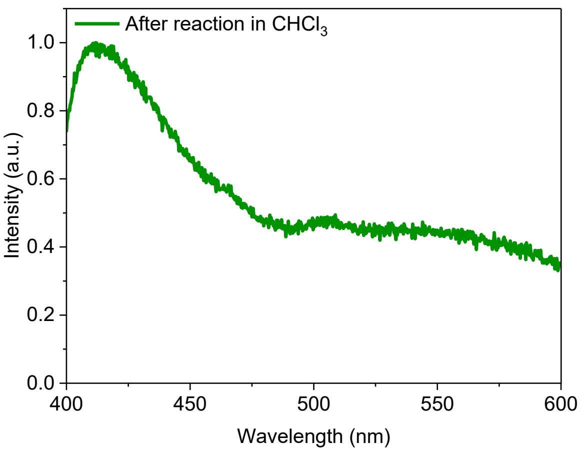
After reaction in $CHCl_3$
Intensity (a.u.)
Wavelength (nm)

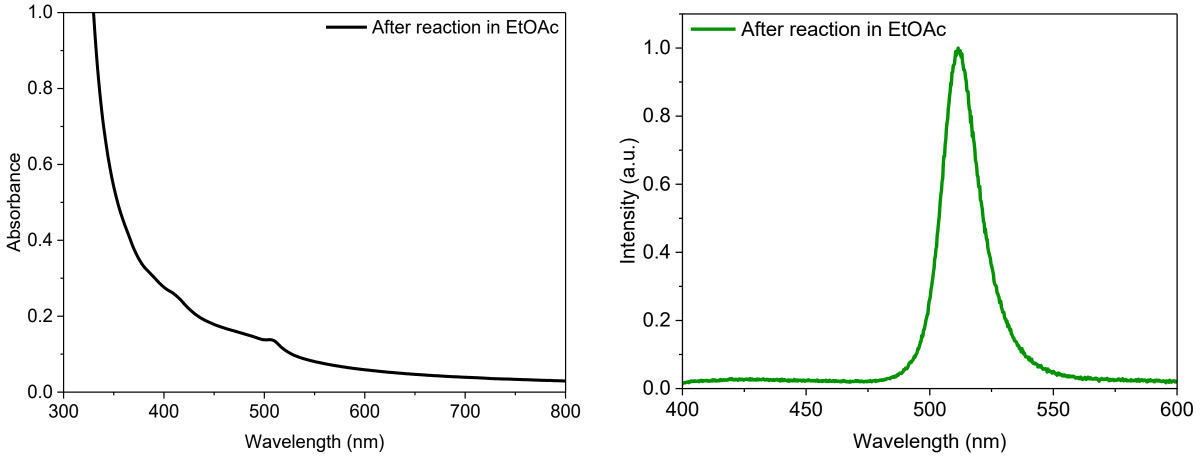

Table 5: Characteristic UV-Vis and PL signals of **ASC18-QDs** under anhydrous conditions.

Solvent	Excitonic Peak from 2nd Derivative (nm)	PL Maximum (nm)	Yield
PhMe (Only Catalyst)	506	511	–
PhCl	509	512	84%
PhH	508	511	39%
PhMe	509	512	55%
$PhCF_3$	509	513	79%
Hexane	508	511	80%
CyH	509	511	78%
Et_2O	508	511	76%
MTBE	507	511	63%
THF	506	509	73%
Dioxane	507	510	64%
DME	509	512	78%
CH_2Cl_2	509	511	54%
$CHCl_3$	–	–	34%
EtOAc	509	512	86%

Analysis of the UV-Vis spectra revealed a bathochromic shift in most reaction media when compared to native **ASC18-QDs** in PhMe. The only exception was $CHCl_3$ and THF. For $CHCl_3$, no characteristic absorption band was observed in the UV-Vis spectrum. Photoluminescence was also not detected, indicating catalyst decomposition which could be an explanation for the comparatively low yield of benzyl bromide **2a** in $CHCl_3$ (34%). For THF a hypsochromic shift was observed in the UV-Vis spectrum. Prior observations by our group suggests the formation of a $THF \cdot PbBr_2$ complex which would lead to the dissolution of the $CsPbBr_3$ QD over time.

Analysis of the PL spectra revealed a similar trend: Only minute bathochromic and hypsochromic shifts were observed. Taken together, we concluded that **ASC18-QDs** tolerate an abundance of solvents, with the $CsPbBr_3$ QD undergoing only minor changes in size.

Next, we were interested how 1.00 µL H_2O in the reaction mixture affected the size of **ASC18-QDs** over the course of the reaction. We consequently measured UV-Vis and PL spectra of these reaction mixtures which are subsequently reported.

Reaction Conditions: 1000 ppm Water

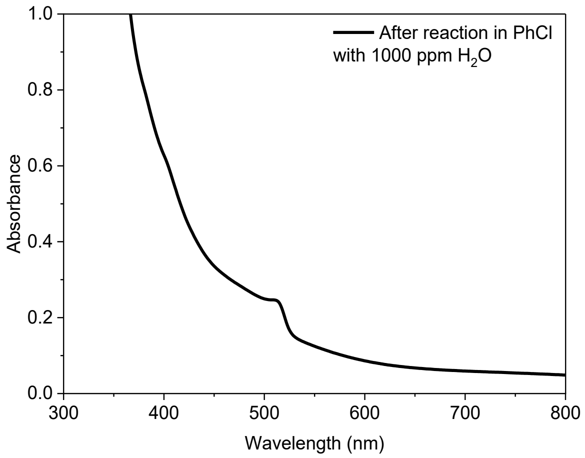

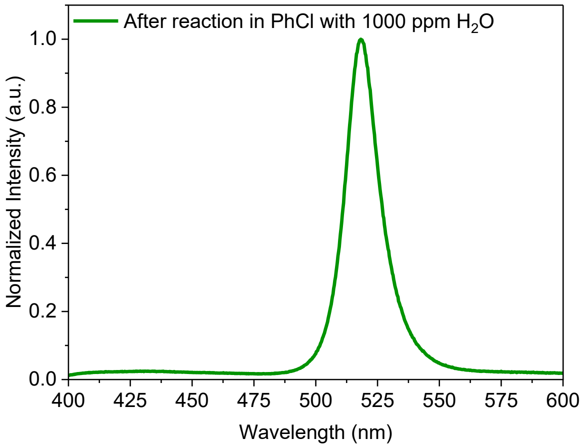

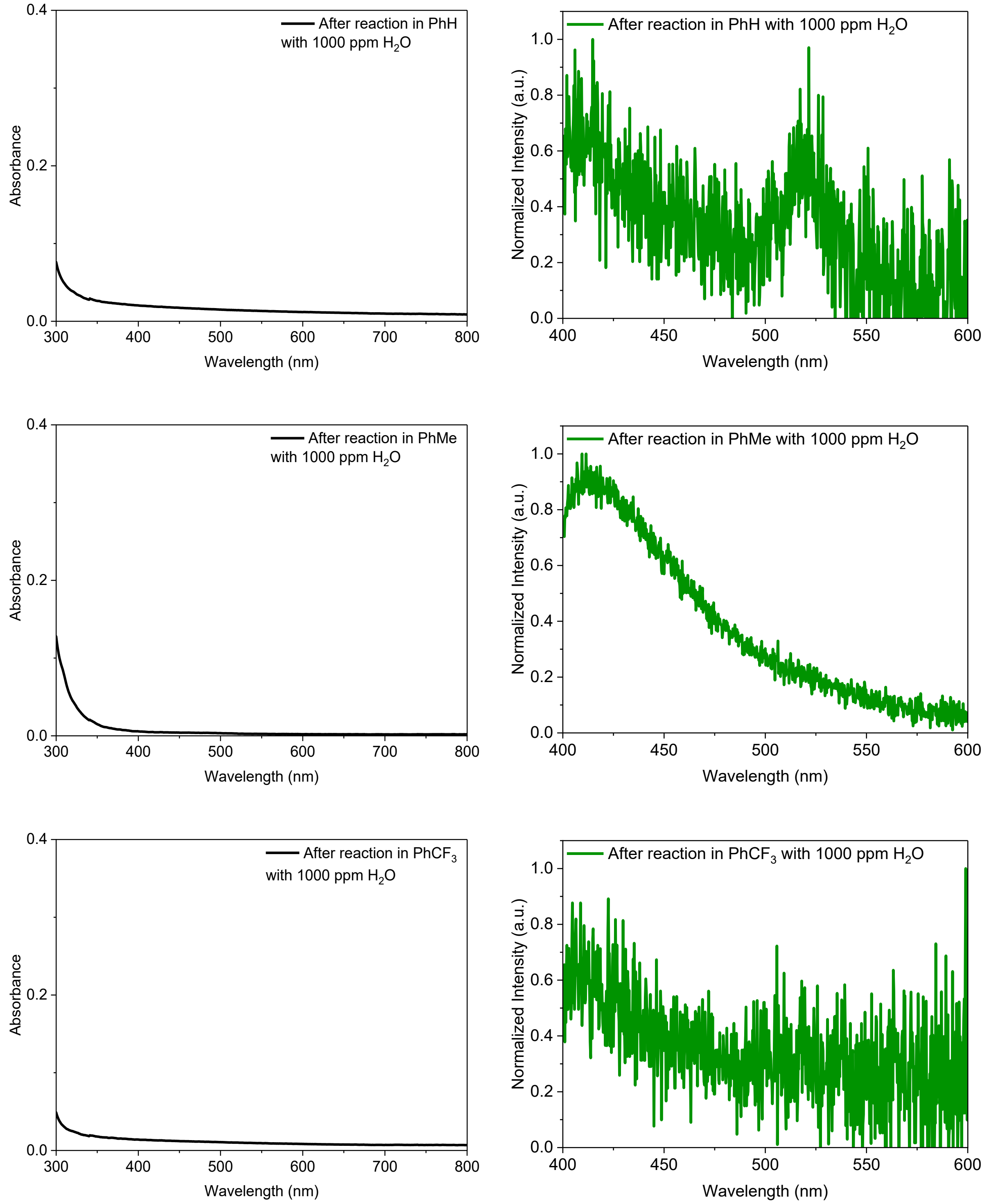
After reaction in PhH with 1000 ppm H_2O
Absorbance
Wavelength (nm)
After reaction in PhH with 1000 ppm H_2O
Normalized Intensity (a.u.)
Wavelength (nm)
After reaction in PhMe with 1000 ppm H_2O
Absorbance
Wavelength (nm)
After reaction in PhMe with 1000 ppm H_2O
Normalized Intensity (a.u.)
Wavelength (nm)
After reaction in $PhCF_3$ with 1000 ppm H_2O
Absorbance
Wavelength (nm)
After reaction in $PhCF_3$ with 1000 ppm H_2O
Normalized Intensity (a.u.)
Wavelength (nm)

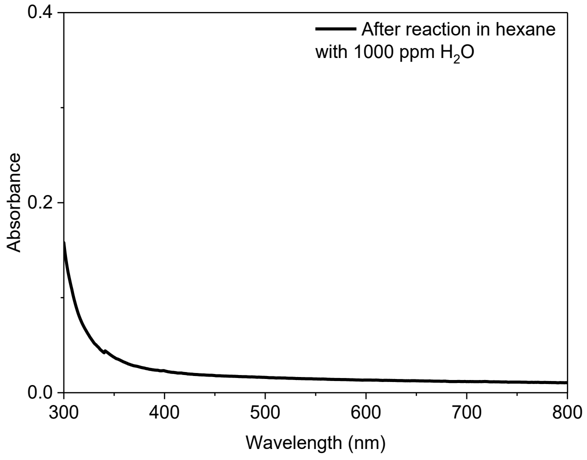
After reaction in hexane
with 1000 ppm H_2O
Absorbance
Wavelength (nm)
0.0
0.2
0.4
300
400
500
600
700
800

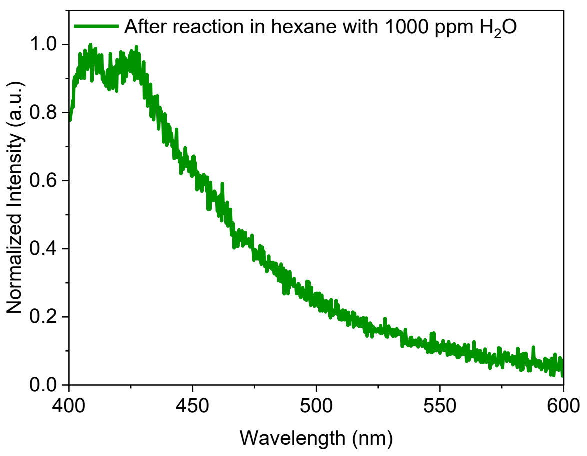
After reaction in hexane with 1000 ppm H_2O
Normalized Intensity (a.u.)
Wavelength (nm)
0.0
0.2
0.4
0.6
0.8
1.0
400
450
500
550
600

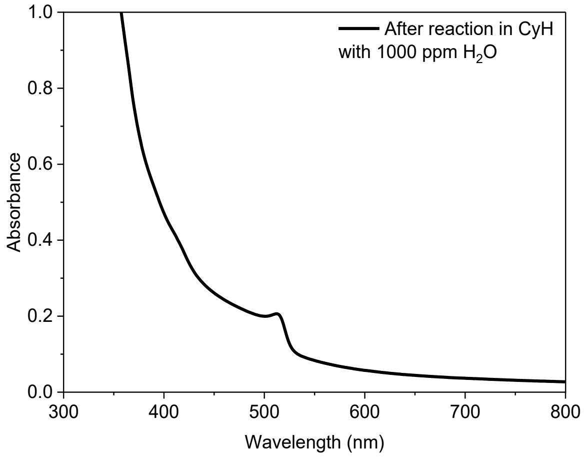
After reaction in CyH
with 1000 ppm H_2O
Absorbance
Wavelength (nm)
0.0
0.2
0.4
0.6
0.8
1.0
300
400
500
600
700
800

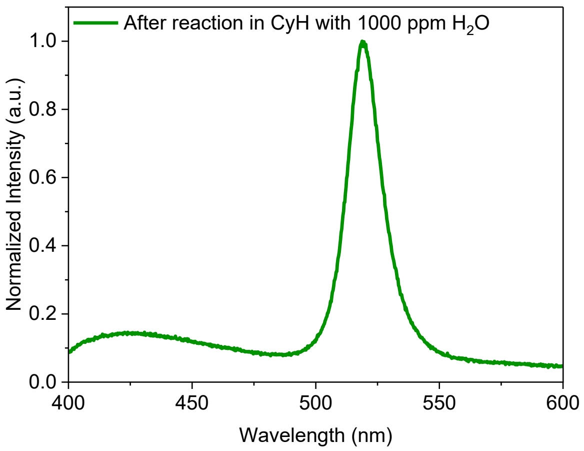
After reaction in CyH with 1000 ppm H_2O
Normalized Intensity (a.u.)
Wavelength (nm)
0.0
0.2
0.4
0.6
0.8
1.0
400
450
500
550
600

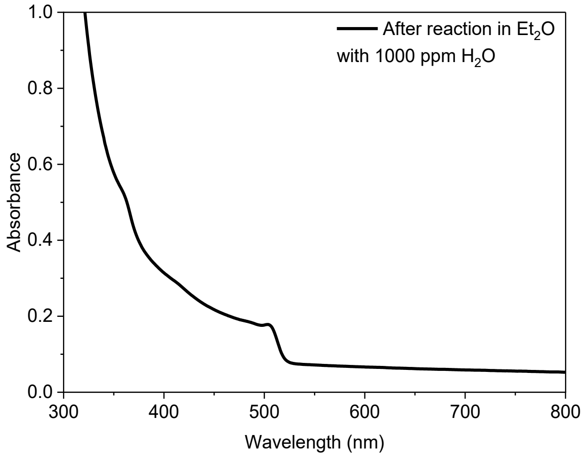
After reaction in Et_2O
with 1000 ppm H_2O
Absorbance
Wavelength (nm)
0.0
0.2
0.4
0.6
0.8
1.0
300
400
500
600
700
800

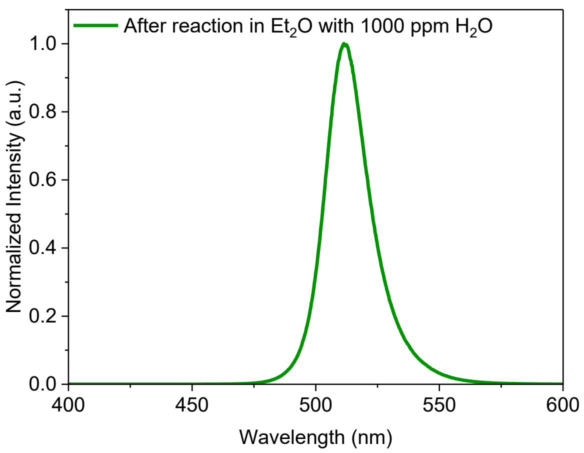
After reaction in Et_2O with 1000 ppm H_2O
Normalized Intensity (a.u.)
Wavelength (nm)
0.0
0.2
0.4
0.6
0.8
1.0
400
450
500
550
600

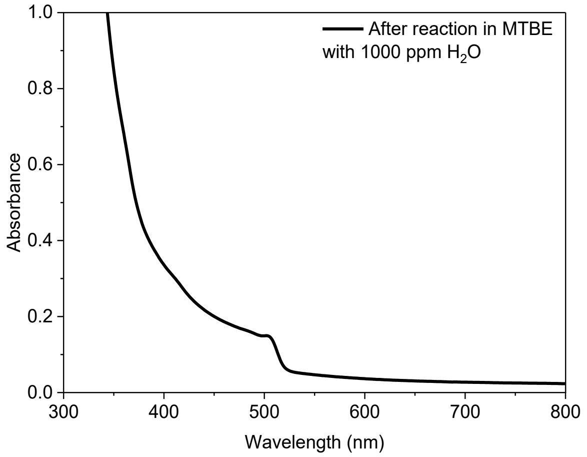
After reaction in MTBE with 1000 ppm H_2O
Absorbance
Wavelength (nm)

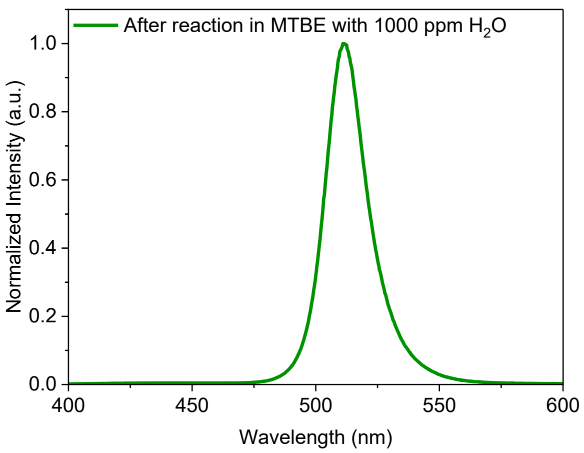
After reaction in MTBE with 1000 ppm H_2O
Normalized Intensity (a.u.)
Wavelength (nm)

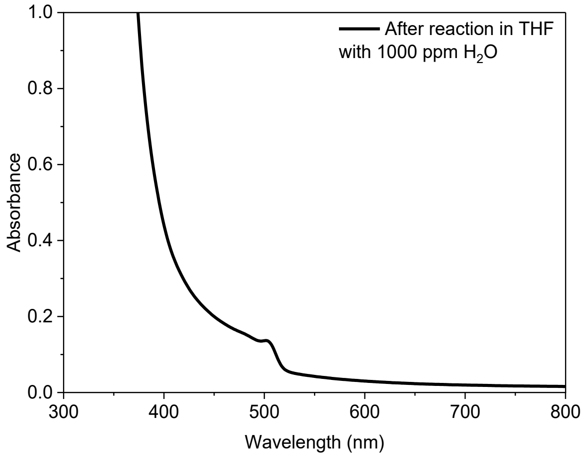
After reaction in THF with 1000 ppm H_2O
Absorbance
Wavelength (nm)

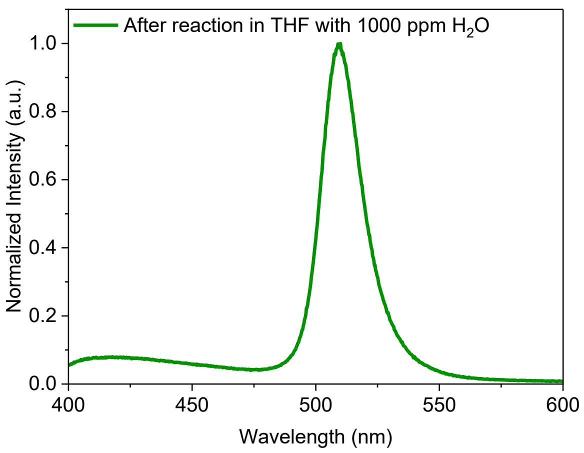
After reaction in THF with 1000 ppm H_2O
Normalized Intensity (a.u.)
Wavelength (nm)

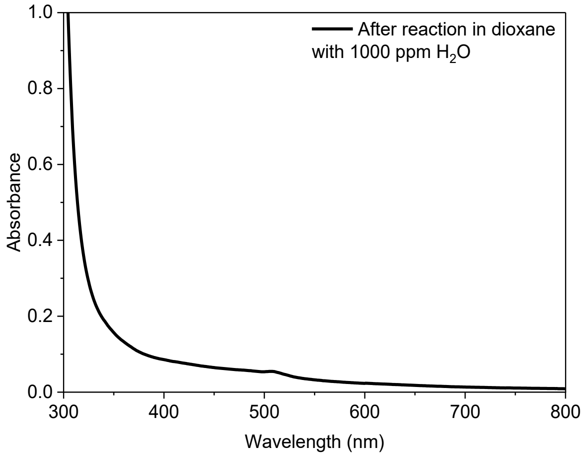
After reaction in dioxane with 1000 ppm H_2O
Absorbance
Wavelength (nm)

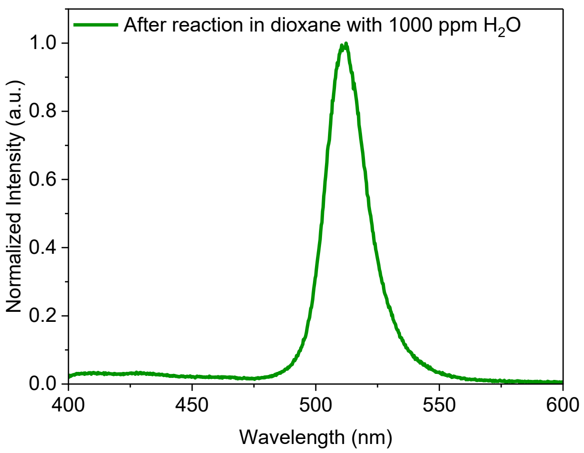
After reaction in dioxane with 1000 ppm H_2O
Normalized Intensity (a.u.)
Wavelength (nm)

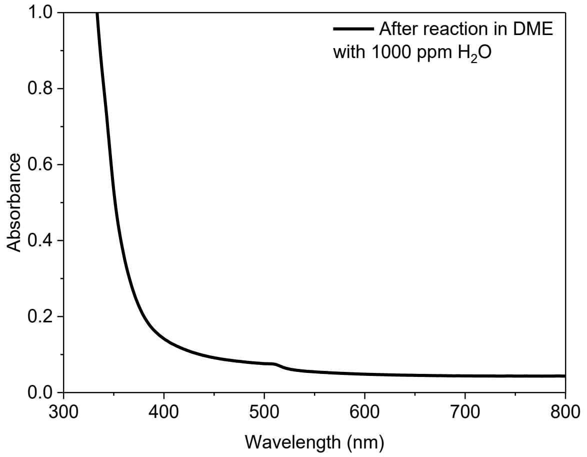
After reaction in DME
with 1000 ppm H_2O
Absorbance
Wavelength (nm)
0.0
0.2
0.4
0.6
0.8
1.0
300
400
500
600
700
800

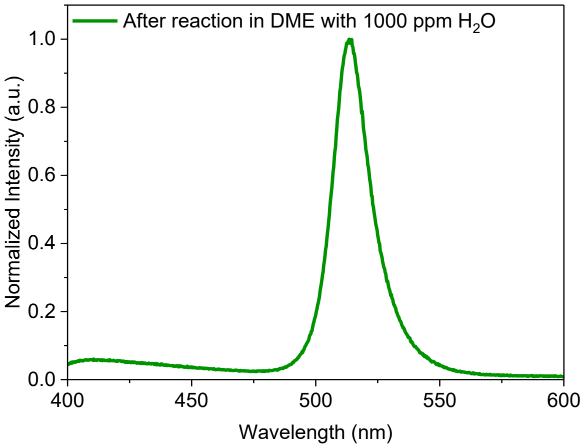
After reaction in DME with 1000 ppm H_2O
Normalized Intensity (a.u.)
Wavelength (nm)
0.0
0.2
0.4
0.6
0.8
1.0
400
450
500
550
600

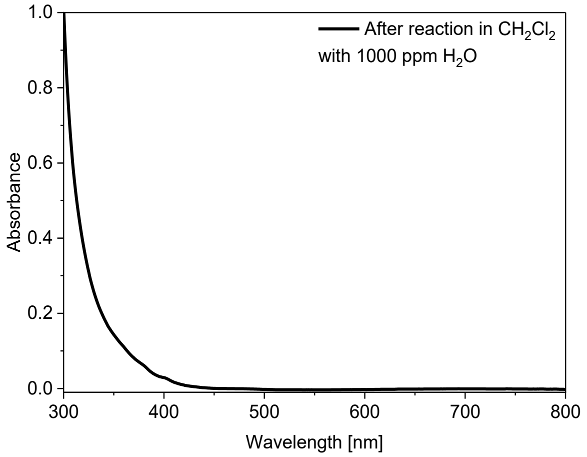
After reaction in CH_2Cl_2
with 1000 ppm H_2O
Absorbance
Wavelength [nm]
0.0
0.2
0.4
0.6
0.8
1.0
300
400
500
600
700
800

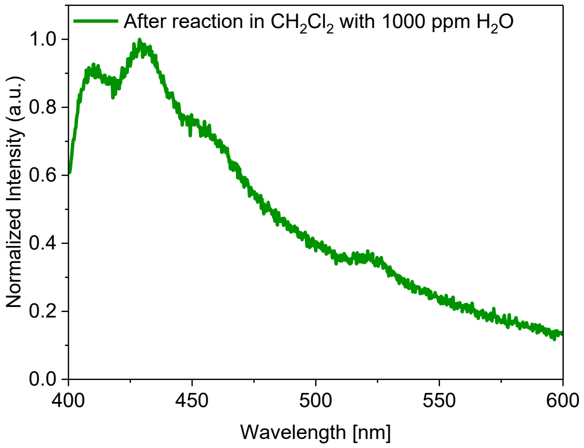
After reaction in CH_2Cl_2 with 1000 ppm H_2O
Normalized Intensity (a.u.)
Wavelength [nm]
0.0
0.2
0.4
0.6
0.8
1.0
400
450
500
550
600

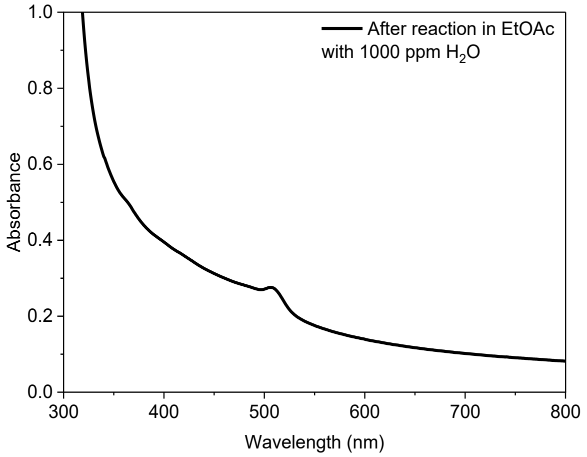
After reaction in EtOAc
with 1000 ppm H_2O
Absorbance
Wavelength (nm)
0.0
0.2
0.4
0.6
0.8
1.0
300
400
500
600
700
800

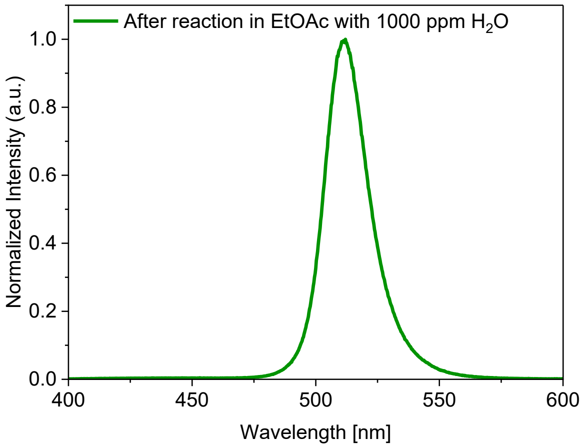
After reaction in EtOAc with 1000 ppm H_2O
Normalized Intensity (a.u.)
Wavelength [nm]
0.0
0.2
0.4
0.6
0.8
1.0
400
450
500
550
600

Table 6: Characteristic UV-Vis and PL signals for **ASC18-QDs** in the presence of 1000 ppm H_2O.

Solvent	Excitonic Peak from 2nd Derivative (nm)	PL Maxima (nm)	Yield
PhMe (Only Catalyst)	506	511	–
PhCl	516	518	76%
PhH	–	–	1%
PhMe	–	–	2%
$PhCF_3$	–	–	0%
Hexane	–	–	6%
CyH	517	519	67%
Et_2O	508	512	82%
MTBE	507	511	71%
THF	505	509	61%
Dioxane	510	512	32%
DME	510	513	87%
CH_2Cl_2	-	-	8%
EtOAc	510	511	85%

UV-Vis and PL spectra of the reaction in PhH, PhMe, $PhCF_3$, hexane, CH_2Cl_2, and $CHCl_3$ did not display characteristic absorption or fluorescence bands. We conclude that **ASC18-QDs** decomposed, which also explains the trace amounts of product. In $CHCl_3$ however, 49% yield of **2a** were observed despite catalyst decomposition. We hypothesize that in $CHCl_3$, **ASC18-QDs** decompose slow enough to allow for some catalyst turnover. When the UV-Vis spectrum of native **ASC18-QDs** in PhMe was compared with the reactions conducted in PhCl and CyH, we noticed a major bathochromic shift (+10 nm and +11 nm respectively). This shift translates to a significant growth of the $CsPbBr_3$ QD which was confirmed by comparable bathochromic shift in the photoluminescence spectrum.[5a] We hypothesize that water dissolved the **ASC18** ligand from the $CsPbBr_3$ surface, exposing the latter and

allowing growth of the quantum dot. Nonetheless, yields >50% were achieved in PhCl and CyH.

The UV-Vis absorption and photoluminescence spectra for Et_2O, MTBE, THF, dioxane, DME, and EtOAC, were remarkably close to native **ASC18-QDs**. The yields for 2a were also high (> 50% for all except dioxane). We concluded that **ASC18-QDs** retained their original size in these solvents despite the presence of 1.00 µL H_2O. This fact was likely beneficial for catalyst performance.

Next, we were interested how 2.00-5.00 µL H_2O in the reaction mixture affected the size of **ASC18-QDs** over the course of the reaction. We consequently measured UV-Vis and PL spectra of these reaction mixtures which are subsequently reported.

Reaction Conditions: 2000 ppm Water

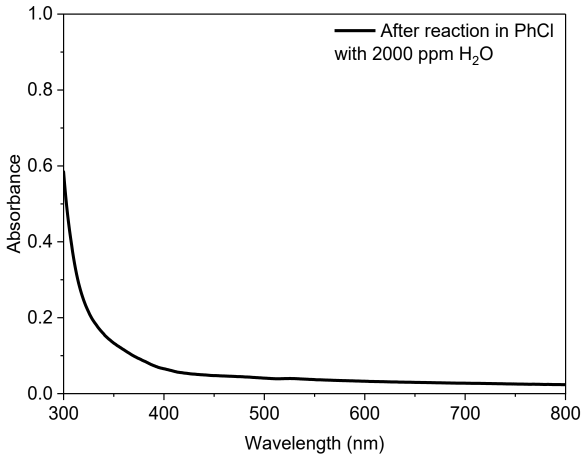

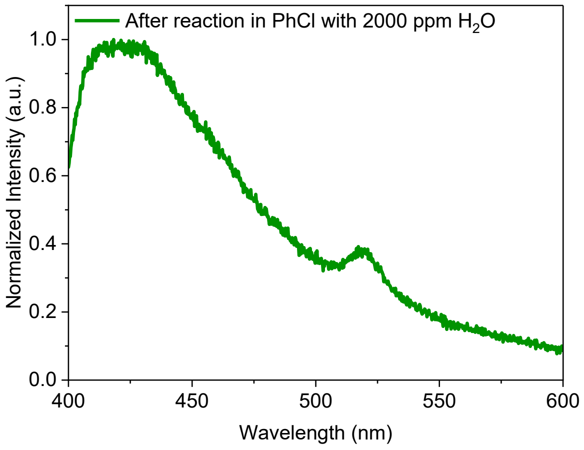

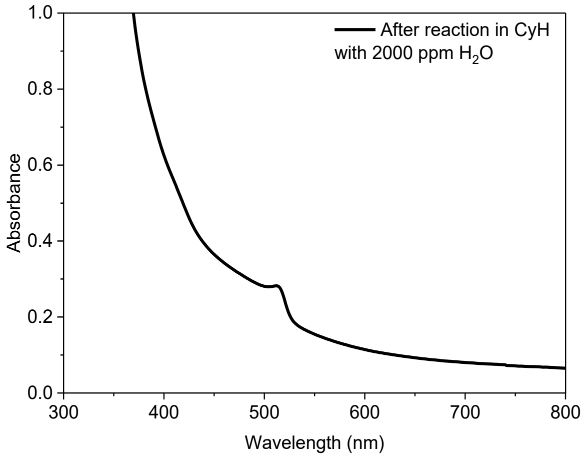

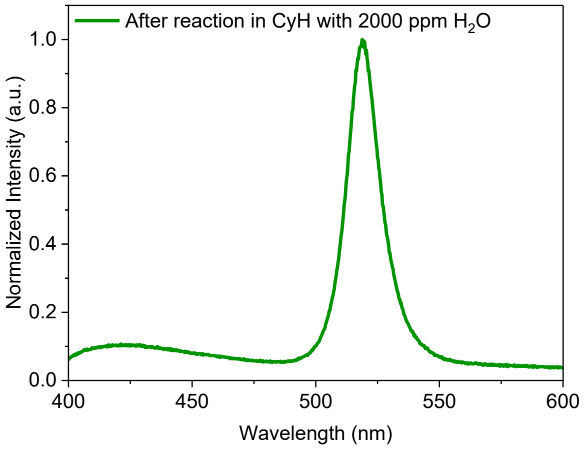

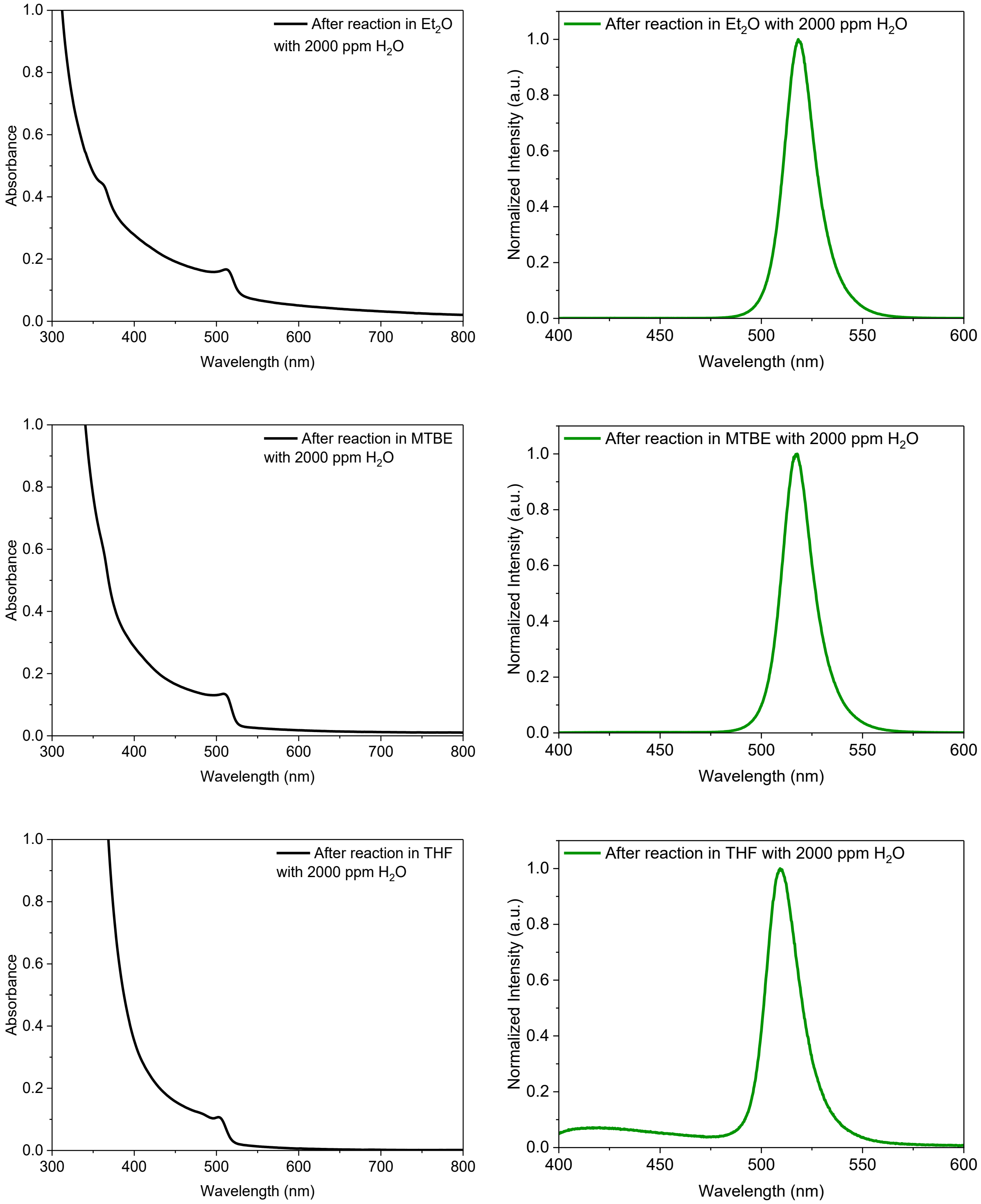
After reaction in Et_2O with 2000 ppm H_2O
Absorbance
Wavelength (nm)
After reaction in Et_2O with 2000 ppm H_2O
Normalized Intensity (a.u.)
Wavelength (nm)
After reaction in MTBE with 2000 ppm H_2O
Absorbance
Wavelength (nm)
After reaction in MTBE with 2000 ppm H_2O
Normalized Intensity (a.u.)
Wavelength (nm)
After reaction in THF with 2000 ppm H_2O
Absorbance
Wavelength (nm)
After reaction in THF with 2000 ppm H_2O
Normalized Intensity (a.u.)
Wavelength (nm)

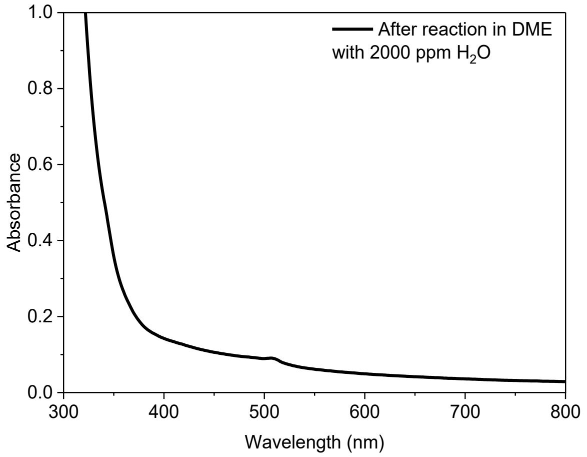

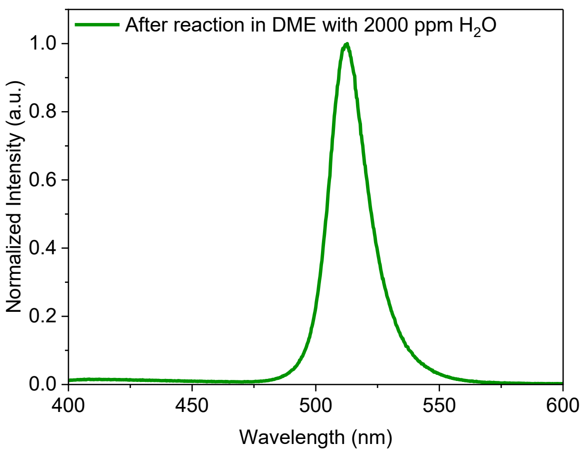

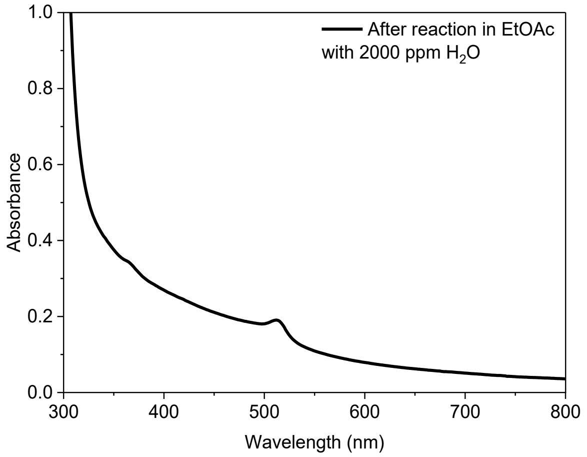

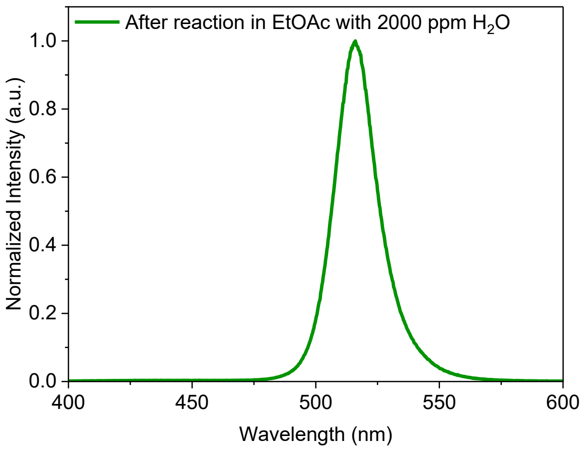

Table 7: Characteristic UV-Vis and PL signals for **ASC18-QDs** in the presence of 2000 ppm H_2O.

Solvent	Excitonic Peak from 2nd Derivative (nm)	PL Maxima (nm)	Yield
PhMe (Only Catalyst)	506	511	–
PhCl	–	–	2%
CyH	516	519	59%
Et_2O	515	518	58%
MTBE	514	518	66%
THF	506	510	62%
DME	509	513	77%
EtOAc	514	516	8%

While catalyst decomposition was observed in PhCl, significant growth of **ASC18-QDs** was observed in every other solvent except for THF and DME. However, the size of the LHP QDs and the yield did not seem to correlate. As an example, the yield in THF was comparable to Et_2O, despite the latter having a bathochromic shift of +9 nm while no shift was observed for the former.

Reaction Conditions: 3000 ppm Water

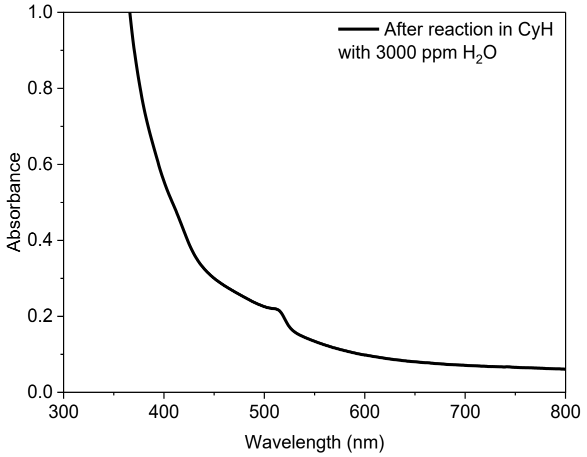

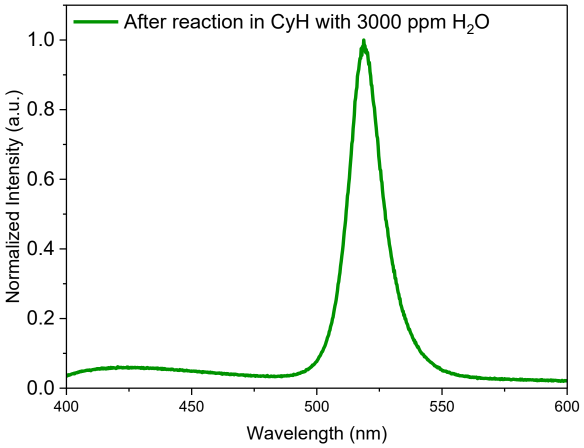

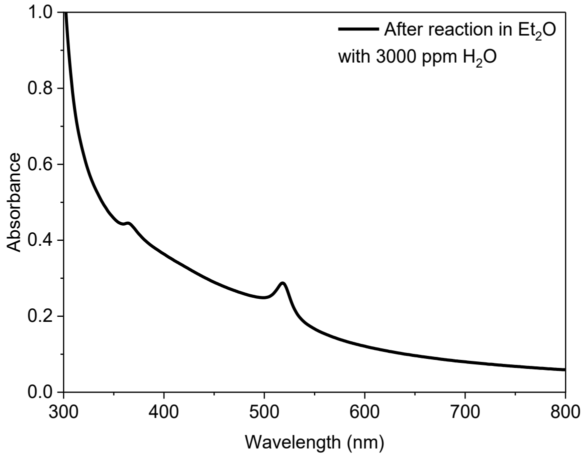

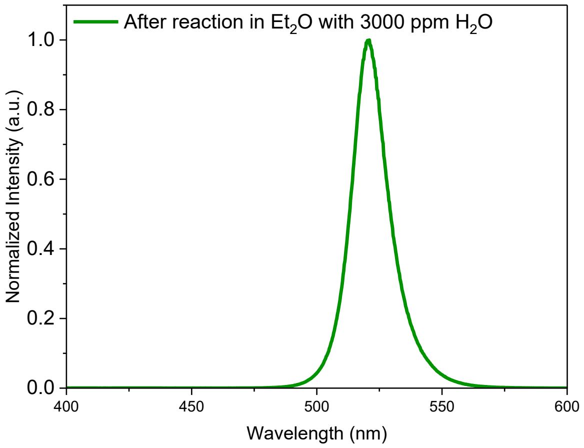

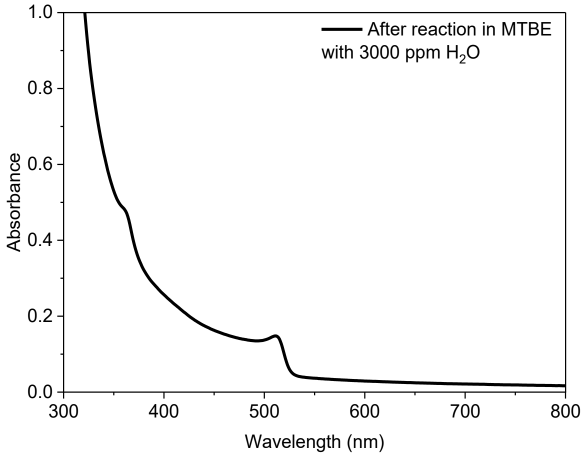
After reaction in MTBE with 3000 ppm H_2O
Absorbance
Wavelength (nm)

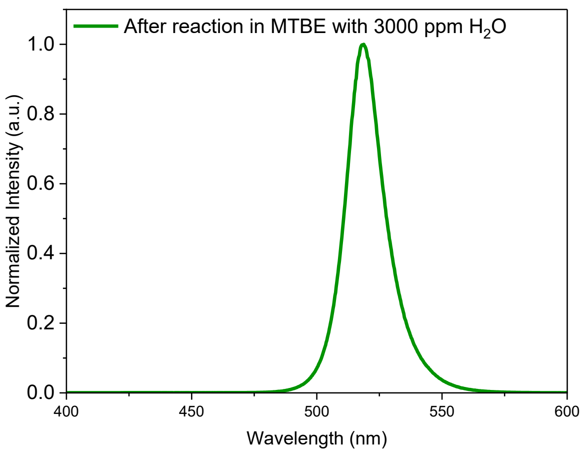
After reaction in MTBE with 3000 ppm H_2O
Normalized Intensity (a.u.)
Wavelength (nm)

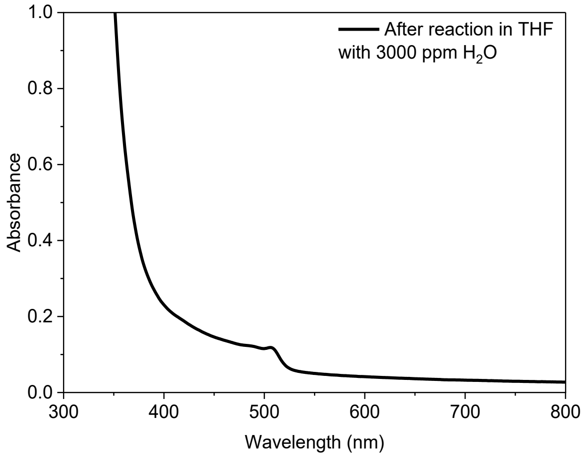
After reaction in THF with 3000 ppm H_2O
Absorbance
Wavelength (nm)

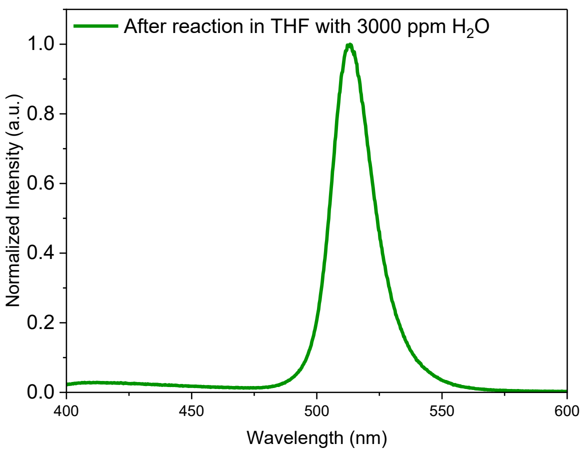
After reaction in THF with 3000 ppm H_2O
Normalized Intensity (a.u.)
Wavelength (nm)

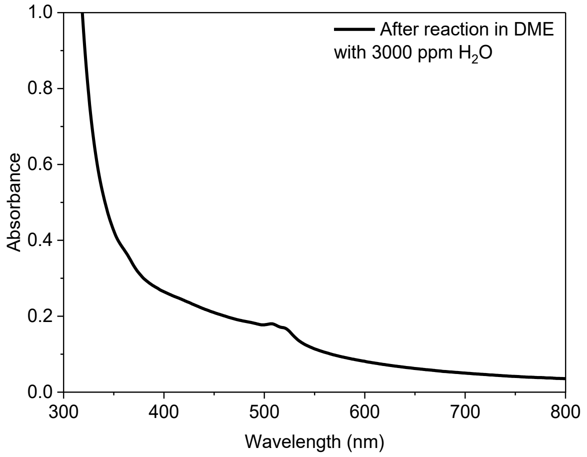
After reaction in DME with 3000 ppm H_2O
Absorbance
Wavelength (nm)

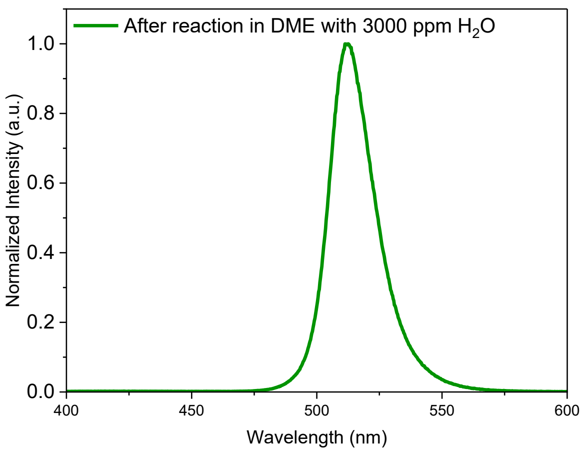
After reaction in DME with 3000 ppm H_2O
Normalized Intensity (a.u.)
Wavelength (nm)

Table 8: Characteristic UV-Vis and PL signals for **ASC18-QDs** in the presence of 3000 ppm H_2O.

Solvent	Excitonic Peak from 2nd Derivative (nm)	PL Maxima (nm)	Yield
PhMe (Only Catalyst)	506	511	–
CyH	516	519	48%
Et_2O	519	521	33%
MTBE	515	519	8%
THF	509	513	64%
DME	508	513	71%

In the presence of 3.00 µL H_2O, **ASC18-QDs** performed best in THF and DME in which the LHP QDs also exhibited the least amount of growth. It is noteworthy that even in the presence of 3.00 µL H_2O a yield of >50% can be achieved in some solvents.

Reaction Conditions: 4000 ppm Water

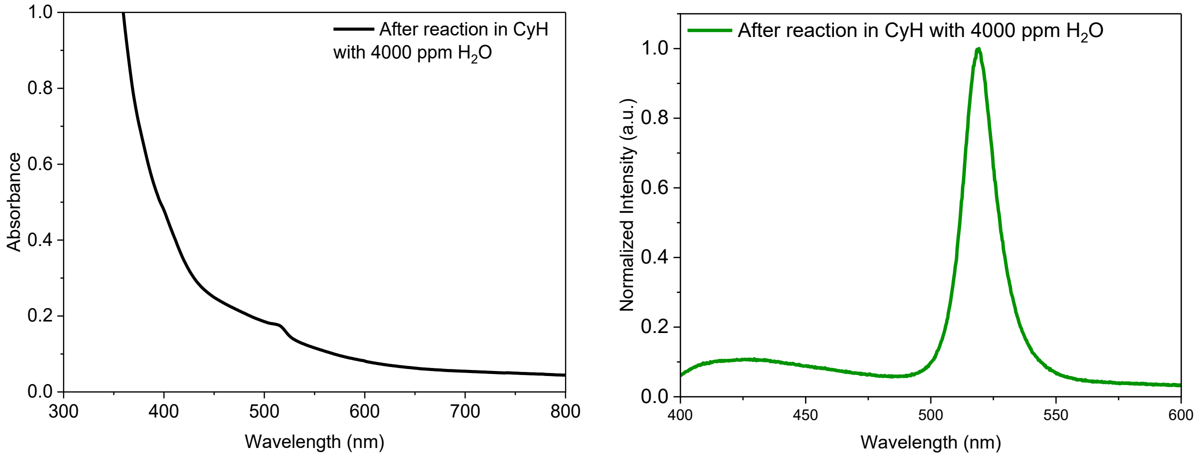

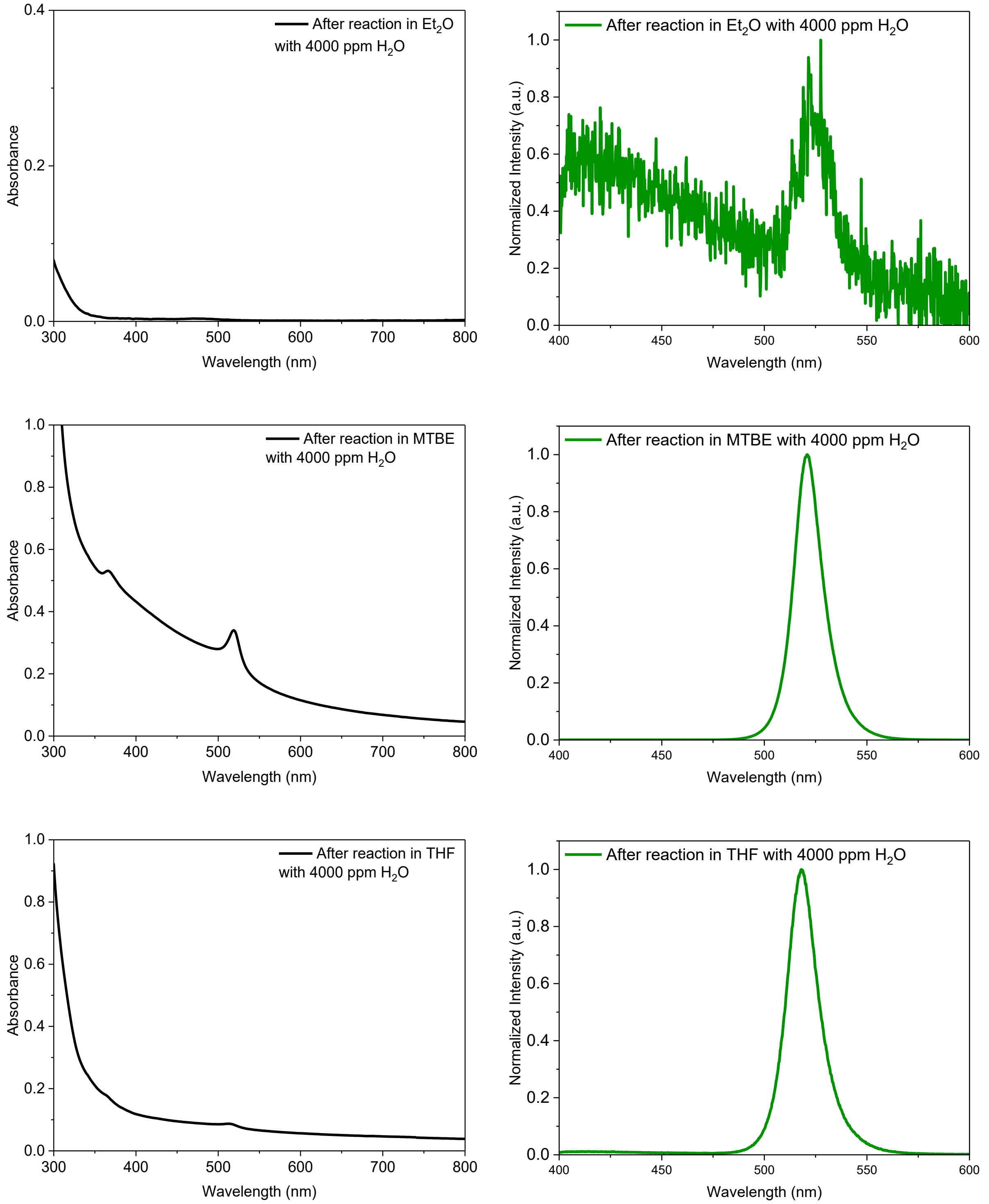
After reaction in Et_2O with 4000 ppm H_2O
Absorbance
Wavelength (nm)
After reaction in Et_2O with 4000 ppm H_2O
Normalized Intensity (a.u.)
Wavelength (nm)
After reaction in MTBE with 4000 ppm H_2O
Absorbance
Wavelength (nm)
After reaction in MTBE with 4000 ppm H_2O
Normalized Intensity (a.u.)
Wavelength (nm)
After reaction in THF with 4000 ppm H_2O
Absorbance
Wavelength (nm)
After reaction in THF with 4000 ppm H_2O
Normalized Intensity (a.u.)
Wavelength (nm)

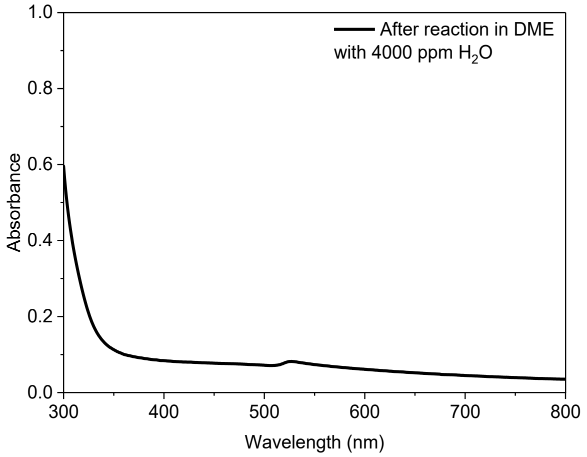

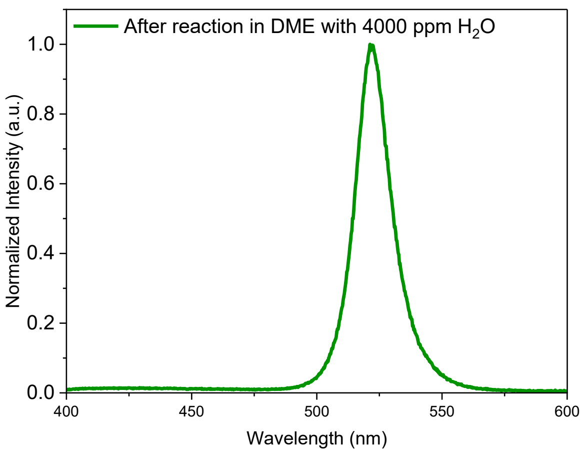

Table 9: Characteristic UV-Vis and PL signals for **ASC18-QDs** in the presence of 4000 ppm H_2O.

Solvent	Excitonic Peak from 2nd Derivative (nm)	PL Maxima (nm)	Yield
PhMe (Only Catalyst)	506	511	–
CyH	517	519	26%
Et_2O	–	–	9%
MTBE	521	521	8%
THF	516	518	10%
DME	525	522	29%

In the presence of 4.00 µL H_2O the yields for **2a** were below 50% in every investigated solvent. When 5.00 µL H_2O were added to the reaction mixture, no fluorescence or absorbance was measurable in any of the investigated solvents, indicating complete catalyst decomposition after the reaction time. This is consistent with low yields in the presence of 5.00 µL H_2O.

Table 10: Characteristic UV-Vis and PL signals for **ASC18-QDs** in the presence of 5000 ppm H_2O.

Solvent	Excitonic Peak from 2nd Derivative (nm)	PL Maxima (nm)	Yield
PhMe (Only Catalyst)	506	511	–
CyH	–	–	10%
Et_2O	–	–	2%
MTBE	–	–	9%
THF	–	–	3%
DME	–	–	7%

To ensure that the observed particle growth is owed to water and not a change in the dielectric constant of the solvent, we conducted additional UV-Vis and PL experiments. We have selected five solvents (CyH, PhH, PhMe, PhCl, THF) with varying dielectric constants (2.02, 2.27, 2.38, 5.62, 7.58 respectively), in which **ASC18**-capped QDs are colloidally stable. For CyH, PhH, PhMe and PhCl no shift in the characteristic UV-Vis or PL signals was observed (UV-Vis: 508 nm PL: 512 nm). For THF, a minor 1 nm hypsochromic shift was observed in the UV-Vis and PL spectrum. This shift can be attributed to THF coordination to the Pb on the QD surface, causing slight etching of the particle (see Figure 44). These results indicate that the observed bathochromic shift in the presence of water (up to 7 nm) cannot be explained by a small difference in dielectric constant but instead is a consequence of particle growth.

a

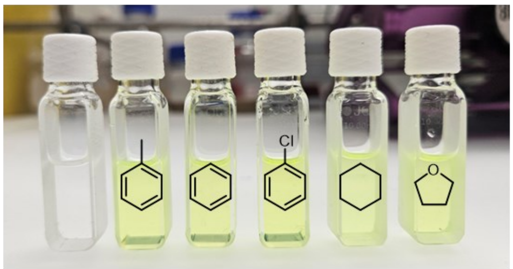

Solvent	Dielectric Constant	PL Maxima [nm]
PhMe	2.38	512
PhH	2.27	512
PhCl	5.62	512
CyH	2.02	512
THF	7.58	511

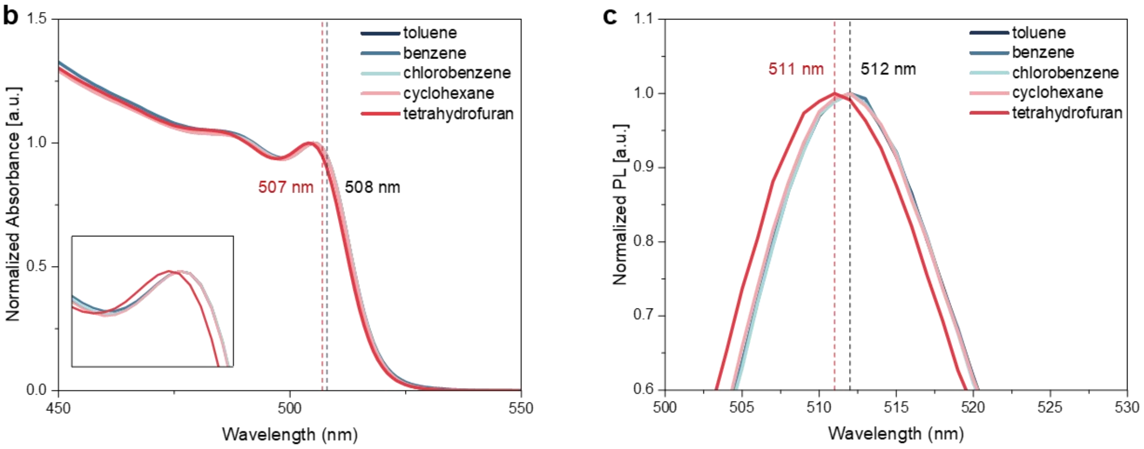

Figure 44: The solvent's dielectric constant effect on the excitonic absorption and PL peak position for **ASC18**-QDs. (a) Colloidally stable QDs in various solvents (b) UV-Vis and (c) Photoluminescence spectra of **ASC18-QDs** in different solvents.

11. Diffusion ordered spectroscopy (DOSY) experiments

The ligand dynamics of **ASC18-QDs**, **BRPE-QDs, DDAB-QDs, OGPE-QDs**, and **OGPC-QDs** were investigated with diffusion ordered spectroscopy experiments. For this measurement, fresh LHP QD samples were prepared according to the procedure described in chapter 5. However, in the last step, the final solvent for redispersing the particles was 0.500 mL d^{12}-cyclohexane.

For **BRPE-QDs** a signal overlap of the ligand tail and solvent (hexane) from the preparation of the particles was observed in the ^{1}H NMR spectrum. To obtain a sample of **BRPE-QDs** with no signal overlaps in the DOSY experiment, the procedure for the synthesis of **BRPE-QDs** was modified:

A glass vial equipped with a stir bar was charged with 4.00 mL hexane and 4.00 mL $PbBr_2$-TOPO (0.04 M stock solution, 0.160 mmol), followed by the rapid injection of 2.00 mL Cs-DOPA (0.02 M stock solution, 0.0400 mmol) under vigorous stirring. The particles were allowed to grow for 10 minutes at room temperature. After this time, 0.200 mL of the **BRPE** ligand solution (0.100 mg/µL in mesitylene) was added, and the mixture was stirred for one minute to ensure passivation of the QDs. The solution was subsequently split into two fractions (A and B) and 10.0 mL acetone were added to each fraction to precipitate the **BRPE-QDs**.

The precipitate of fraction A was dried under vacuum for one hour before the solid was redispersed in 0.500 mL cyclohexane-d^{12} for the DOSY measurements (non-washed sample).

The precipitate of fraction B was washed with 2 mL of a 1:1 mixture of cyclohexane:acetone and subsequently centrifuged at 12.1 krpm. The supernatant was subsequently discarded. This process was repeated two more times. The sample was subsequently dried under vacuum for one hour, and the solid was redispersed in 0.500 mL cyclohexane-d^{12} for the DOSY experiment (thrice-washed sample).

DOSY experiments were performed immediately after synthesizing the LHP QD sample using a double stimulated echo pulse sequence with a gradient up to 50 G/cm at a field of 11.7 T (γ_H = 500 MHz). The length of the gradient pulses (δ) and diffusion time (Δ) was set to 4 and 200 ms respectively. DOSY curves were constructed from 32 slices with linearly increasing gradient power (G_z) from 2 to 95%. The DOSY

measurements for **ASC18-QDs**, **DDAB-QDs**, and **BRPE-QDs** were conducted at 40 °C since the bromination reaction reaches 40 °C in the blue LED photoreactor. The curves were fitted to extract the diffusion coefficient (D) using either one or two exponentials depending on the nature of the ligand-surface interaction using the following equation:

$$\frac{I}{I_0} = e^{\left(-D(\gamma * G_z * \delta)^2\left(\Delta - \frac{\delta}{3}\right)\right)}$$

For all non-washed particles, a bi-exponential decay was observed. In case of the thrice washed particles, a bi-exponential decay was observed for **ASC18-QDs** and **DDAB-QDs**, while for **BRPE-QDs, OGPE-QDs**, and **OGPC-QDs** a mono-exponential decay was operative. The ratio between the fast (ligand) and slow (ligand attached to the LHP QD) component is defined as the ratio of free to bound ligand (bound ligand fraction, see manuscript for detailed discussion). Figure 45 shows the DOSY traces for the investigated LHP QDs as well as the bound ligand fraction.

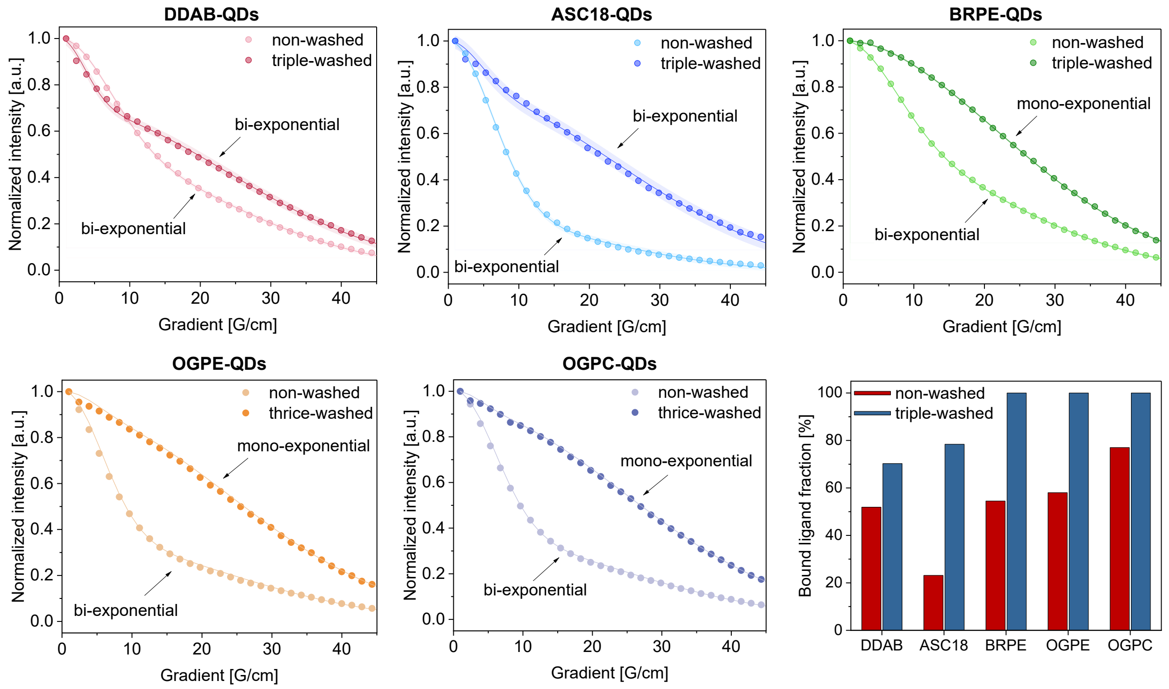

Figure 45: Investigation of ligand dynamics.

To substantiate that the washing steps do indeed remove excess ligand, we conducted additional ^{1}H NMR experiments. We measured ^{1}H NMR spectra of BRPE-QDs, DDAB-QDs, and ASC18-QDs directly after their synthesis (denoted as non-washed) and after being washed three times with their respective solvent/anti-solvent system. In the

NMR spectrum, broad peaks correspond to ligands that are a) bound to the QD surface b) located near the QD surface c) undergoing fast adsorption/desorption from the QD surface. In contrast, sharp peaks arise from free, excess ligands. In Figure 46, we focused mainly on the distinct -CH_3 signals of the alkyl chains in each ligand. Both broad and sharp peaks were present for non-washed samples, indicating a mixture of bound/unbound ligands. After three washing cycles with the respective solvent/anti-solvent pair, the sharp signals were significantly reduced, indicating the removal of excess ligands and synthesis-related impurities, while a large fraction of bound ligands remained.

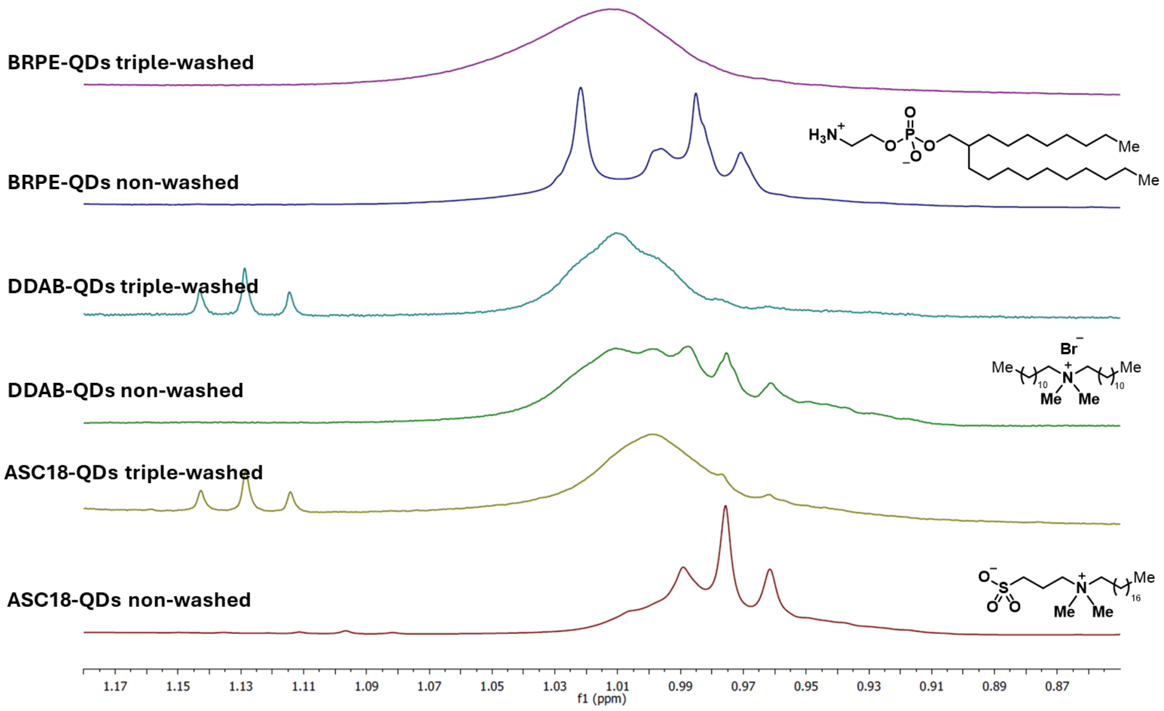

Figure 46: ^{1}H-NMR spectra of **ASC18-**, **DDAB-**, and **BRPE**-capped $CsPbBr_3$ QDs before and after washing. The broad peaks are associated with the ligand bound to the quantum dot surface while sharp ones indicate the free ligand. All spectra are zoomed at the region showing.

12. Stern-Volmer quenching studies

For the fluorescence studies, a Fluorolog iHR 320 Horiba Jobin Yvon spectrofluorometer equipped with a PMT detector was used to obtain steady-state photoluminescence (PL) spectra from glass cuvettes. In a typical experiment, 0.30 mg of the catalyst (**ASC18-QDs**) was added to 2 mL of benzene, which results in a catalyst concentration which is the same as for our transformation (0.15 mg/mL). The photoluminescence and absorption spectra of the blank catalyst solution were measured before the addition of each quencher (represented as 0.00 M in the plots). Subsequently, increasing amounts of selected quenchers were added sequentially to the same cuvette to reach the pre-determined concentrations (see table below).

Table 11: Amount of CH_2Br_2 and PhEt added to **ASC18-QDs** for quenching studies

Concentration [M]	CH_2Br_2 [µL]	PhEt (1a) [µL]
0.00	0.00	0.00
0.02	2.81	4.90
0.06	8.42	14.69
0.10	14.04	24.49
0.14	19.65	34.29
0.18	25.26	44.08
0.22	30.88	53.88
0.26	36.49	63.68

The change in volume was considered negligible. Consequently, a volume of 2.00 mL was used for the determination of each concentration. The UV-Vis/PL spectra of the new mixture were then measured immediately while excitation at 350 nm was used for photoluminescence measurements. The absorption spectra remained unchanged after the addition of selected quenchers, indicating steady state quenching results from the addition of charge transfer reagents rather than catalyst degradation (Figure 47)

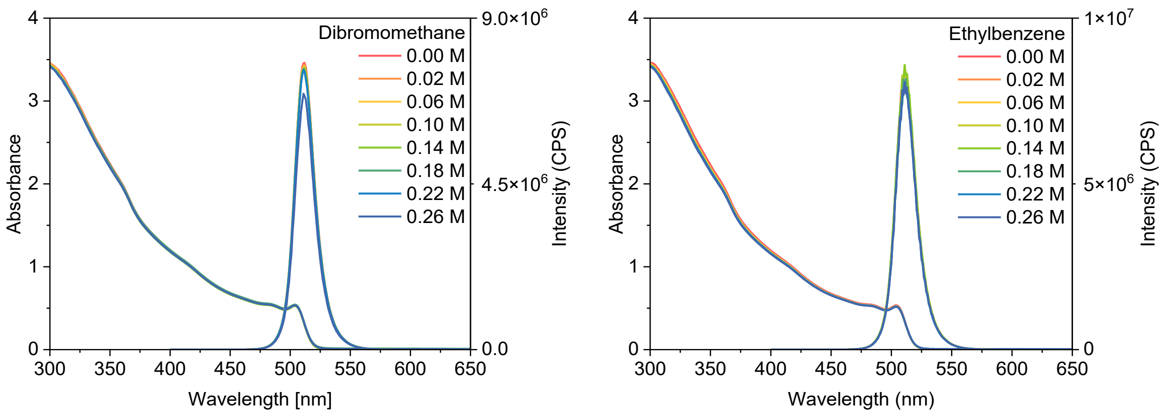

Figure 47: UV-Vis and PL spectra of **ASC18-QDs** at different CH_2Br_2 (left) and PhEt concentrations (right).

Stern-Volmer plots were generated using the integrated areas of photoluminescence spectra from 400 nm to 600 nm. The area of the blank catalyst solution (A_0) was divided by the area of each spectrum containing a quencher (A), and A_0/A was plotted against the concentration. Dibromomethane quenched the excited state photocatalyst while no quenching was observed with ethyl benzene.

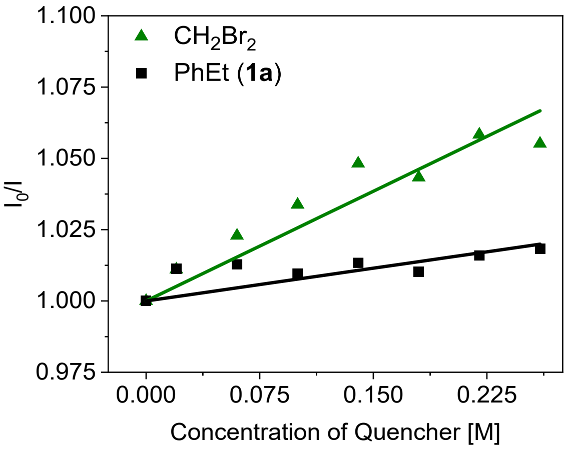

Figure 48: Stern-Volmer quenching plots for CH_2Br_2 and PhEt.

13. Further mechanistic studies

The benzylic bromination of ethyl benzene was chosen as model system for the mechanistic studies. As photocatalyst **ASC18-QDs** were employed as they performed best among the investigated quantum dots. The reaction order in substrate is determined from the initial rate of the reaction and the conversion of starting material was determined via ^1H NMR spectroscopy.

The bromination reaction was conducted according to general procedure 1 (*vide infra*) in a nitrogen filled glove box with slight modifications. As reaction solvent anhydrous C_6D_6 was chosen. C_6H_6 proved competent in our extensive solvent study for **ASC18-QDs** and we hypothesize that this would translate to C_6D_6. UV-Vis and fluorescence measurements of the reaction mixture in anhydrous C_6H_6 furthermore suggested that **ASC18-QDs** maintain their size over the course of the transformation. This ensured a well-defined catalyst throughout our mechanistic investigation.

Once the benzylic bromination was set up in the usual reaction vial, 0.800 mL of the reaction mixture were transferred into an oven dried NMR tube. The latter was subsequently placed into the blue LED photoreactor and irradiated for two minutes. Then, a ^1H NMR spectrum of the reaction was measured. This procedure was repeated four more times to gather five datapoints per reactant concentration and determine the initial rate.

To quantify the product formation after each irradiation cycle via ^1H NMR spectroscopy an internal standard was added to the reaction (for instructions on quantitative ^1H NMR spectroscopy, see Chapter 7. For the mechanistic studies described in this chapter, the interscan delay was set to 90.0 seconds). We refrained from using mesitylene as the compound contains benzylic positions which could be brominated during the transformation. Instead, 0.50 equiv of 1,4-dioxane were added. 1,4-Dioxane has a characteristic singlet in the ^1H NMR spectrum at 3.35 ppm. Our solvent study for **ASC18-QDs** furthermore showed that 1,4-dioxane is a viable solvent for the benzylic bromination of PhEt and is thus unlikely to obstruct product formation.

The reaction order of CH_2Br_2 was determined first. For these experiments, the **ASC18-QDs** ($9.88 \cdot 10^{-6}$ mol %) catalyzed benzylic bromination of PhEt (0.60 mmol) was set up in a reaction vial with 1.20 mmol (2.00 equiv.), 1.50 mmol (2.50 equiv.),

1.80 mmol (3.00 equiv.), 2.10 mmol (3.50 equiv.), and 2.40 mmol (4.00 equiv.) CH_2Br_2 respectively, 1.00 mL C_6D_6, and 1,4-dioxane (0.30 mmol) as internal standard. The sample was subsequently irradiated five times in 2-minute intervals. After each interval, a ^{1}H NMR spectrum was measured. Then, the initial rate was plotted against the respective concentration of CH_2Br_2 in the reaction, resulting in a calculated order of 0.71. We hypothesize that surface saturation of **ASC18-QDs** by CH_2Br_2 and thermodynamic equilibria in the adsorption of CH_2Br_2 could alter the reaction rate at varying CH_2Br_2 concentrations, resulting in a reaction order below one. The rate could further be limited by the diffusion rate of CH_2Br_2 to the surface of **ASC18-QDs**.

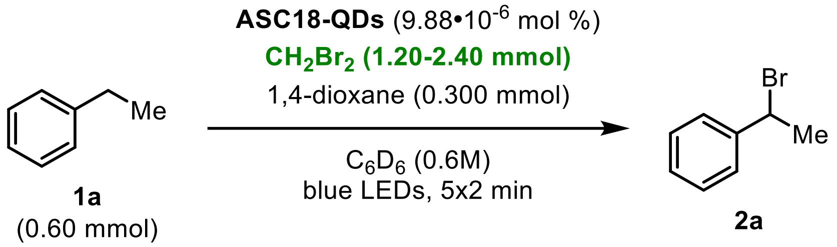

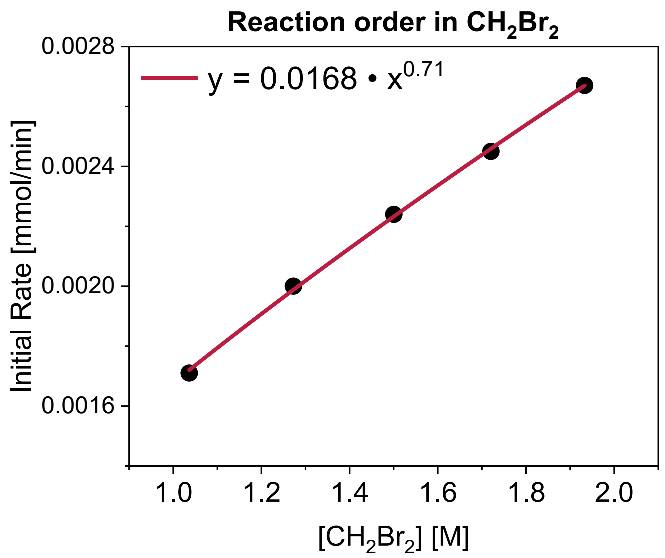

Figure 49: Reaction order for CH_2Br_2.

To support the hypothesis that increasing CH_2Br_2 concentration could decompose **ASC18-QDs**, we prepared two solutions: 1) **ASC18-QDs** in pure CH_2Br_2 and 2) **ASC18-QDs** in a 1:1 mixture of PhMe and CH2Br2. For each solvent mixture, two samples were prepared. One sample was stirred for 3 h under blue light irradiation and the second was stirred for 3 h in the dark. After this time, UV-Vis spectra were measured and compared to **ASC18-QDs** in toluene. UV-Vis absorption spectra will

provide more reliable evidence for QDs degradation, as they are not affected by quenching like PL.

Our studies indicate that in pure CH_2Br_2, QDs degrade via two distinct pathways: merging to larger particles and dissolution. First, we observed that the high concentration of CH_2Br_2 caused significant scattering and a pronounced red shift of the excitonic peak (7 nm), indicative of QD growth. Second, a reduction in absorption at shorter wavelengths (around 400 nm), which serves as a marker for the total volume of QDs in solution, clearly demonstrates that particle dissolution is occurring, even in the dark (Figure 50 b). Under reaction conditions (Figure 50 a), this reduction in absorption is partially masked by increased scattering, which introduces a monotonically decaying background in the absorption spectra. However, correcting for this background reveals a clear decrease in absorption at 400 nm, even in this case. Taken together, the UV-Vis absorption spectra of the four samples confirm the degradation of **ASC18-QDs** in CH_2Br_2.

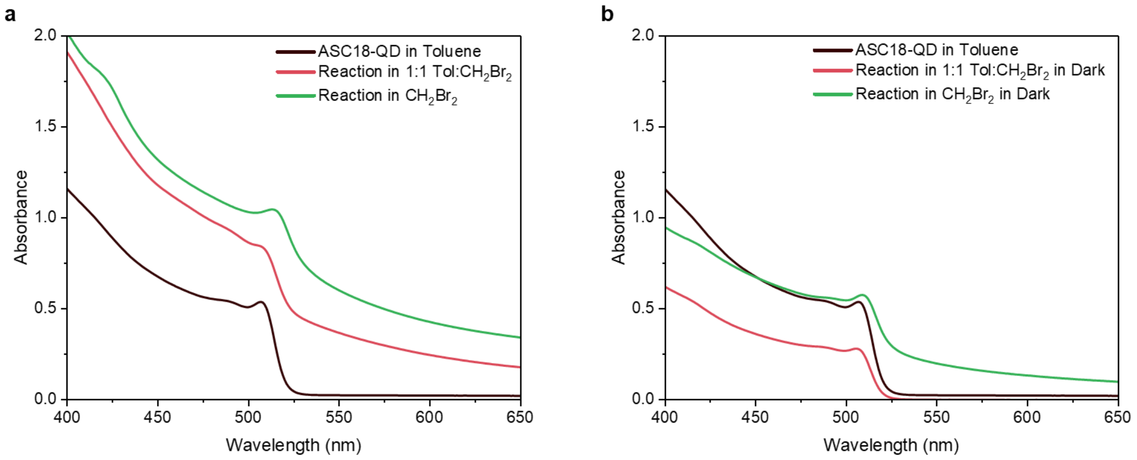

Figure 50: UV-Vis spectra of **ASC18-QDs** in CH_2Br_2, CH_2Br_2:PhMe (1:1), and PhMe with (a) and without (b) blue light irradiation.

The reaction order in PhEt was investigated next. To 1.00 mL C_6D_6 with 1,4-dioxane (0.300 mmol) as internal standard, was added 1.80 mmol CH_2Br_2, **ASC18-QDs** ($9.88 \cdot 10^{-6}$ mol %), and 0.300 mmol, 0.600 mmol, 0.900 mmol, 1.20 mmol, or 1.50 mmol PhEt, respectively. The reaction mixture was transferred into an NMR tube and irradiated five times for 2-minute intervals. Each initial rate was plotted against the

respective concentration in PhEt. The absence of a slope led us to the conclusion that the benzylic bromination proceeds in 0th order in PhEt.

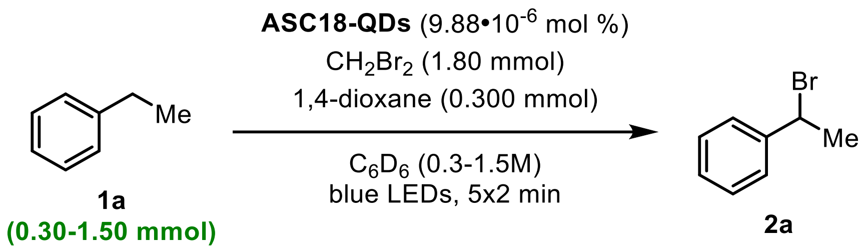

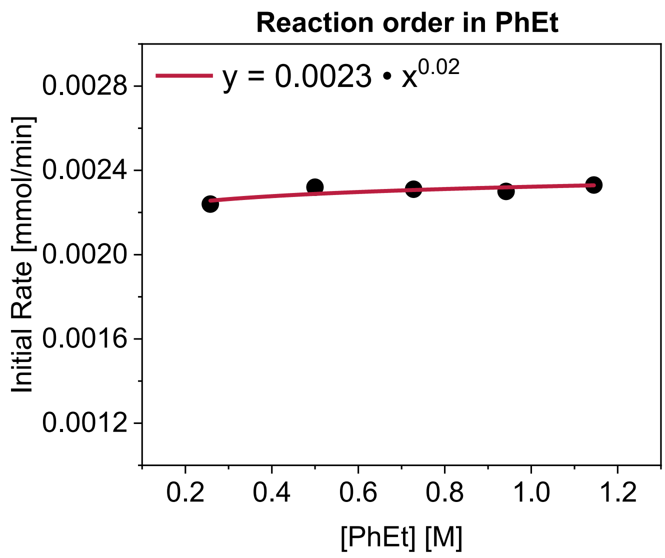

Figure 51: Reaction order for PhEt.

Lastly, the order in catalyst was determined. To 1.00 mL C_6D_6 with 1,4-dioxane (0.300 mmol) as internal standard was added PhEt (0.600 mmol), CH_2Br_2 (1.80 mmol) and 0.150 mg, 0.225 mg, 0.300 mg, 0.375 mg, or 0.450 mg **ASC18-QDs** ($9.88 \cdot 10^{-6}$ – $29.6 \cdot 10^{-6}$ mol %) respectively. The reaction mixture was transferred into an NMR tube and irradiated five times for 2-minute intervals. The initial rate for each catalyst loading was plotted against the amount of QD in mg. As shown, the reaction is 1st order in **ASC18-QDs**.

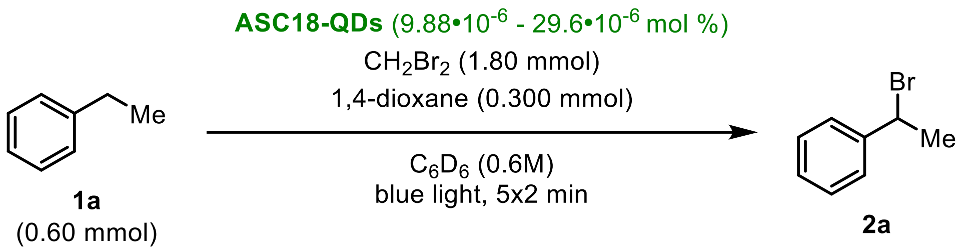

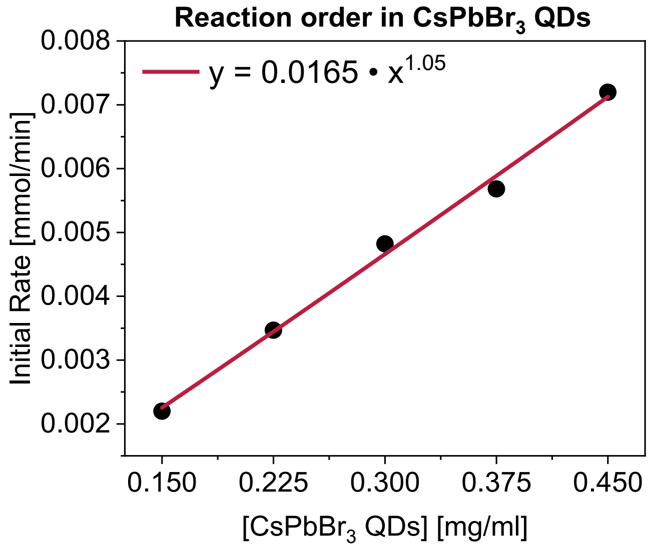

Figure 52: Reaction order for **ASC18-QDs**.

After we determined the reaction order in each reaction component, we found that CH_2Br_2 and the photocatalyst **ASC18-QDs** are 1st order and consequently both involved in the rate determining step.

To probe whether radicals are involved in the course of the reaction, we conducted two radical trapping experiments. First, the benzylic bromination of PhEt (0.60 mmol) was conducted in PhH with 3.00 equivalents CH_2Br_2, 9.88•10^{-6} mol % **ASC18-QDs** and 1.00 equivalent of TEMPO. After blue light irradiation for 3 h, no product formation was observed. However, upon addition of TEMPO to the reaction mixture, catalyst decomposition was observed which could also explain the lack of product formation.

To avoid a false negative result, we conducted the transformation with a different radical trapping agent, tosyl cyanide (TsCN).[8] Ethylbenzene (0.60 mmol), CH_2Br_2 (3.00 equiv.), **ASC18-QDs** (9.88•10^{-6} mol %) and TsCN (1.00 equiv.) were combined in a reaction vial with 1.00 mL PhH. After blue light irradiation for 3 h, we observed 3% of benzyl cyanide **S2**, 7% benzyl bromide **2a** and 90% starting material in the ^{1}H NMR spectrum of the unpurified reaction mixture. Furthermore, we were able to identify the

formation of bromoacetonitrile **S3** in 3% yield. **S2** and **S3** were identified by comparing the unpurified reaction mixture with the literature NMRs of **S2** and **S3**.[9] When we conducted the same reaction with three times the amount of **ASC18-QDs** (29.6•10^{-6} mol %), benzyl cyanide **S2** was obtained in 10% yield alongside 25% benzyl bromide **2a** and 58% starting material. Bromoacetonitrile **S3** was observed in 4% yield.

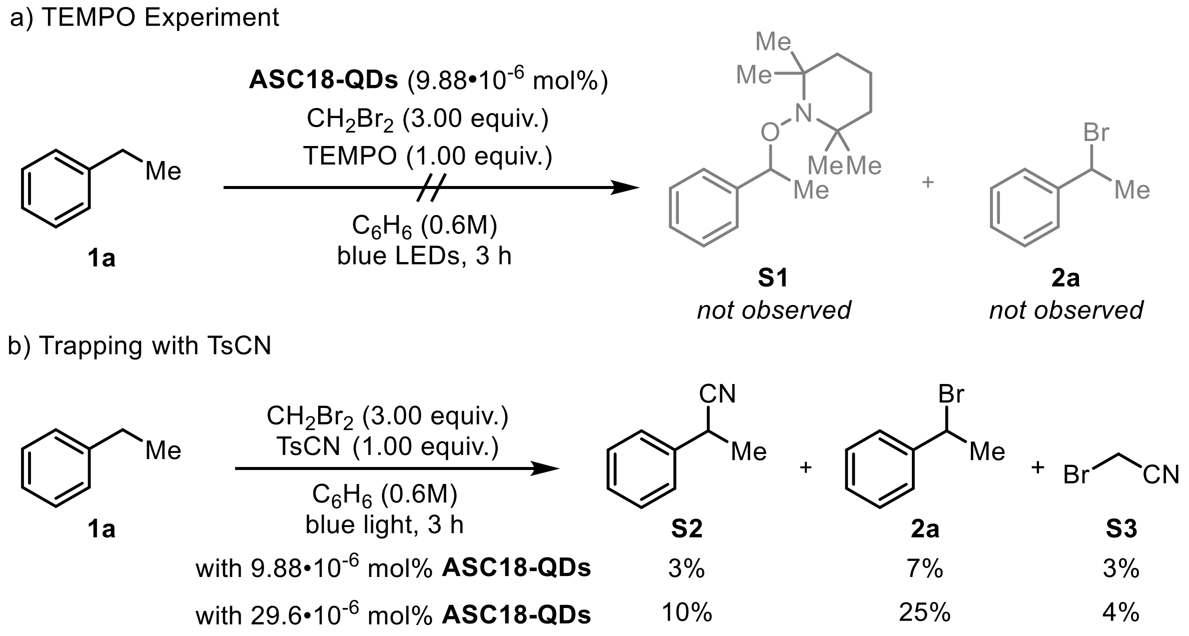

Figure 53: Radical trapping experiments.

The radical trapping studies confirmed two proposed species from our catalytic cycle. We suggest the formation of methyl bromide radical **7** after **ASC18-QDs** catalyzed C–Br bond reduction in CH_2Br_2. Trapping of this species by TsCN explains the formation of bromoacetonitrile. The observation of benzyl cyanide **S2** further substantiates the formation of a benzyl radical **9** throughout the reaction.

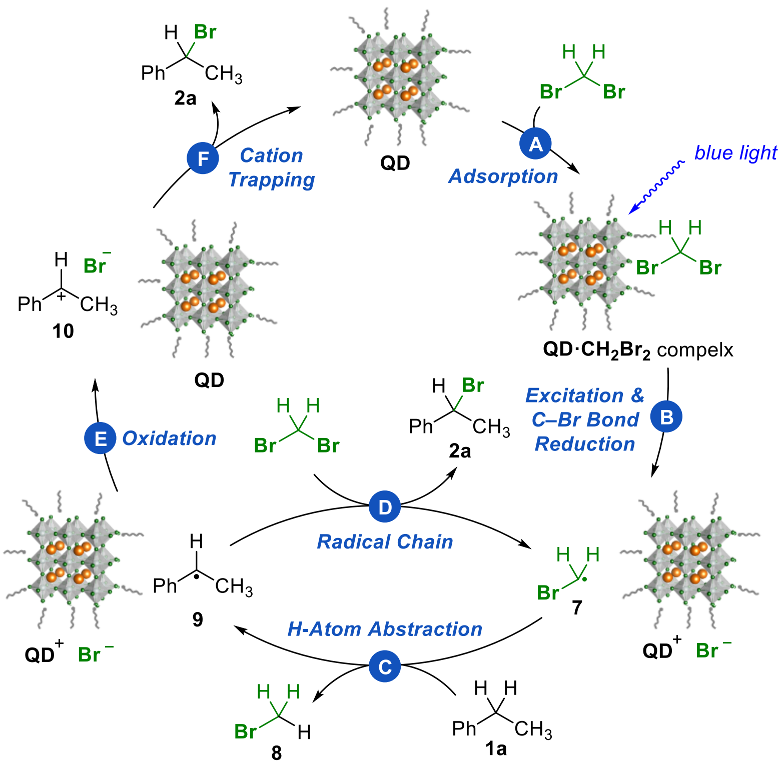

Kinetic isotope experiments were conducted next. For this study we conducted the benzylic bromination reaction with CD_2Br_2 instead of CH_2Br_2 and PhEt-d10 instead of PhEt, respectively. To 1.00 mL C_6D_6 with 1,4-dioxane (0.300 mmol) as internal standard were added 0.60 mmol PhEt, CD_2Br_2 (3.00 equiv.) and **ASC18-QDs** ($9.88 \cdot 10^{-6}$ mol %). The reaction mixture was transferred into an NMR tube and irradiated five times for 2-minute intervals. For CD_2Br_2 we determined a kinetic isotope effect of 1.8. This value is in agreement with a secondary KIE in which the deuterated substrate undergoes planarization during the reaction and hints towards an intermediary formation of either CD_2Br^+ or $CD_2Br^{\bullet}$ which are both planar compounds.[10] Furthermore, we identified CD_2HBr in the ^1H NMR spectrum of the unpurified reaction mixture. This observation is in agreement with our mechanistic construct in which the methyl bromide radical **7** abstracts the benzylic H-atom. Lastly, we identified α-deuterated PhEt **1a-d1** in the reaction mixture. This additional observation hints toward an additional pathway in our reaction mechanism. We suggested that upon formation of the benzyl radical, it can either abstract a bromide from CH_2Br_2 or undergo oxidation to the benzyl cation. With this new observation from the KIE study in hand, we propose a third pathway for benzyl radical **9**: Upon formation it can abstract an H-atom from CH_2Br_2 to regenerate ethyl benzene.

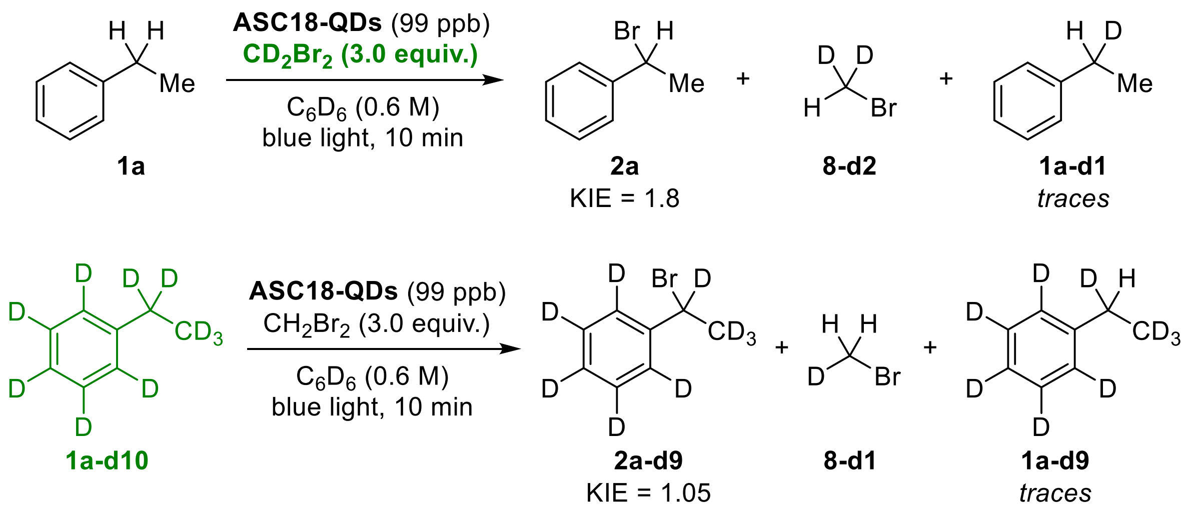

Figure 54: KIE experiments.

Fully deuterated PhEt (**1a-d10**) was investigated for a kinetic isotope effect next. To 1.00 mL C_6D_6 with 1,4-dioxane (0.30 mmol) as internal standard were added 0.60 mmol **1a-d10**, CH_2Br_2 (3.00 equiv.) and **ASC18-QDs** ($9.88 \cdot 10^{-6}$ mol %). The reaction mixture was transferred into an NMR tube and irradiated five times for 2-minute intervals. After analysis of the ^1H NMR spectrum of the unpurified reaction mixture we determined a kinetic isotope effect of 1.05 for **1a-d10**. This value signifies that neither a primary nor a secondary KIE is operational for d10-PhEt. This observation is in line with our reaction order study, which suggested 0th order in PhEt and implies that ethyl benzene is not involved in the rate determining step and can thus not exhibit a KIE. We also identified CH_2DBr alongside **1a-d9** in the ^1H NMR spectrum of the unpurified reaction mixture. Like **1a-d1**, the formation of **1a-d9** suggests a third reaction pathway for benzyl radical **9**, namely the H• abstraction from CH_2Br_2 to return to PhEt **1a**.

The reaction quantum yield was determined according to the literature.[2] The photon flux for our reaction set-up has previously been calculated to be 9.12×10^{-7} Einstein s^{-1}.[3] For the benzylic bromination of **1a** we determined a reaction quantum yield of $\Phi = 0.011$, while for **5a** we measured $\Phi = 0.014$.

To control for the ability of organic dyes to effect the desired bromination reaction, several control experiments were conducted:

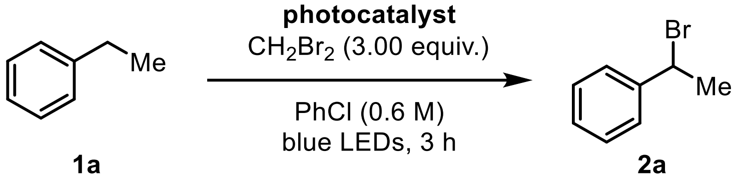

Entry	Photocatalyst	[mol%]	yield [%]
1	*N*,*N*,*N*,*N*-tetramethylbenzene-1,4-diamine	1.00	<1
2	$Ir(ppy)_3$	1.00	<1
3	12-phenyl-12*H*-benzo[5,6][1,4]thiazino [2,3-b]quinoxaline	1.00	<1
4	$Ru(bpy)_3Cl_2$	1.00	<1
5	4CzIPN	1.00	<1
6	EosinY	1.00	<1
7	*N*,*N*,*N*,*N*-tetramethylbenzene-1,4-diamine	0.50	<1
8	$Ir(ppy)_3$	0.50	<1
9	12-phenyl-12*H*-benzo[5,6][1,4]thiazino [2,3-b]quinoxaline	0.50	<1
10	$Ru(bpy)_3Cl_2$	0.50	<1
11	4CzIPN	0.50	<1
12	EosinY	0.50	<1
13	*N*,*N*,*N*,*N*-tetramethylbenzene-1,4-diamine	0.10	<1
14	$Ir(ppy)_3$	0.10	<1
15	12-phenyl-12*H*-benzo[5,6][1,4]thiazino [2,3-b]quinoxaline	0.10	<1
16	$Ru(bpy)_3Cl_2$	0.10	<1
17	4CzIPN	0.10	<1
18	EosinY	0.10	<1
19	*N*,*N*,*N*,*N*-tetramethylbenzene-1,4-diamine	0.01	<1
20	$Ir(ppy)_3$	0.01	<1
21	12-phenyl-12*H*-benzo[5,6][1,4]thiazino [2,3-b]quinoxaline	0.01	<1
22	$Ru(bpy)_3Cl_2$	0.01	<1
23	4CzIPN	0.01	<1
24	EosinY	0.01	<1

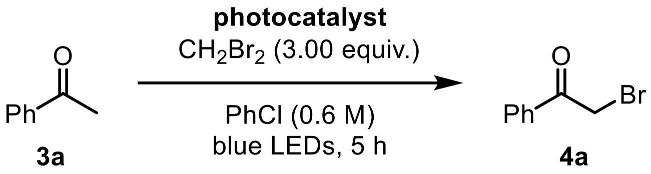

Entry	Photocatalyst	[mol%]	yield [%]
1	*N*,*N*,*N*,*N*-tetramethylbenzene-1,4-diamine	1.00	<1
2	Ir(ppy)$_3$	1.00	<1
3	12-phenyl-12*H*-benzo[5,6][1,4]thiazino [2,3-b]quinoxaline	1.00	<1
4	Ru(bpy)$_3$Cl$_2$	1.00	<1
5	4CzIPN	1.00	<1
6	EosinY	1.00	<1

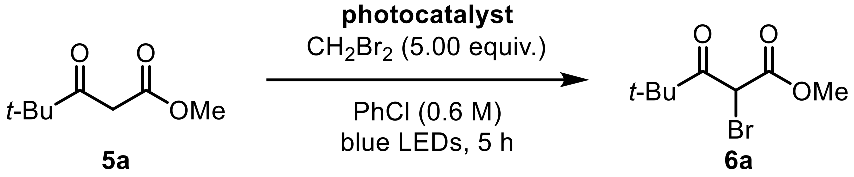

Entry	Photocatalyst	[mol%]	yield [%]
1	*N*,*N*,*N*,*N*-tetramethylbenzene-1,4-diamine	1.00	<1
2	Ir(ppy)$_3$	1.00	<1
3	12-phenyl-12*H*-benzo[5,6][1,4]thiazino [2,3-b]quinoxaline	1.00	<1
4	Ru(bpy)$_3$Cl$_2$	1.00	<1
5	4CzIPN	1.00	<1
6	EosinY	1.00	<1

14. General procedures for C–H bromination

GP1: Benzylic bromination

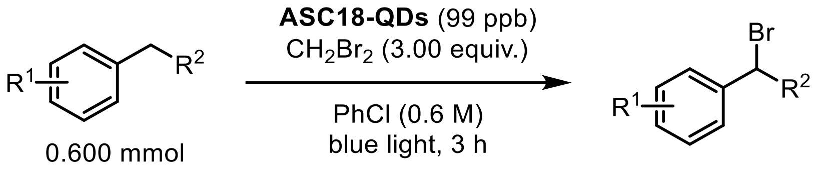

All reactions were conducted in a nitrogen filled glove box with anhydrous PhCl and anhydrous CH_2Br_2. A glass vial was sequentially charged with PhCl (1.00 mL), CH_2Br_2 (125 µL, 311 mg 1.80 mmol, 3.00 equiv.) starting material (0.600 mmol, 1.00 equiv.), and 3.35 µL **ASC18-QDs** stock solution (44.77 mg/mL, 0.15 mg, corresponds to $9.88 \cdot 10^{-6}$ mol %). The vial was equipped with a magnetic stir bar and capped with a screwcap. The reaction was irradiated in a 350 W photoreactor (*vide supra*) for 3 h unless otherwise stated. The crude reaction mixture was purified using silica gel chromatography with the appropriate eluent to obtain pure product.

GP2: Bromination of ketones

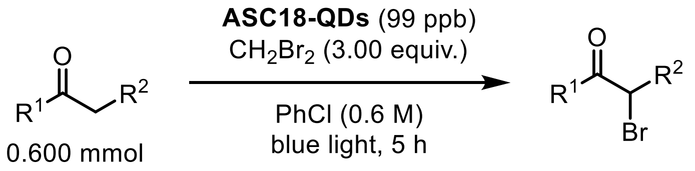

All reactions were conducted in a nitrogen filled glove box with anhydrous PhCl and anhydrous CH_2Br_2. A glass vial was sequentially charged with PhCl (1.00 mL), CH_2Br_2 (125 µL, 311 mg, 1.80 mmol, 3.00 equiv.), ketone starting material (0.600 mmol, 1.00 equiv.), and 3.35 µL **ASC18-QDs** stock solution (44.77 mg/mL, 0.15 mg, corresponds to $9.88 \cdot 10^{-6}$ mol %). The vial was equipped with a magnetic stir bar and capped with a screwcap. The reaction was irradiated in a 350 W photoreactor (*vide supra*) for 5 h unless otherwise stated. The crude reaction mixture was purified using silica gel chromatography with the appropriate eluent to obtain pure product.

GP3: Bromination of β-Ketoesters

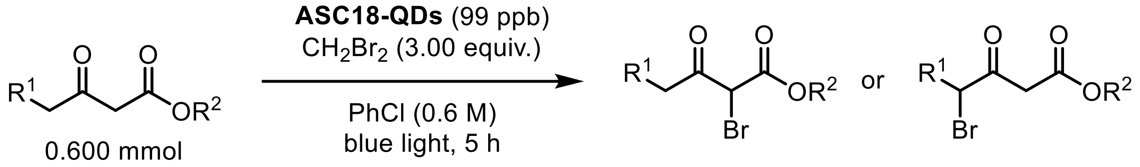

All reactions were conducted in a nitrogen filled glove box with anhydrous PhCl and anhydrous CH_2Br_2. A glass vial was sequentially charged with PhCl (1.00 mL), CH_2Br_2 (125 µL, 1.80 mmol, 3.00 equiv.), β-ketoester starting material (0.600 mmol, 1.00 equiv.), and 3.35 µL **ASC18-QDs** stock solution (44.77 mg/mL, 0.15 mg, corresponds to $9.88 \cdot 10^{-6}$ mol %). The vial was equipped with a magnetic stir bar and capped with a screwcap. The reaction was irradiated in a 350 W photoreactor (*vide supra*) for 5 h unless otherwise stated. The crude reaction mixture was purified using silica gel chromatography with the appropriate eluent to obtain pure product.

15. Substrate scope

Compound 2a:

(1-Bromoethyl)benzene

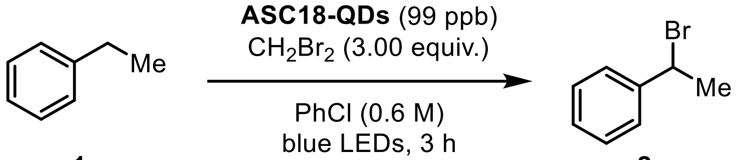

1a **2a**

Bromide **2a** was prepared via **GP1** from compound **1a** (73.5 µL, 63.7 mg, 0.600 mmol, 1.00 equiv) in PhCl. Due to the volatility of the product, **2a** was not isolated and the yield was determined via ^{1}H NMR spectroscopy with mesitylene as internal standard. Bromide **2a** is a known compound and the ^{1}H NMR spectrum of the unpurified reaction mixture matched with the literature.[11]

Note: An analytically pure sample was obtained via flash column chromatography (100% pentane). Evaporation of the solvent had to be conducted slowly at 30 °C and 750 mbar.

Yield: 84% (NMR)

Yield (10 mmol scale): 75% (NMR)

^{1}H NMR (400 MHz, $CDCl_3$): δ (ppm) = 7.49 – 7.41 (m, 2H), 7.39 – 7.26 (m, 3H), 5.23 (q, J = 6.9 Hz, 1H), 2.06 (d, J = 6.9 Hz, 3H).

^{13}C NMR (101 MHz, $CDCl_3$): δ (ppm) = 143.4, 128.8, 128.5, 126.9, 49.7, 27.0.

IR (thin film, cm^{-1}): 2989, 1454, 1212, 1179, 1044, 1026, 964, 761, 693, 625, 590, 562.

HRMS (EI+): *m/z* for C_8H_9 [M–Br]$^+$: calc.: 105.0699, found: 105.0696

Compound 2b:

1-(1-Bromoethyl)-4-chlorobenzene

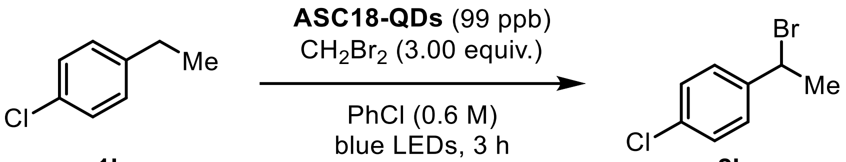

1b **2b**

Bromide **2b** was prepared via **GP1** from compound **1b** (80.7 µL, 84.4 mg, 0.600 mmol, 1.00 equiv) in PhCl. Due to the volatility of the product, **2b** was not isolated and the yield was determined via ^{1}H NMR spectroscopy with mesitylene as internal standard. Bromide **2b** is a known compound and the ^{1}H NMR spectrum of the unpurified reaction mixture matched with the literature.[12]

Note: When the reaction was conducted under ambient conditions (under air, wet solvent), the desired product was formed in 85% yield.

Yield: 99% (NMR)

Compound 2c:

4-(1-Bromoethyl)-1,1'-biphenyl

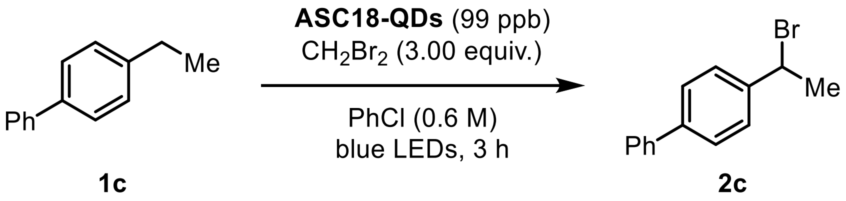

Bromide **2c** was prepared via **GP1** from compound **1c** (109 mg, 0.600 mmol, 1.00 equiv) in PhCl. Due to decomposition upon isolation, the yield of **2c** was determined via ^{1}H NMR spectroscopy with mesitylene as internal standard. Bromide **2c** is a known compound and the ^{1}H NMR spectrum of the unpurified reaction mixture matched with the literature.[13]

Note: When the reaction was conducted under ambient conditions (under air, wet solvent), the desired product was formed in 67% yield.

Yield: 73% (NMR)

Compound 2d:

1-(1-Bromoethyl)-4-fluorobenzene

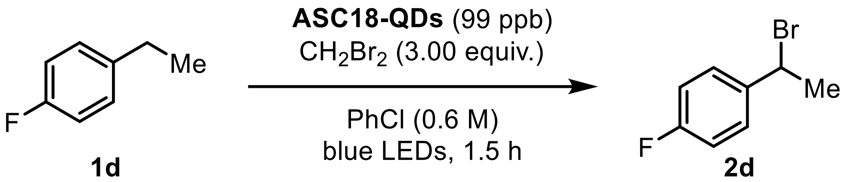

Bromide **2d** was prepared via **GP1** from compound **1d** (74.5 µL, 74.5 mg, 0.600 mmol, 1.00 equiv) in PhCl. Due to the volatility of the product, **2d** was not isolated and the yield was determined via ^{1}H NMR spectroscopy with mesitylene as internal standard. Bromide **2d** is a known compound and the ^{1}H NMR spectrum of the unpurified reaction mixture matched with the literature.[14]

Note: The reaction was stirred for 1.5 h instead of 3 h in the blue LED photoreactor.

Yield: 72% (NMR)

Compound 2e:

1-(1-bromoethyl)-3-(*tert*-butyl)benzene

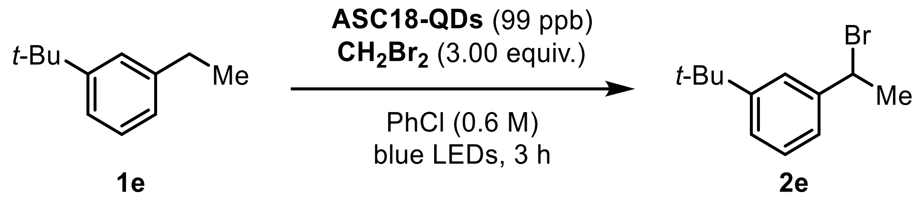

Bromide **2e** was prepared via **GP1** from compound **1e** (113 µL, 97.4 mg, 0.600 mmol, 1.00 equiv) in PhCl. Due to the volatility of the product, **2e** was not isolated and the yield was determined via ^{1}H NMR spectroscopy with mesitylene as internal standard. Bromide **2e** is a known compound and the ^{1}H NMR spectrum of the unpurified reaction mixture matched with the literature.[14]

Yield: 80% (NMR)

Compound 2f:

1-(1-Bromobutyl)-4-chlorobenzene

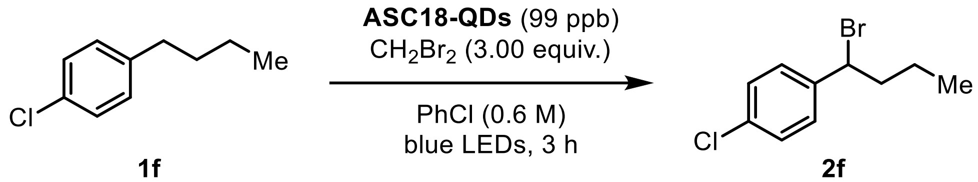

Bromide **2f** was prepared via **GP1** from compound **1f** (101 mg, 0.600 mmol, 1.00 equiv) in PhCl. The crude product was purified via flash column chromatography (100% pentane) to give **2f** as a colorless oil.

Note: Due to potential volatility of the product, the solvent was evaporated at 750 mbar at 35 °C.

Yield: 120 mg, 0.484 mmol, 81%

^{1}H NMR (400 MHz, $CDCl_3$): δ (ppm) = 7.37 – 7.28 (m, 4H), 4.93 (t, *J* = 7.0 Hz, 1H), 2.25 (dddd, *J* = 14.2, 9.7, 8.1, 5.4 Hz, 1H), 2.07 (dddd, *J* = 14.2, 9.6, 7.0, 5.7 Hz, 1H), 1.57 – 1.42 (m, 1H), 1.40 – 1.26 (m, 1H), 0.94 (t, *J* = 7.4 Hz, 3H).

^{13}C NMR (101 MHz, $CDCl_3$): δ (ppm) = 141.0, 134.1, 129.0, 128.8, 54.2, 42.1, 21.5, 13.4.

IR (thin film, cm^{-1}): 2960, 1491, 1465, 1410, 1173, 1090, 1014, 826, 790, 757, 748, 724, 678, 661, 625, 583, 529.

HRMS (EI): *m/z* for $C_{10}H_{12}Cl$ $[M–Br]^+$: calc.: 167.0622, found: 167.0624

Compound 2g:

Methyl 4-(1-bromoethyl)benzoate

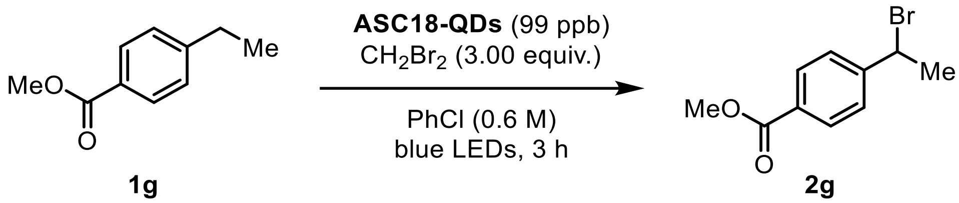

Bromide **2g** was prepared via **GP1** from compound **1g** (98.5 mg, 0.600 mmol, 1.00 equiv) in PhCl. Due to the volatility of the product, **2g** was not isolated and the yield was determined via ^{1}H NMR spectroscopy with mesitylene as internal standard. Bromide **2g** is a known compound and the ^{1}H NMR spectrum of the unpurified reaction mixture matched with the literature.[15]

Yield: 81% (NMR)

Compound 2h:

(Bromomethylene)dibenzene

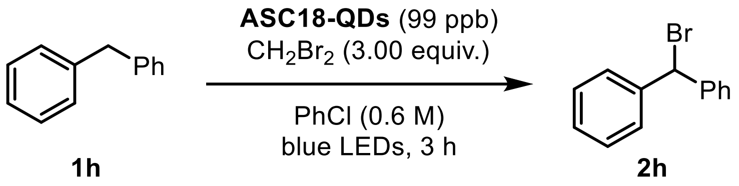

Bromide **2h** was prepared via **GP1** from compound **1h** (100 µL, 101 mg, 0.600 mmol, 1.00 equiv) in PhCl. Compound **2h** was susceptible to hydrolysis. Thus, the yield of **2h** was determined via ^{1}H NMR spectroscopy with mesitylene as internal standard. Bromide **2h** is a known compound and the ^{1}H NMR spectrum of the unpurified reaction mixture matched with the literature.[16]

Yield: 59% (NMR)

Compound 2i:

2-Bromo-1,2-diphenylethan-1-one

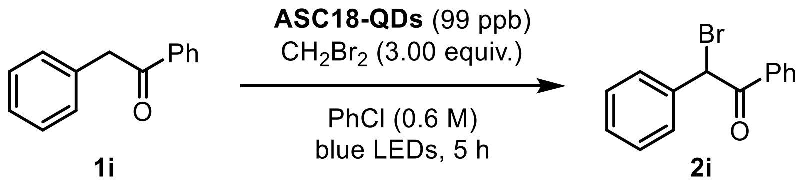

Bromide **2i** was prepared via **GP1** from compound **1i** (118 mg, 0.600 mmol, 1.00 equiv) in PhCl. The crude product was purified via flash column chromatography (100:0 to 60:40 pentane:CH_2Cl_2) to give **2i** as a white solid.

Note: The reaction was stirred for 5 h instead of 3 h under blue light irradiation.

Yield: 80.4 mg, 0.292 mmol, 49%

^{1}H NMR (400 MHz, $CDCl_3$): δ (ppm) = 8.04 – 7.95 (m, 2H), 7.61 – 7.50 (m, 3H), 7.49 – 7.42 (m, 2H), 7.40 – 7.30 (m, 3H), 6.40 (s, 1H).

^{13}C NMR (101 MHz, $CDCl_3$): δ (ppm) = 191.2, 136.0, 134.3, 133.8, 129.3, 129.3, 129.1, 128.9, 51.2.

IR (thin film, cm^{-1}): 3062, 1689, 1595, 1448, 1271, 1214, 1183, 1160, 989, 824, 749, 685, 626, 603, 568, 541.

HRMS (ESI+): *m/z* for $C_{14}H_{11}BrONa$ [M+Na]$^+$: calc.: 296.9885, found: 296.9879

Compound 2j:

1-Bromo-1-phenylpropan-2-one

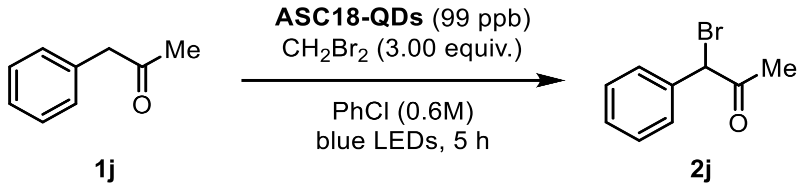

Bromide **2j** was prepared via **GP1** from compound **1j** (79.3 µL, 80.5 mg, 0.600 mmol, 1.00 equiv) in PhCl. The crude product was purified via flash column chromatography (100:0 to 60:40 pentane:CH_2Cl_2) to give **2j** as a colorless oil.

Note: The reaction was stirred for 5 h instead of 3 h under blue light irradiation.

Yield: 79.3 mg, 0.372 mmol, 62%

^{1}H NMR (400 MHz, $CDCl_3$): δ (ppm) = 7.47 – 7.42 (m, 2H), 7.41 – 7.32 (m, 3H), 5.43 (s, 1H), 2.30 (s, 3H).

^{13}C NMR (101 MHz, $CDCl_3$): δ (ppm) = 199.4, 135.2, 129.3, 129.2, 128.9, 56.5, 26.4.

IR (thin film, cm^{-1}): 3030, 1729, 1710, 1454, 1356, 1143, 748, 695, 637, 627, 560, 547.

HRMS (ESI+): *m/z* for C_9H_9BrONa $[M+Na]^+$: calc.: 234.9729, found: 234.9728

Compound 2k:

Ethyl 4-((4-bromo-4-phenylbutanoyl)oxy)benzoate

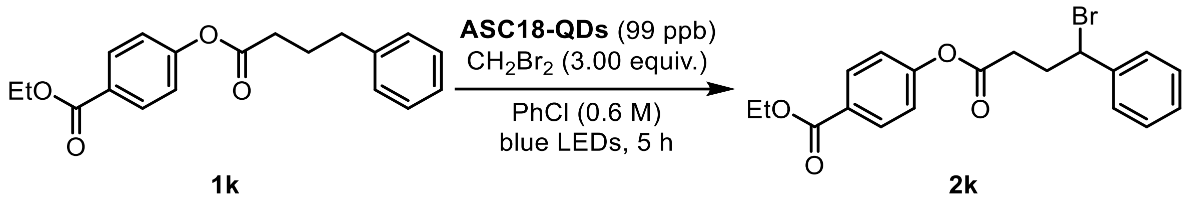

1k **2k**

Bromide **2k** was prepared via **GP1** from compound **1k** (187 mg, 0.600 mmol, 1.00 equiv) in PhCl. The crude product was purified via flash column chromatography (100:0 to 90:10 hexane:acetone) to give **2k** as a colorless oil.

Note: Purification led to elimination of the bromide and resulted in formation of the α,β-unsaturated ester and a decreased isolated yield.

Yield: 143 mg, 0.366 mmol, 61%

Yield: 81% (NMR)

^{1}H NMR (400 MHz, $CDCl_3$): δ (ppm) = 8.09 (d, *J* = 8.9 Hz, 2H), 7.47 – 7.42 (m, 2H), 7.40 – 7.29 (m, 3H), 7.16 (d, *J* = 9.0 Hz, 2H), 5.11 (dd, *J* = 8.7, 5.9 Hz, 1H), 4.38 (q, *J* = 7.2 Hz, 2H), 2.87 – 2.62 (m, 3H), 2.62 – 2.50 (m, 1H), 1.39 (t, *J* = 7.1 Hz, 3H).

^{13}C NMR (101 MHz, $CDCl_3$): δ (ppm) = 170.6, 165.9, 154.2, 141.4, 131.3, 129.0, 128.9, 128.3, 127.4, 121.6, 61.3, 54.0, 34.9, 33.1, 14.5.

IR (thin film, cm^{-1}): 3033, 2982, 1707, 1606, 1591, 1455, 1273, 1202, 1162, 1105, 1017, 853, 822, 761, 696, 617.

HRMS (ESI+): *m/z* for $C_{19}H_{19}BrO_4Na$ $[M+Na]^+$: calc.: 413.0359, found: 413.0351

Compound 2l:

Methyl 2-(4-benzoylphenyl)-2-bromopropanoate

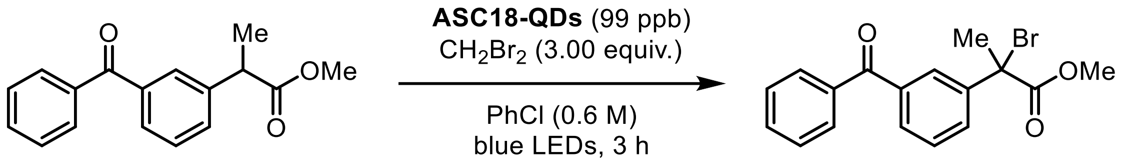

1l **2l**

Bromide **2l** was prepared via **GP1** from compound **1l** (161 mg, 0.600 mmol, 1.00 equiv) in PhCl. The crude product was purified via flash column chromatography (95:5 to 80:20 pentane:Et_2O) to give **2l** as a colorless oil.

Yield: 140 mg, 0.403 mmol, 67%

^{1}H NMR (400 MHz, $CDCl_3$): δ (ppm) = 7.99 (ddd, *J* = 2.2, 1.6, 0.5 Hz, 1H), 7.83 – 7.76 (m, 3H), 7.71 (ddd, *J* = 7.7, 1.6, 1.1 Hz, 1H), 7.60 – 7.55 (m, 1H), 7.50 – 7.43 (m, 3H), 3.78 (s, 3H), 2.31 (s, 3H).

^{13}C NMR (101 MHz, $CDCl_3$): δ (ppm) = 195.8, 171.1, 141.4, 137.6, 137.2, 132.7, 131.1, 130.1, 130.1, 128.4, 128.4, 128.3, 60.7, 53.7, 31.1.

IR (thin film, cm^{-1}): 3001, 2952, 1734, 1659, 1596, 1578, 1447, 1378, 1318, 1279, 1247, 1216, 1178, 1145, 1114, 1076, 1054, 971, 953, 911, 816, 788, 769, 709, 692.

HRMS (ESI+): m/z for $C_{17}H_{15}BrNaO_3$ $[M+Na]^+$: calc.: 369.0097, found: 369.0099.

Compound 2m:

3-Bromo-3-phenylpropyl 4-(*N,N*-dipropylsulfamoyl)benzoate

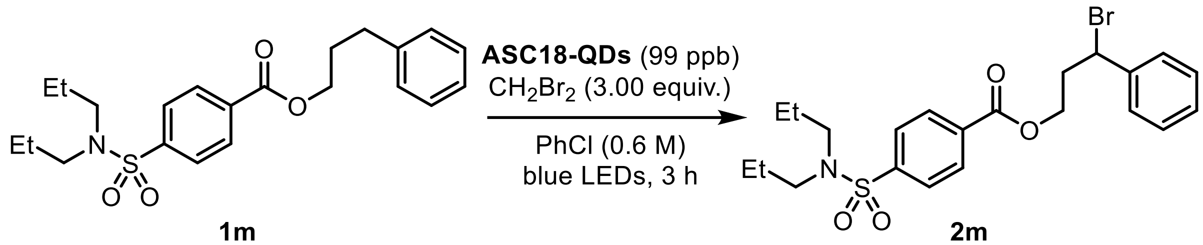

1m 2m

Bromide **2m** was prepared via **GP1** from compound **1m** (107 mg, 0.600 mmol, 1.00 equiv) in PhCl. The crude product was purified via flash column chromatography (100:0 to 90:10 hexane:EtOAc) to give **2m** as a colorless oil.

Yield: 188 mg, 0.390 mmol, 65%

^{1}H NMR (400 MHz, $CDCl_3$): δ (ppm) = 8.12 – 8.04 (m, 2H), 7.91 – 7.83 (m, 2H), 7.45 – 7.40 (m, 2H), 7.38 – 7.27 (m, 3H), 5.15 (dd, *J* = 8.5, 6.4 Hz, 1H), 4.55 – 4.39 (m, 2H), 3.16 – 3.06 (m, 4H), 2.80 – 2.57 (m, 2H), 1.62 – 1.48 (m, 4H), 0.87 (t, *J* = 7.4 Hz, 6H).

^{13}C NMR (101 MHz, $CDCl_3$): δ (ppm) = 165.0, 144.5, 141.3, 133.3, 130.3, 129.0, 128.8, 127.3, 127.1, 63.7, 51.1, 50.0, 38.9, 22.0, 11.3.

IR (thin film, cm^{-1}): 2966, 1722, 1456, 1399, 1341, 1270, 1189, 1176, 1157, 1117, 1106, 1087, 1017, 992, 777, 761, 741, 695, 601, 562.

HRMS (ESI+): *m/z* for $C_{22}H_{28}BrNSO_4Na$ $[M+Na]^+$: calc.: 504.0815, found: 504.0813

Compound 4a:

2-Bromo-1-phenylethan-1-one

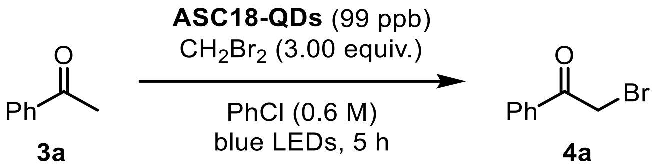

3a 4a

Bromide **4a** was prepared via **GP2** from ketone **3a** (70.1 µL, 72.1 mg, 0.600 mmol, 1.00 equiv) in PhCl. The crude product was purified via flash column chromatography (100:0 to 60:40 pentane:CH_2Cl_2) to give **4a** as a colorless oil.

Yield: 87.0 mg, 0.437 mmol, 73%

^{1}H NMR (400 MHz, $CDCl_3$): δ (ppm) = 8.03 – 7.94 (m, 2H), 7.64 –7.59 (m, 1H), 7.55 – 7.45 (m, 2H), 4.46 (s, 2H).

^{13}C NMR (101 MHz, $CDCl_3$): δ (ppm) = 191.4, 134.1, 129.1, 129.0, 31.1.

IR (thin film, cm^{-1}): 2943, 1698, 1678, 1596, 1580, 1448, 1277, 1193, 1012, 1001, 989, 795, 750, 709, 685, 622, 608, 554, 529.

HRMS (ESI+): *m/z* for C_8H_7BrONa $[M+Na]^+$: calc.: 220.9572, found: 220.9572

Compound 4b:

2-Bromo-1-phenylpropan-1-one

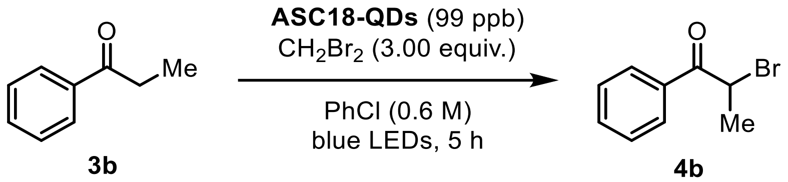

Bromide **4b** was prepared via **GP2** from ketone **3b** (79.8 µL, 80.5 mg, 0.600 mmol, 1.00 equiv) in PhCl. The crude product was purified via flash column chromatography (100:0 to 60:40 pentane:CH_2Cl_2) to give **4b** as a colorless oil.

Yield: 117 mg, 0.549 mmol, 92%

^{1}H NMR (400 MHz, $CDCl_3$): δ (ppm) = 8.07 – 7.99 (m, 2H), 7.64 – 7.54 (m, 1H), 7.54 – 7.44 (m, 2H), 5.30 (q, *J* = 6.6 Hz, 1H), 1.91 (d, *J* = 6.6 Hz, 3H).

^{13}C NMR (101 MHz, $CDCl_3$): δ (ppm) = 193.5, 134.2, 133.8, 129.1, 128.9, 41.6, 20.3.

IR (thin film, cm^{-1}): 2979, 1684, 1596, 1582, 1448, 1345, 1236, 1160, 993, 948, 796, 705, 684, 646, 544.

HRMS (ESI+): *m/z* for C_9H_9BrONa $[M+Na]^+$: calc.: 234.9729, found: 234.9727

Compound 4c:

2-Bromo-2-methyl-1-phenylpropan-1-one

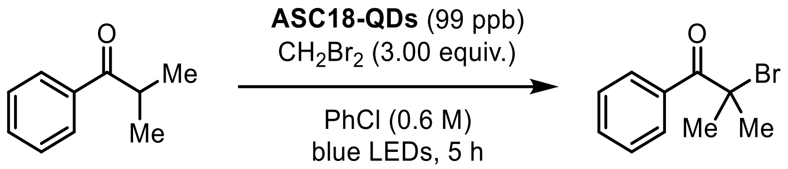

3c **4c**

Bromide **4c** was prepared via **GP2** from ketone **3c** (90.0 µL, 88.9 mg, 0.600 mmol, 1.00 equiv) in PhCl. The crude product was purified via flash column chromatography (100:0 to 60:40 pentane:CH_2Cl_2) to give **4c** as a colorless oil.

Yield: 126 mg, 0.555 mmol, 93%

^{1}H NMR (400 MHz, $CDCl_3$): δ (ppm) = 8.18 – 8.09 (m, 2H), 7.58 – 7.49 (m, 1H), 7.47 – 7.38 (m, 2H), 2.04 (s, 6H).

^{13}C NMR (101 MHz, $CDCl_3$): δ (ppm) = 197.0, 135.0, 132.5, 130.2, 128.3, 60.5, 31.7.

IR (thin film, cm^{-1}): 2976, 1674, 1459, 1446, 1387, 1370, 1268, 1167, 1103, 977, 884, 791, 721, 703, 689, 627, 477.

HRMS (ESI+): *m/z* for $C_{10}H_{11}BrONa$ [M+Na]$^+$: calc.: 248.9885, found: 248.9883

Compound 4d:

2-Bromo-1-(4-bromophenyl)propan-1-one

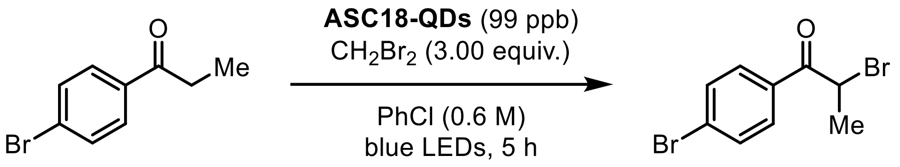

3d **4d**

Bromide **4d** was prepared via **GP2** from ketone **3d** (128 mg, 0.600 mmol, 1.00 equiv) in PhCl. The crude product was purified via flash column chromatography (100:0 to 60:40 pentane:CH_2Cl_2) to give **4d** as a colorless oil.

Yield: 166 mg, 0.569 mmol, 95%

^{1}H NMR (400 MHz, $CDCl_3$): δ (ppm) = 7.92 – 7.84 (m, 2H), 7.67 – 7.58 (m, 2H), 5.21 (q, *J* = 6.6 Hz, 1H), 1.89 (d, *J* = 6.6 Hz, 3H).

^{13}C NMR (101 MHz, $CDCl_3$): δ (ppm) = 192.5, 132.9, 132.2, 130.5, 129.1, 41.4, 20.1.

IR (thin film, cm^{-1}): 2997, 1677, 1584, 1564, 1397, 1341, 1244, 1164, 1070, 1057, 1011, 994, 957, 947, 843, 763, 744, 537, 487.

HRMS (ESI+): *m/z* for $C_9H_8Br_2ONa$ $[M+Na]^+$: calc.: 312.8834, found: 312.8829

Compound 4e:

2-Bromo-1-(4-methoxyphenyl)propan-1-one

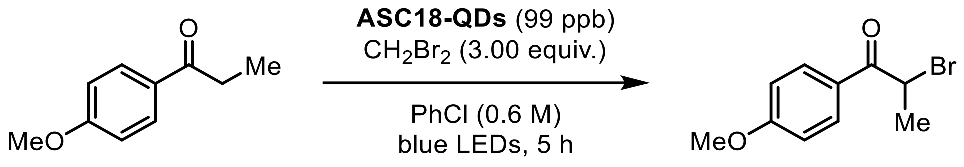

Bromide **4e** was prepared via **GP2** from ketone **3e** (98.5 mg, 0.600 mmol, 1.00 equiv) in PhCl. The crude product was purified via flash column chromatography (100:0 to 60:40 pentane:CH_2Cl_2) to give **4e** as a colorless oil.

Yield: 134 mg, 0.551 mmol, 92%

^{1}H NMR (400 MHz, $CDCl_3$): δ (ppm) = 8.05 – 7.97 (m, 2H), 6.99 – 6.91 (m, 2H), 5.26 (q, *J* = 6.6 Hz, 1H), 3.88 (s, 3H), 1.88 (d, *J* = 6.6 Hz, 3H).

^{13}C NMR (101 MHz, $CDCl_3$): δ (ppm) = 192.1, 164.1, 131.4, 127.0, 114.1, 55.7, 41.6, 20.4.

IR (thin film, cm^{-1}): 2974, 1674, 1597, 1573, 1510, 1441, 1420, 1345, 1308, 1264, 1238, 1177, 1157, 1026, 993, 949, 841, 762, 603, 547.

HRMS (ESI+): *m/z* for $C_{10}H_{11}BrO_2Na$ $[M+Na]^+$: calc.: 264.9835, found: 264.9830

Compound 4f:

(1-Bromocyclohexyl)(phenyl)methanone

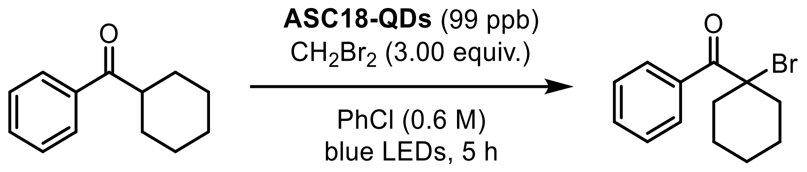

3f **4f**

Bromide **4f** was prepared via **GP2** from ketone **3f** (113 mg, 0.600 mmol, 1.00 equiv) in PhCl. The crude product was purified via flash column chromatography (100:0 to 60:40 pentane:CH_2Cl_2) to give **4f** as a colorless oil.

Yield: 120 mg, 0.449 mmol, 75%

^{1}H NMR (400 MHz, $CDCl_3$): δ (ppm) = 8.12 – 8.03 (m, 2H), 7.56 – 7.46 (m, 1H), 7.45 – 7.33 (m, 2H), 2.38 – 2.26 (m, 2H), 2.23 – 2.12 (m, 2H), 1.87 – 1.72 (m, 2H), 1.65 – 1.45 (m, 3H), 1.45 – 1.31 (m, 1H)

^{13}C NMR (101 MHz, $CDCl_3$): δ (ppm) = 197.6, 136.0, 132.1, 129.9, 128.2, 68.1, 38.4, 25.1, 23.7.

IR (thin film, cm^{-1}): 2934, 1674, 1446, 1281, 1244, 1232, 1200, 1181, 984, 973, 874, 824, 789, 752, 698, 655, 475.

HRMS (ESI+): *m/z* for $C_{13}H_{15}BrONa$ $[M+Na]^+$: calc.: 289.0198, found: 289.0198

Compound 4g:

(1-Bromocyclopentyl)(phenyl)methanone

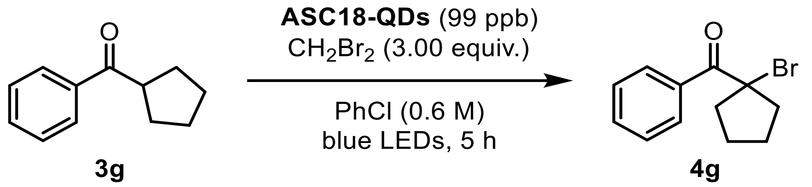

Bromide **4g** was prepared via **GP2** from ketone **3g** (105 mg, 0.600 mmol, 1.00 equiv) in PhCl. The crude product was purified via flash column chromatography (100:0 to 60:40 pentane:CH_2Cl_2) to give **4g** as a colorless oil.

Yield: 76.2 mg, 0.301 mmol, 50%

^{1}H NMR (400 MHz, $CDCl_3$): δ (ppm) = 8.22 – 8.12 (m, 2H), 7.60 – 7.49 (m, 1H), 7.49 – 7.40 (m, 2H), 2.58 – 2.36 (m, 4H), 2.17 – 1.97 (m, 2H), 1.89 – 1.71 (m, 2H).

^{13}C NMR (101 MHz, $CDCl_3$): δ (ppm) = 195.4, 134.9, 132.9, 130.4, 128.3, 71.3, 41.2, 23.7.

IR (thin film, cm^{-1}): 2960, 1672, 1597, 1447, 1259, 1172, 945, 888, 795, 702, 688, 660.

HRMS (ESI+): *m/z* for $C_{12}H_{13}BrONa$ $[M+Na]^+$: calc.: 275.0042, found: 275.0043

Compound 4h:

(1-Bromocyclobutyl)(phenyl)methanone

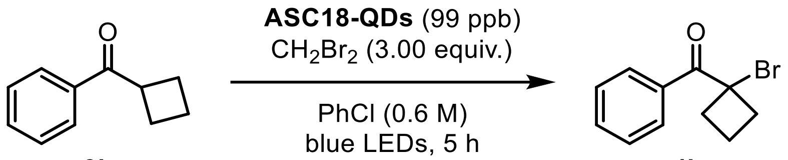

3h **4h**

Bromide **4h** was prepared via **GP2** from ketone **3h** (96.1 µL, 91.5 mg, 0.600 mmol, 1.00 equiv) in PhCl. The crude product was purified via flash column chromatography (100:0 to 60:40 pentane:CH_2Cl_2) to give **4h** as a colorless oil.

Yield: 102 mg, 0.427 mmol, 71%

^{1}H NMR (400 MHz, $CDCl_3$): δ (ppm) = 8.09 – 8.01 (m, 2H), 7.60 – 7.51 (m, 1H), 7.51 – 7.40 (m, 2H), 3.20 – 3.07 (m, 2H), 2.83 – 2.71 (m, 2H), 2.49 – 2.33 (m, 1H), 1.94 – 1.79 (m, 1H).

^{13}C NMR (101 MHz, $CDCl_3$): δ (ppm) = 194.6, 133.4, 132.4, 130.3, 128.5, 59.8, 37.2, 16.6.

IR (thin film, cm^{-1}): 2953, 1675, 1597, 1448, 1279, 1248, 1209, 1182, 1073, 958, 850, 798, 698, 688, 667, 608.

HRMS (ESI+): *m/z* for $C_{11}H_{11}BrONa$ $[M+Na]^+$: calc.: 260.9885, found: 260.9884

Compound 4i:

1-((3r,5r,7r)-Adamantan-1-yl)-2-bromoethan-1-one

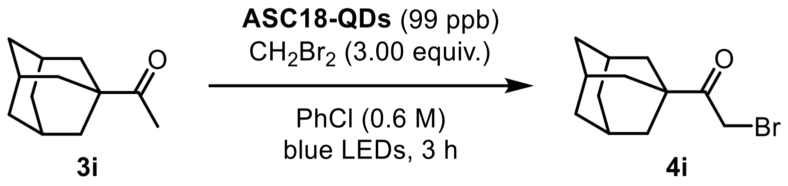

Bromide **4i** was prepared via **GP2** from ketone **3i** (107 mg, 0.600 mmol, 1.00 equiv) in PhCl. The crude product was purified via flash column chromatography (100:0 to 60:40 pentane:CH_2Cl_2) to give **4i** as a white solid.

Note: The reaction was stirred for 3 h instead of 5 h in the blue LED photoreactor.

Yield: 108 mg, 0.420 mmol, 70%

^{1}H NMR (400 MHz, $CDCl_3$): δ (ppm) = 4.15 (s, 2H), 2.08 – 2.05 (m, 3H), 1.88 – 1.87 (m, 6H), 1.78 – 1.67 (m, 6H).

^{13}C NMR (101 MHz, $CDCl_3$): δ (ppm) = 205.7, 46.8, 38.7, 36.5, 32.0, 28.0.

IR (thin film, cm^{-1}): 2906, 2850, 1705, 1452, 1151, 1010, 907, 729, 693, 648, 629.

HRMS (ESI+): *m/z* for $C_{12}H_{17}BrONa$ $[M+Na]^+$: calc.: 279.0355, found: 279.0353

X-Ray: See page 149

Compound 4j:

6-Bromo-2,2-dimethylcyclohexan-1-one

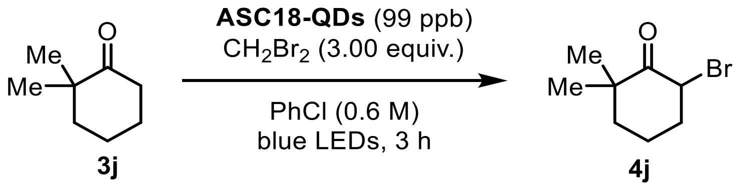

Bromide **4j** was prepared via **GP2** from ketone **3j** (83.0 µL, 75.7 mg, 0.600 mmol, 1.00 equiv) in PhCl. The crude product was purified via flash column chromatography (100:0 to 60:40 pentane:CH_2Cl_2) to give **4j** as a colorless oil.

Note: The reaction was stirred for 3 h instead of 5 h in the blue LED photoreactor.

Yield: 77.1 mg, 0.376 mmol, 63%

^{1}H NMR (400 MHz, $CDCl_3$): δ (ppm) = 4.87 (dd, *J* = 11.9, 5.9 Hz, 1H), 2.63 – 2.52 (m, 1H), 2.17 – 2.03 (m, 1H), 1.99 – 1.71 (m, 3H), 1.71 – 1.57 (m, 1H), 1.19 (d, *J* = 4.8 Hz, 6H).

^{13}C NMR (101 MHz, $CDCl_3$): δ (ppm) = 206.1, 54.2, 46.6, 40.8, 39.8, 26.8, 25.4, 22.1.

IR (thin film, cm^{-1}): 2967, 2939, 1722, 1472, 1452, 1079, 1052, 991, 919, 752, 710, 555, 514.

HRMS (ESI+): *m/z* for $C_8H_{13}BrONa$ $[M+Na]^+$: calc.: 227.0042, found: 227.0043

Compound 4k:

4-Bromo-2,2-dimethylhexan-3-one

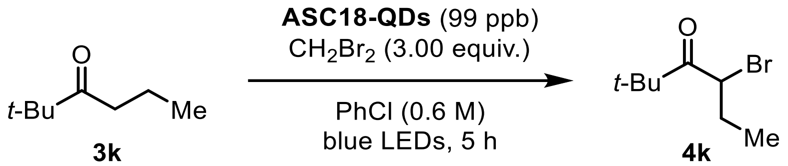

3k **4k**

Bromide **4k** was prepared via **GP2** from ketone **3k** (94.7 µL, 76.9 mg, 0.600 mmol, 1.00 equiv) in PhCl. The crude product was purified via flash column chromatography (100:0 to 60:40 pentane:CH_2Cl_2) to give **4k** as a colorless oil.

Yield: 101 mg, 0.488 mmol, 81%

^{1}H NMR (400 MHz, $CDCl_3$): δ (ppm) = 4.54 (t, *J* = 7.2 Hz, 1H), 2.09 – 1.87 (m, 2H), 1.22 (d, *J* = 0.9 Hz, 9H), 0.95 (td, *J* = 7.3, 0.9 Hz, 3H).

^{13}C NMR (101 MHz, $CDCl_3$): δ (ppm) = 209.2, 47.1, 44.4, 27.9, 26.8, 12.4.

IR (thin film, cm^{-1}): 2970, 2937, 1710, 1478, 1464, 1368, 1113, 1054, 993, 900, 808, 773, 578.

HRMS (ESI+): *m/z* for $C_8H_{15}BrONa$ $[M+Na]^+$: calc.: 229.0198, found: 229.0197

Compound 4l:

1-Bromo-3,3-dimethylbutan-2-one

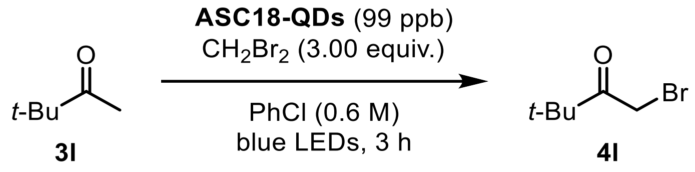

Bromide **4l** was prepared via **GP2** from ketone **3l** (75.0 µL, 60.1 mg, 0.600 mmol, 1.00 equiv) in PhCl. The crude product was purified via flash column chromatography (100:0 to 60:40 pentane:CH_2Cl_2) to give **4l** as a colorless oil.

Note: The reaction was stirred for 3 h instead of 5 h in the blue LED photoreactor.

Yield: 69.5 mg, 0.388 mmol, 65%

^{1}H NMR (400 MHz, $CDCl_3$): δ (ppm) = 4.16 (s, 2H), 1.22 (s, 9H).

^{13}C NMR (101 MHz, $CDCl_3$): δ (ppm) = 206.2, 44.4, 31.8, 26.8.

IR (thin film, cm^{-1}): 2969, 1717, 1478, 1467, 1394, 1367, 1056, 1002, 683, 581, 566.

HRMS (ESI+): *m/z* for $C_6H_{11}BrONa$ [M+Na]$^+$: calc.: 200.9885, found: 200.9884

Compound 4m:

2-Bromo-2,4-dimethylpentan-3-one

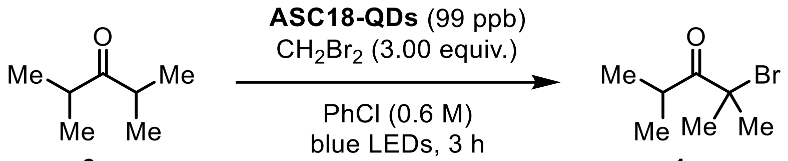

3m **4m**

Bromide **4m** was prepared via **GP2** from ketone **3m** (85.0 µL, 68.5 mg, 0.600 mmol, 1.00 equiv) in PhCl. Due to the volatility of the product, **4m** was not isolated and the yield was determined via ^{1}H NMR spectroscopy with mesitylene as internal standard. Bromide **4m** is a known compound and the ^{1}H NMR spectrum of the unpurified reaction mixture matched with the literature.[17]

Note: The reaction was stirred for 3 h instead of 5 h in the blue LED photoreactor.

Yield: 66% (NMR)

Compound 6a:

Methyl 2-bromo-4,4-dimethyl-3-oxopentanoate

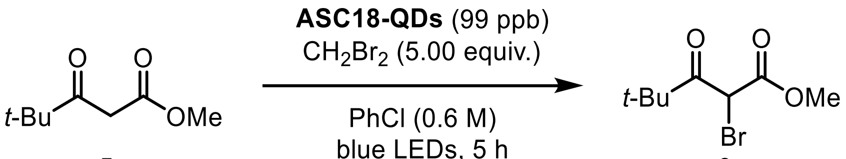

5a **6a**

Bromide **6a** was prepared via **GP3** from β-ketoester **5a** (96.9 µL, 94.9 mg, 0.600 mmol, 1.00 equiv) in PhCl. The crude product was purified via flash column chromatography (100:0 to 60:40 pentane:CH_2Cl_2) to give **6a** as a colorless oil.

Note: In a modification of **GP3**, 5.00 equivalents CH_2Br_2 (209 µL, 518 mg, 3.00 mmol) were used in the bromination reaction.

Yield: 133 mg, 0.561 mmol, 93%

Yield (10 mmol scale): 2.31 g, 9.74 mmol, 97%

^{1}H NMR (400 MHz, $CDCl_3$): δ (ppm) = 5.25 (s, 1H), 3.81 (s, 3H), 1.27 (s, 9H).

^{13}C NMR (101 MHz, $CDCl_3$): δ (ppm) = 203.7, 165.8, 54.0, 45.5, 43.1, 26.8.

IR (thin film, cm^{-1}): 2973, 1764, 1744, 1716, 1478, 1436, 1300, 1268, 1217, 1197, 1150, 1055, 988.

HRMS (ESI+): *m/z* for $C_8H_{13}BrO_3Na$ $[M+Na]^+$: calc.: 258.9940, found: 258.9942

Compound 6b:

Ethyl 2-bromo-3-cyclopropyl-3-oxopropanoate

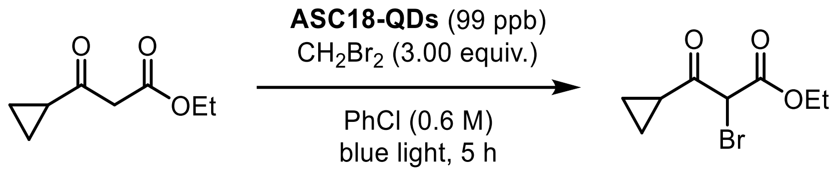

5b **6b**

Bromide **6b** was prepared via **GP3** from β-ketoester **5b** (88.6 μL, 93.7 mg, 0.600 mmol, 1.00 equiv) in PhCl. The crude product was purified via flash column chromatography (100:0 to 60:40 pentane:CH_2Cl_2) to give **6b** as a colorless oil.

Yield: 119.1 mg, 0.476 mmol, 79%

^{1}H NMR (400 MHz, $CDCl_3$): δ (ppm) = 4.89 (s, 1H), 4.29 (q, *J* = 7.1 Hz, 2H), 2.29 (tt, *J* = 7.8, 4.5 Hz, 1H), 1.30 (t, *J* = 7.1 Hz, 3H), 1.20 – 1.14 (m, 2H), 1.09 – 1.03 (m, 2H).

^{13}C NMR (101 MHz, $CDCl_3$): δ (ppm) = 198.9, 165.3, 63.2, 49.7, 18.7, 14.1, 13.3, 13.2.

IR (thin film, cm^{-1}): 2984, 1737, 1720, 1702, 1382, 1298, 1266, 1215, 1194, 1139, 1088, 1064, 1018, 904, 858.

HRMS (ESI+): *m/z* for $C_8H_{11}BrO_3Na$ $[M+Na]^+$: calc.: 256.9784, found: 256.9778

Compound 6c:

***tert*-Butyl 2-bromo-3-oxobutanoate**

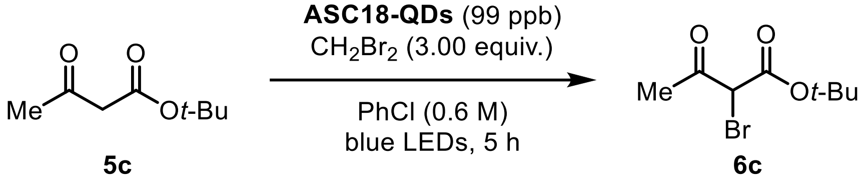

Bromide **6c** was prepared via **GP3** from β-ketoester **5c** (96.9 µL, 94.9 mg, 0.600 mmol, 1.00 equiv) in PhCl. The crude product was purified via flash column chromatography (100:0 to 60:40 pentane:CH_2Cl_2) to give **6c** as a colorless oil.

Yield: 94.3 mg, 0.398 mmol, 66%

^{1}H NMR (400 MHz, $CDCl_3$): δ (ppm) = 4.67 (s, 1H), 2.42 (s, 3H), 1.50 (s, 9H).

^{13}C NMR (101 MHz, $CDCl_3$): δ (ppm) = 196.8, 164.2, 84.6, 50.8, 27.9, 26.4.

IR (thin film, cm^{-1}): 2981, 1725, 1371, 1396, 1360, 1306, 1287, 1259, 1137, 846.

HRMS (ESI+): *m/z* for $C_8H_{13}BrO_3Na$ $[M+Na]^+$: calc.: 258.9940, found: 258.9940

Compound 6d:

Methyl 2-bromo-3-oxobutanoate

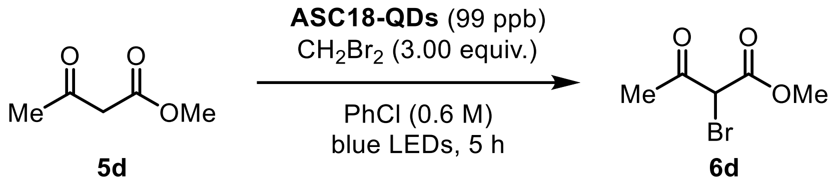

Bromide **6d** was prepared via **GP3** from β-ketoester **5d** (64.7 µL, 69.7 mg, 0.600 mmol, 1.00 equiv) in PhCl. Due to the volatility of the product, **6d** was not isolated and the yield was determined via ^{1}H NMR spectroscopy with mesitylene as internal standard. Bromide **6d** is a known compound and the ^{1}H NMR spectrum of the unpurified reaction mixture matched with the literature.[3]

Yield: 65% (NMR)

Compound 6e:

Methyl 4-bromo-3-oxopentanoate

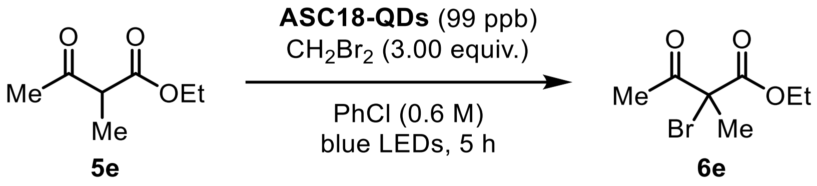

Bromide **6e** was prepared via **GP3** from β-ketoester **5e** (84.9 µL, 86.5 mg, 0.600 mmol, 1.00 equiv) in PhCl. The crude product was purified via flash column chromatography (100:0 to 60:40 pentane:CH_2Cl_2) to give **6e** as a colorless oil.

Yield: 89.0 mg, 0.399 mmol, 66%

^{1}H NMR (400 MHz, $CDCl_3$): δ (ppm) = 4.27 (qd, *J* = 7.1, 1.6 Hz, 2H), 2.43 (s, 3H), 1.97 (s, 3H), 1.30 (t, *J* = 7.1 Hz, 3H).

^{13}C NMR (101 MHz, $CDCl_3$): δ (ppm) = 198.3, 168.4, 63.3, 62.8, 25.9, 25.4, 14.0.

IR (thin film, cm^{-1}): 2986, 1723, 1445, 1376, 1358, 1244, 1200, 1117, 1096, 1068, 1015, 862.

HRMS (ESI+): *m/z* for $C_7H_{11}BrO_3Na$ $[M+Na]^+$: calc.: 244.9784, found: 244.9780

Compound 6f:

Methyl 2-bromo-2-ethyl-3-oxobutanoate

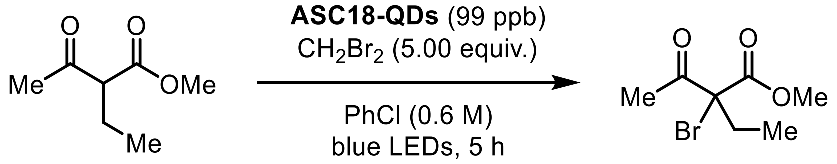

5f **6f**

Bromide **6f** was prepared via **GP3** from β-ketoester **5f** (85.6 μL, 86.5 mg, 0.600 mmol, 1.00 equiv) in PhCl. The crude product was purified via flash column chromatography (100:0 to 60:40 pentane:CH_2Cl_2) to give **6f** as a colorless oil.

Note: In a modification of **GP3**, 5.00 equivalents CH_2Br_2 (209 μL, 518 mg, 3.00 mmol) were used in the bromination reaction.

Yield: 60.3 mg, 0.270 mmol, 45%

^{1}H NMR (400 MHz, $CDCl_3$): δ (ppm) = 3.82 (s, 3H), 2.39 (s, 3H), 2.25 (ddt, *J* = 19.5, 14.8, 7.4 Hz, 2H), 1.00 (t, *J* = 7.3 Hz, 3H).

^{13}C NMR (101 MHz, $CDCl_3$): δ (ppm) = 197.8, 168.2, 70.4, 53.9, 30.8, 26.4, 10.0.

IR (thin film, cm^{-1}): 2957, 1720, 1458, 1437, 1358, 1296, 1280, 1229, 1208, 1183, 1126, 1091, 1020, 982, 836, 804, 601, 555.

HRMS (ESI+): *m/z* for $C_7H_{11}BrO_3Na$ $[M+Na]^+$: calc.: 244.9784, found: 244.9780

Compound 6g:

Ethyl 3-(1-bromocyclohexyl)-3-oxopropanoate

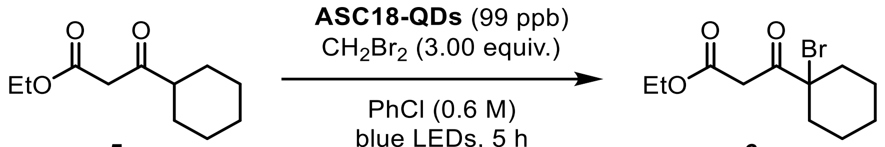

5g 6g

Bromide **6g** was prepared via **GP3** from β-ketoester **5g** (119.0 mg, 0.600 mmol, 1.00 equiv) in PhCl. The crude product was purified via flash column chromatography (100:0 to 60:40 pentane:CH_2Cl_2) to give **6g** as a colorless oil.

Note: The ^1H and ^{13}C NMR spectra show small amounts of the enol form of **6g**.

Yield: 123.7 mg, 0.448 mmol, 75%

^{1}H NMR (500 MHz, $CDCl_3$): δ (ppm) = 4.20 (q, *J* = 7.1 Hz, 2H), 3.84 (s, 2H), 2.18 – 2.11 (m, 2H), 2.00 – 1.91 (m, 2H), 1.82 – 1.72 (m, 2H), 1.70 – 1.56 (m, 2H), 1.28 (t, *J* = 7.1 Hz, 3H).

^{13}C NMR (126 MHz, $CDCl_3$): δ (ppm) = 197.6, 167.5, 71.9, 61.6, 43.4, 36.2, 25.0, 23.1, 14.3.

IR (thin film, cm^{-1}): 2937, 1744, 1711, 1447, 1367, 1317, 1248, 1231, 1143, 1116, 1033, 1001.

HRMS (ESI+): *m/z* for $C_{11}H_{17}BrO_3Na$ $[M+Na]^+$: calc.: 299.0253, found: 299.0258

Compound 6h:

Ethyl 3-(1-bromocyclopentyl)-3-oxopropanoate

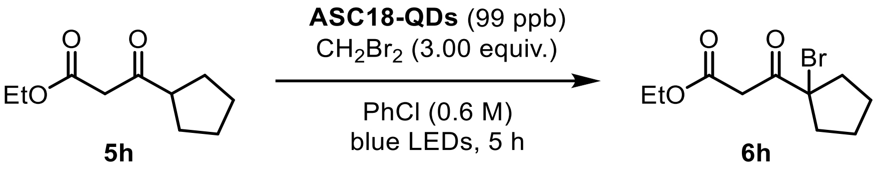

Bromide **6h** was prepared via **GP3** from β-ketoester **5h** (110.5 mg, 0.600 mmol, 1.00 equiv) in PhCl. The crude product was purified via flash column chromatography (100:0 to 60:40 pentane:CH_2Cl_2) to give **6h** as a colorless oil.

Note: The ^{1}H and ^{13}C NMR spectra show small amounts of the enol form of **6h**.

Yield: 131.3 mg, 0.499 mmol, 83%

^{1}H NMR (500 MHz, $CDCl_3$): δ (ppm) = 4.20 (q, *J* = 7.1 Hz, 2H), 3.87 (s, 2H), 2.38 – 2.28 (m, 2H), 2.26 – 2.18 (m, 2H), 2.09 – 1.95 (m, 2H), 1.85 – 1.76 (m, 2H), 1.28 (t, *J* = 7.1 Hz, 3H).

^{13}C NMR (126 MHz, $CDCl_3$): δ (ppm) = 197.1, 167.3, 74.8, 61.7, 45.1, 39.3, 23.4, 14.2.

IR (thin film, cm^{-1}): 2977, 1744, 1711, 1409, 1368, 1318, 1248, 1212, 1144, 1097, 1031.

HRMS (ESI+): *m/z* for $C_{10}H_{15}BrO_3Na$ $[M+Na]^+$: calc.: 285.0097, found: 285.0101

Compound 6i:

Methyl 4-bromo-3-oxopentanoate

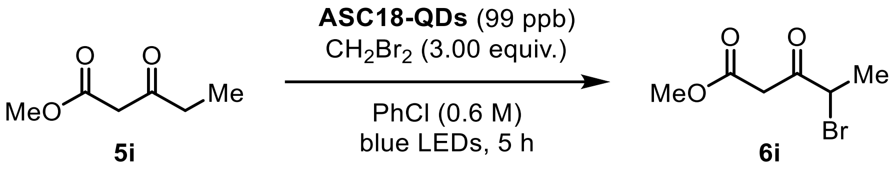

Bromide **6i** was prepared via **GP3** from β-ketoester **5i** (78.1 mg, 0.600 mmol, 1.00 equiv) in PhCl. The crude product was purified via flash column chromatography (100:0 to 60:40 pentane:CH_2Cl_2) to give **6i** as a colorless oil.

Note: The ^{1}H and ^{13}C NMR spectra show small amounts of the enol form of **6i**.

Yield: 82.9 mg, 0.397 mmol, 66%

^{1}H NMR (500 MHz, $CDCl_3$): δ (ppm) = 4.62 (q, *J* = 6.8 Hz, 1H), 3.85 (d, *J* = 16.1 Hz, 1H), 3.75 (s, 3H), 3.68 (d, *J* = 16.1 Hz, 1H), 1.77 (d, *J* = 6.8 Hz, 3H).

^{13}C NMR (126 MHz, $CDCl_3$): δ (ppm) = 196.5, 167.6, 52.7, 47.3, 45.1, 19.8.

IR (thin film, cm^{-1}): 2956, 1748, 1719, 1438, 1322, 1237, 1201, 1151, 1109, 1005.

HRMS (ESI+): *m/z* for $C_6H_9BrO_3Na$ $[M+Na]^+$: calc.: 230.9627, found: 230.9627

Compound 6j:

Methyl 4-bromo-4-methyl-3-oxopentanoate

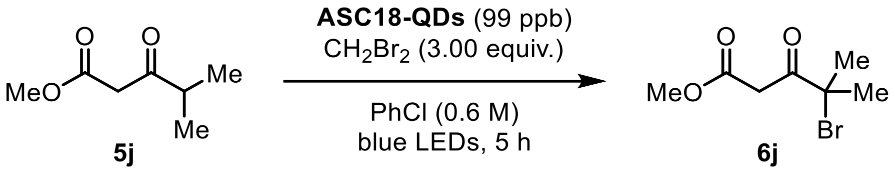

Bromide **6j** was prepared via **GP3** from β-ketoester **5j** (86.5 mg, 0.600 mmol, 1.00 equiv) in PhCl. The crude product was purified via flash column chromatography (100:0 to 60:40 pentane:CH_2Cl_2) to give **6j** as a colorless oil.

Note: The ^{1}H and ^{13}C NMR spectra show small amounts of the enol form of **6j**.

Yield: 95.1 mg, 0.426 mmol, 71%

^{1}H NMR (500 MHz, $CDCl_3$): δ (ppm) = 3.88 (s, 2H), 3.74 (s, 3H), 1.88 (s, 6H).

^{13}C NMR (126 MHz, $CDCl_3$): δ (ppm) = 197.9, 167.8, 63.7, 52.6, 43.1, 29.4.

IR (thin film, cm^{-1}): 1747, 1715, 1437, 1324, 1260, 1216, 1153, 1108, 1058, 1018, 996.

HRMS (ESI+): *m/z* for $C_7H_{11}BrO_3Na$ $[M+Na]^+$: calc.: 244.9784, found: 244.9780

16. Synthesis of starting materials

Compound 1k:

Ethyl 4-((4-phenylbutanoyl)oxy)benzoate

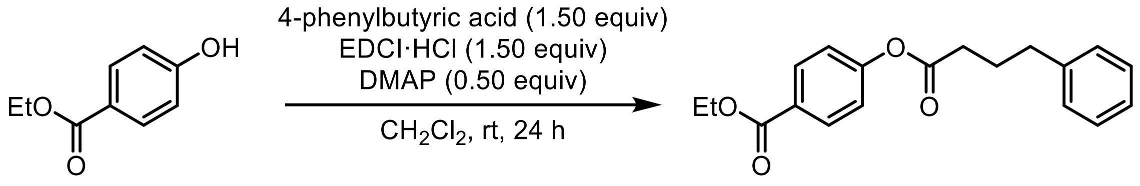

1k

To a 250 mL round-bottom flask charged with ethylparaben (1.25 g, 7.50 mmol, 1.00 equiv) in CH_2Cl_2 (50 mL) were added 4-phenylbutyric acid (1.85 g, 11.3 mmol, 1.50 equiv), 1-ethyl-3-(3-dimethylaminopropyl)carbodiimide hydrochloride (2.16 g, 11.3 mmol, 1.50 equiv), and 4-dimethylaminopyridine (458 mg, 3.75 mmol, 0.50 equiv) at 0 °C. After stirring for 24 h at room temperature, the reaction mixture was diluted with water (100 mL) and extracted with CH_2Cl_2 (3 x 150 mL). The combined organic phases were dried over Na_2SO_4. The solvent was removed in vacuo and the crude product was purified via flash column chromatography (0-20% EtOAc in hexane) to give **1k** as a colorless oil.

Yield: 2.23 g, 7.12 mmol, 95%

^{1}H NMR (400 MHz, $CDCl_3$): δ (ppm) = 8.11 – 8.04 (m, 2H), 7.36 – 7.28 (m, 2H), 7.25 – 7.20 (m, 3H), 7.17 – 7.10 (m, 2H), 4.38 (q, *J* = 7.1 Hz, 2H), 2.75 (t, *J* = 7.6 Hz, 2H), 2.60 (t, *J* = 7.5 Hz, 2H), 2.10 (p, *J* = 7.6 Hz, 2H), 1.39 (t, *J* = 7.1 Hz, 3H).

^{13}C NMR (101 MHz, $CDCl_3$): δ (ppm) = 171.5, 166.0, 154.4, 141.2, 131.2, 128.7, 128.6, 128.1, 126.3, 121.6, 61.2, 35.2, 33.8, 26.5, 14.5.

IR (thin film, cm^{-1}): 2981, 2937, 1758, 1714, 1603, 1504, 1454, 1413, 1367, 1271, 1200, 1160, 1096, 1017, 925, 858, 745, 698.

HRMS (ESI+): m/z for $C_{19}H_{20}NaO_4$ $[M+Na]^+$: calc.: 335.1254, found: 335.1258.

Compound 1m:

3-Phenylpropyl 4-(*N*,*N*-dipropylsulfamoyl)benzoate

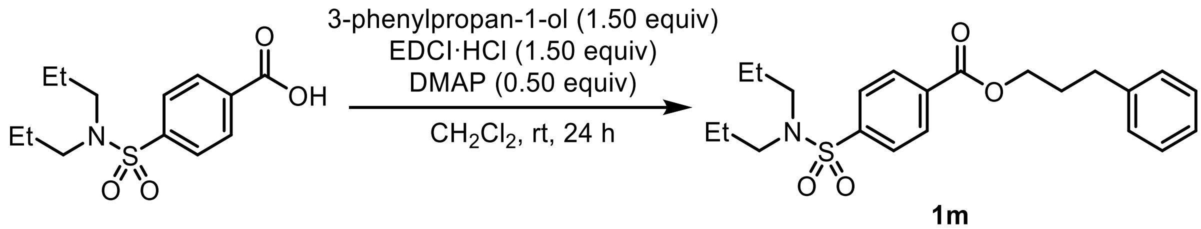

To a 250 mL round-bottom flask charged with probenecid (1.43 g, 5.00 mmol, 1.00 equiv) in CH_2Cl_2 (50 mL) were added 3-phenylpropan-1-ol (1.02 mL, 1.02 g, 7.50 mmol, 1.50 equiv), 1-ethyl-3-(3-dimethylaminopropyl)carbodiimide hydrochloride (1.44 g, 7.50 mmol, 1.50 equiv), and 4-dimethylaminopyridine (305 mg, 2.50 mmol, 0.50 equiv) at 0 °C. After stirring for 24 h at room temperature, the reaction mixture was diluted with water (100 mL) and extracted with CH_2Cl_2 (3 x 150 mL). The combined organic phases were dried over Na_2SO_4. The solvent was removed in vacuo and the crude product was purified via flash column chromatography (0-15% EtOAc in hexane) to give **1m** as a colorless oil.

Yield: 1.89 g, 4.68 mmol, 94%

^{1}H NMR (400 MHz, $CDCl_3$): δ (ppm) = 8.15 – 8.08 (m, 2H), 7.91 – 7.83 (m, 2H), 7.34 – 7.26 (m, 2H), 7.24 – 7.17 (m, 3H), 4.38 (t, *J* = 6.5 Hz, 2H), 3.15 – 3.06 (m, 4H), 2.79 (dd, *J* = 8.3, 6.8 Hz, 2H), 2.19 – 2.07 (m, 2H), 1.62 – 1.48 (m, 4H), 0.87 (t, *J* = 7.4 Hz, 6H).

^{13}C NMR (101 MHz, $CDCl_3$): δ (ppm) = 165.3, 144.3, 141.1, 133.7, 130.3, 128.6, 128.5, 127.1, 126.2, 65.1, 50.0, 32.4, 30.2, 22.0, 11.3.

IR (thin film, cm^{-1}): 2965, 2935, 2986, 1720, 1467, 1342, 1270, 1157, 1117, 1087, 1107, 991, 861, 739, 696.

HRMS (ESI+): m/z for $C_{22}H_{30}NO_4S$ $[M+H]^+$: calc.: 404.1890, found: 404.1885.

17. ^{1}H NMR and ^{13}C NMR spectra

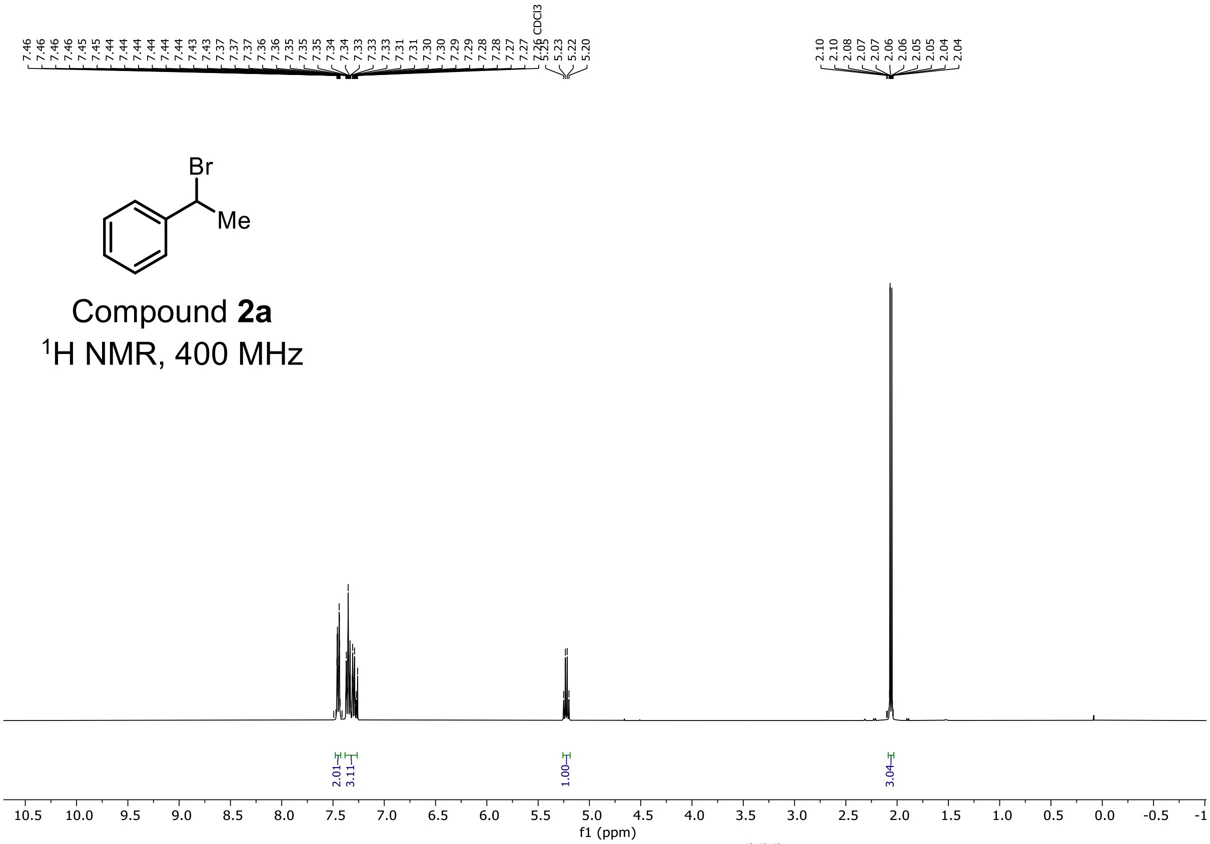

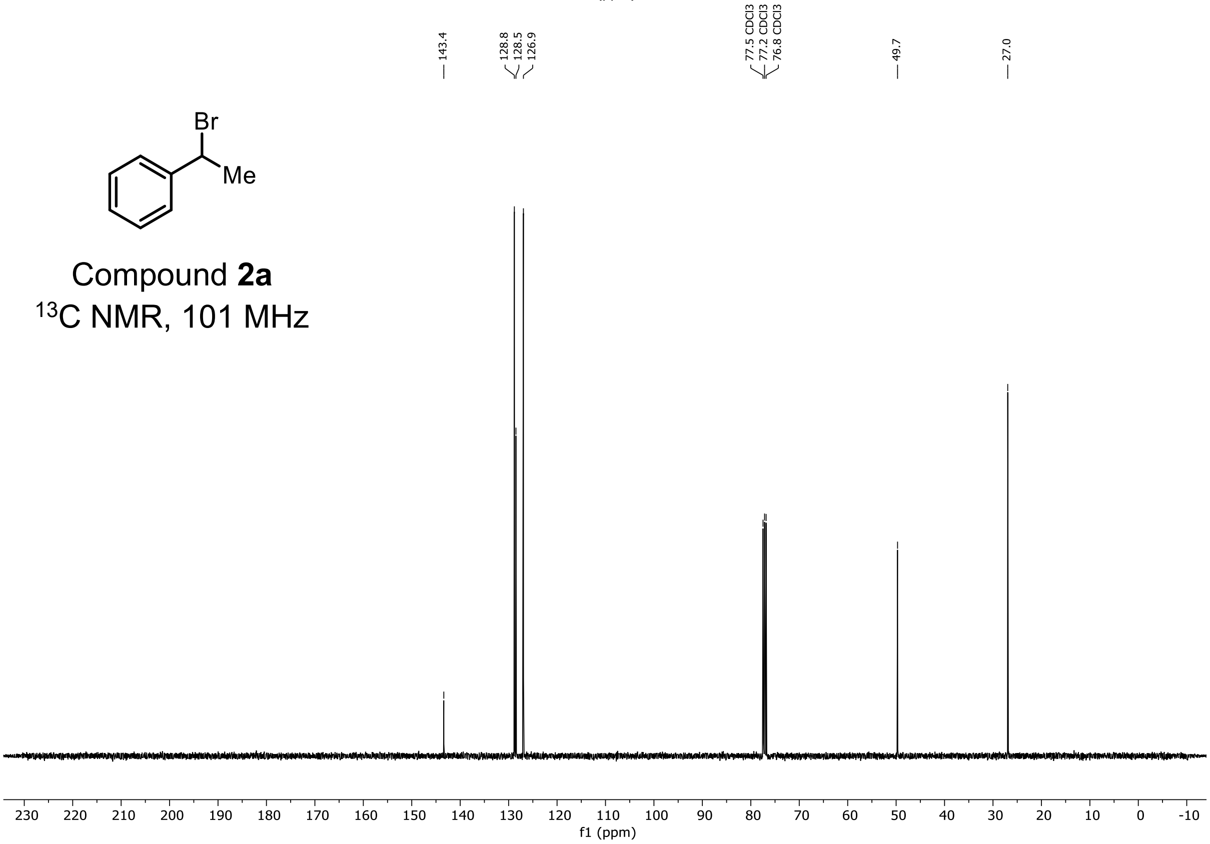

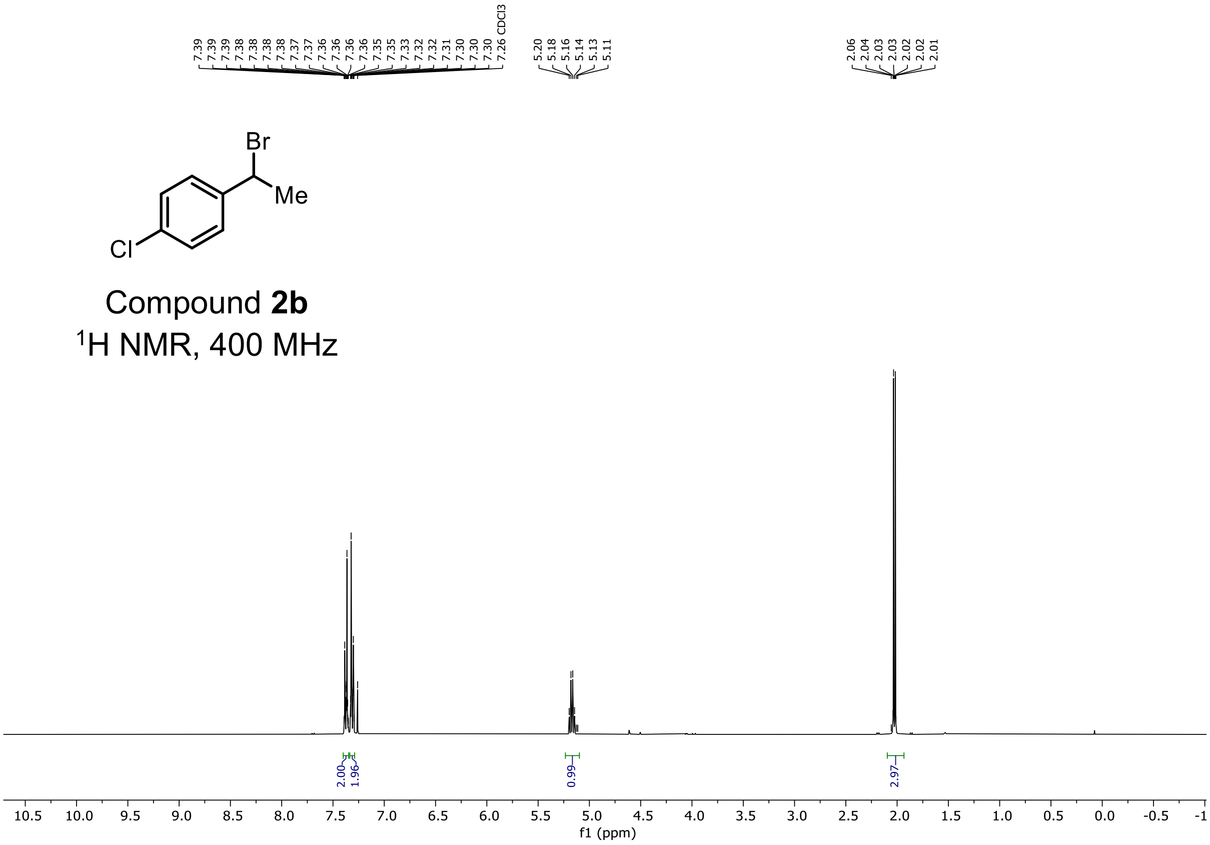

Compound **2b**
^{1}H NMR, 400 MHz

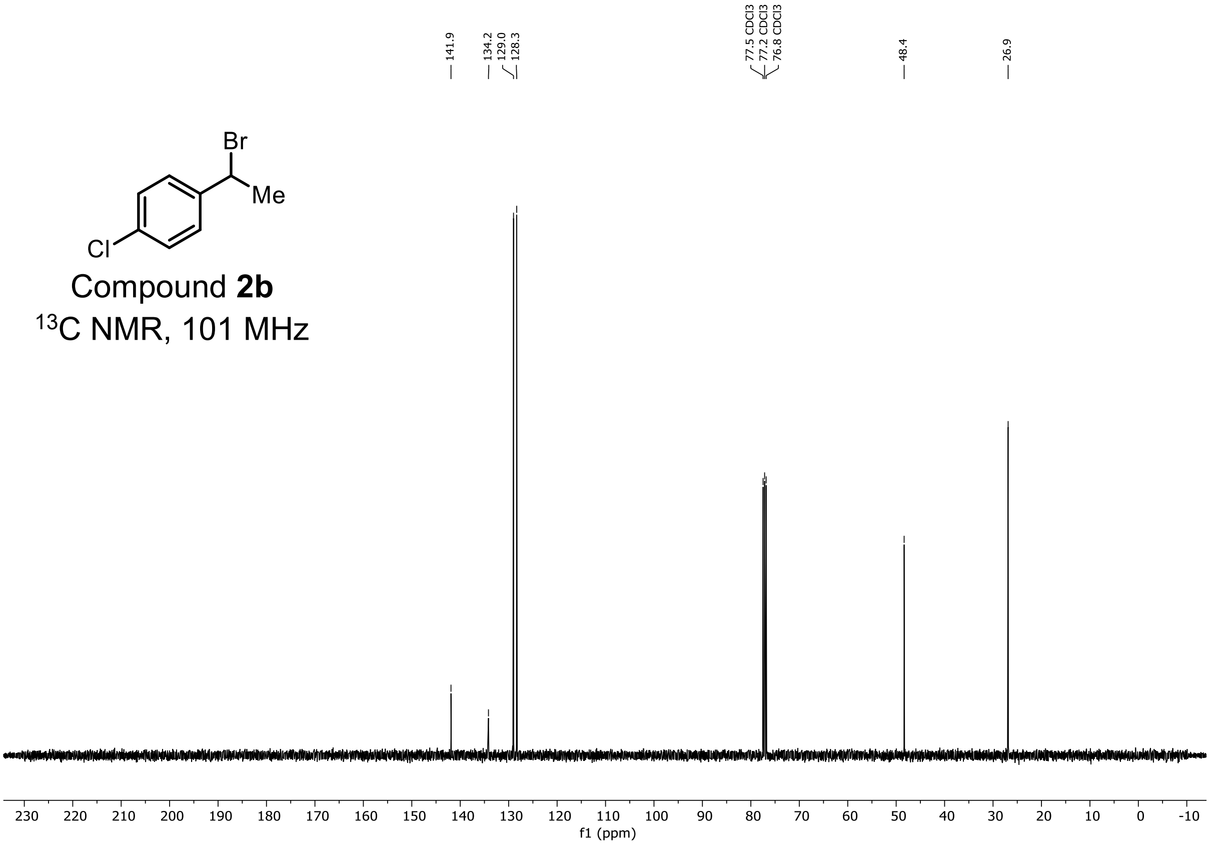

Compound **2b**
^{13}C NMR, 101 MHz

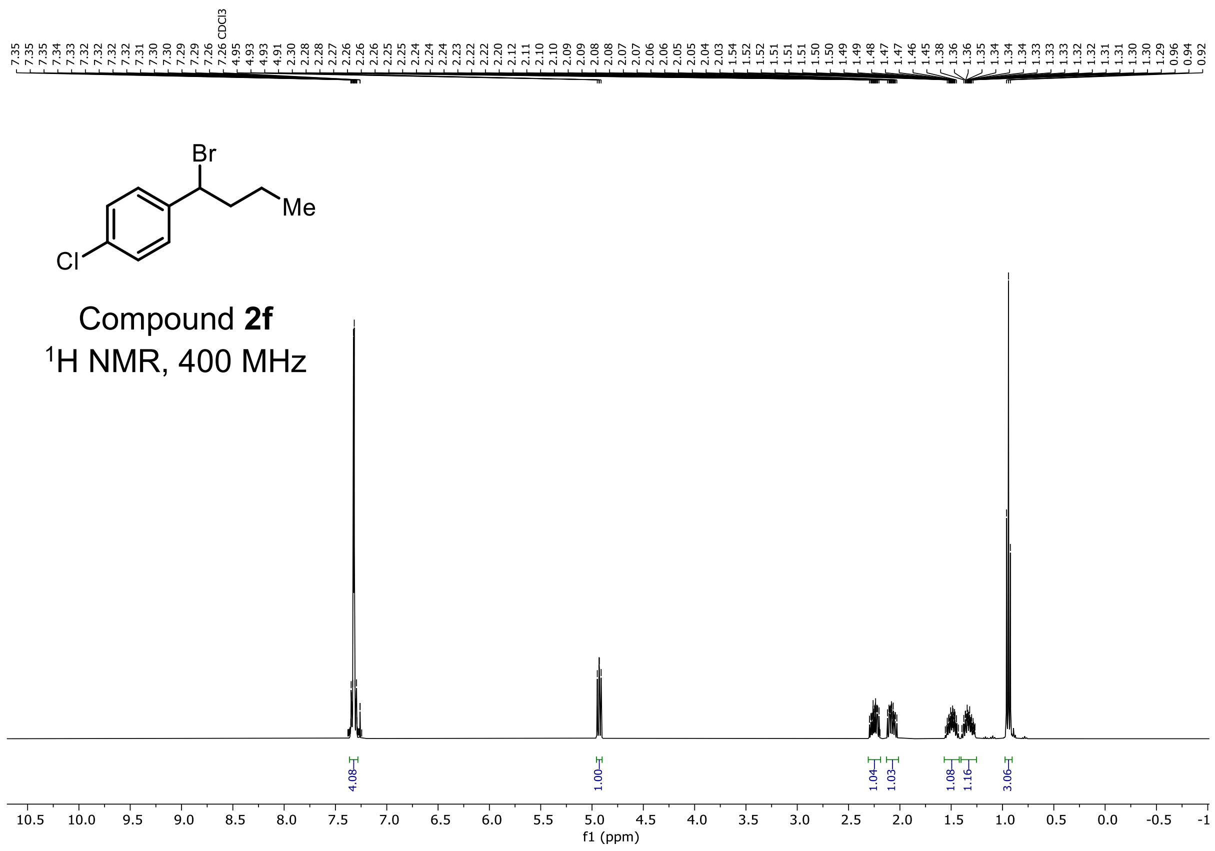
7.35 7.35 7.34 7.33 7.32 7.32 7.32 7.31 7.30 7.29 7.29 7.26 7.26 CDCl3 4.95 4.93 4.93 4.91 2.30 2.28 2.28 2.27 2.26 2.26 2.26 2.25 2.25 2.24 2.24 2.24 2.23 2.22 2.22 2.20 2.12 2.11 2.10 2.10 2.09 2.09 2.08 2.08 2.07 2.07 2.06 2.06 2.05 2.05 2.04 2.03 1.54 1.52 1.52 1.51 1.51 1.51 1.50 1.50 1.49 1.49 1.48 1.47 1.47 1.46 1.45 1.38 1.36 1.36 1.35 1.34 1.34 1.34 1.33 1.33 1.33 1.32 1.32 1.31 1.31 1.30 1.30 1.29 0.96 0.94 0.92
Br
Me
Cl
Compound **2f**
^{1}H NMR, 400 MHz
4.08
1.00
1.04
1.03
1.08
1.16
3.06
10.5 10.0 9.5 9.0 8.5 8.0 7.5 7.0 6.5 6.0 5.5 5.0 4.5 4.0 3.5 3.0 2.5 2.0 1.5 1.0 0.5 0.0 -0.5 -1
f1 (ppm)

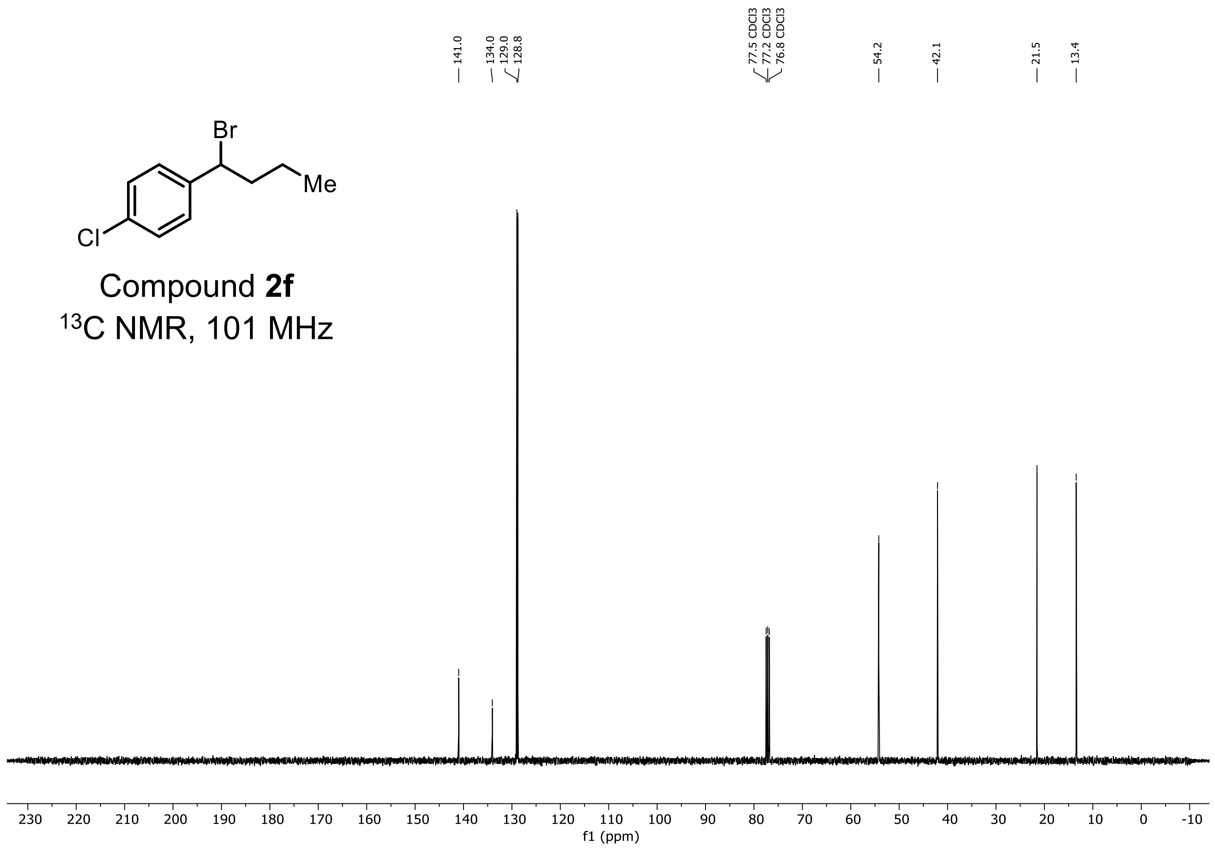
141.0
134.0
129.0
128.8
77.5 CDCl3
77.2 CDCl3
76.8 CDCl3
54.2
42.1
21.5
13.4
Br
Me
Cl
Compound **2f**
^{13}C NMR, 101 MHz
230 220 210 200 190 180 170 160 150 140 130 120 110 100 90 80 70 60 50 40 30 20 10 0 -10
f1 (ppm)

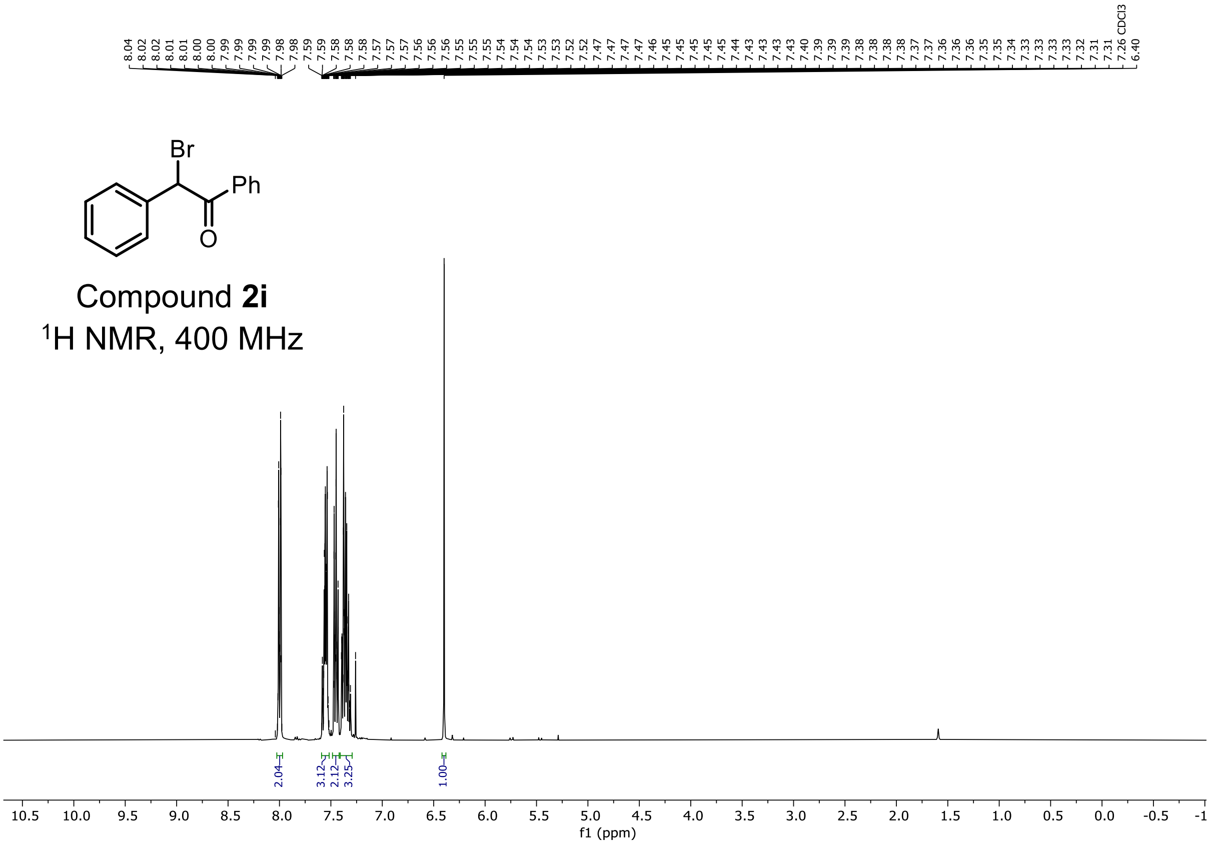
8.04 8.02 8.02 8.01 8.01 8.00 8.00 7.99 7.99 7.99 7.99 7.98 7.98 7.59 7.59 7.58 7.58 7.58 7.57 7.57 7.56 7.56 7.56 7.55 7.55 7.55 7.54 7.54 7.54 7.53 7.53 7.52 7.52 7.47 7.47 7.47 7.46 7.45 7.45 7.45 7.45 7.44 7.43 7.43 7.43 7.43 7.40 7.39 7.39 7.38 7.38 7.38 7.37 7.37 7.36 7.36 7.35 7.35 7.34 7.33 7.33 7.33 7.33 7.32 7.31 7.26 CDCl3 6.40
Br
Ph
O
Compound **2i**
^{1}H NMR, 400 MHz
2.04
3.12
2.12
3.25
1.00
10.5 10.0 9.5 9.0 8.5 8.0 7.5 7.0 6.5 6.0 5.5 5.0 4.5 4.0 3.5 3.0 2.5 2.0 1.5 1.0 0.5 0.0 -0.5 -1
f1 (ppm)

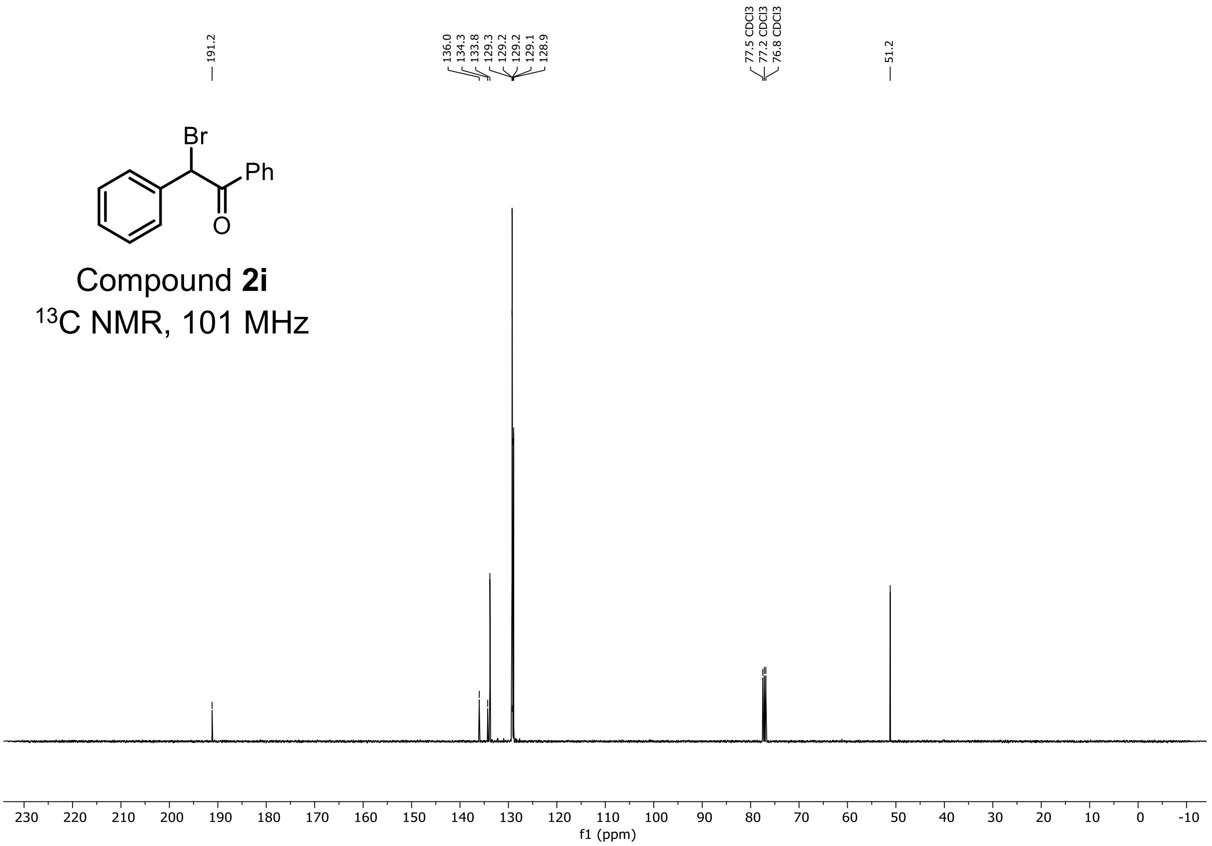
191.2
136.0 134.3 133.8 129.3 129.2 129.2 129.1 128.9
77.5 CDCl3 77.2 CDCl3 76.8 CDCl3
51.2
Br
Ph
O
Compound **2i**
^{13}C NMR, 101 MHz
230 220 210 200 190 180 170 160 150 140 130 120 110 100 90 80 70 60 50 40 30 20 10 0 -10
f1 (ppm)

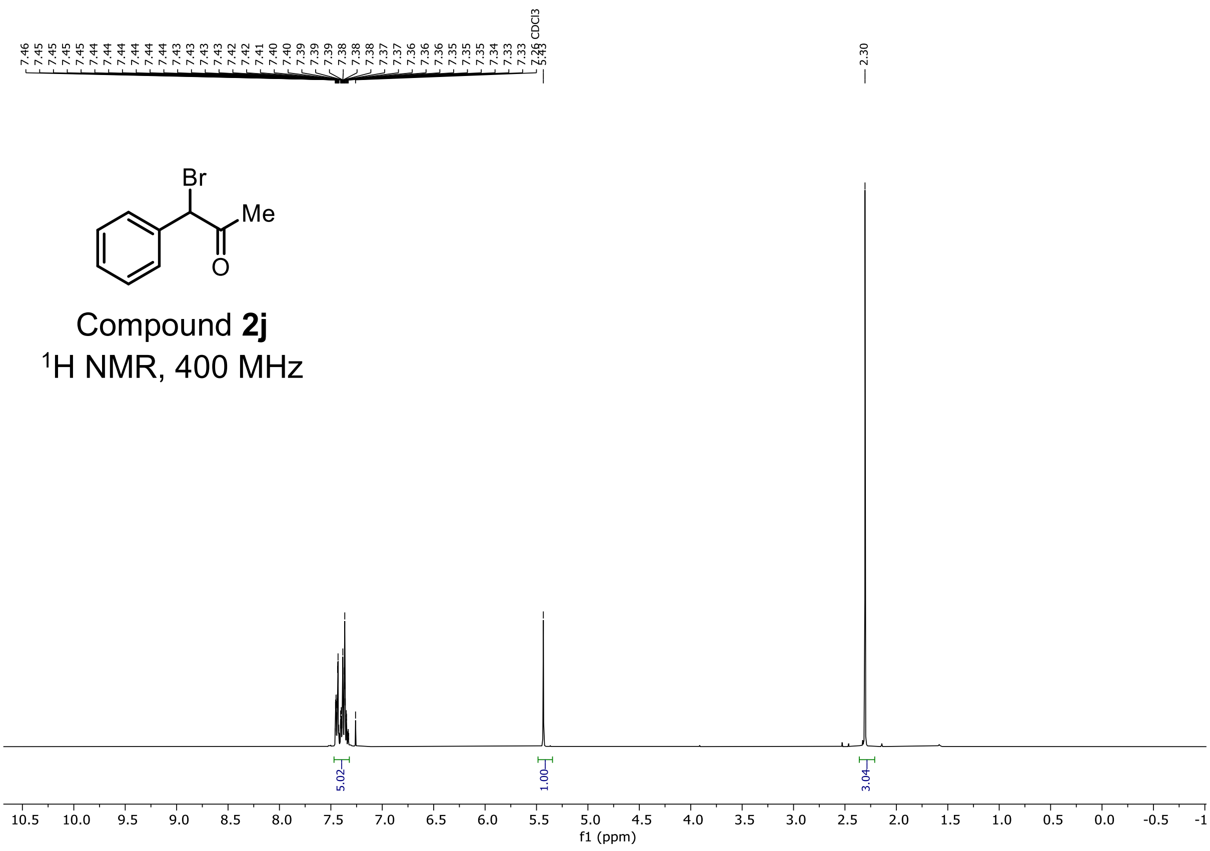

Compound **2j**
^{1}H NMR, 400 MHz

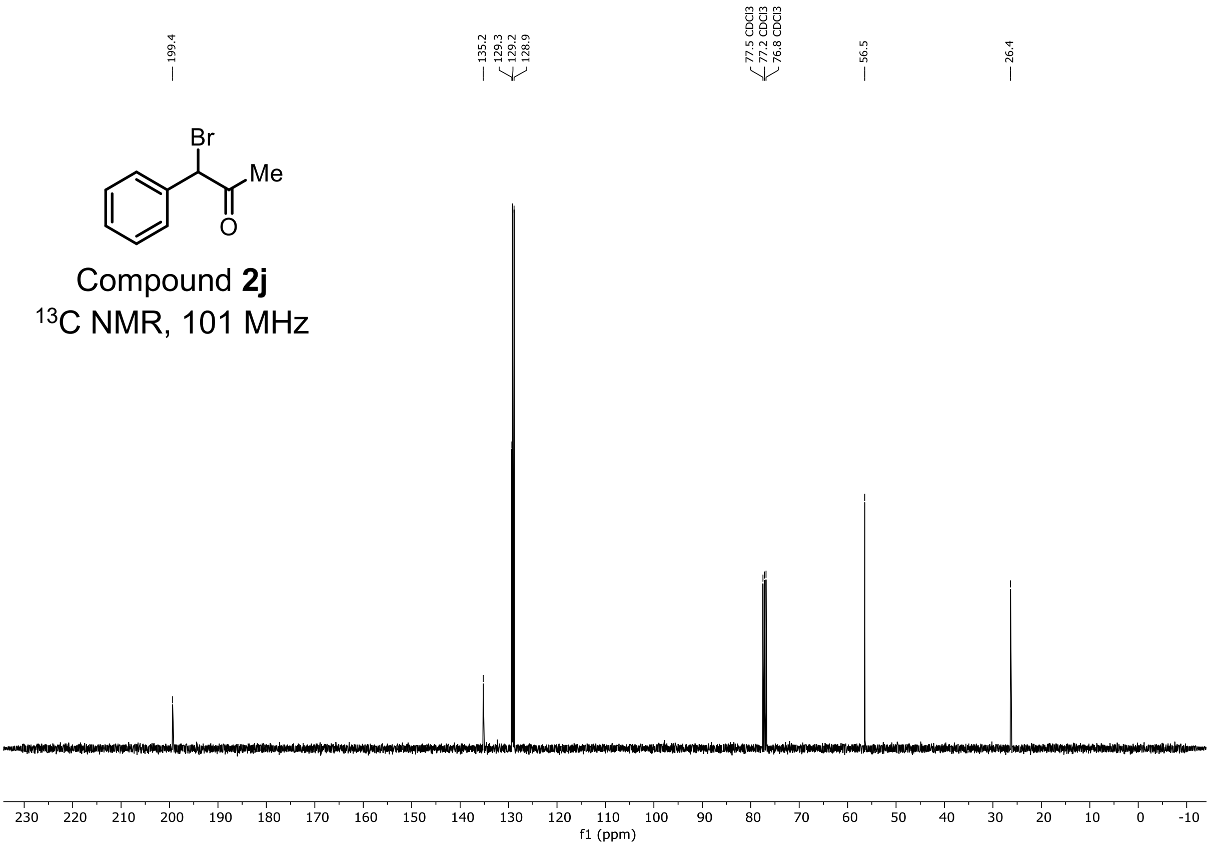

Compound **2j**
^{13}C NMR, 101 MHz

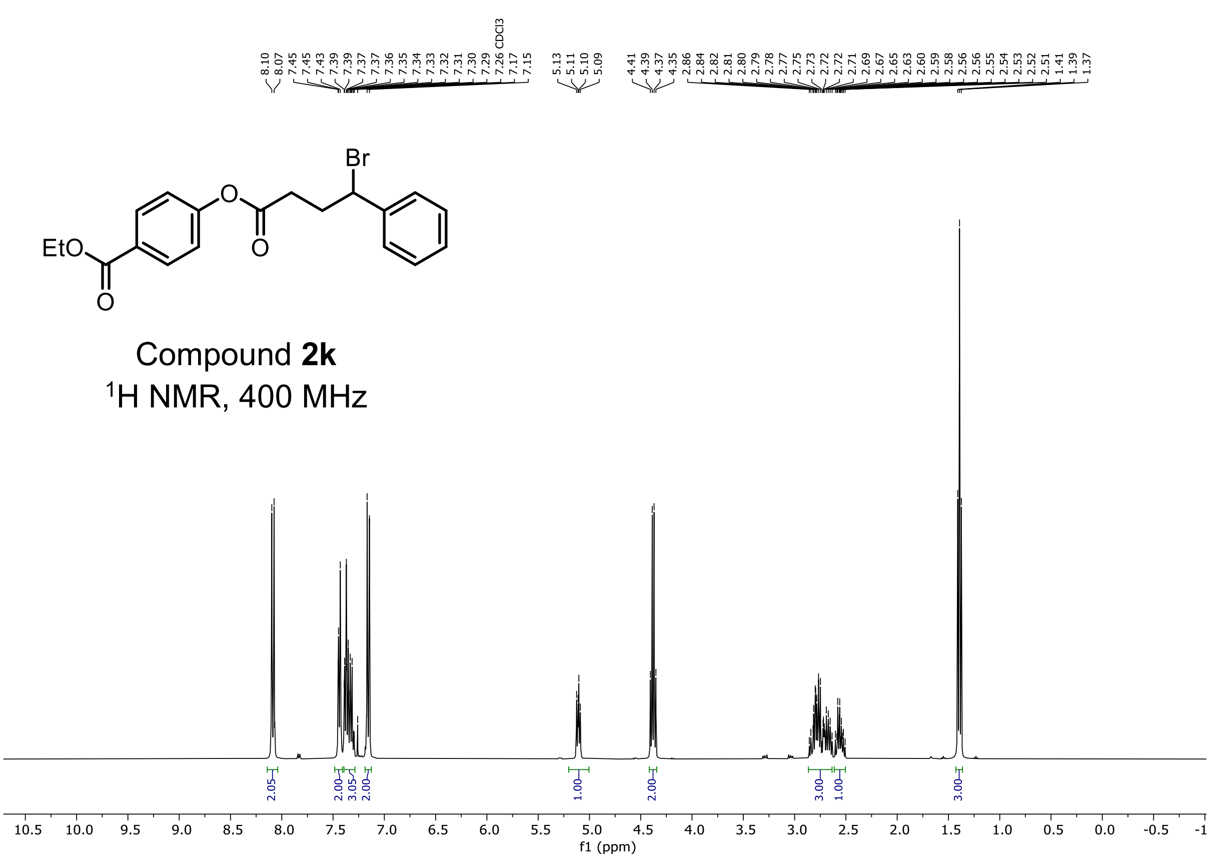
Compound **2k**
^{1}H NMR, 400 MHz
f1 (ppm)

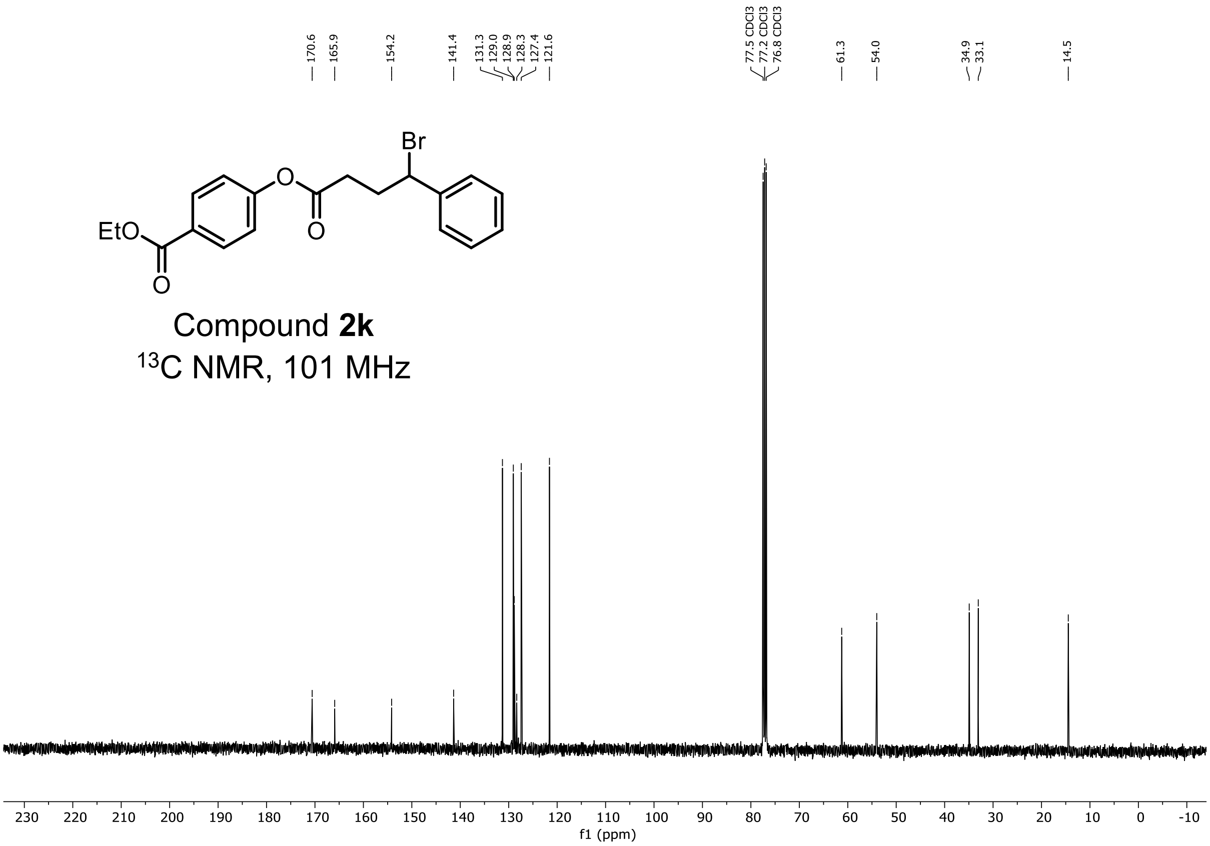
Compound **2k**
^{13}C NMR, 101 MHz
f1 (ppm)

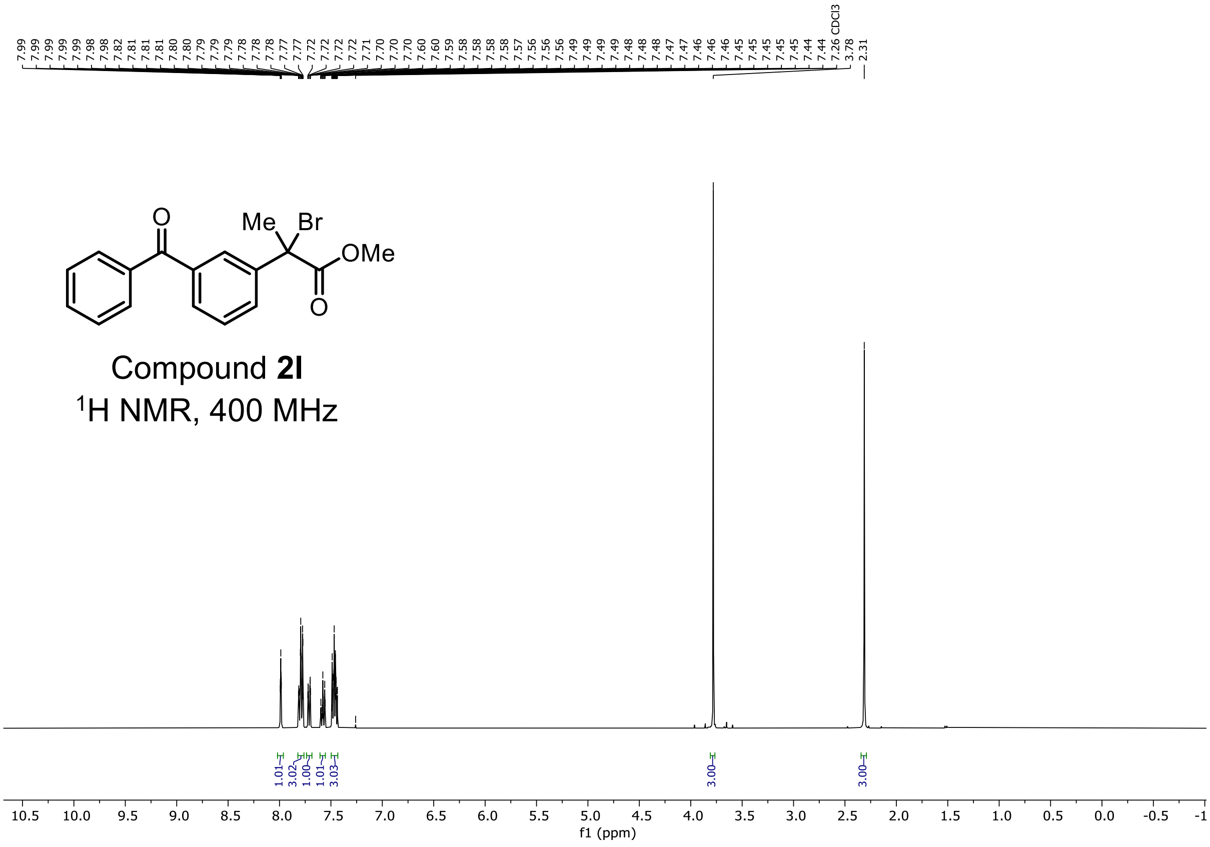
7.99 7.99 7.99 7.99 7.98 7.98 7.82 7.81 7.81 7.80 7.80 7.79 7.79 7.79 7.78 7.78 7.78 7.77 7.77 7.72 7.72 7.72 7.72 7.71 7.70 7.70 7.60 7.60 7.59 7.58 7.58 7.58 7.58 7.57 7.56 7.56 7.56 7.49 7.49 7.49 7.48 7.48 7.47 7.47 7.46 7.46 7.45 7.45 7.45 7.45 7.44 7.26 CDCl3 3.78 2.31
Me
Br
OMe
O
Compound **2l**
^{1}H NMR, 400 MHz
1.01 3.02 1.00 1.01 3.03 3.00 3.00
f1 (ppm)

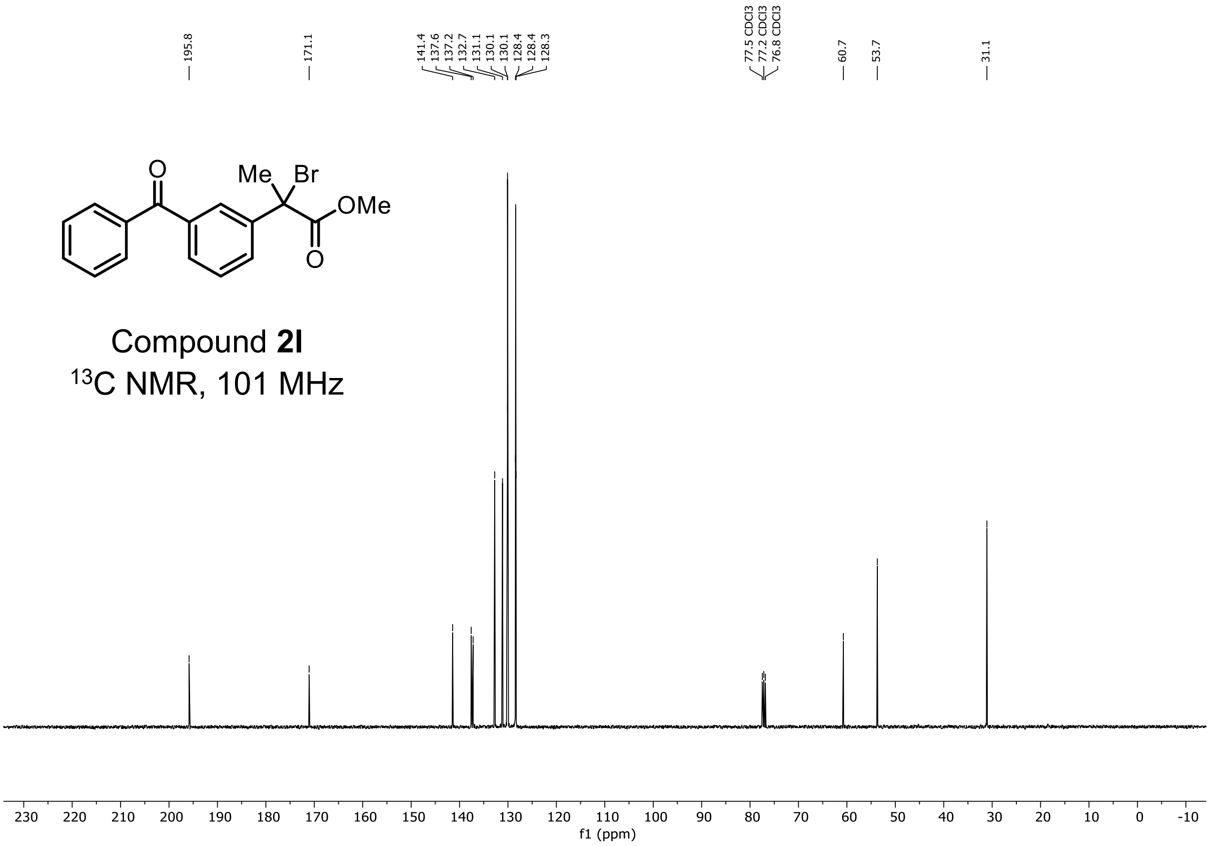
195.8 171.1 141.4 137.6 137.2 132.7 131.1 130.1 130.1 128.4 128.4 128.3 77.5 CDCl3 77.2 CDCl3 76.8 CDCl3 60.7 53.7 31.1
Me
Br
OMe
O
Compound **2l**
^{13}C NMR, 101 MHz
f1 (ppm)

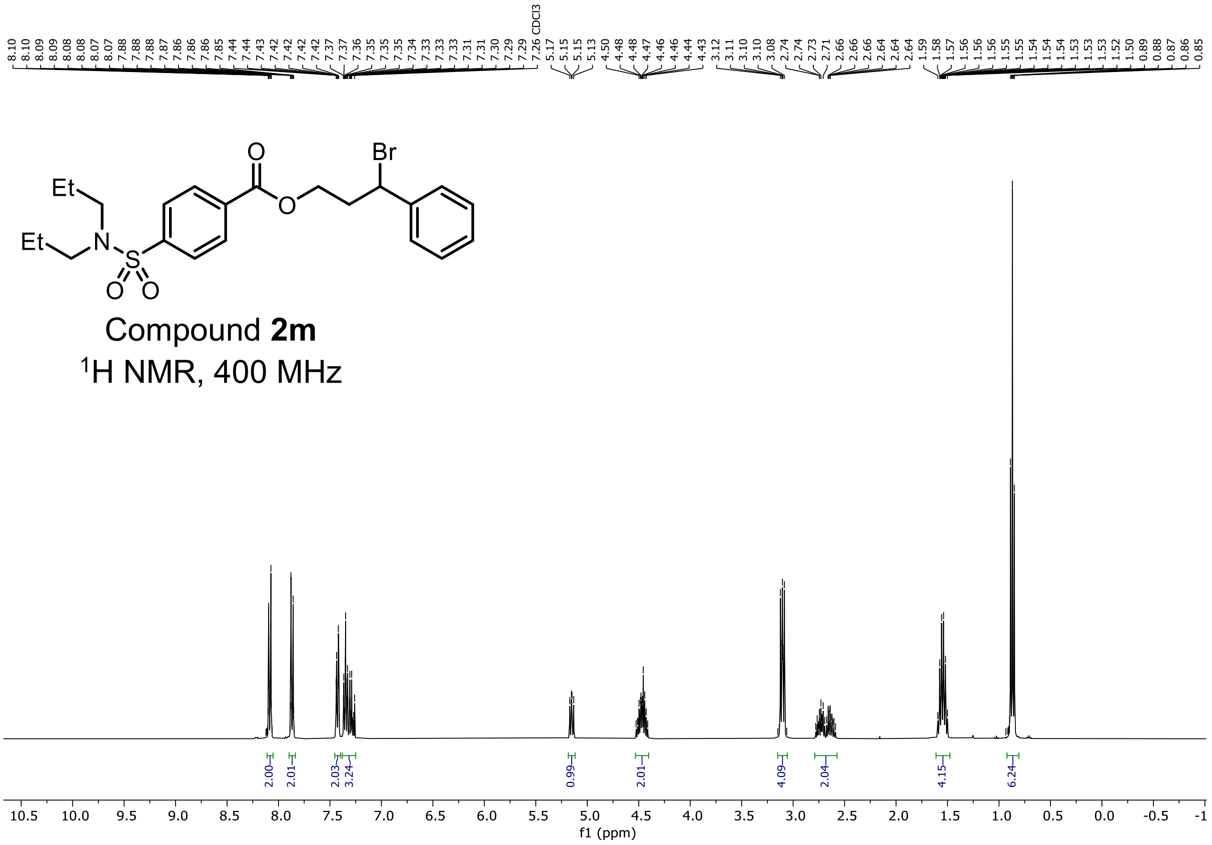
8.10 8.10 8.09 8.09 8.08 8.08 8.07 7.88 7.88 7.88 7.87 7.86 7.86 7.86 7.85 7.44 7.43 7.42 7.42 7.42 7.42 7.37 7.36 7.35 7.35 7.35 7.34 7.33 7.33 7.33 7.31 7.31 7.30 7.29 7.29 7.26 CDCl3 5.17 5.15 5.15 5.13 4.50 4.48 4.48 4.47 4.46 4.46 4.44 4.43 3.12 3.11 3.10 3.10 3.08 2.74 2.74 2.73 2.71 2.66 2.66 2.66 2.64 2.64 2.64 1.59 1.58 1.57 1.56 1.56 1.55 1.55 1.54 1.54 1.54 1.53 1.53 1.53 1.52 1.50 0.89 0.88 0.87 0.86 0.85
Et
Et
N
S
O
O
O
O
Br
Compound **2m**
^{1}H NMR, 400 MHz
2.00 2.01 2.03 3.24 0.99 2.01 4.09 2.04 4.15 6.24
10.5 10.0 9.5 9.0 8.5 8.0 7.5 7.0 6.5 6.0 5.5 5.0 4.5 4.0 3.5 3.0 2.5 2.0 1.5 1.0 0.5 0.0 -0.5 -1
f1 (ppm)

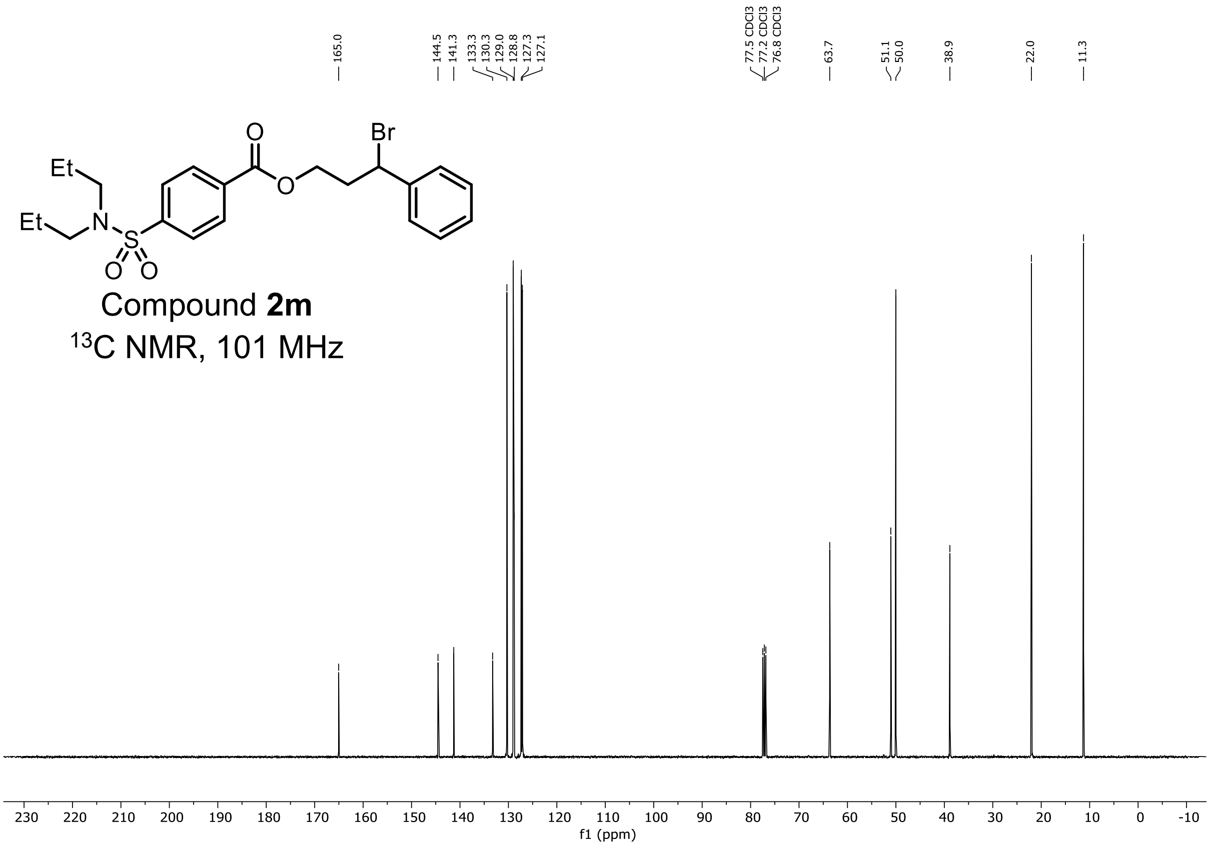
165.0 144.5 141.3 133.3 130.3 129.0 128.8 127.3 127.1 77.5 CDCl3 77.2 CDCl3 76.8 CDCl3 63.7 51.1 50.0 38.9 22.0 11.3
Et
Et
N
S
O
O
O
O
Br
Compound **2m**
^{13}C NMR, 101 MHz
230 220 210 200 190 180 170 160 150 140 130 120 110 100 90 80 70 60 50 40 30 20 10 0 -10
f1 (ppm)

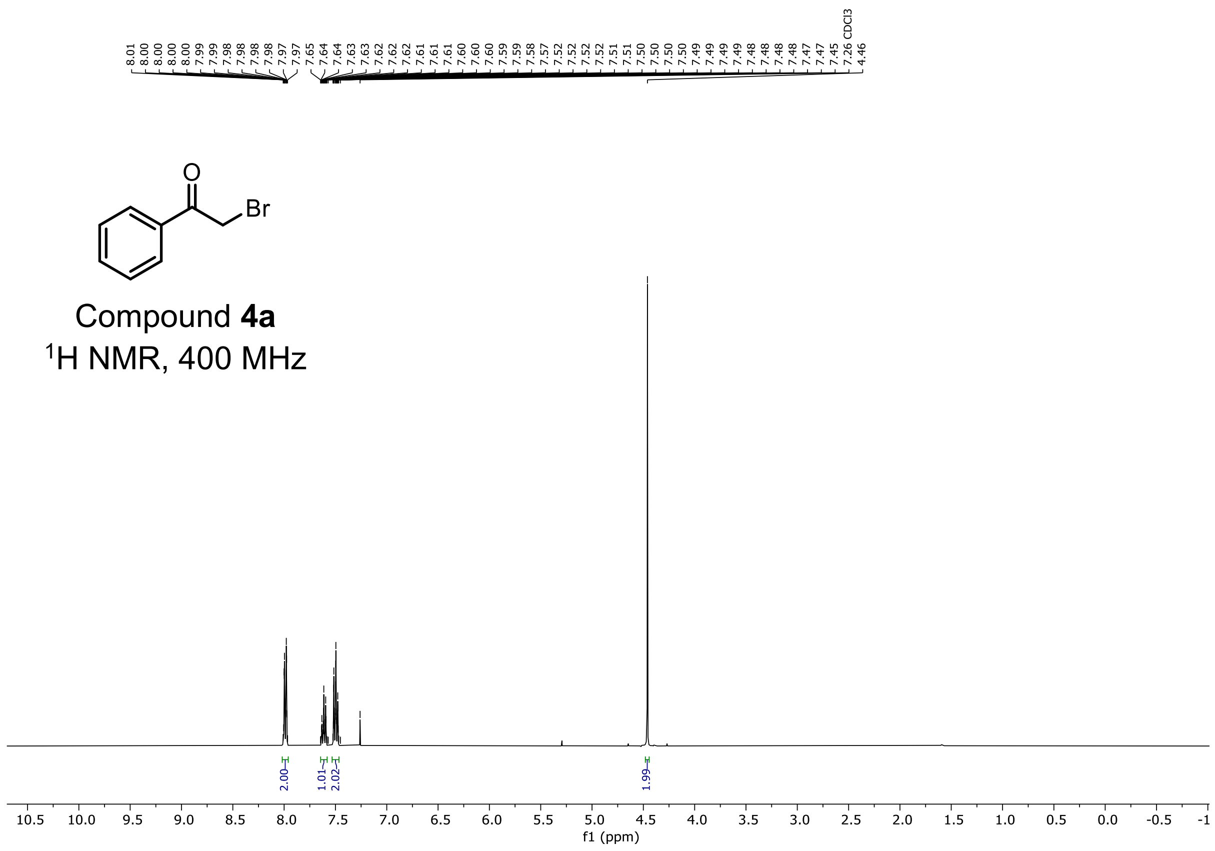

8.01 8.00 8.00 8.00 8.00 7.99 7.99 7.98 7.98 7.98 7.98 7.97 7.97 7.65 7.64 7.64 7.63 7.63 7.62 7.62 7.62 7.61 7.61 7.61 7.60 7.60 7.59 7.59 7.58 7.57 7.52 7.52 7.52 7.52 7.51 7.51 7.50 7.50 7.50 7.50 7.49 7.49 7.49 7.49 7.48 7.48 7.48 7.48 7.47 7.47 7.45 7.26 CDCl3 4.46
O
Br
Compound 4a
1H NMR, 400 MHz
2.00
1.01
2.02
1.99
10.5 10.0 9.5 9.0 8.5 8.0 7.5 7.0 6.5 6.0 5.5 5.0 4.5 4.0 3.5 3.0 2.5 2.0 1.5 1.0 0.5 0.0 -0.5 -1
f1 (ppm)

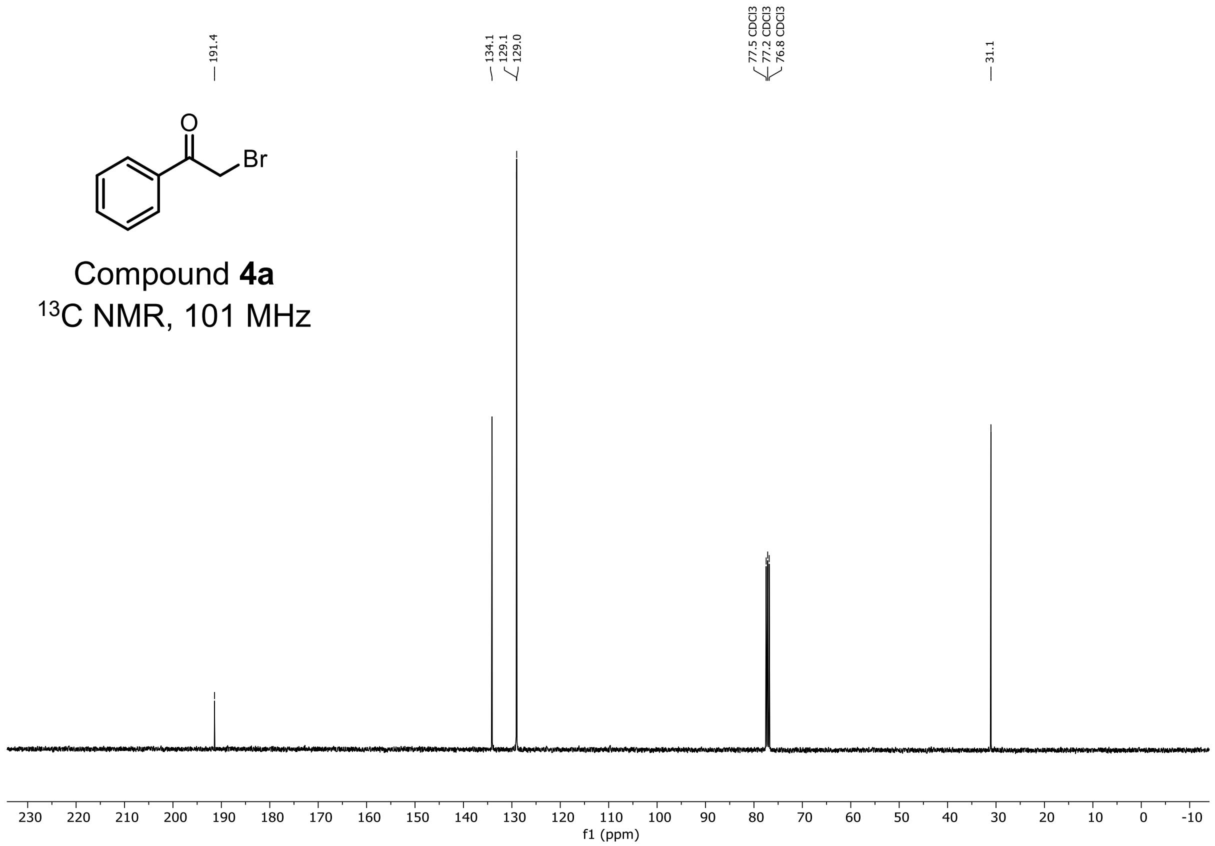

191.4
134.1 129.1 129.0
77.5 CDCl3 77.2 CDCl3 76.8 CDCl3
31.1
O
Br
Compound 4a
13C NMR, 101 MHz
230 220 210 200 190 180 170 160 150 140 130 120 110 100 90 80 70 60 50 40 30 20 10 0 -10
f1 (ppm)

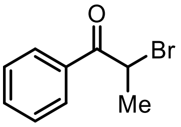

Compound **4b**
^{1}H NMR, 400 MHz

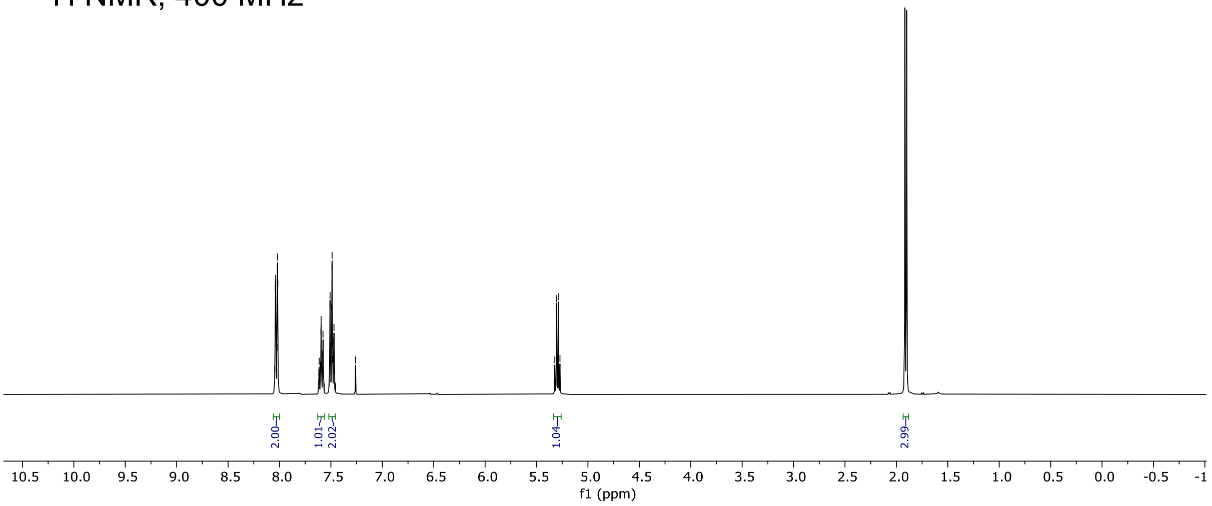

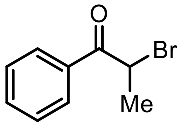

Compound **4b**
^{13}C NMR, 101 MHz

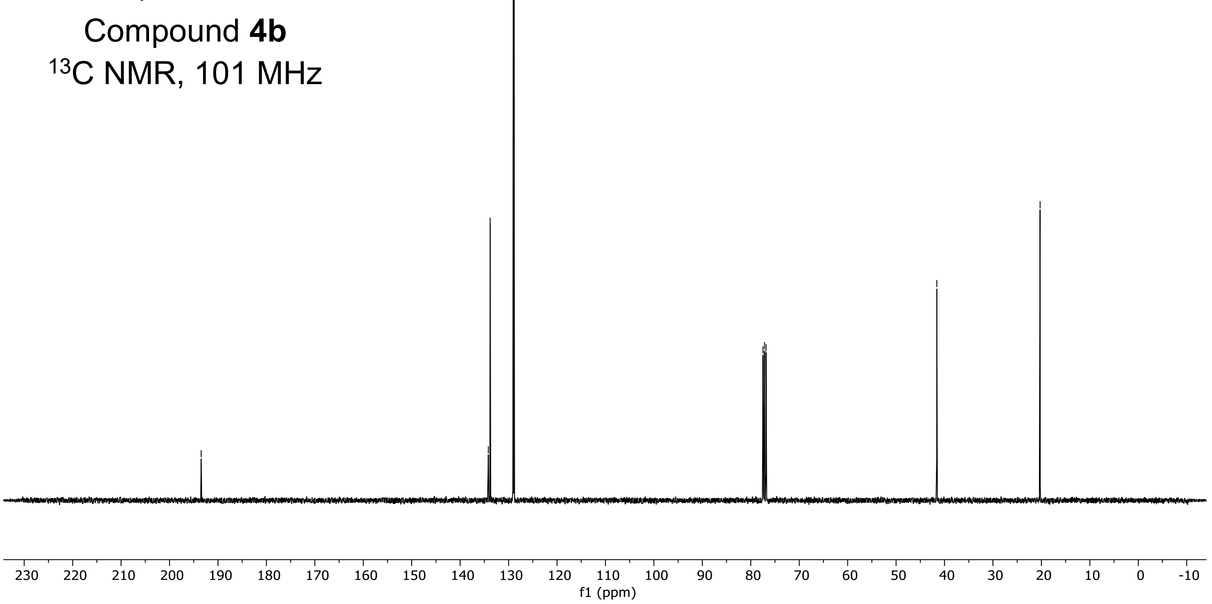

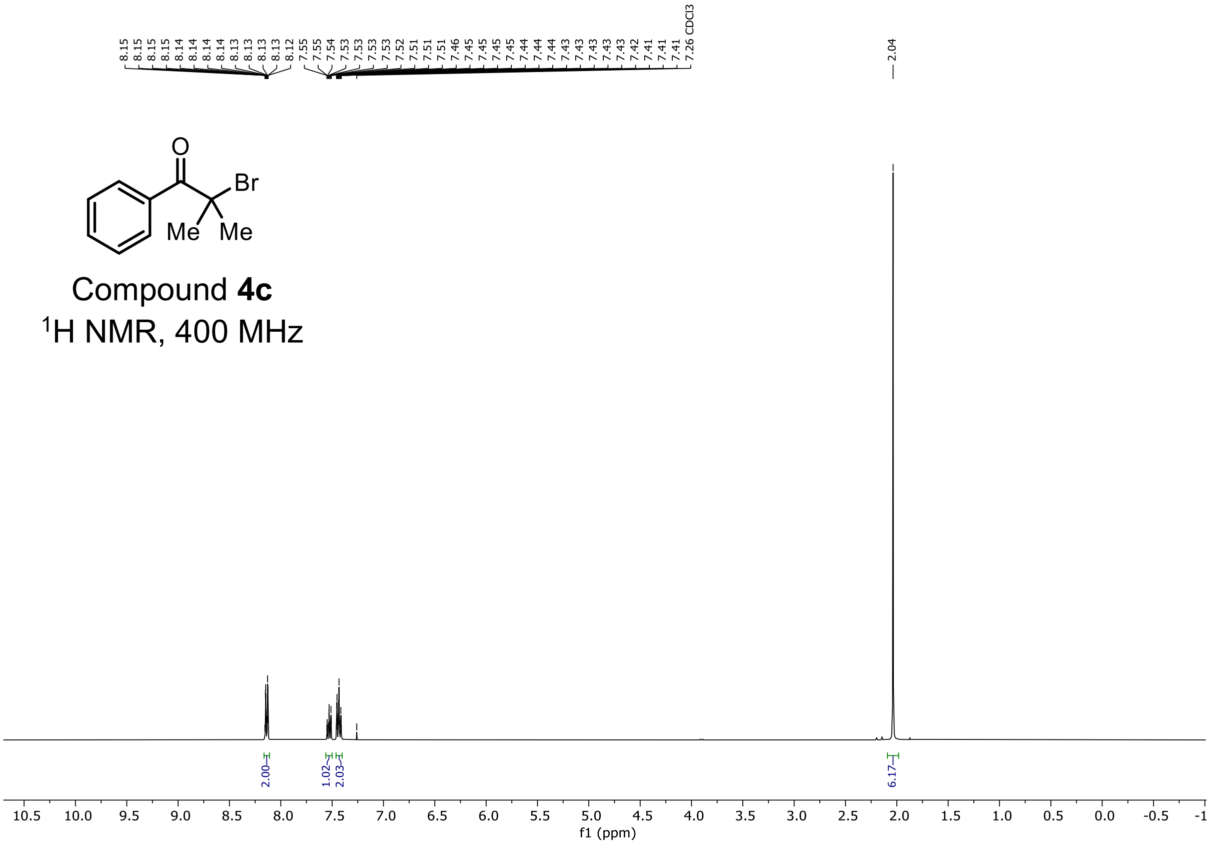

8.15
8.15
8.15
8.15
8.14
8.14
8.14
8.14
8.13
8.13
8.13
8.13
8.12
7.55
7.55
7.54
7.53
7.53
7.53
7.53
7.52
7.51
7.51
7.51
7.46
7.45
7.45
7.45
7.45
7.44
7.44
7.44
7.43
7.43
7.43
7.43
7.42
7.41
7.41
7.41
7.26 CDCl3
2.04
O
Br
Me Me
Compound **4c**
^{1}H NMR, 400 MHz
2.00
1.02
2.03
6.17
10.5 10.0 9.5 9.0 8.5 8.0 7.5 7.0 6.5 6.0 5.5 5.0 4.5 4.0 3.5 3.0 2.5 2.0 1.5 1.0 0.5 0.0 -0.5 -1
f1 (ppm)

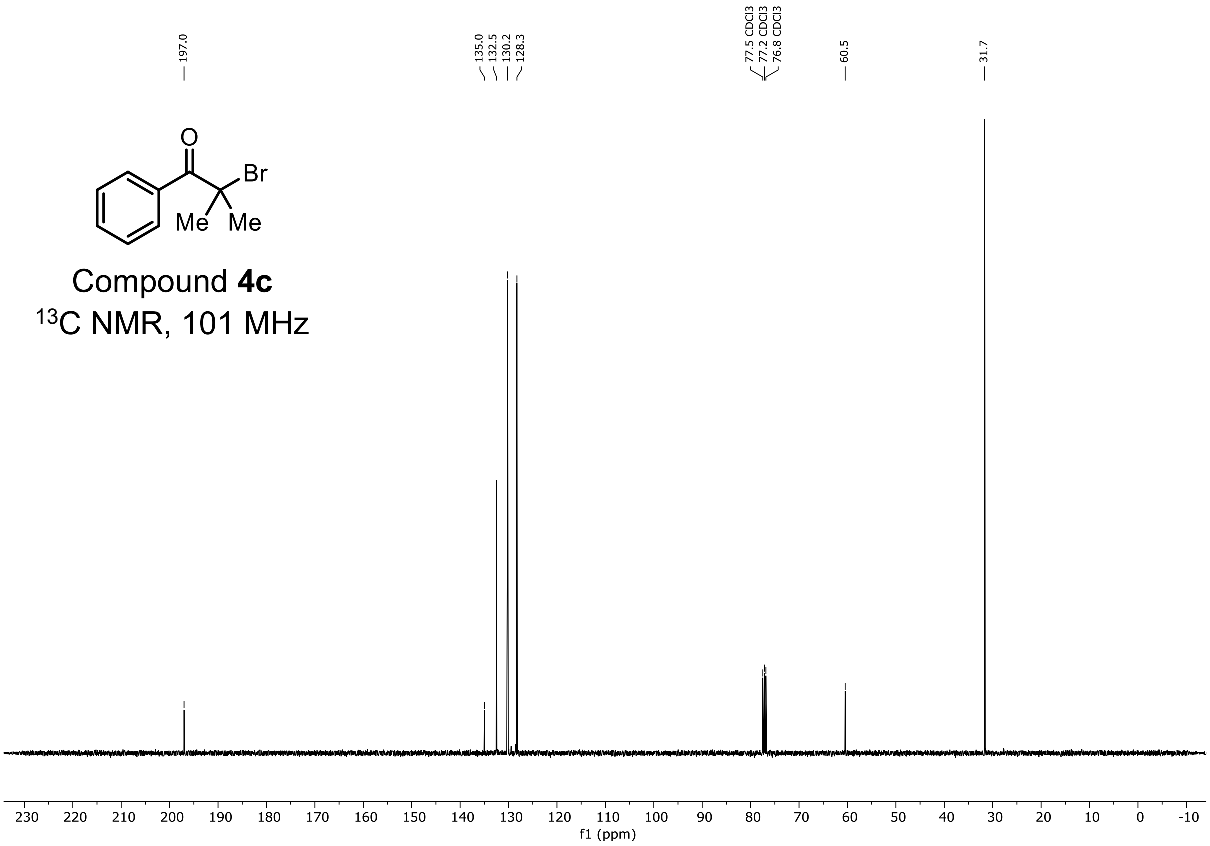

197.0
135.0
132.5
130.2
128.3
77.5 CDCl3
77.2 CDCl3
76.8 CDCl3
60.5
31.7
O
Br
Me Me
Compound **4c**
^{13}C NMR, 101 MHz
230 220 210 200 190 180 170 160 150 140 130 120 110 100 90 80 70 60 50 40 30 20 10 0 -10
f1 (ppm)

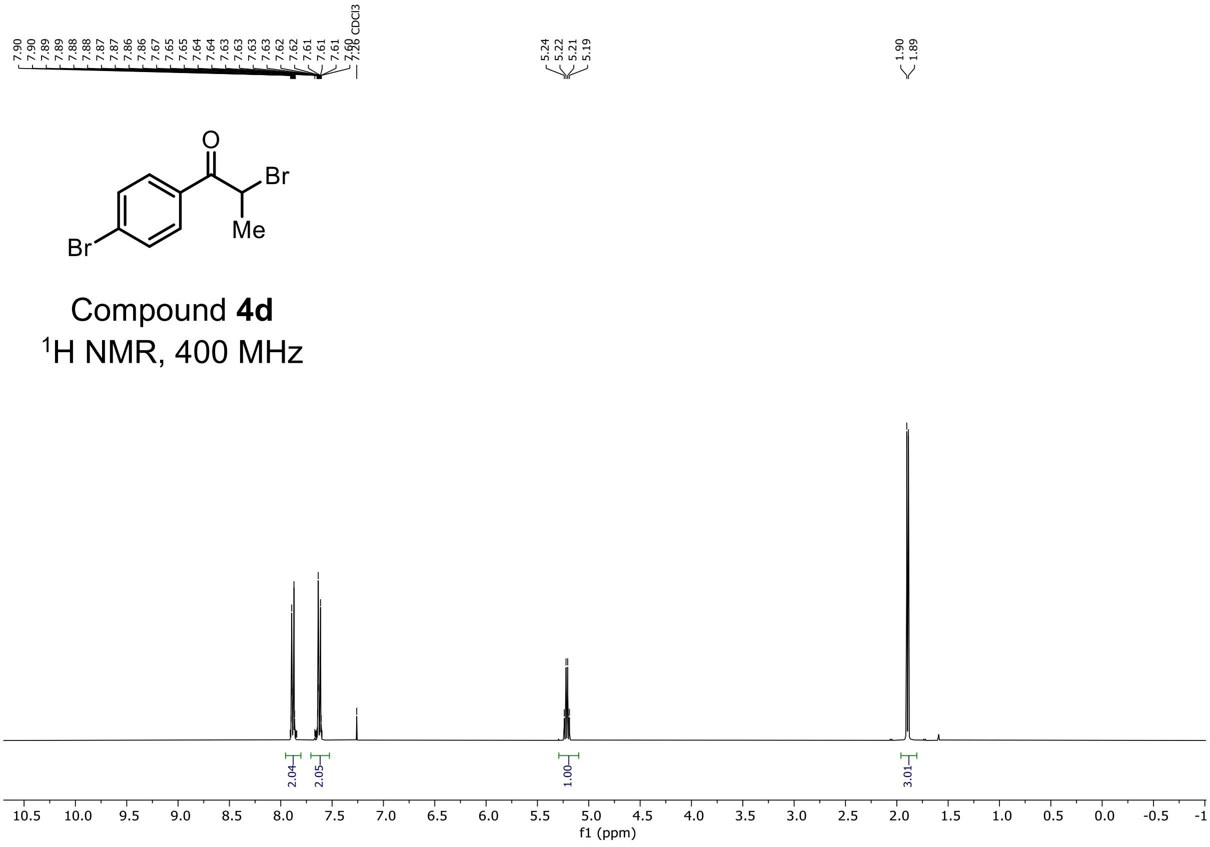

Compound **4d**
^{1}H NMR, 400 MHz

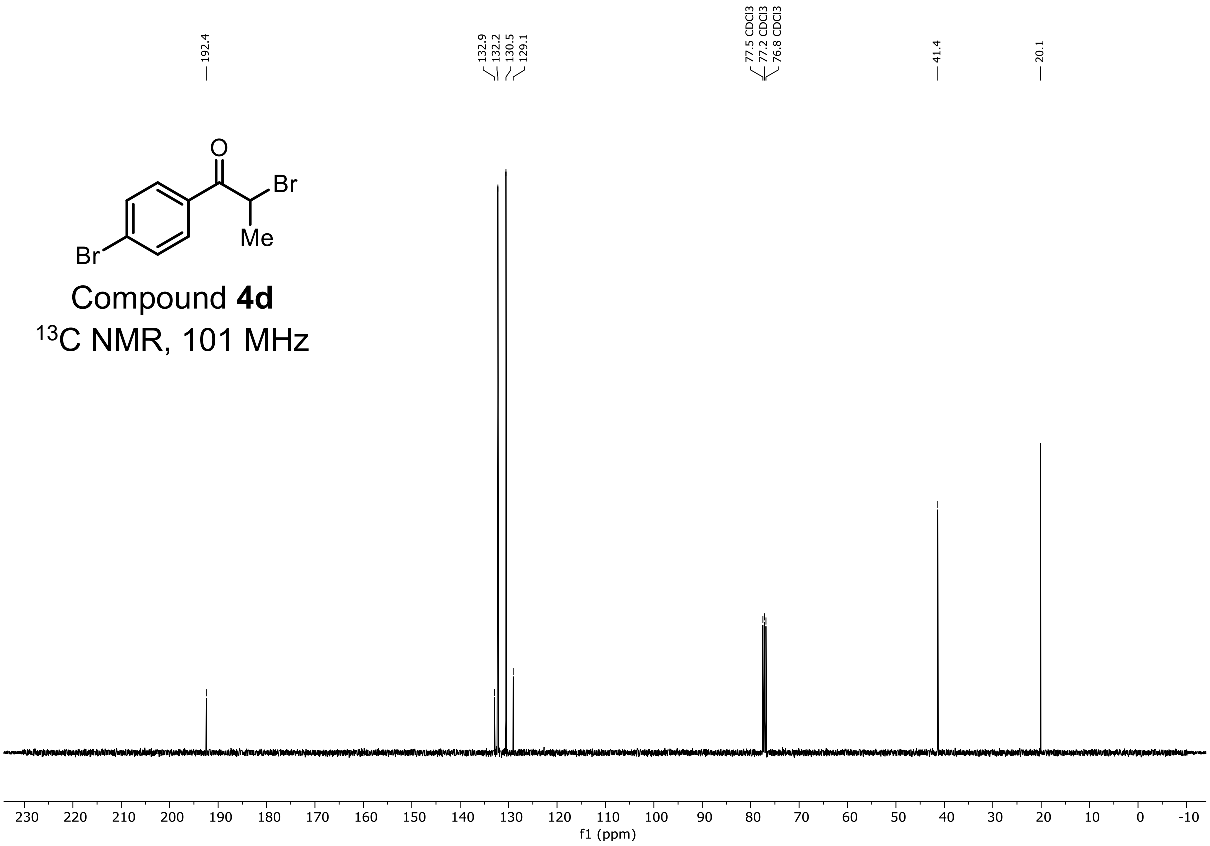

Compound **4d**
^{13}C NMR, 101 MHz

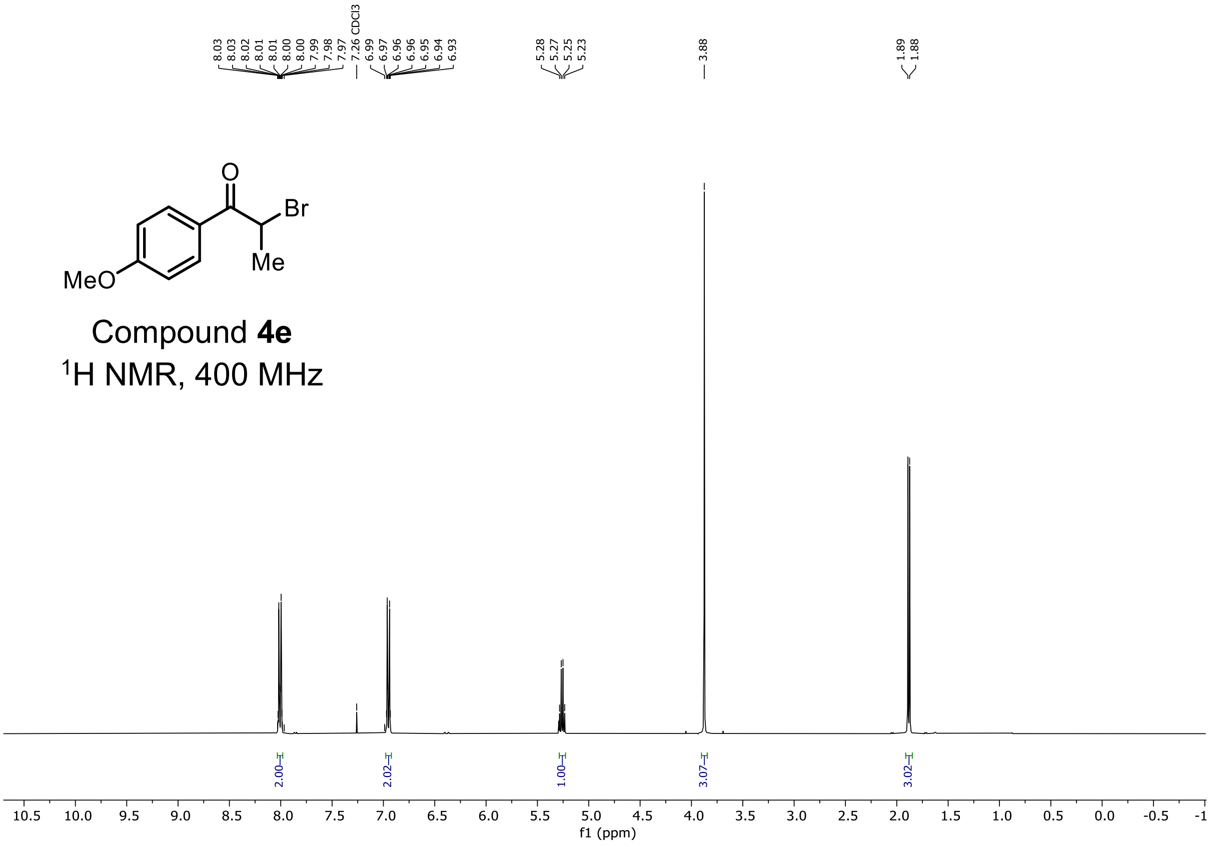
8.03
8.03
8.02
8.01
8.01
8.00
8.00
7.99
7.98
7.97
7.26 CDCl3
6.99
6.97
6.96
6.96
6.95
6.94
6.93
5.28
5.27
5.25
5.23
3.88
1.89
1.88
O
Br
Me
MeO
Compound 4e
1H NMR, 400 MHz
2.00
2.02
1.00
3.07
3.02
f1 (ppm)

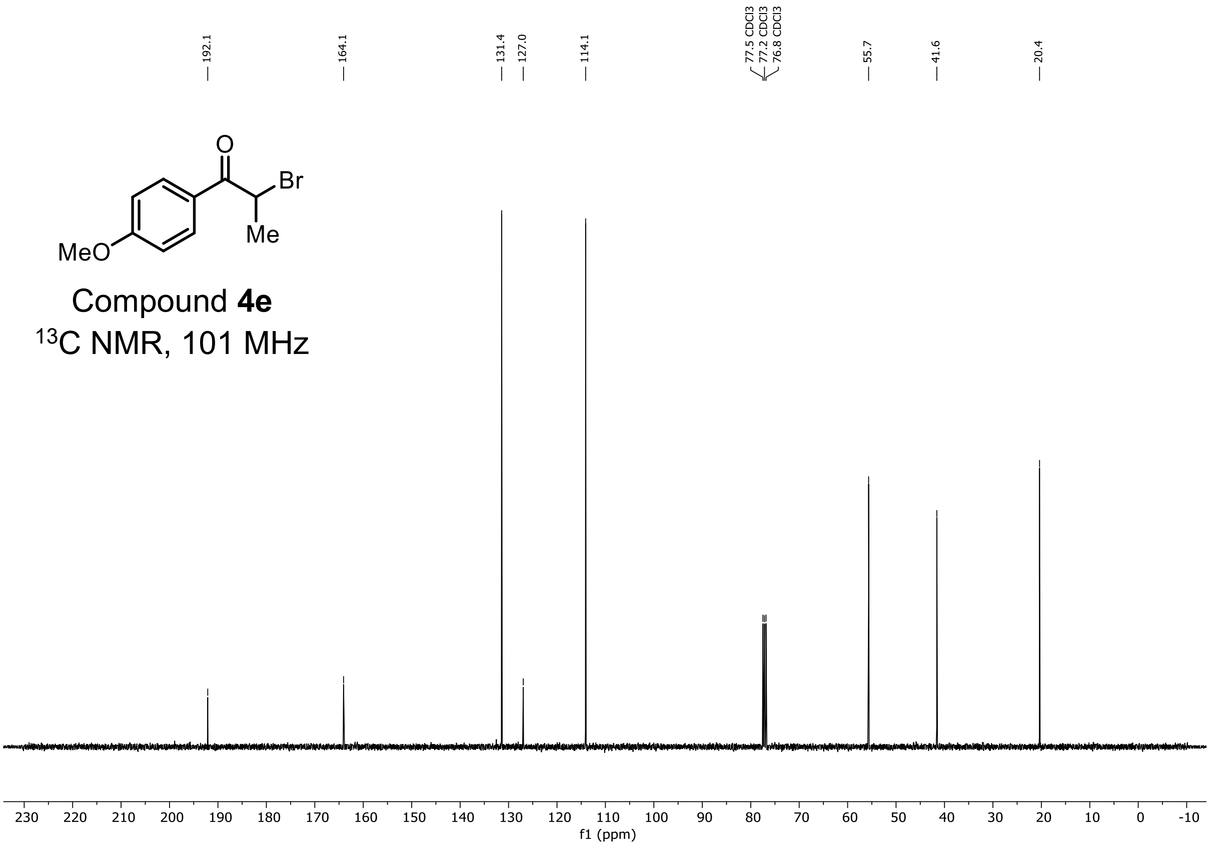
192.1
164.1
131.4
127.0
114.1
77.5 CDCl3
77.2 CDCl3
76.8 CDCl3
55.7
41.6
20.4
O
Br
Me
MeO
Compound 4e
13C NMR, 101 MHz
f1 (ppm)

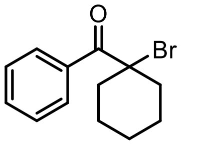

Compound **4f**
^{1}H NMR, 400 MHz

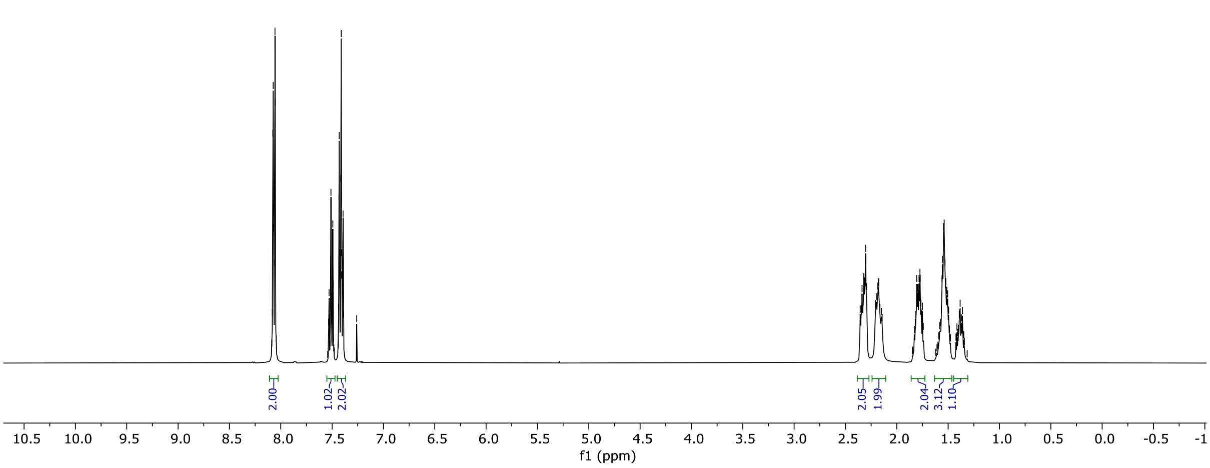

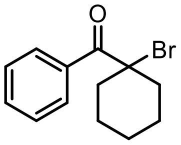

Compound **4f**
^{13}C NMR, 101 MHz

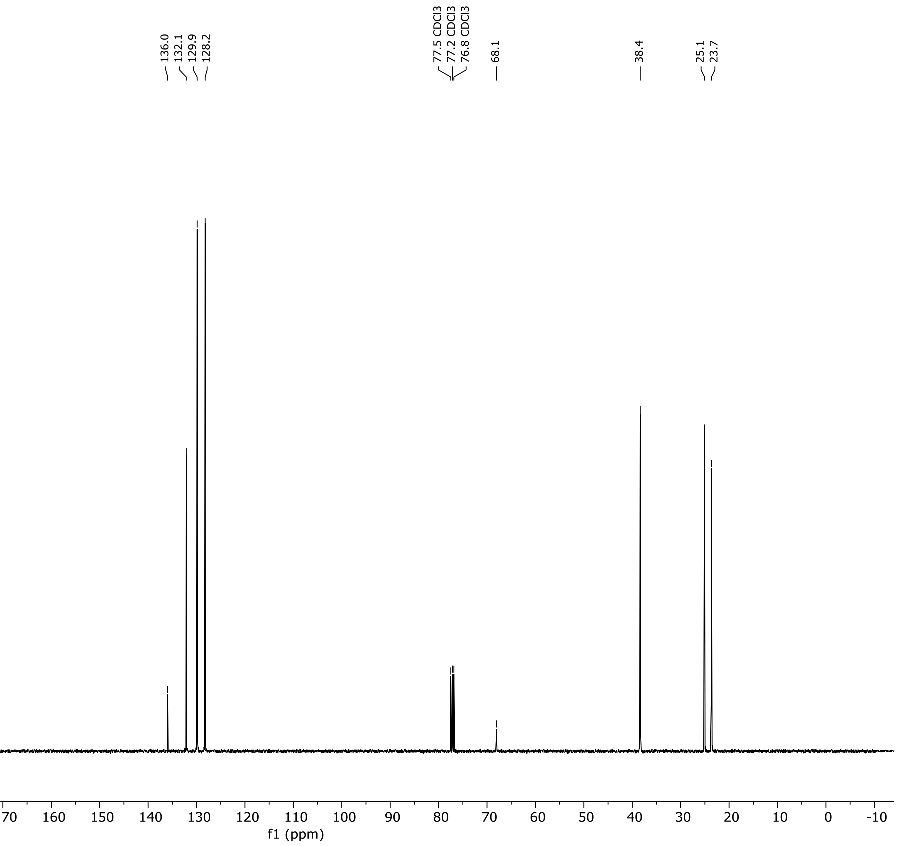

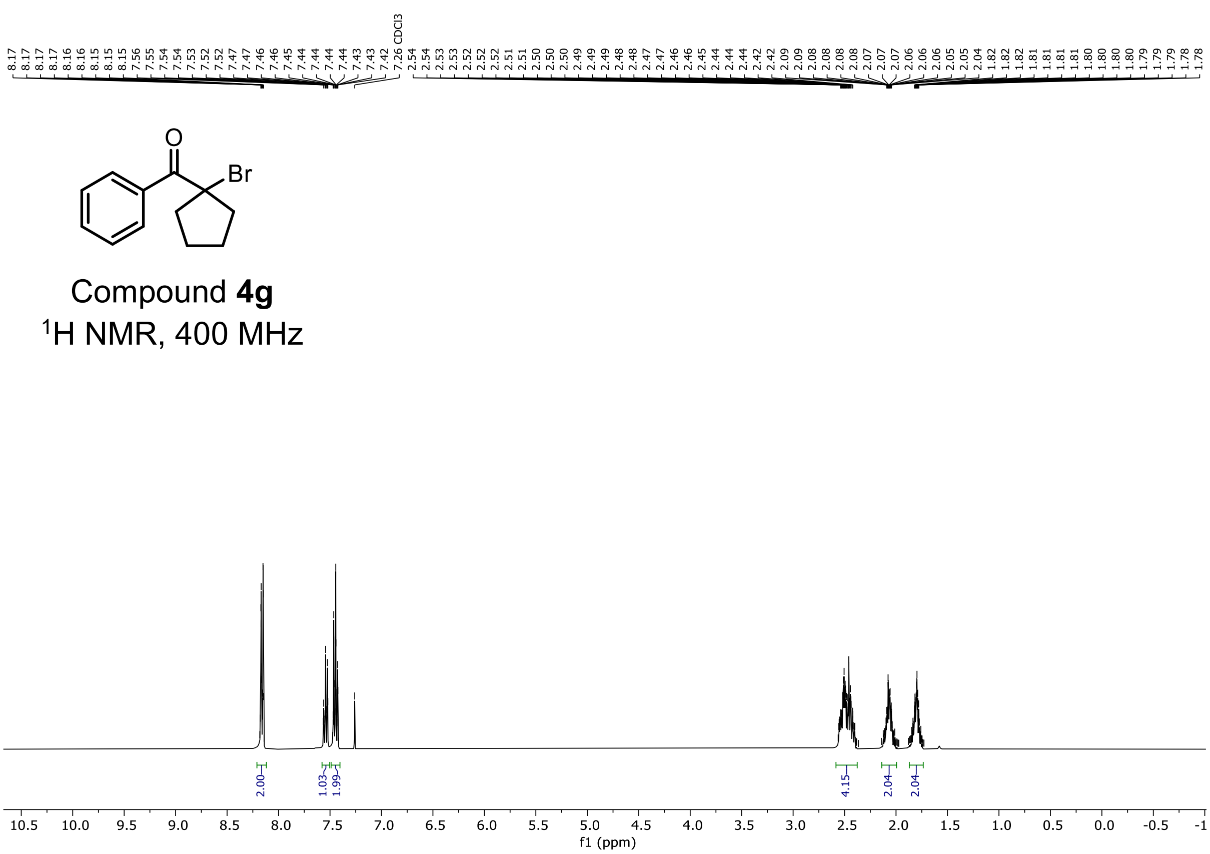
8.17 8.17 8.17 8.17 8.16 8.16 8.15 8.15 8.15 7.56 7.55 7.54 7.54 7.53 7.52 7.52 7.47 7.47 7.46 7.46 7.45 7.44 7.44 7.44 7.44 7.43 7.42 7.26 CDCl3 2.54 2.54 2.53 2.53 2.52 2.52 2.52 2.51 2.51 2.50 2.50 2.50 2.49 2.49 2.49 2.48 2.48 2.47 2.47 2.46 2.46 2.45 2.44 2.44 2.44 2.42 2.42 2.09 2.09 2.08 2.08 2.08 2.08 2.07 2.07 2.07 2.06 2.06 2.06 2.05 2.05 2.04 1.82 1.82 1.82 1.82 1.81 1.81 1.81 1.81 1.80 1.80 1.80 1.80 1.79 1.79 1.78 1.78
O
Br
Compound 4g
1H NMR, 400 MHz
2.00
1.03
1.99
4.15
2.04
2.04
10.5 10.0 9.5 9.0 8.5 8.0 7.5 7.0 6.5 6.0 5.5 5.0 4.5 4.0 3.5 3.0 2.5 2.0 1.5 1.0 0.5 0.0 -0.5 -1
f1 (ppm)

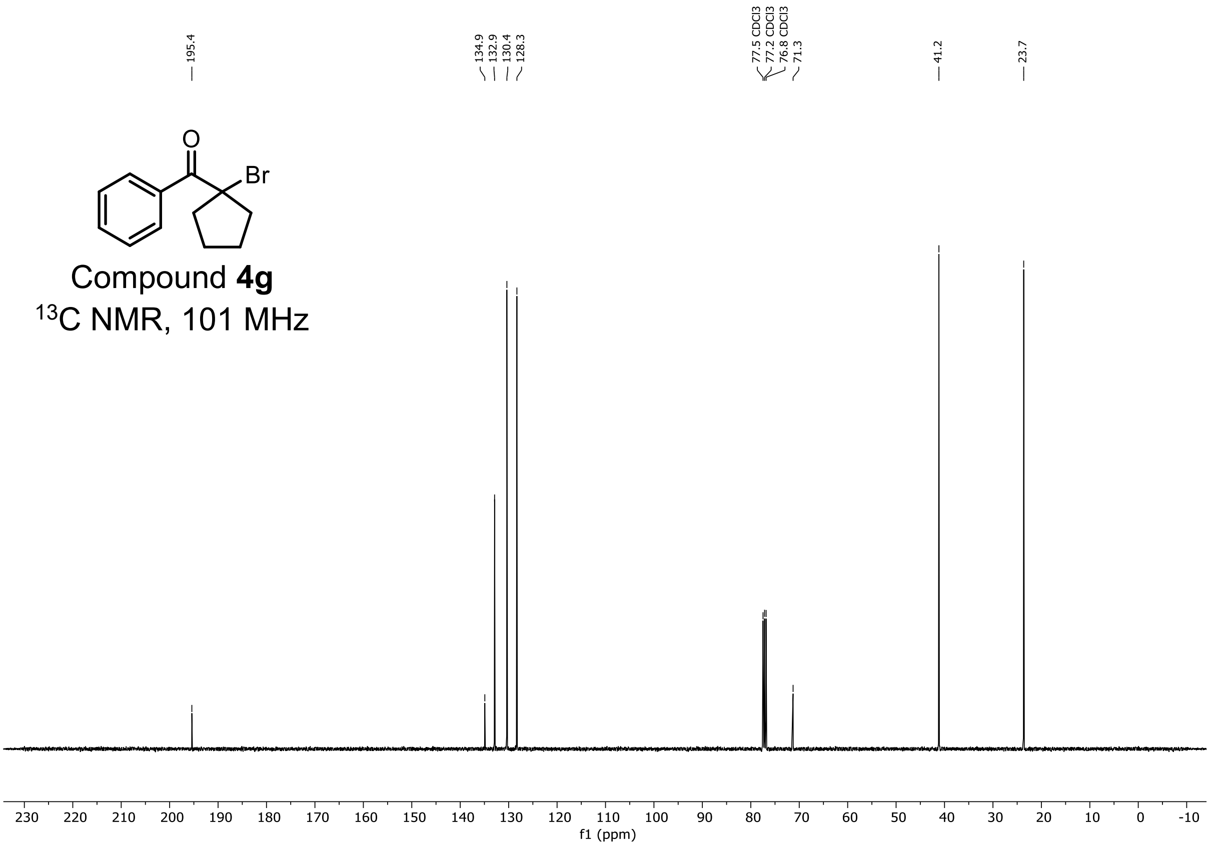
195.4
134.9
132.9
130.4
128.3
77.5 CDCl3
77.2 CDCl3
76.8 CDCl3
71.3
41.2
23.7
O
Br
Compound 4g
13C NMR, 101 MHz
230 220 210 200 190 180 170 160 150 140 130 120 110 100 90 80 70 60 50 40 30 20 10 0 -10
f1 (ppm)

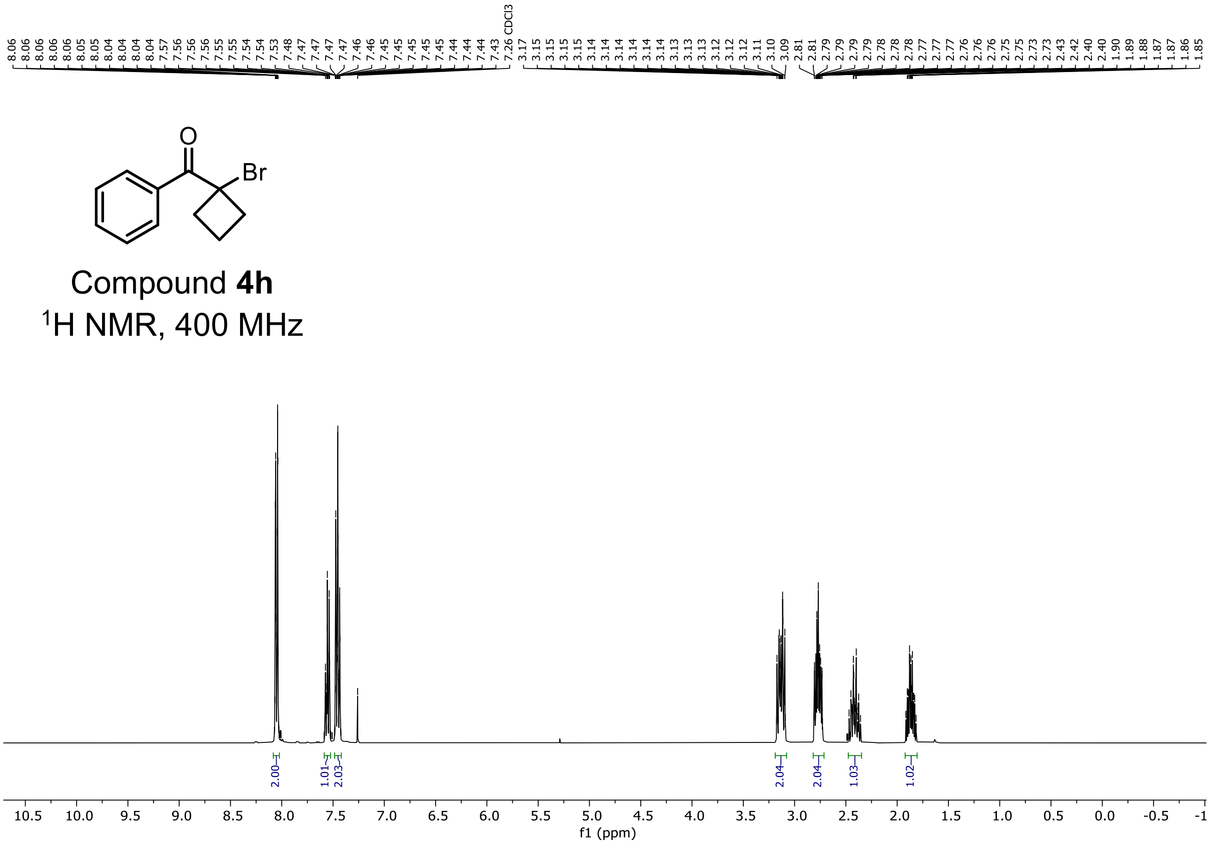
8.06 8.06 8.06 8.06 8.06 8.05 8.04 8.04 8.04 8.04 7.57 7.56 7.56 7.56 7.55 7.55 7.54 7.53 7.48 7.47 7.47 7.47 7.47 7.46 7.45 7.45 7.45 7.45 7.44 7.44 7.44 7.43 7.26 CDCl3 3.17 3.15 3.15 3.15 3.15 3.14 3.14 3.14 3.14 3.14 3.13 3.13 3.13 3.12 3.12 3.12 3.11 3.10 3.09 2.81 2.81 2.79 2.79 2.79 2.79 2.78 2.78 2.78 2.77 2.77 2.77 2.76 2.76 2.76 2.75 2.75 2.73 2.73 2.43 2.42 2.40 2.40 1.90 1.89 1.88 1.87 1.87 1.86 1.85
O
Br
Compound **4h**
^{1}H NMR, 400 MHz
2.00 1.01 2.03 2.04 2.04 1.03 1.02
f1 (ppm)

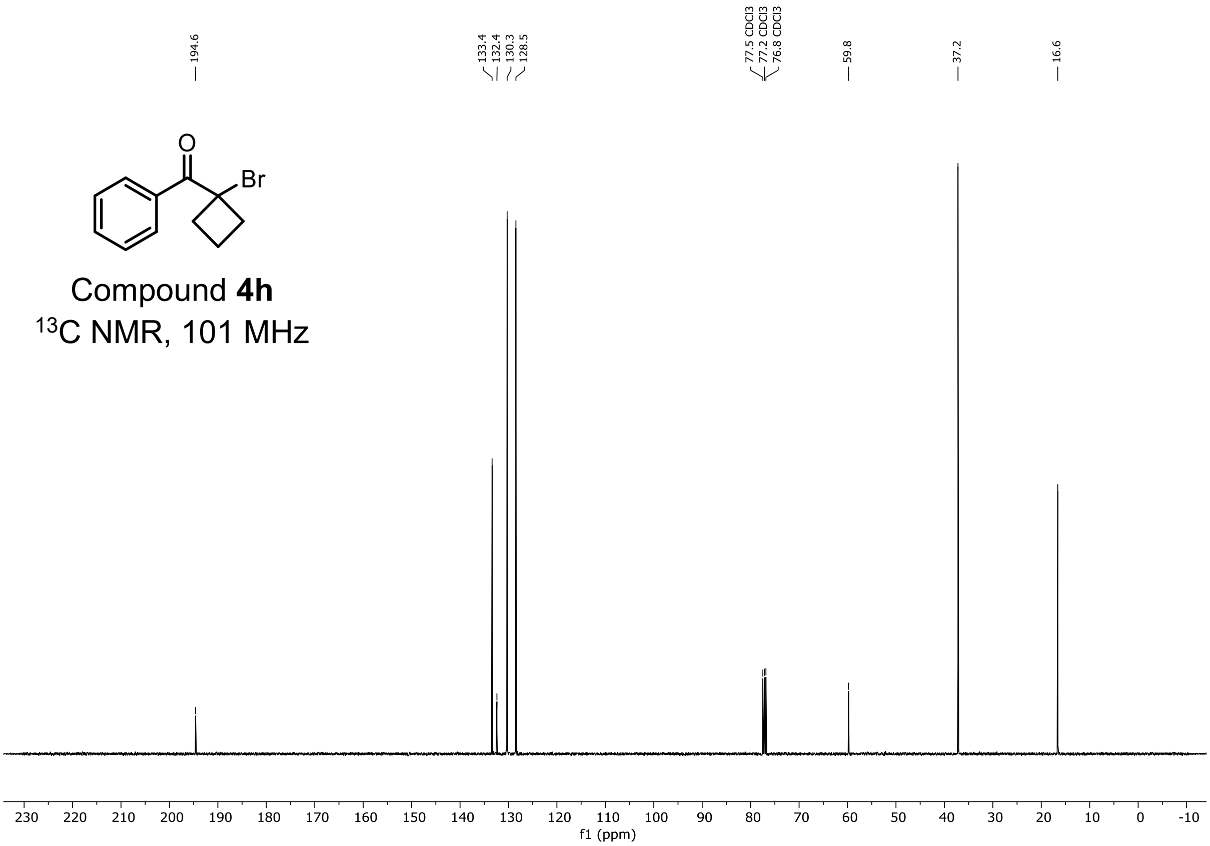
194.6
133.4 132.4 130.3 128.5
77.5 CDCl3 77.2 CDCl3 76.8 CDCl3
59.8
37.2
16.6
O
Br
Compound **4h**
^{13}C NMR, 101 MHz
f1 (ppm)

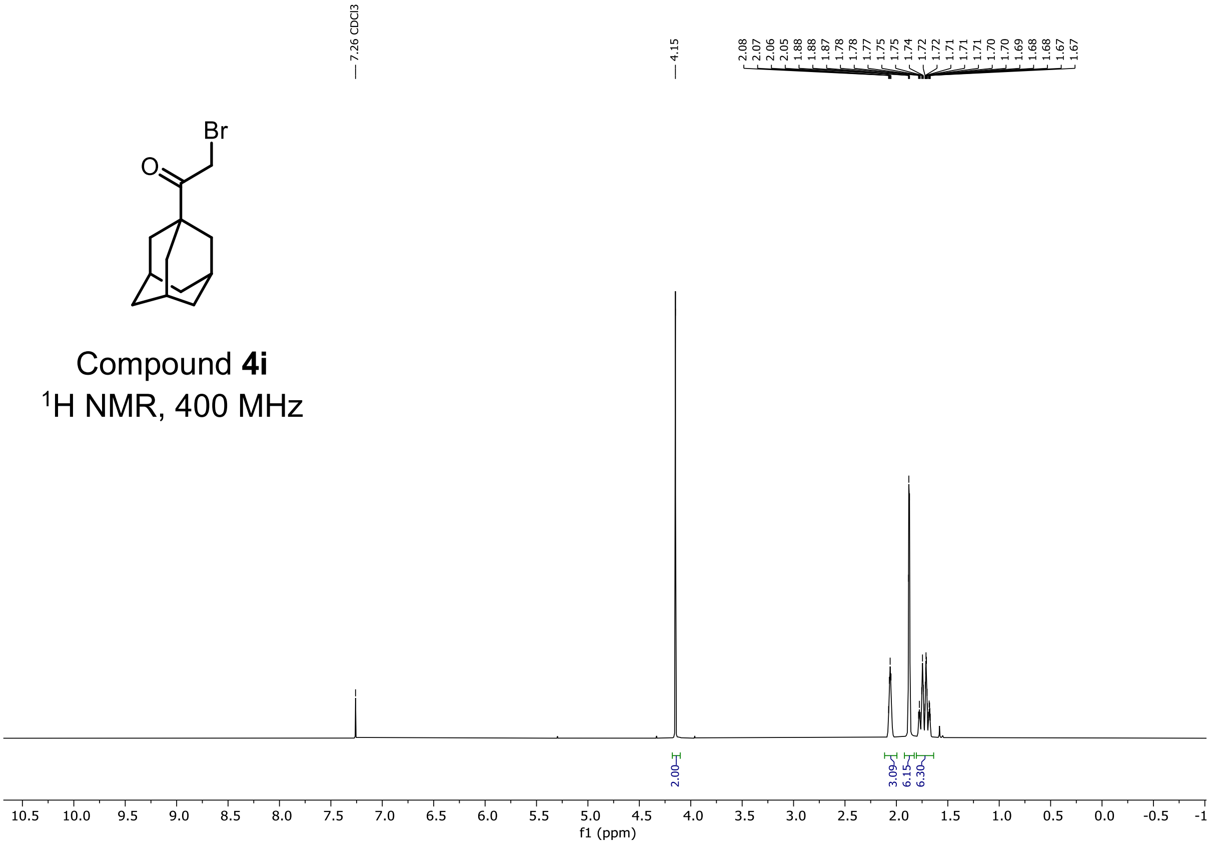

Compound **4i**
^{1}H NMR, 400 MHz

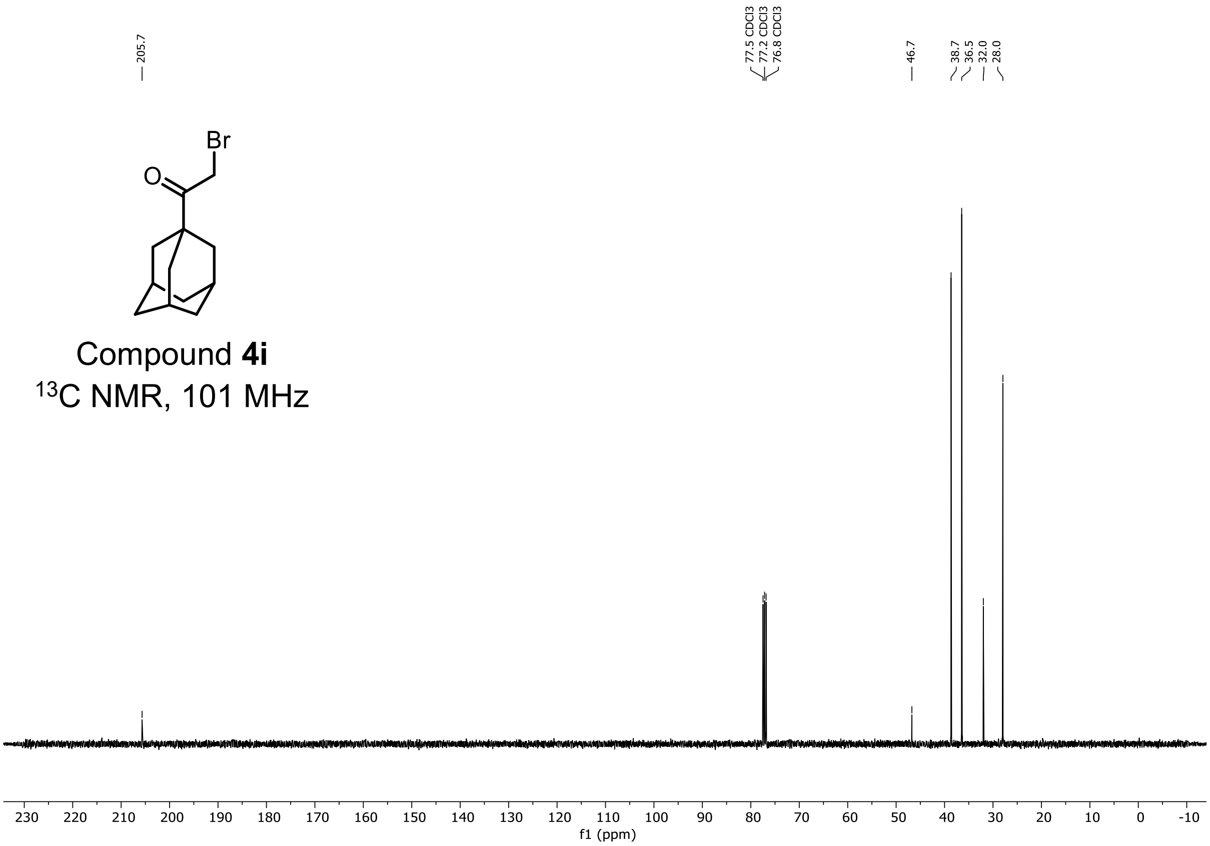

Compound **4i**
^{13}C NMR, 101 MHz

Compound **4j**
^{1}H NMR, 400 MHz

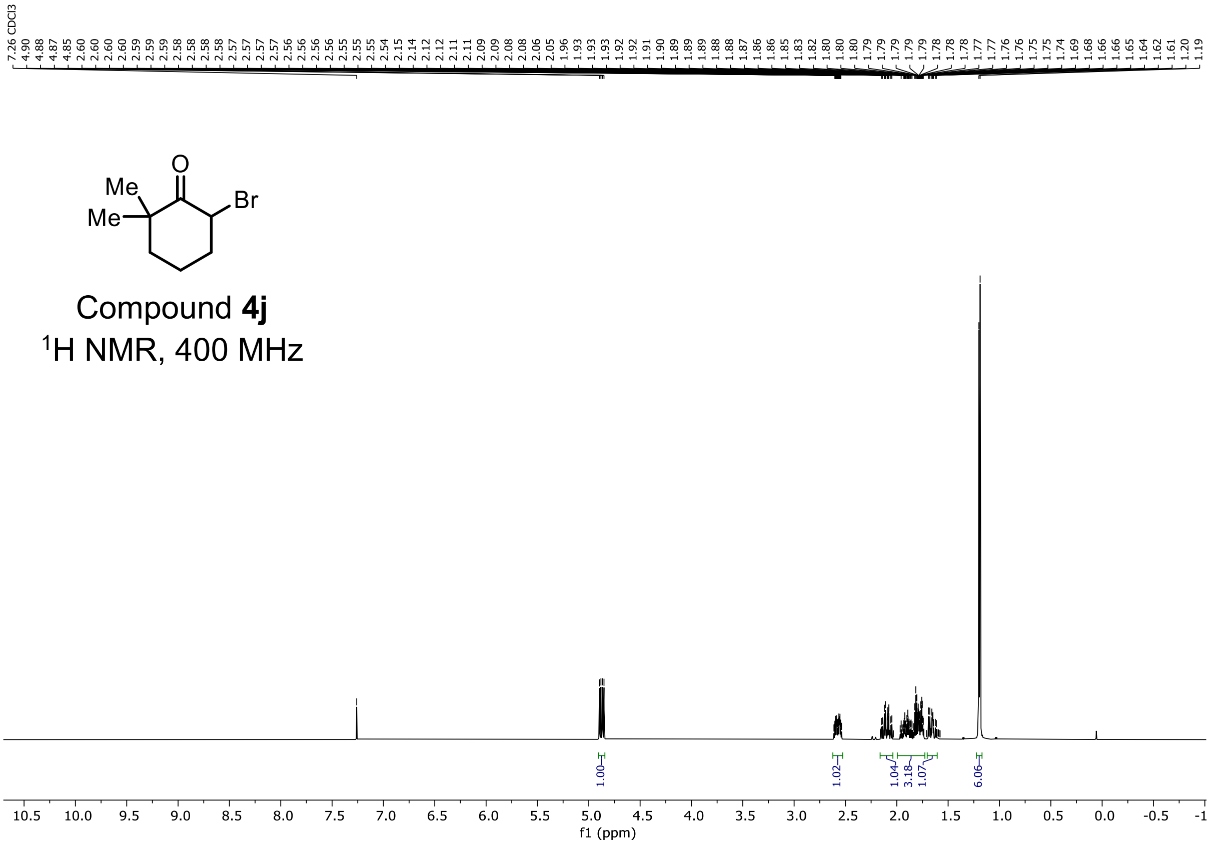

Compound **4j**
^{13}C NMR, 101 MHz

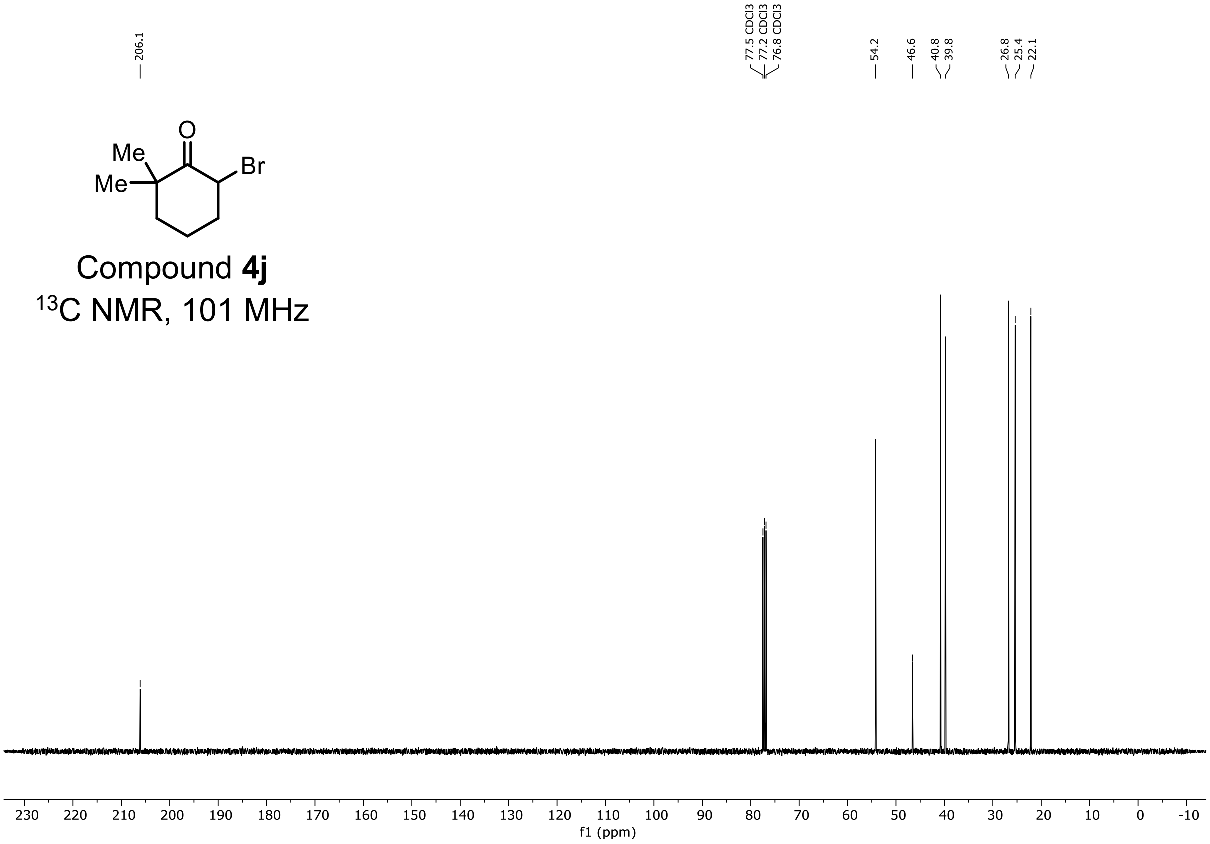

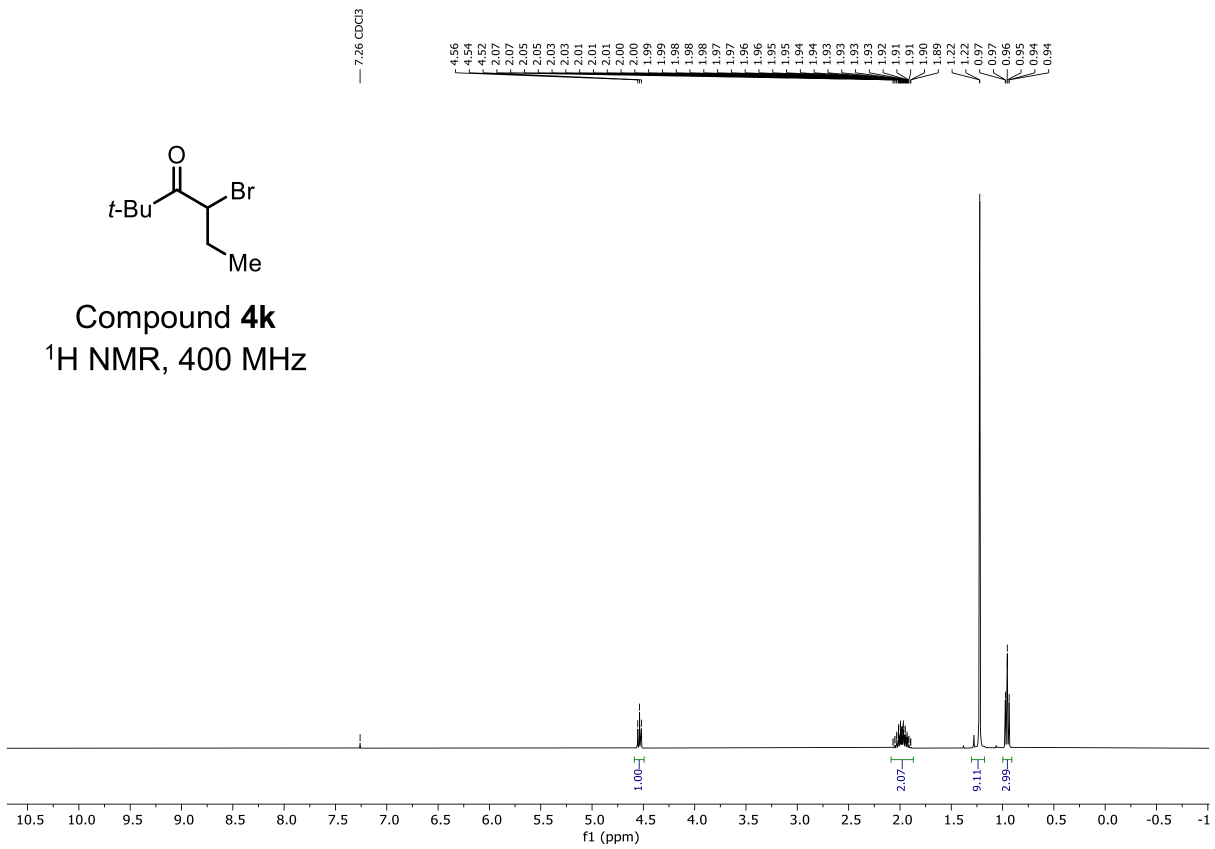
7.26 CDCl3
O
Br
t-Bu
Me
Compound 4k
1H NMR, 400 MHz
1.00
2.07
9.11
2.99
f1 (ppm)

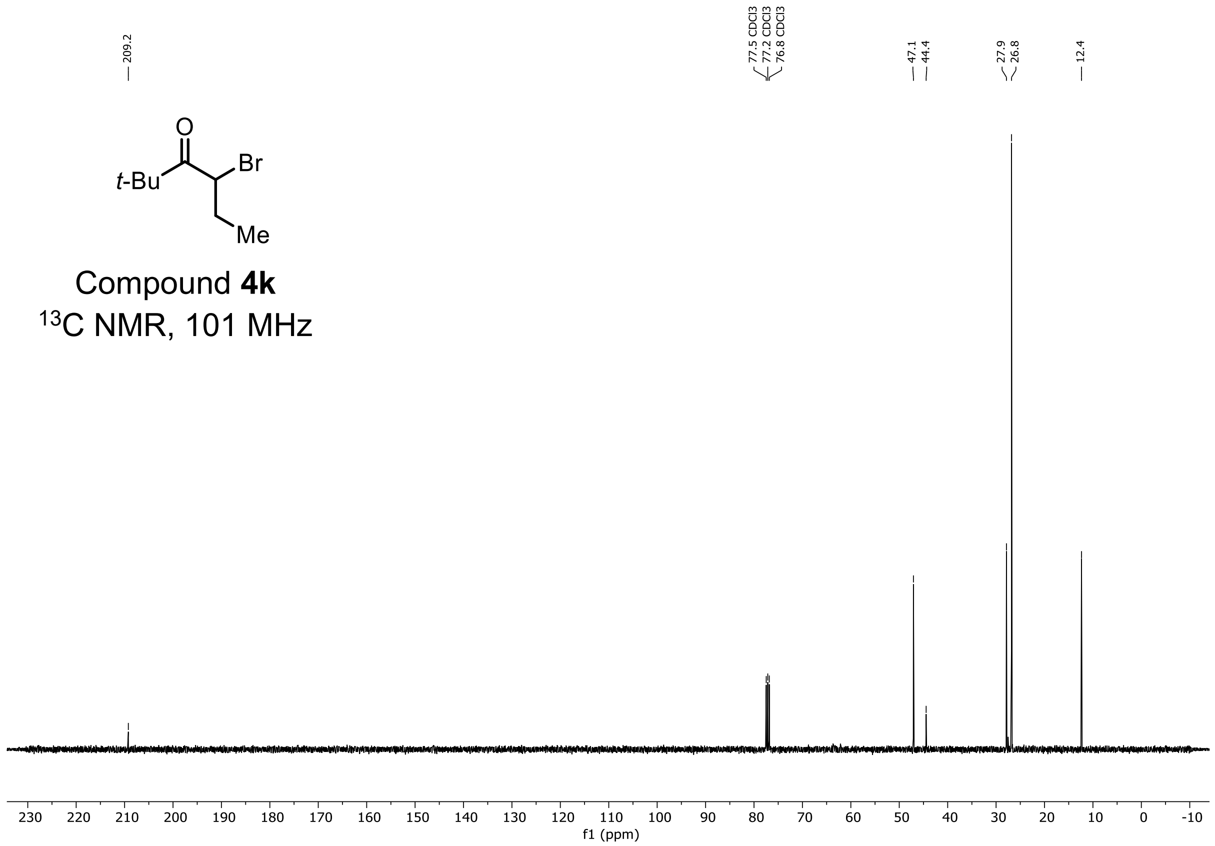
209.2
77.5 CDCl3
77.2 CDCl3
76.8 CDCl3
47.1
44.4
27.9
26.8
12.4
O
Br
t-Bu
Me
Compound 4k
13C NMR, 101 MHz
f1 (ppm)

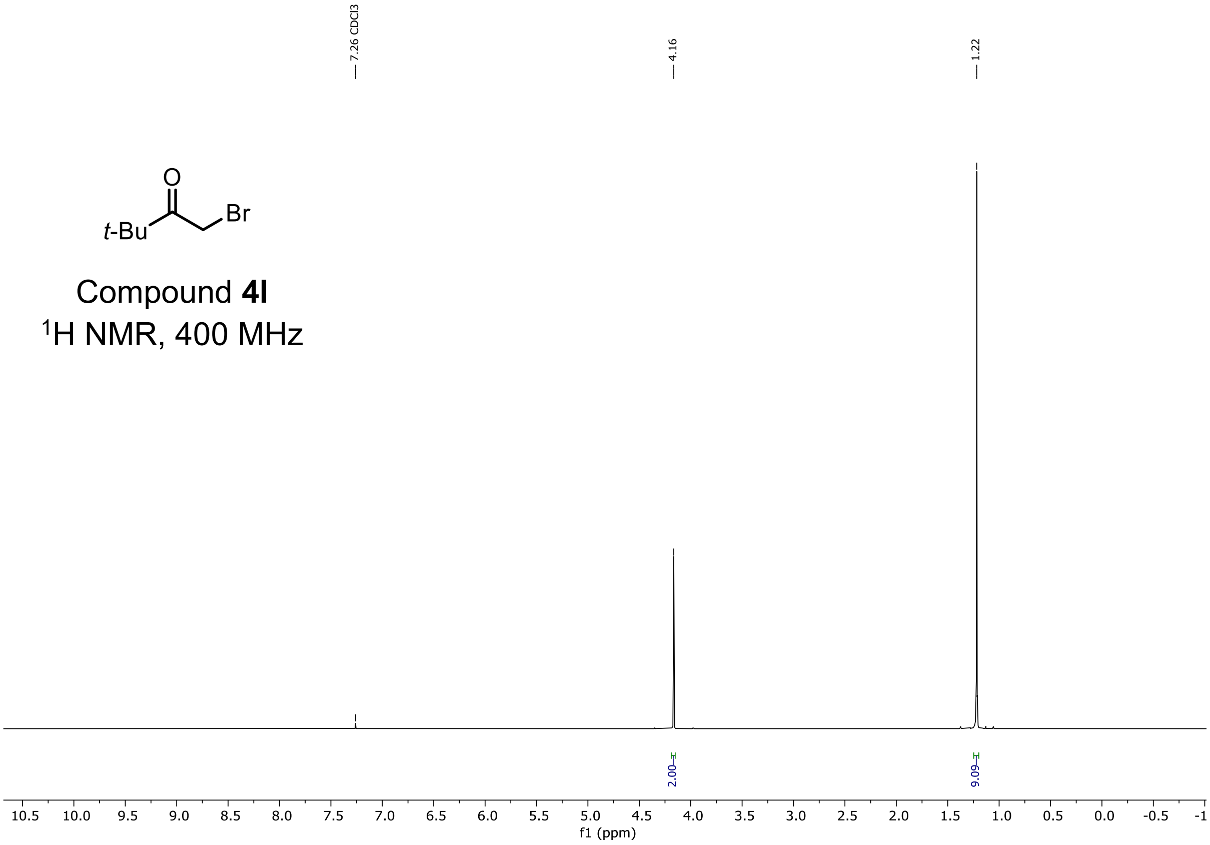
7.26 CDCl3
4.16
1.22
O
t-Bu
Br
Compound **4l**
^{1}H NMR, 400 MHz
2.00
9.09
10.5 10.0 9.5 9.0 8.5 8.0 7.5 7.0 6.5 6.0 5.5 5.0 4.5 4.0 3.5 3.0 2.5 2.0 1.5 1.0 0.5 0.0 -0.5 -1
f1 (ppm)

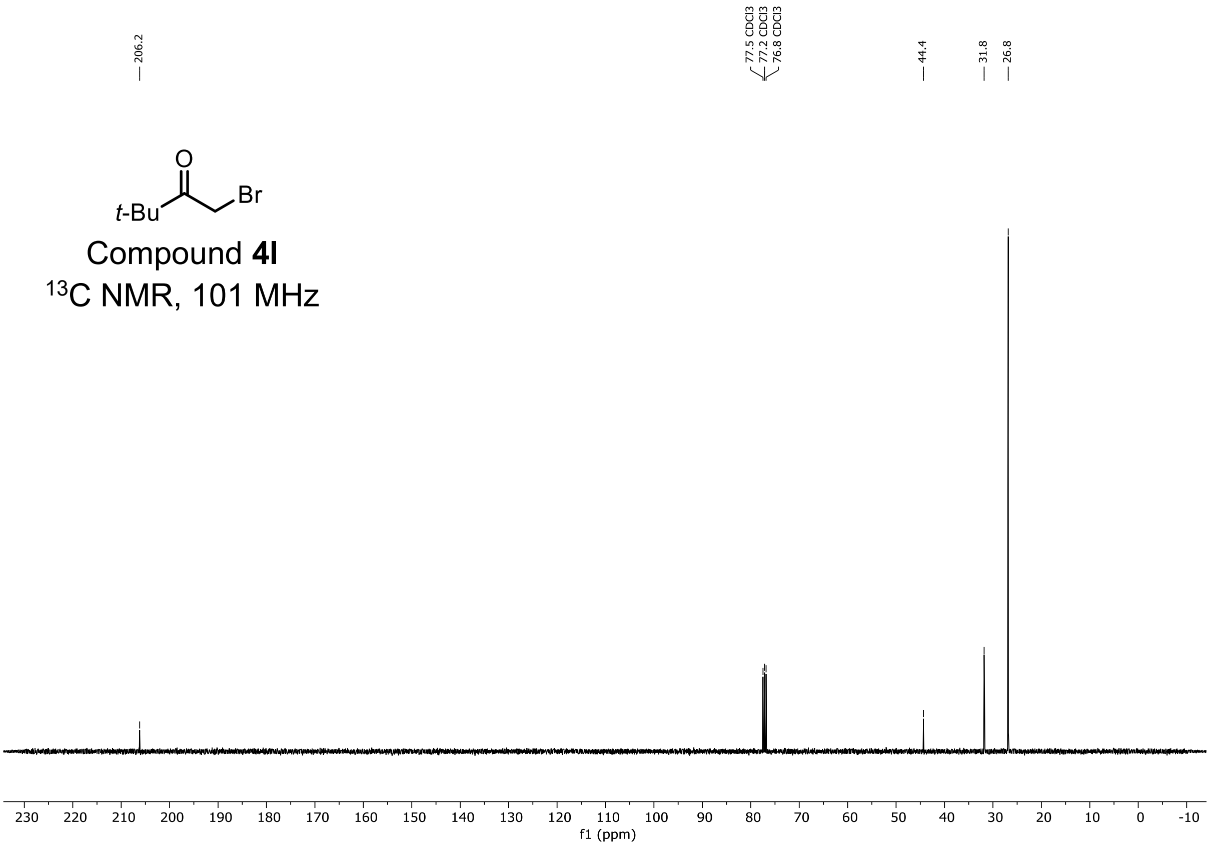
206.2
77.5 CDCl3
77.2 CDCl3
76.8 CDCl3
44.4
31.8
26.8
O
t-Bu
Br
Compound **4l**
^{13}C NMR, 101 MHz
230 220 210 200 190 180 170 160 150 140 130 120 110 100 90 80 70 60 50 40 30 20 10 0 -10
f1 (ppm)

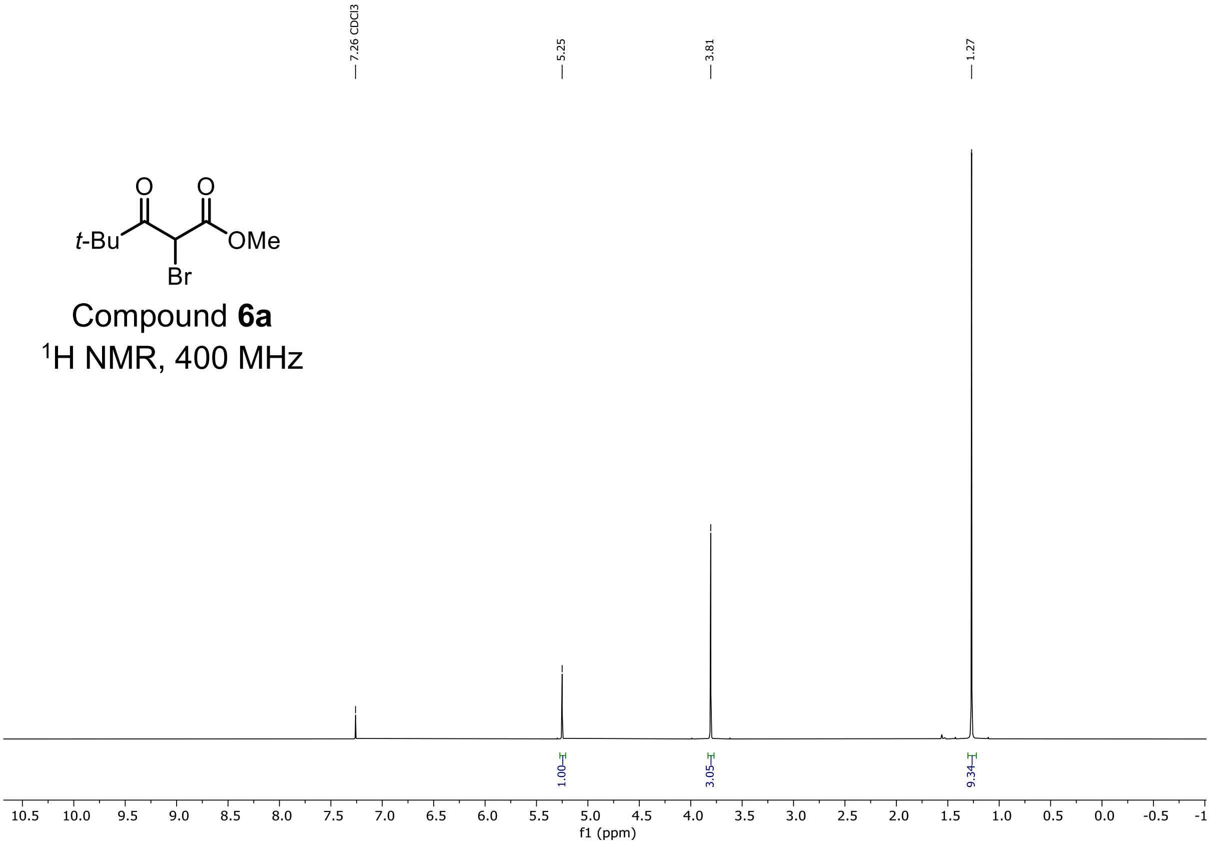

Compound **6a**
^{1}H NMR, 400 MHz

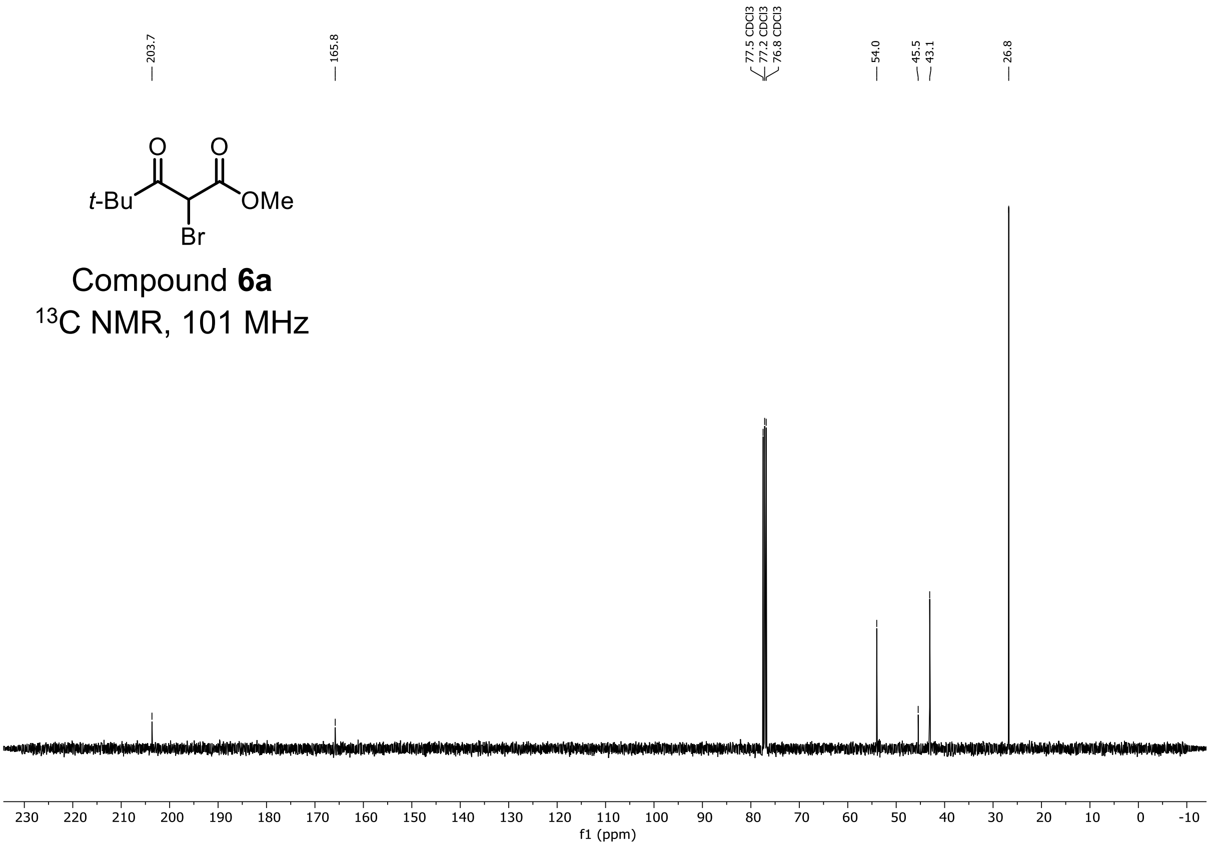

Compound **6a**
^{13}C NMR, 101 MHz

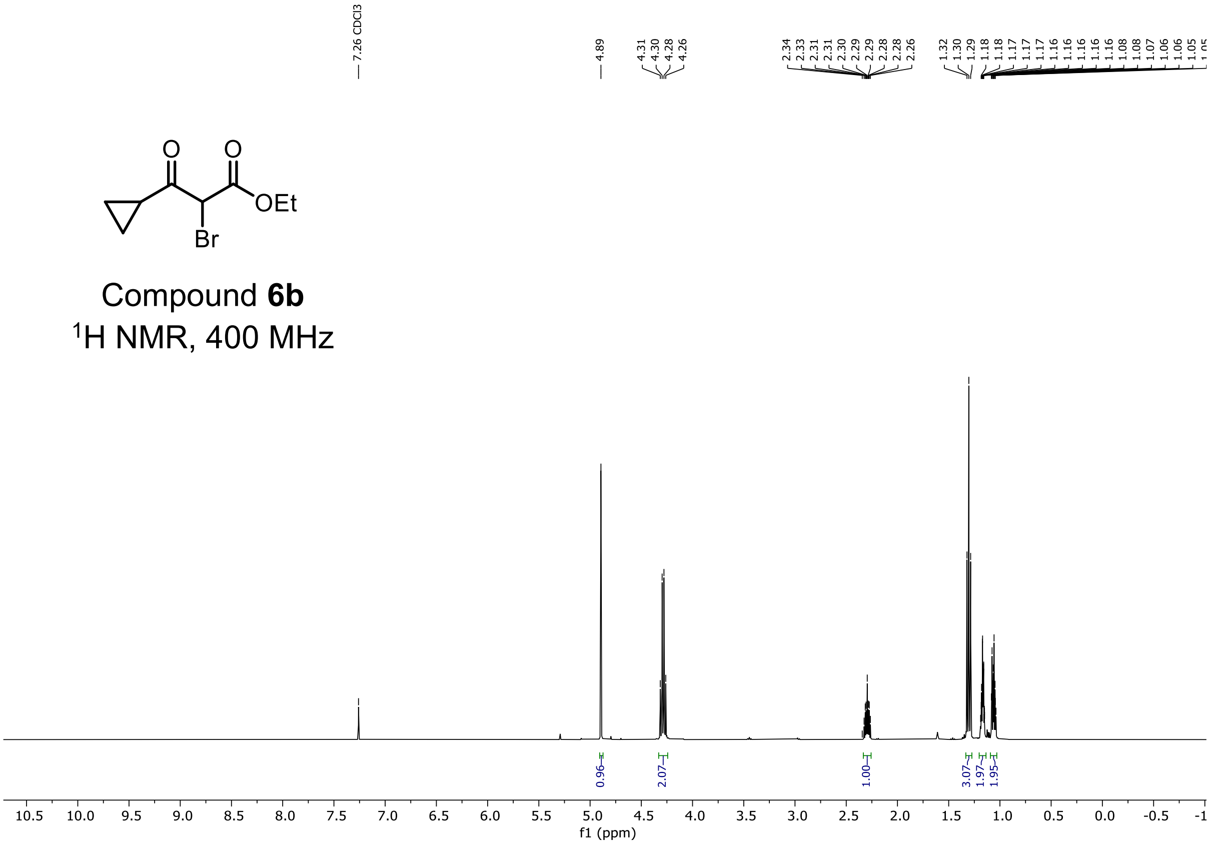

Compound **6b**
^{1}H NMR, 400 MHz

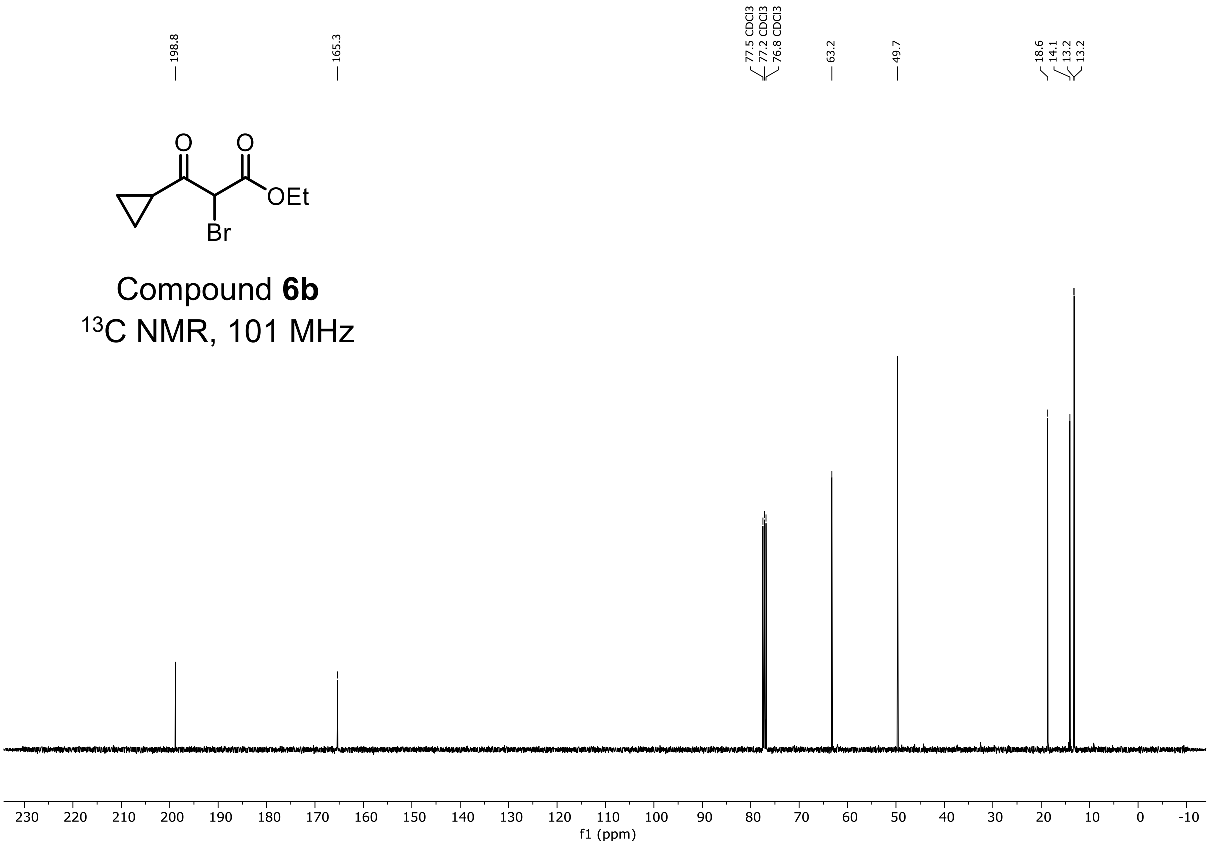

Compound **6b**
^{13}C NMR, 101 MHz

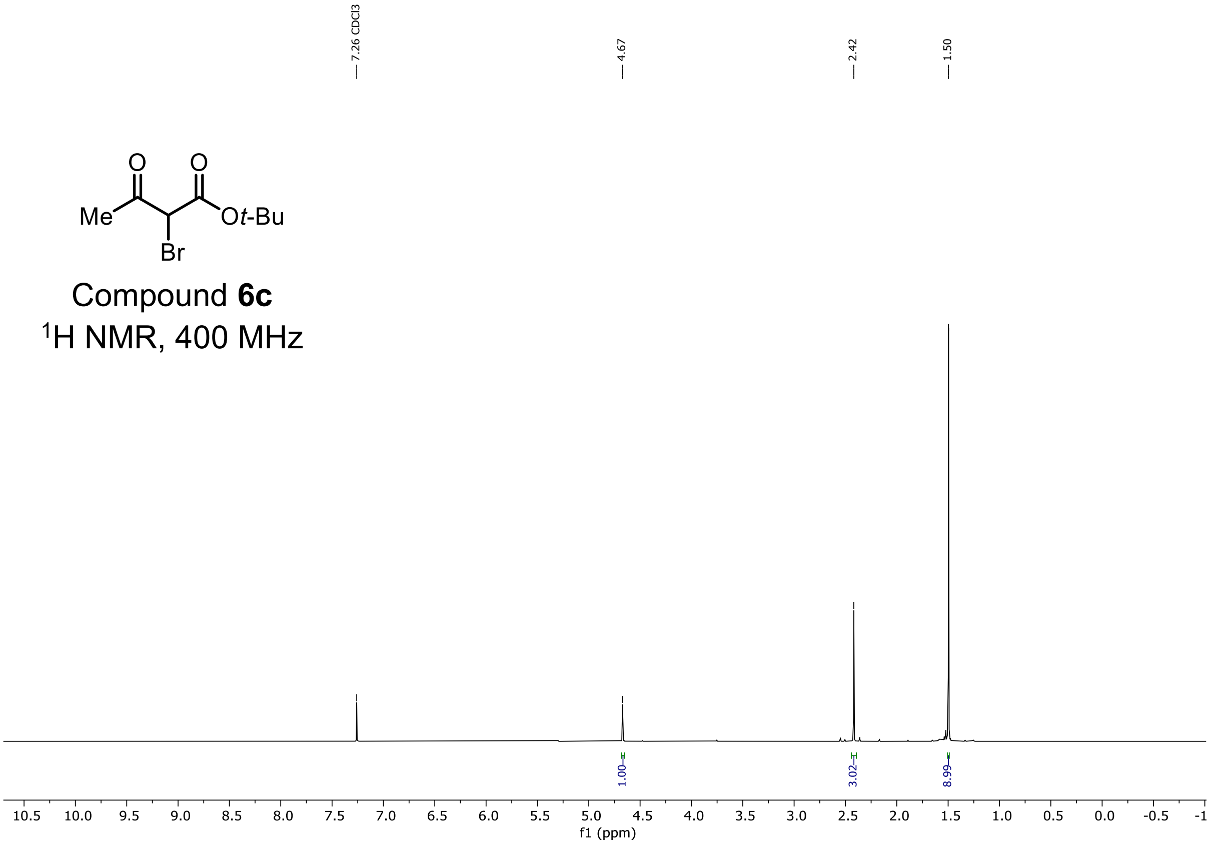

Compound **6c**
^{1}H NMR, 400 MHz

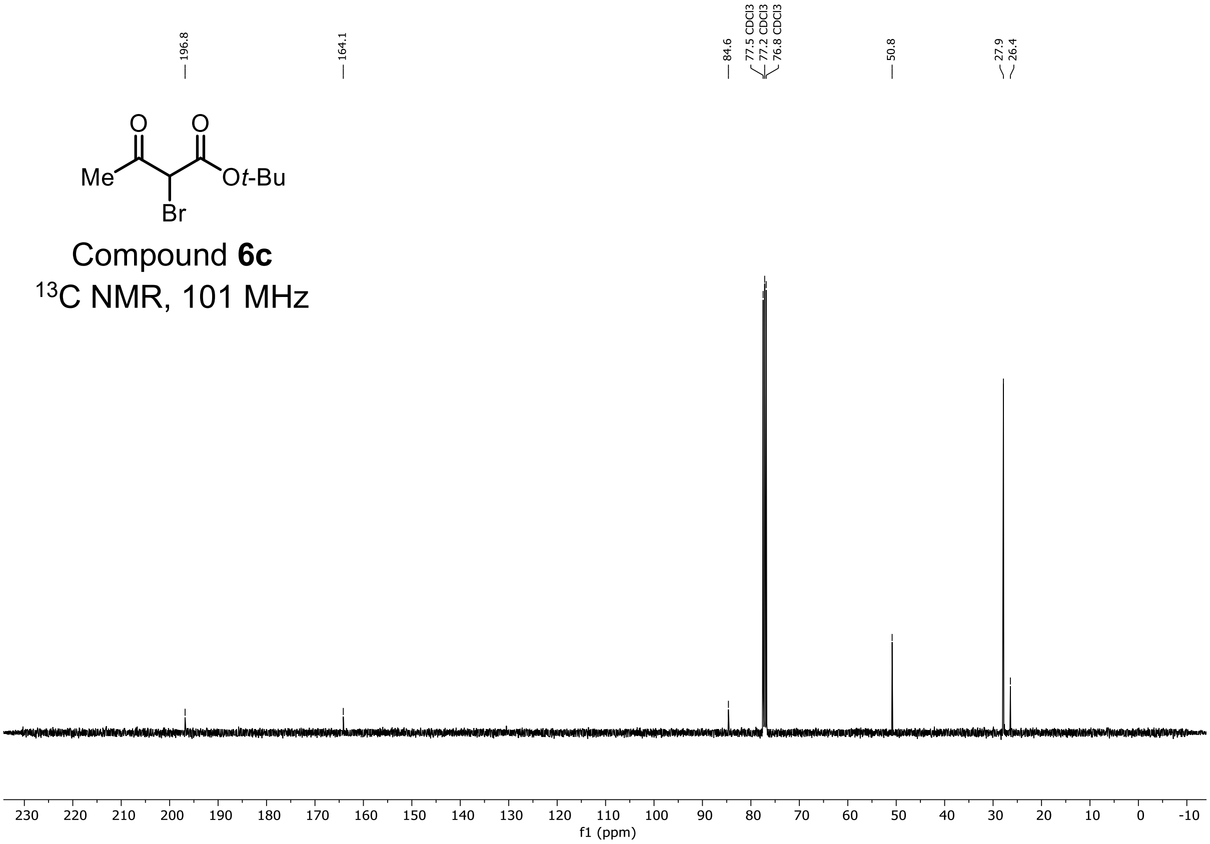

Compound **6c**
^{13}C NMR, 101 MHz

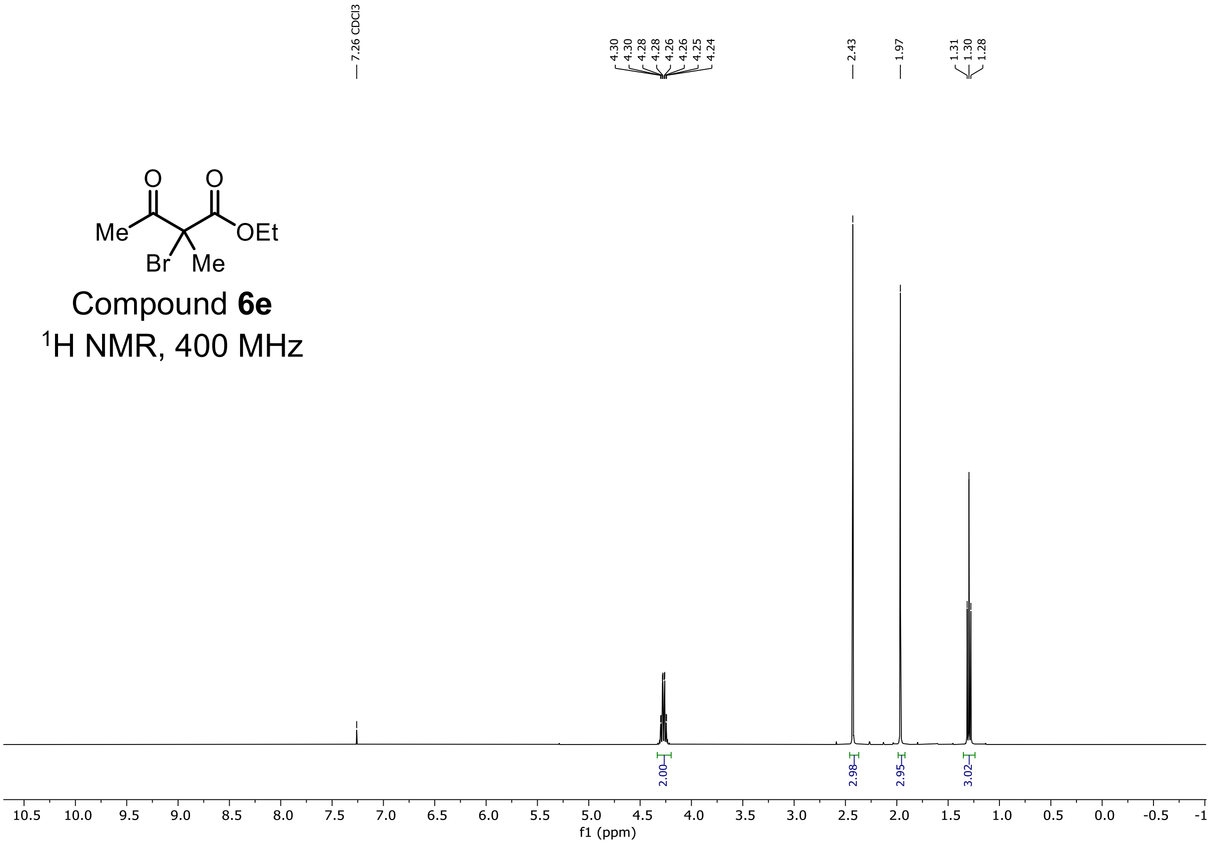

Compound **6e**
^{1}H NMR, 400 MHz

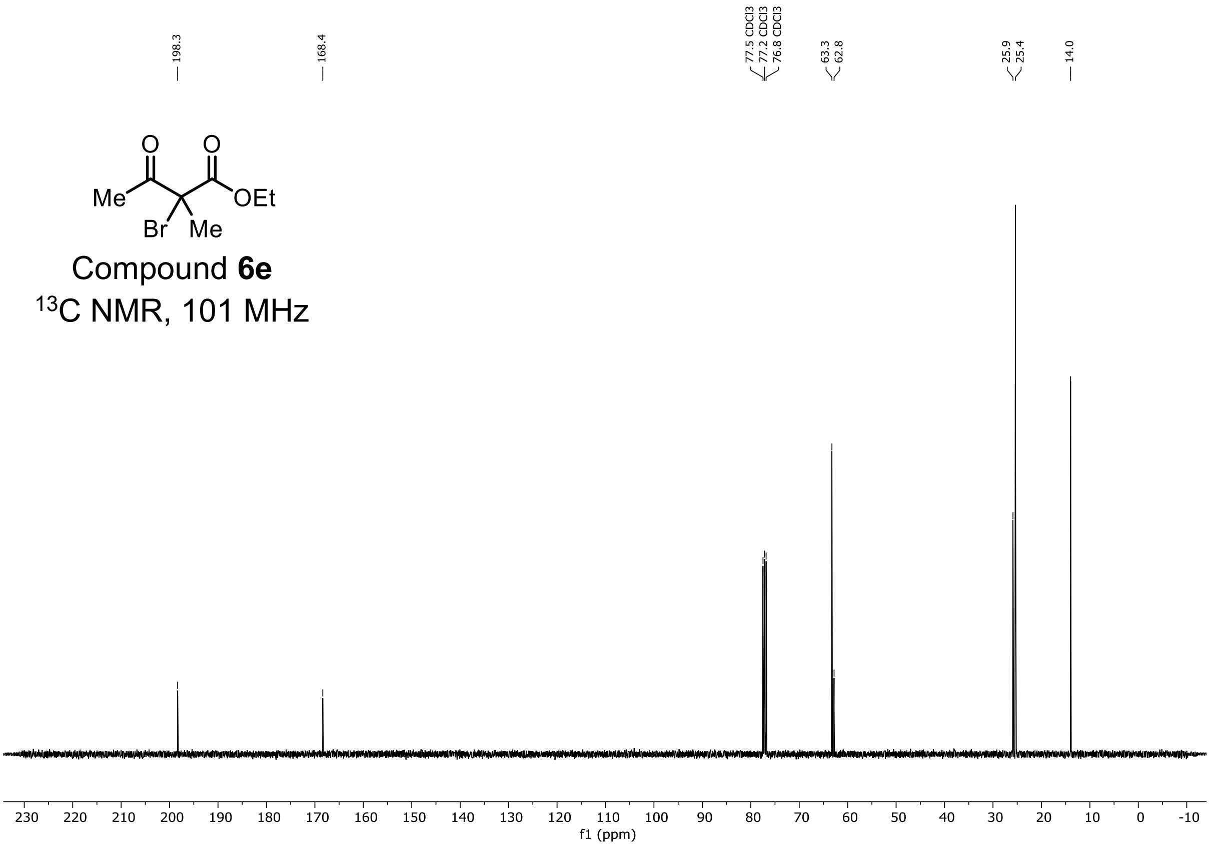

Compound **6e**
^{13}C NMR, 101 MHz

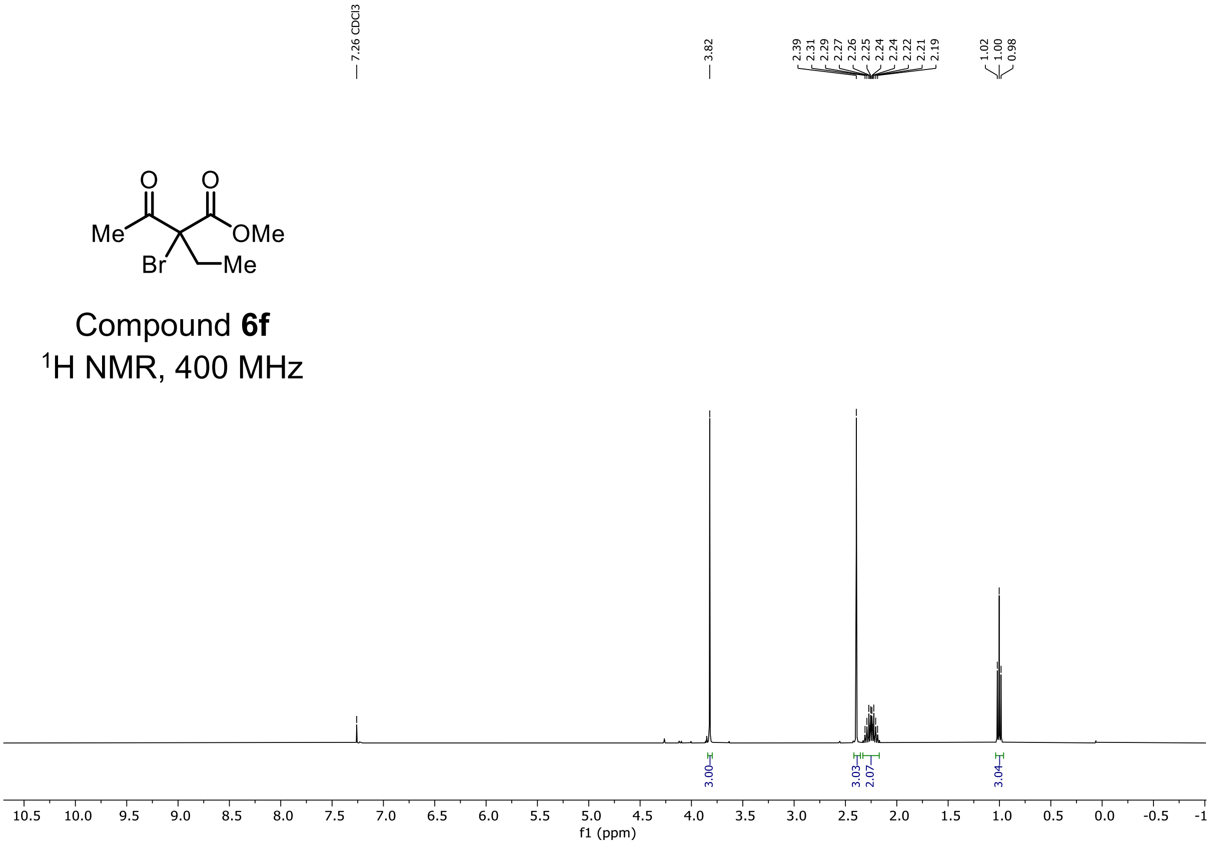

Compound **6f**

^{1}H NMR, 400 MHz

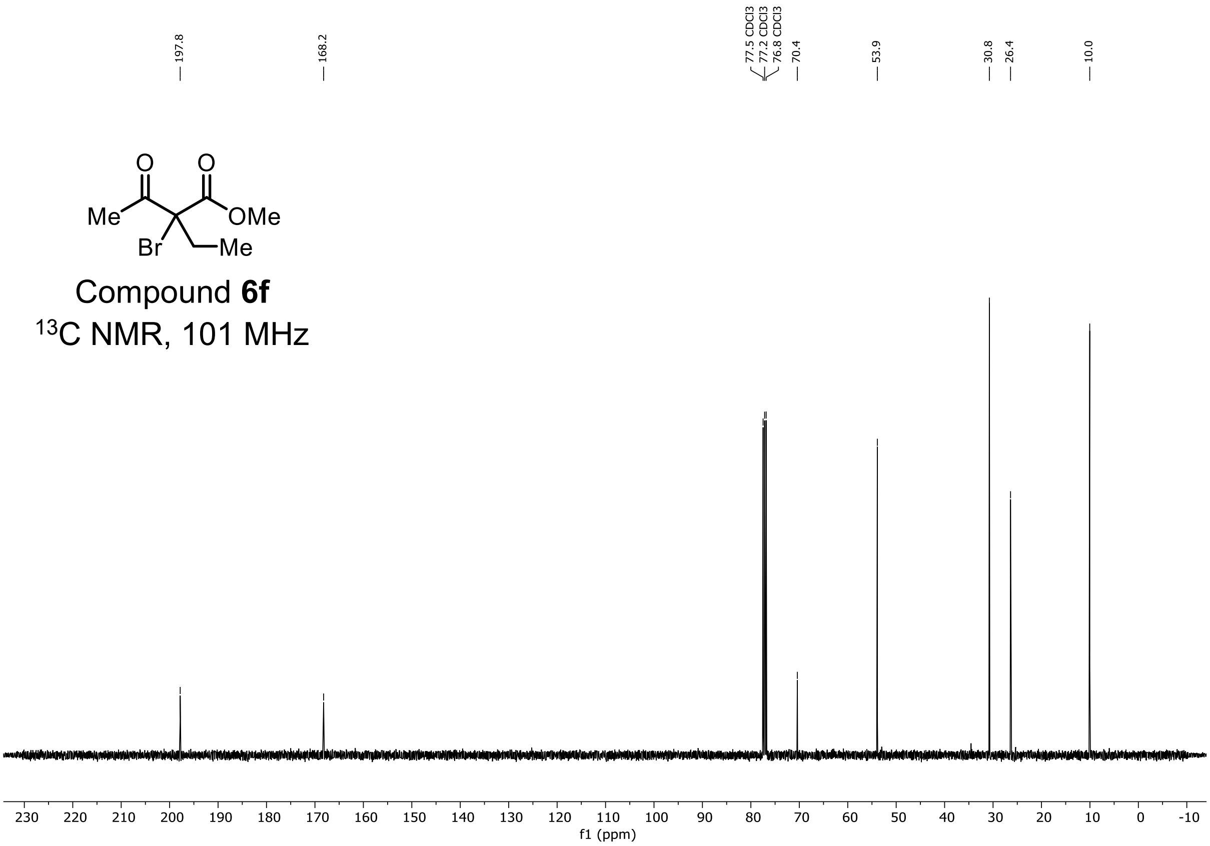

Compound **6f**

^{13}C NMR, 101 MHz

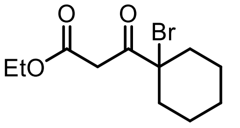

Compound **6g**
^{1}H NMR, 500 MHz

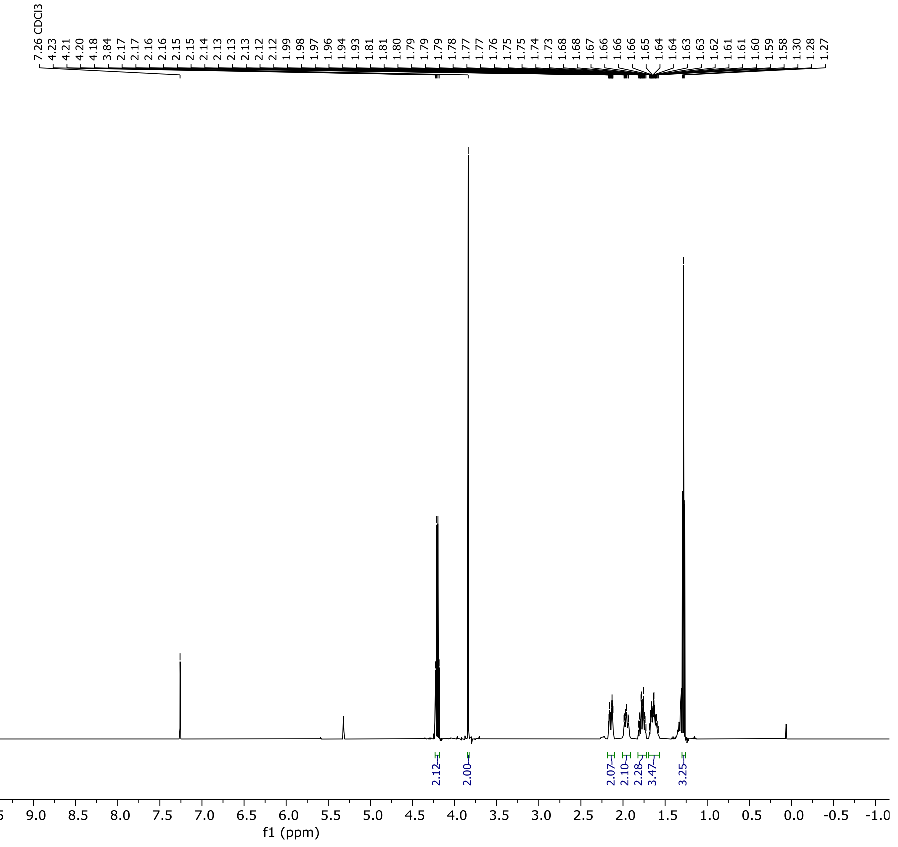

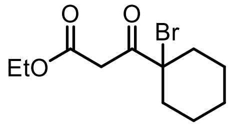

Compound **6g**
^{13}C NMR, 126 MHz

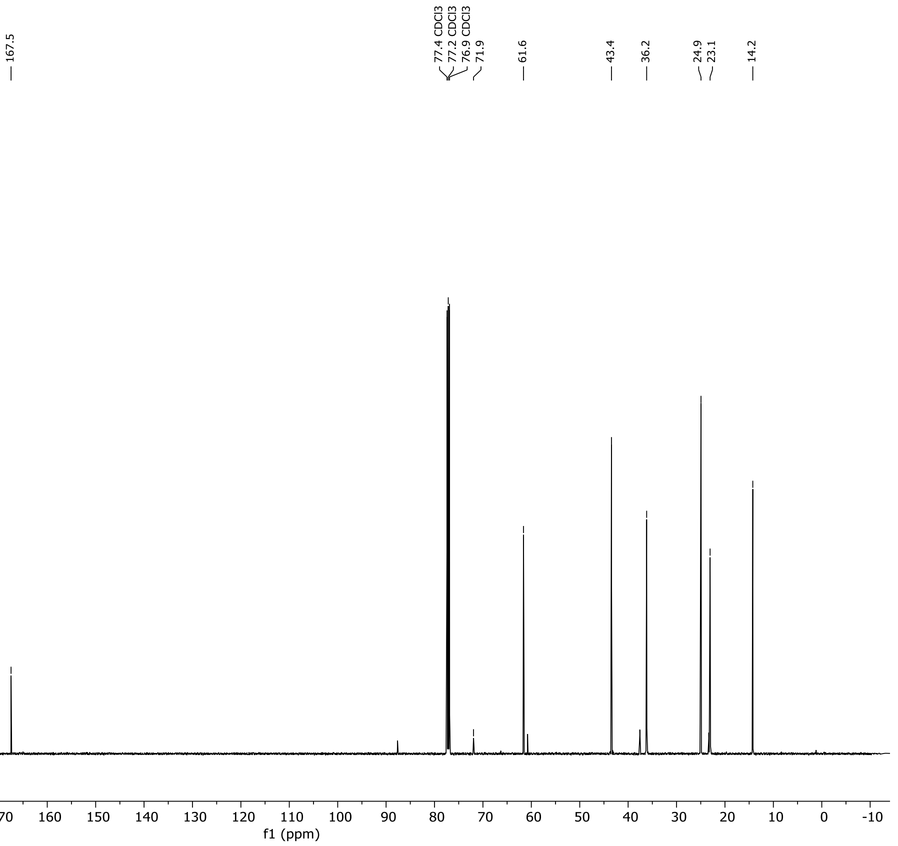

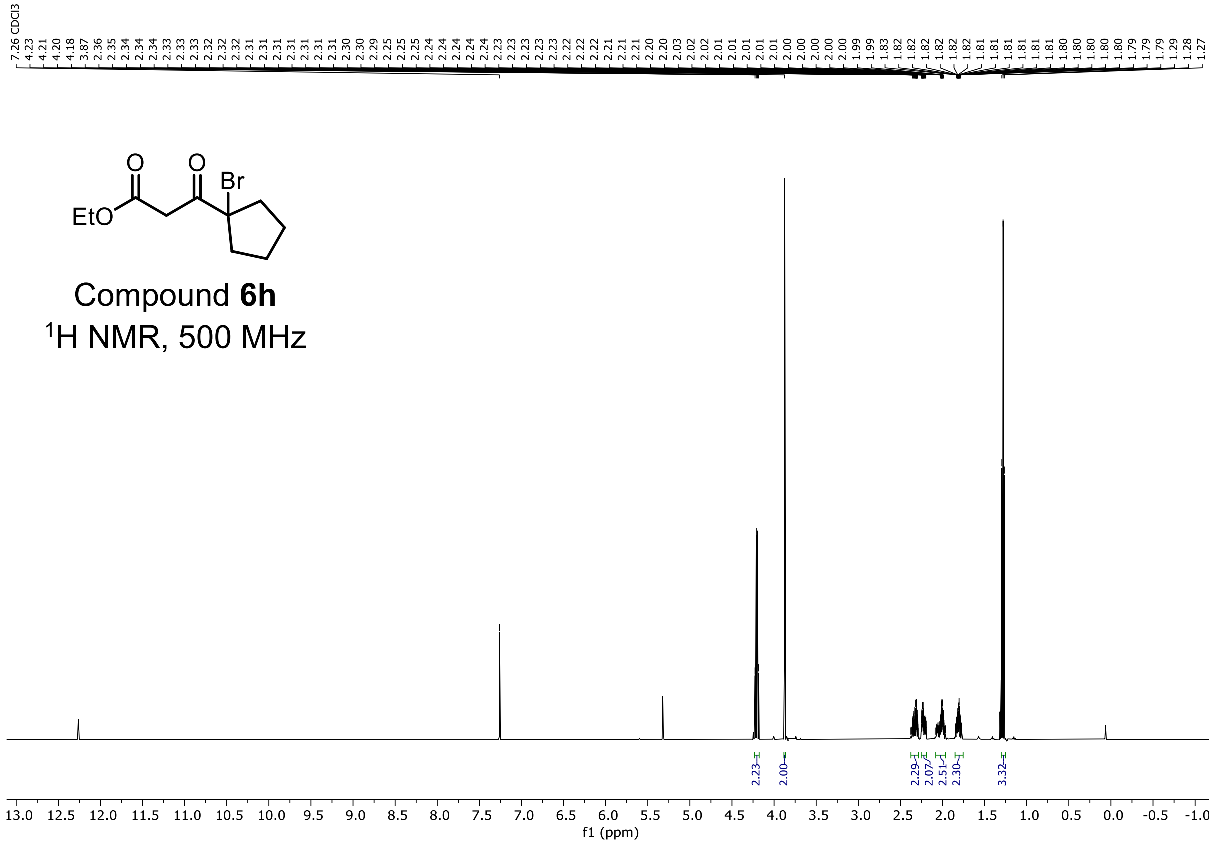
EtO
O
O
Br
Compound 6h
1H NMR, 500 MHz
f1 (ppm)

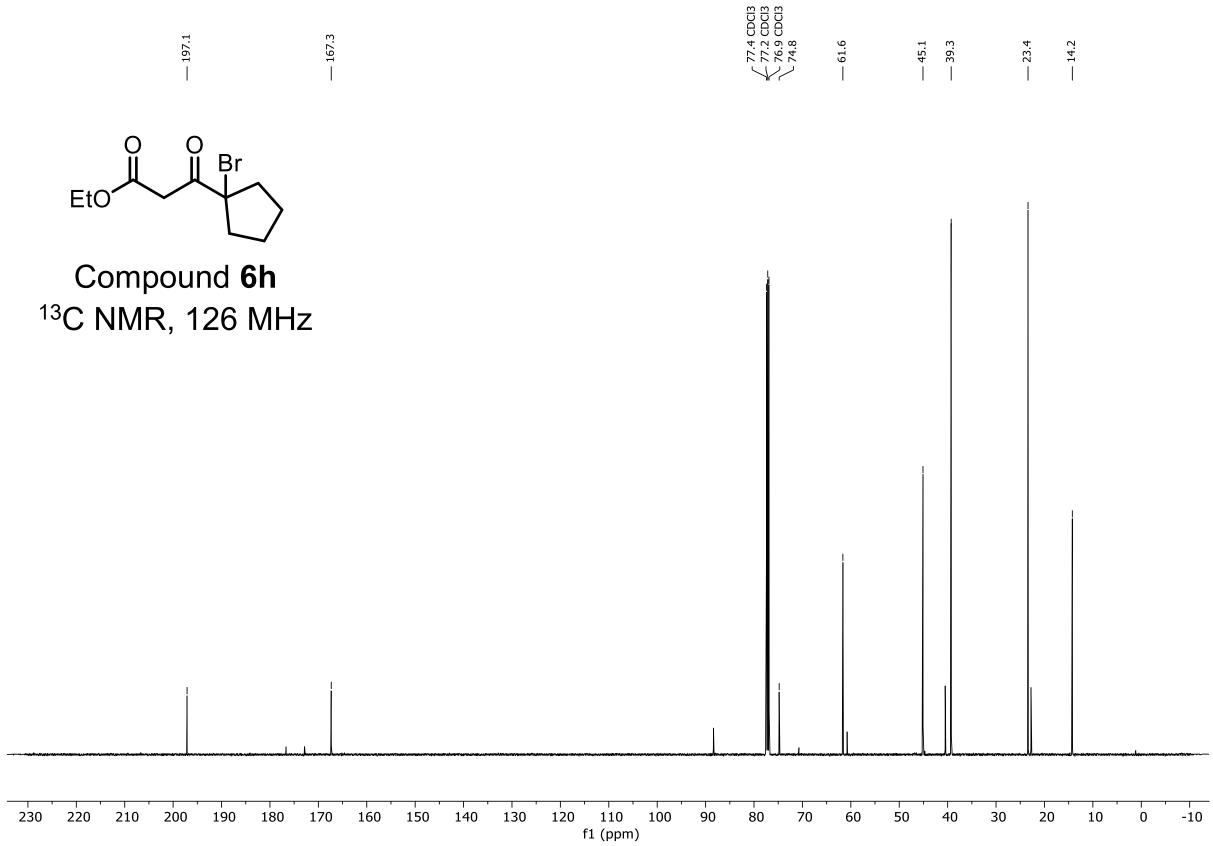
197.1
167.3
77.4 CDCl3
77.2 CDCl3
76.9 CDCl3
74.8
61.6
45.1
39.3
23.4
14.2
EtO
O
O
Br
Compound 6h
13C NMR, 126 MHz
f1 (ppm)

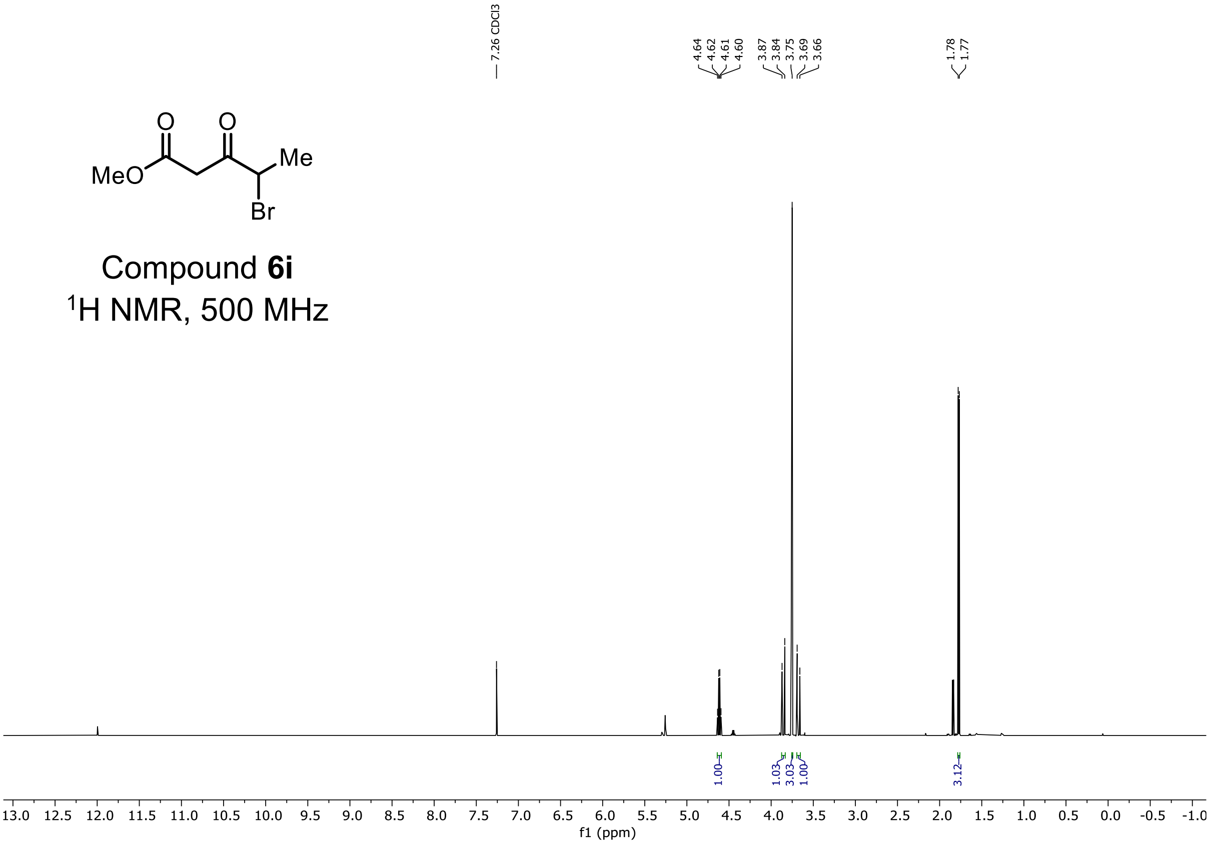
Compound **6i**
^{1}H NMR, 500 MHz
MeO
O
O
Me
Br
7.26 CDCl3
4.64
4.62
4.61
4.60
3.87
3.84
3.75
3.69
3.66
1.78
1.77
1.00
1.03
3.03
1.00
3.12
f1 (ppm)

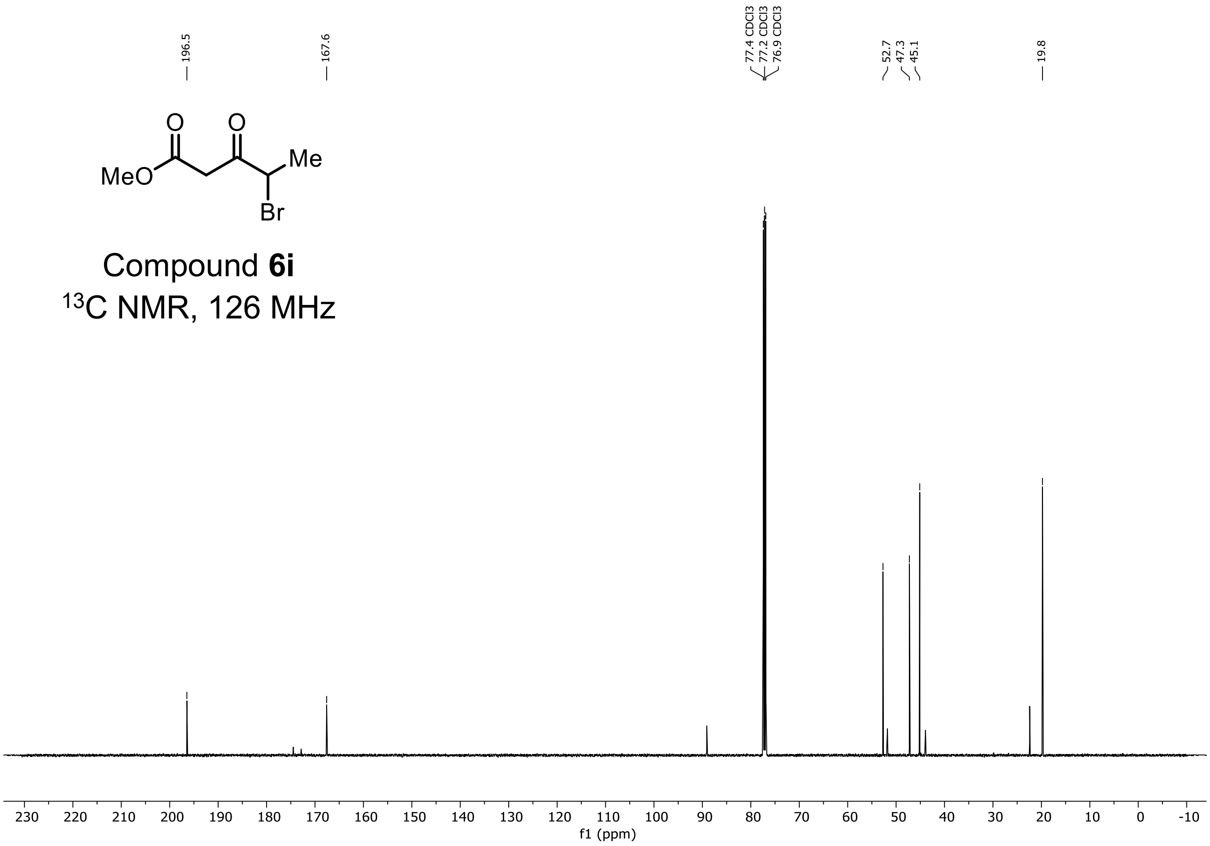
Compound **6i**
^{13}C NMR, 126 MHz
MeO
O
O
Me
Br
196.5
167.6
77.4 CDCl3
77.2 CDCl3
76.9 CDCl3
52.7
47.3
45.1
19.8
f1 (ppm)

Compound **6j**
^{1}H NMR, 500 MHz

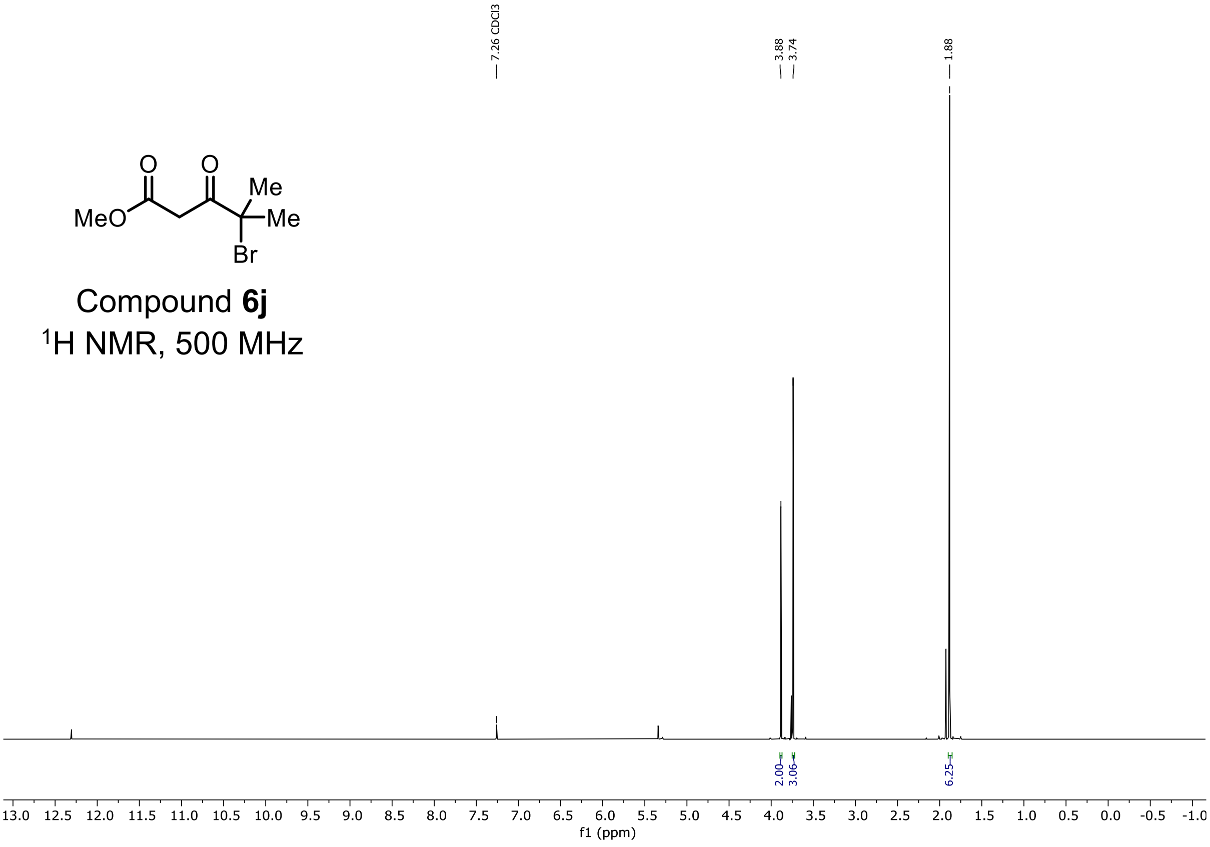

Compound **6j**
^{13}C NMR, 126 MHz

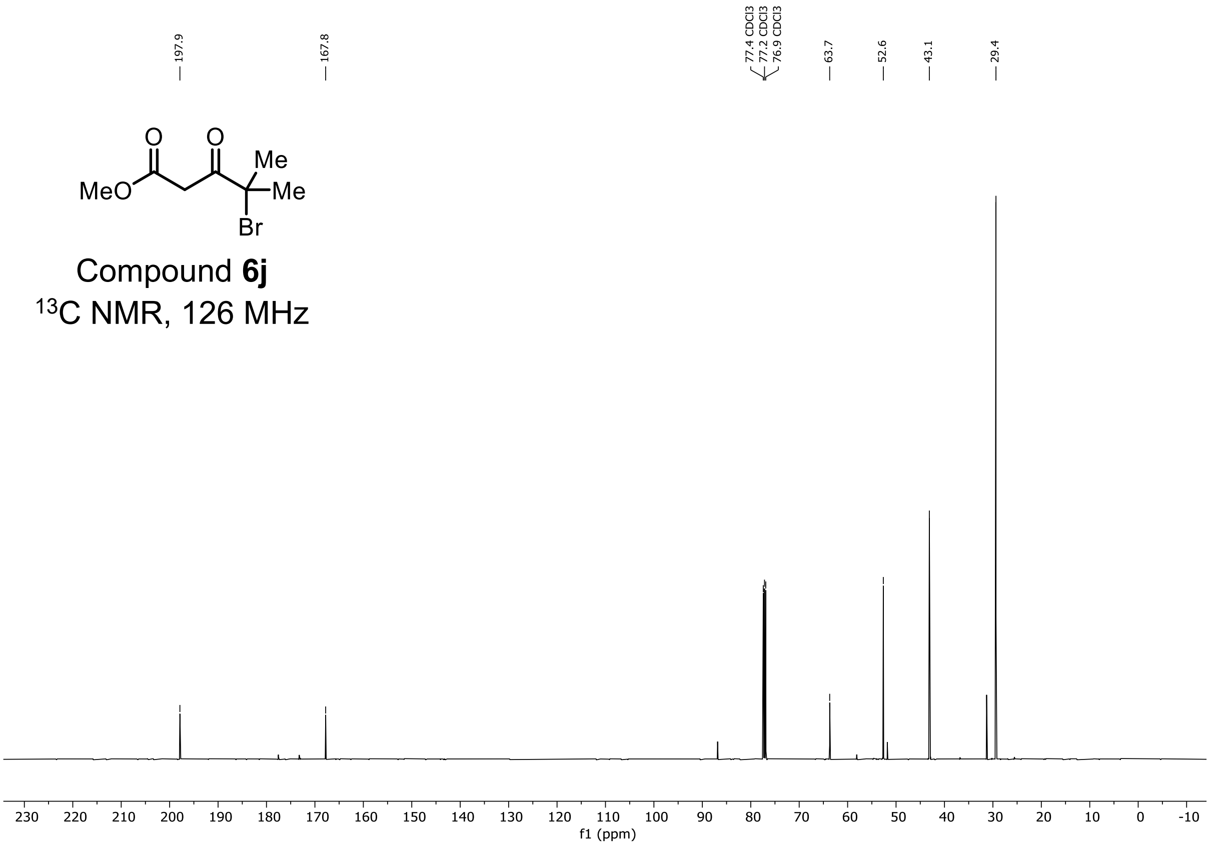

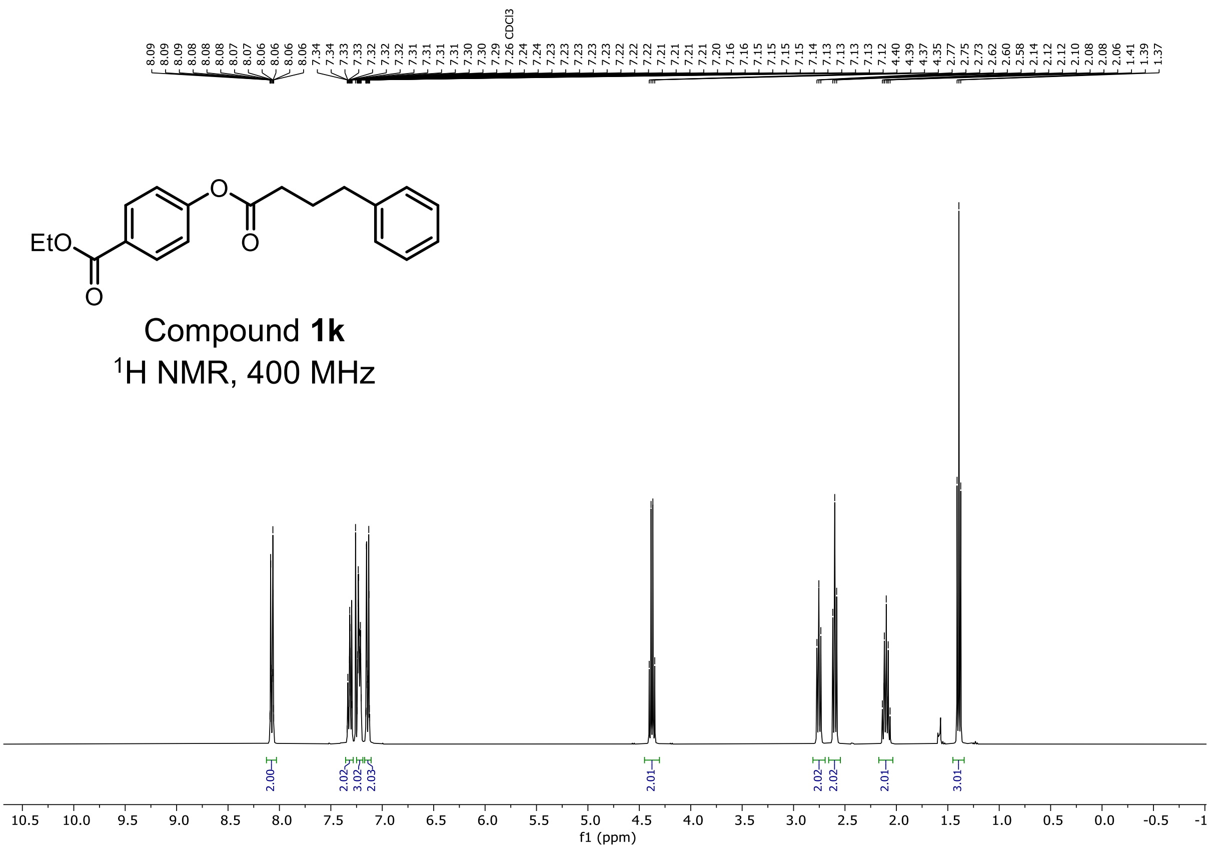
8.09 8.09 8.09 8.08 8.08 8.08 8.07 8.06 8.06 8.06 8.06 7.34 7.33 7.33 7.32 7.32 7.32 7.31 7.31 7.31 7.30 7.29 7.26 CDCl3 7.24 7.24 7.23 7.23 7.23 7.23 7.23 7.22 7.22 7.22 7.21 7.21 7.21 7.21 7.20 7.16 7.15 7.15 7.15 7.15 7.14 7.13 7.13 7.13 7.13 7.12 4.40 4.39 4.37 4.35 2.77 2.75 2.73 2.62 2.60 2.58 2.14 2.12 2.12 2.10 2.08 2.08 2.06 1.41 1.39 1.37
EtO
O
O
O
Compound **1k**
^{1}H NMR, 400 MHz
2.00 2.02 3.02 2.03 2.01 2.02 2.02 2.01 3.01
10.5 10.0 9.5 9.0 8.5 8.0 7.5 7.0 6.5 6.0 5.5 5.0 4.5 4.0 3.5 3.0 2.5 2.0 1.5 1.0 0.5 0.0 -0.5 -1
f1 (ppm)

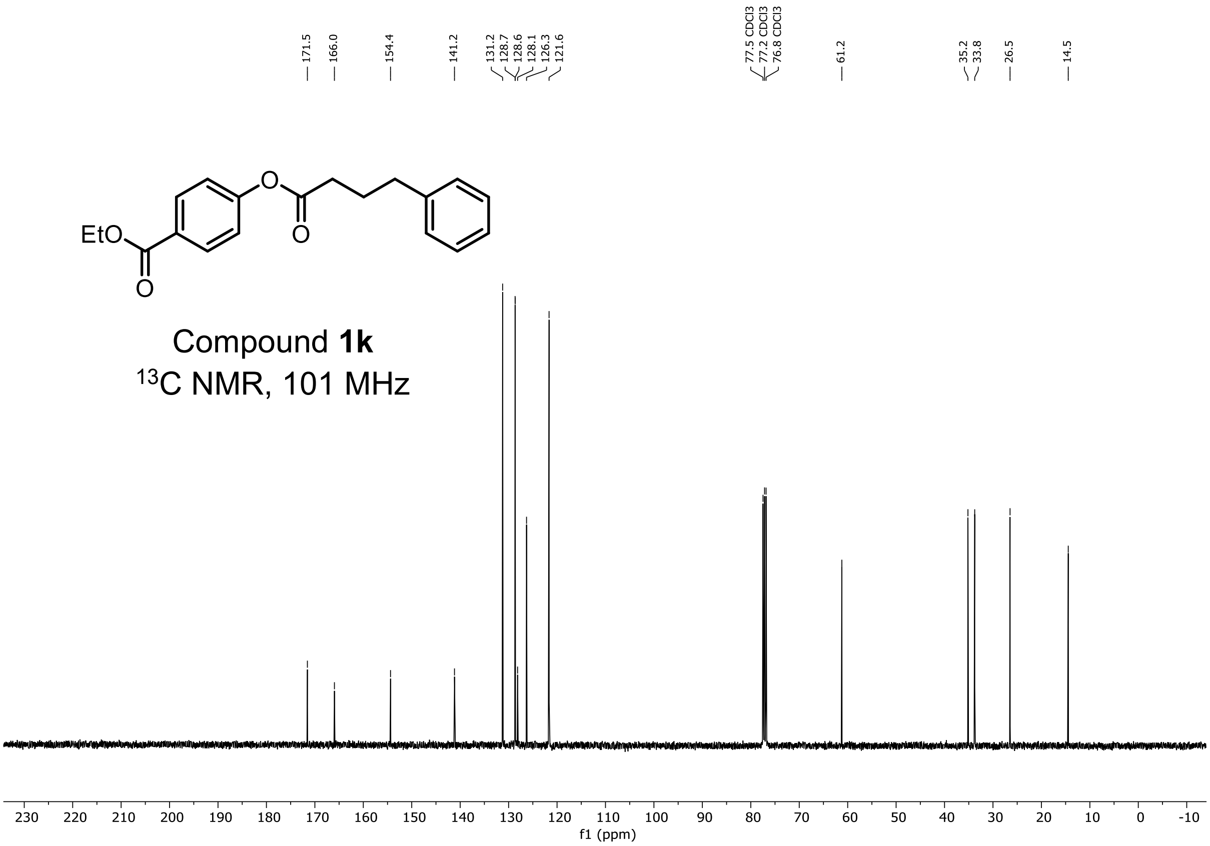
171.5 166.0 154.4 141.2 131.2 128.7 128.6 128.1 126.3 121.6 77.5 CDCl3 77.2 CDCl3 76.8 CDCl3 61.2 35.2 33.8 26.5 14.5
EtO
O
O
O
Compound **1k**
^{13}C NMR, 101 MHz
230 220 210 200 190 180 170 160 150 140 130 120 110 100 90 80 70 60 50 40 30 20 10 0 -10
f1 (ppm)

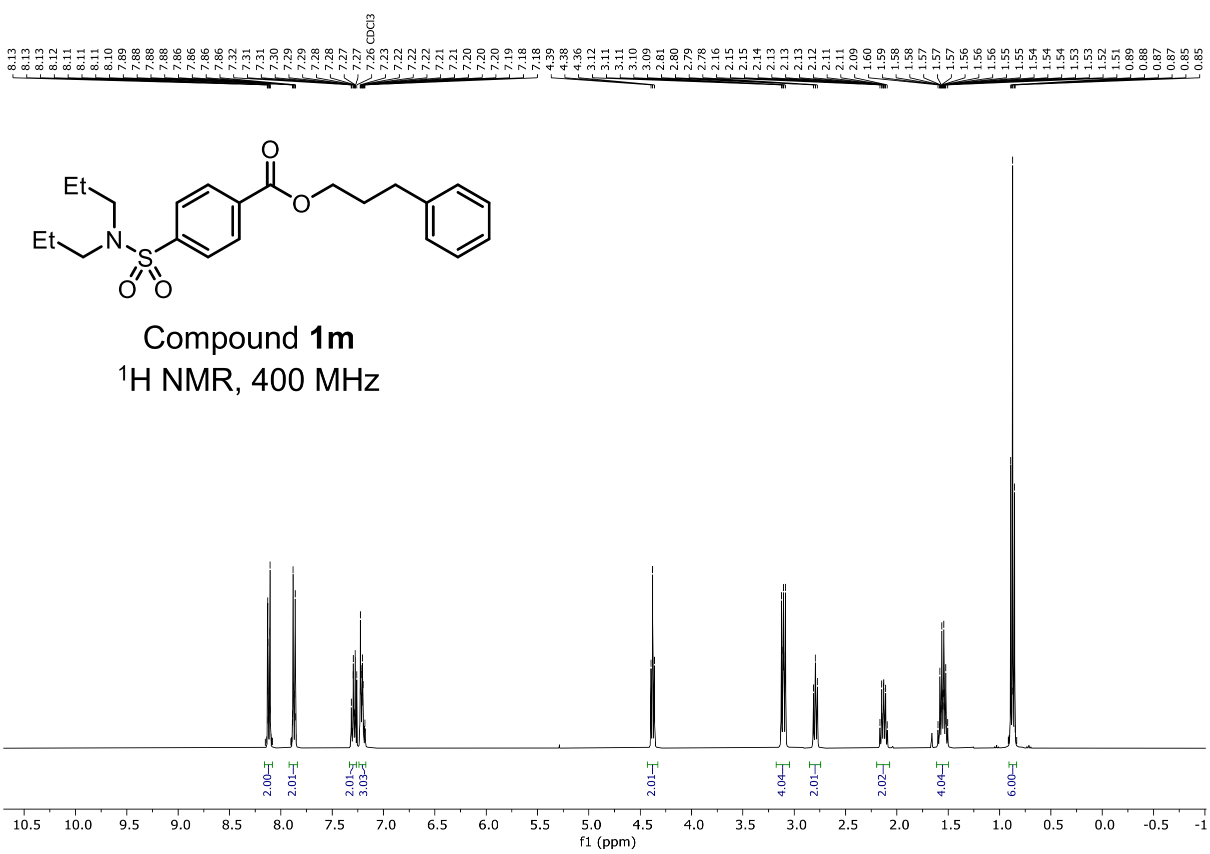
Et
Et
N
S
O
O
O
O
Compound **1m**
^{1}H NMR, 400 MHz
2.00
2.01
2.01
3.03
2.01
4.04
2.01
2.02
4.04
6.00
f1 (ppm)

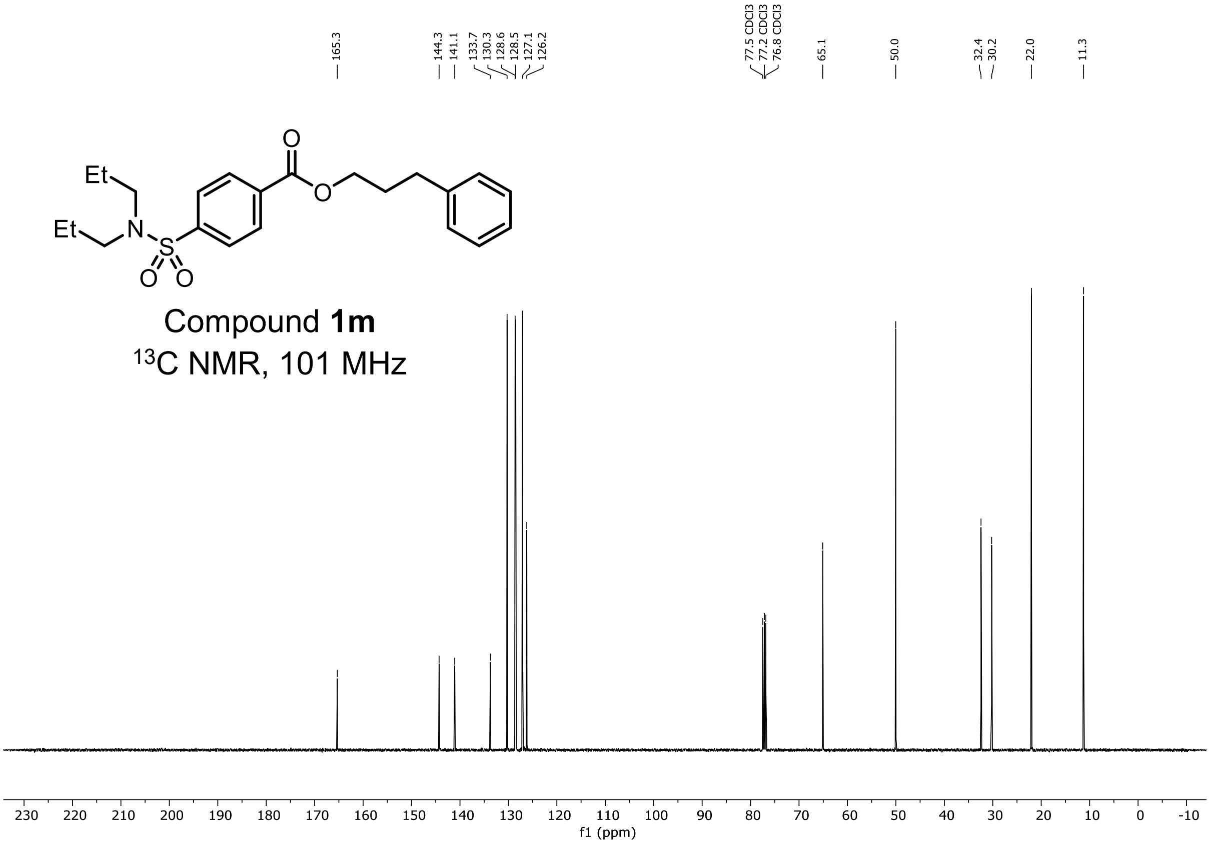
165.3
144.3
141.1
133.7
130.3
128.6
128.5
127.1
126.2
77.5 CDCl3
77.2 CDCl3
76.8 CDCl3
65.1
50.0
32.4
30.2
22.0
11.3
Et
Et
N
S
O
O
O
O
Compound **1m**
^{13}C NMR, 101 MHz
f1 (ppm)

18. X-Ray crystallographic data

Compound 4i:

1-((3r,5r,7r)-Adamantan-1-yl)-2-bromoethan-1-one

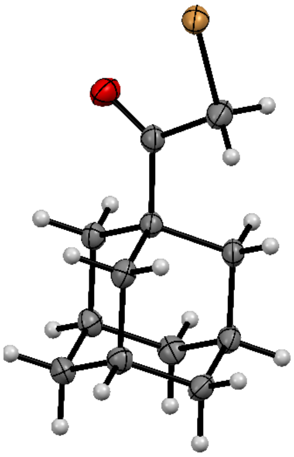

CCDC 2404075

Bond precision:	C–C = 0.0029 A		Wavelength = 1.54184
Cell:	a=9.9025(1)	b=9.9120(1)	c=22.0122(2)
	alpha=90	beta=90	gamma=90
Temperature:	100 K		

	Calculated	Reported
Volume	2160.58(4)	2160.58(4)
Space group	P b c a	P b c a
Hall group	-P 2ac 2ab	-P 2ac 2ab
Moiety formula	C12 H17 Br O	C12 H17 Br O
Sum formula	C12 H17 Br O	C12 H17 Br O
Mr	257.16	257.16
Dx,g cm–3	1.581	1.581
Z	8	8
Mu (mm–1)	4.885	4.885
F000	1056.0	1056.0

F000’	1052.64	
h,k,lmax	12,12,27	12,12,27
Nref	2224	2182
Tmin,Tmax	0.759,0.920	0.752,0.971
Tmin’	0.608	

Correction method= # Reported T Limits: Tmin=0.752 Tmax=0.971
AbsCorr = GAUSSIAN

Data completeness= 0.981 Theta(max)= 74.885

R(reflections)= 0.0242(2018) wR2(reflections)= 0.0631(2182)
S = 1.079 Npar = 127

19. References

1. (a) Jerschow, A.; Müller, N., 3D Diffusion-Ordered TOCSY for Slowly Diffusing Molecules. *Journal of Magnetic Resonance, Series A* **1996,** *123*, 222-225; (b) Jerschow, A.; Müller, N., Suppression of Convection Artifacts in Stimulated-Echo Diffusion Experiments. Double-Stimulated-Echo Experiments. *Journal of Magnetic Resonance* **1997,** *125*, 372-375; (c) Connell, M. A.; Bowyer, P. J.; Adam Bone, P.; Davis, A. L.; Swanson, A. G.; Nilsson, M.; Morris, G. A., Improving the accuracy of pulsed field gradient NMR diffusion experiments: Correction for gradient non-uniformity. *Journal of Magnetic Resonance* **2009,** *198*, 121-131.
2. Jelier, B. J.; Tripet, P. F.; Pietrasiak, E.; Franzoni, I.; Jeschke, G.; Togni, A., Radical Trifluoromethoxylation of Arenes Triggered by a Visible-Light-Mediated N−O Bond Redox Fragmentation. *Angew. Chem. Int. Ed.* **2018,** *57*, 13784-13789.
3. Fischer, D. M.; Lindner, H.; Amberg, W. M.; Carreira, E. M., Intermolecular Organophotocatalytic Cyclopropanation of Unactivated Olefins. *J. Am. Chem. Soc.* **2023,** *145*, 774-780.
4. Akkerman, Q. A.; Nguyen, T. P. T.; Boehme, S. C.; Montanarella, F.; Dirin, D. N.; Wechsler, P.; Beiglböck, F.; Rainò, G.; Erni, R.; Katan, C.; Even, J.; Kovalenko, M. V., Controlling the nucleation and growth kinetics of lead halide perovskite quantum dots. *Science* **2022,** *377*, 1406-1412.
5. (a) Protesescu, L.; Yakunin, S.; Bodnarchuk, M. I.; Krieg, F.; Caputo, R.; Hendon, C. H.; Yang, R. X.; Walsh, A.; Kovalenko, M. V., Nanocrystals of Cesium Lead Halide Perovskites (CsPbX3, X = Cl, Br, and I): Novel Optoelectronic Materials Showing Bright Emission with Wide Color Gamut. *Nano Lett.* **2015,** *15*, 3692-3696; (b) Morad, V.; Stelmakh, A.; Svyrydenko, M.; Feld, L. G.; Boehme, S. C.; Aebli, M.; Affolter, J.; Kaul, C. J.; Schrenker, N. J.; Bals, S.; Sahin, Y.; Dirin, D. N.; Cherniukh, I.; Raino, G.; Baumketner, A.; Kovalenko, M. V., Designer phospholipid capping ligands for soft metal halide nanocrystals. *Nature* **2024,** *626*, 542-548.
6. (a) MØLler, C. K., Crystal Structure and Photoconductivity of Cæsium Plumbohalides. *Nature* **1958,** *182*, 1436-1436; (b) Murtaza, G.; Ahmad, I., First

principle study of the structural and optoelectronic properties of cubic perovskites CsPbM3 (M=Cl, Br, I). *Physica B: Condensed Matter* **2011,** *406*, 3222-3229; (c) Jiang, L. Q.; Guo, J. K.; Liu, H. B.; Zhu, M.; Zhou, X.; Wu, P.; Li, C. H., Prediction of lattice constant in cubic perovskites. *J. Phys. Chem. Solids* **2006,** *67*, 1531-1536.

7. Findeisen, M.; Berger, S., *50 and more essential NMR experiments: a detailed guide*. John Wiley & Sons: 2013.
8. Gaspar, B.; Carreira, E. M., Mild Cobalt-Catalyzed Hydrocyanation of Olefins with Tosyl Cyanide. *Angew. Chem. Int. Ed.* **2007,** *46*, 4519-4522.
9. (a) Zhang, B.; Li, T.-T.; Mao, Z.-C.; Jiang, M.; Zhang, Z.; Zhao, K.; Qu, W.-Y.; Xiao, W.-J.; Chen, J.-R., Enantioselective Cyanofunctionalization of Aromatic Alkenes via Radical Anions. *J. Am. Chem. Soc.* **2024,** *146*, 1410-1422; (b) Hensinger, M. J.; Closs, A. C.; Trapp, O.; Ofial, A. R., The effect of S-alkylation on organocatalytic enamine activation through imidazolidine-4-thiones. *Chem. Commun.* **2023,** *59*, 8091-8094.
10. (a) Bailleux, S.; Dréan, P.; Zelinger, Z.; Civiš, S.; Ozeki, H.; Saito, S., Millimeter wave spectrum of bromomethyl radical, CH2Br. *The Journal of Chemical Physics* **2005,** *122*; (b) Haakansson, C. T.; Corkish, T. R.; Watson, P. D.; McKinley, A. J.; Wild, D. A., The bromide-bromomethyl radical dimer complex: Anion photoelectron spectroscopy and CCSD(T) calculations. *Chem. Phys. Lett.* **2020,** *761*, 138060.
11. Verschueren, R. H.; Voets, L.; Saliën, J.; Balcaen, T.; De Borggraeve, W. M., Solvent-Free Hydrohalogenation and Deuteriohalogenation by Ex Situ Generation of HX and DX Gas. *Eur. J. Org. Chem.* **2023,** *26*, e202300785.
12. Wicker, G.; Zhou, R.; Schoch, R.; Paradies, J., Sigmatropic [1,5] Carbon Shift of Transient C3 Ammonium Enolates. *Angew. Chem. Int. Ed.* **2022,** *61*, e202204378.
13. Ruos, M. E.; Kinney, R. G.; Ring, O. T.; Doyle, A. G., A General Photocatalytic Strategy for Nucleophilic Amination of Primary and Secondary Benzylic C–H Bonds. *J. Am. Chem. Soc.* **2023,** *145*, 18487-18496.
14. Li, C.; Zhang, Y.; Sun, Q.; Gu, T.; Peng, H.; Tang, W., Transition-Metal-Free Stereospecific Cross-Coupling with Alkenylboronic Acids as Nucleophiles. *J. Am. Chem. Soc.* **2016,** *138*, 10774-10777.

15. López-Pérez, A.; Adrio, J.; Carretero, J. C., Palladium-Catalyzed Cross-Coupling Reaction of Secondary Benzylic Bromides with Grignard Reagents. *Org. Lett.* **2009,** *11*, 5514-5517.

16. Keaveney, S. T.; White, B. P.; Haines, R. S.; Harper, J. B., The effects of an ionic liquid on unimolecular substitution processes: the importance of the extent of transition state solvation. *Organic & Biomolecular Chemistry* **2016,** *14*, 2572-2580.

17. Ding, R.; Li, J.; Jiao, W.; Han, M.; Liu, Y.; Tian, H.; Sun, B., A Highly Efficient Method for the Bromination of Alkenes, Alkynes and Ketones Using Dimethyl Sulfoxide and Oxalyl Bromide. *Synthesis* **2018,** *50*, 4325-4335.
